# Reversible photolithography of quantum dots achieves high-performance electroluminescence

**DOI:** 10.1038/s41467-026-77916-z

**Published:** 2026-09-24

**Authors:** Chang Gu, Zhixin Zhai, Shuo Ding, Changfeng Han, Ting Zhang, Chaoyu Xiang

**Affiliations:** 1 Laboratory of Optoelectronic Information Technology and Devices, Ningbo Institute of Materials Technology and Engineering, Chinese Academy of Sciences, Ningbo, China; 2 Hangzhou Bay Laboratory of Advanced Nano-Optoelectronic Materials and Devices, Qianwan Institute of CNITECH, Ningbo, China

**Keywords:** Quantum dots, Electronic devices, Quantum dots

## Abstract

Direct photolithography offers a transformative photoresist-free route for high-precision integration in electronic and optoelectronic platforms. Despite significant advancements in quantum dots (QDs), existing methods often degrade optoelectronic performance due to the inherent surface ligand changes during photolithographic processing. Here, we introduce a reversible photolithography strategy to overcome this bottleneck. This strategy is based on the first wavelength-gated, reversible ligand crosslinking and decrosslinking cycle, enabling the dynamic and reversible control over the photolithographic process. Using this approach, we achieve high-efficient and high-fidelity QD photolithography with restored QD structure and functionality. The reversible process further enables high-performance electroluminescence, with one of the highest luminance values of 125,016 cd m^−2^ at 5.0 V and a peak external quantum efficiency (EQE) of 24.41%. Moreover, we demonstrate an ultrahigh-resolution nano-pixelated device with a peak EQE of 17.08% at 21,000 PPI. This work presents a photo-reversible crosslinking system that decouples photolithographic patterning from performance degradation, offering a versatile platform for high-end QD-based displays and other advanced optoelectronics.

## Abstract (web summary)

Direct quantum dot photolithography can impair performance by altering surface ligands. Gu et al developed reversible, wavelength-controlled ligand crosslinking to produce high-resolution, efficient light-emitting devices while restoring quantum dot structure and function.

## Introduction

The relentless drive toward miniaturization and high integration in electronic and optoelectronic devices demands patterning techniques that combine high resolution with structural fidelity^1–3^. Conventional photoresist-based photolithography, while fundamental to silicon-based semiconductor and integrated circuit manufacturing^4,5^, suffers from inadequate chemical orthogonality and poor compatibility with advanced optoelectronic functional materials, such as inorganic nanomaterials and organic semiconductors, often leading to severe performance degradation^6–9^. Direct photolithography (also known as direct photo-patterning) has therefore emerged as a transformative, photoresist-free platform that streamlines fabrication protocols while preserving material integrity and functionality^10–12^.

Colloidal quantum dots (QDs) are key emitters for next-generation displays owing to their tunable emission, high color purity, and excellent photoluminescence quantum yield (PL QY)^13,14^. Recently, direct photolithography of QDs has been successfully demonstrated through tailored surface ligand chemistry^15–25^, enabling high-precision QD patterns and corresponding light-emitting diodes (QLEDs) for promising near-eye virtual/augmented reality interactions, chip-scale displays, and artificial intelligence interfaces^26–32^. Unfortunately, while peak external quantum efficiencies (EQEs) exceeding 20% are commonly achieved in mainstream QD platforms^14^, most photolithographic devices still fall below this threshold^29^. Achieving high-performance electroluminescence after photolithographic processing thus lies at the core of ongoing research in the field. Specifically, despite considerable efforts in photosensitive wavelength regulation^33^, functional unit/skeleton engineering^34,35^, and compatibility optimization^36^, the degradation of QD optoelectronic performance remains a serious concern.

The primary cause lies in the inherent surface ligand changes of QDs during photolithographic processing (e.g., ligand decomposition, exchange, or crosslinking, along with the resulting harmful photochemical products)^22^, which can readily induce adverse effects^26–28^. For instance, compact ionic ligands generated from ligand decomposition are less effective at passivating surface traps^29^. Ligand exchange processes mediated by photoacid generators often suffer from acid-induced degradation and the formation of surface defects^19^. Highly crosslinked ligand networks tend to increase electrical resistance^24,35,37^. However, such surface ligand changes are precisely the core mechanism that enables photolithographic patterning. This creates an intrinsic coupling between patterning capability and performance degradation, a trade-off that remains fundamentally unresolvable within the framework of reported approaches. Given this situation, restoring the structure and optoelectronic performance of QDs after photolithography has emerged as the most promising route forward^18,24^. However, the post-photolithographic film-state ligand exchange approach^31,38–40^, while effective, is invasive and challenging to control precisely. A straightforward strategy that can restore the surface ligand states of QDs is highly desirable for achieving high-performance electroluminescence.

**Fig. 1:**
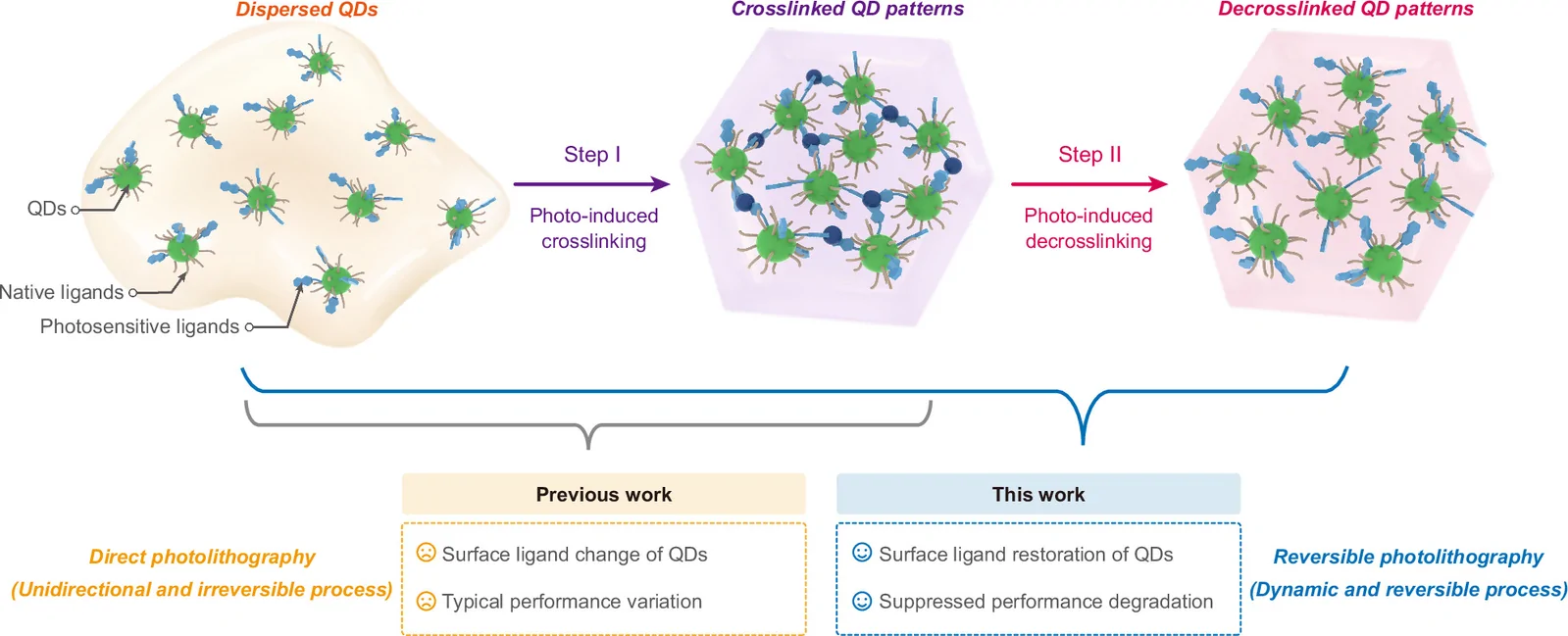
Reversible photolithography strategy for QDs. Schematic of direct photolithography (previous work, represented by ligand crosslinking approaches) and reversible photolithography (this work) of QDs.

Here, we introduce a reversible photolithography strategy to directly address the coupled challenge in photolithographic patterning and optoelectronic performance (Fig. 1). The strategy employs a rationally designed coumarin-based photo-reversible ligand for reversible crosslinking and decrosslinking cycles between neighboring QDs. The reversible process enables highly efficient QD photolithography with a high PL QY retention of 107.4% at an ultralow energy dose. It further restores the structure and functionality of QDs, thereby achieving high-performance electroluminescence with restored luminance, current density, EQE, and operational lifetime. As a result, the as-prepared QLED demonstrates an attractive luminance of 125,016 cd m^−2^ at 5.0 V and a peak EQE of 24.41%. Furthermore, we also fabricate a compelling ultrahigh-resolution nano-pixelated QLED with a peak EQE of 17.08% at 21,000 PPI. These results overcome the long-standing challenge of damage associated with direct photolithography and establish a QD micro-/nanofabrication pathway for high-definition QD-based displays.

## Results

### Design of reversible photolithography of QDs

Existing direct photolithography of QDs, represented by the most promising ligand crosslinking approaches, typically induces variations in optoelectronic performance due to surface ligand changes caused by the inherent irreversibility of the photolithographic process. Moreover, in many representative studies in the field, such variations are often observed to result in performance degradation^32–35,37^. To address these challenges, we introduce a reversible photolithography strategy of QDs for the dynamic and reversible control over the photolithographic process. As shown in Fig. 1, this strategy comprises two accessible steps. In the first step, a photo-induced crosslinking process between neighboring QDs is applied for direct QD photo-patterning. In the second step, a photo-induced decrosslinking process is applied to restore the surface ligands of QDs and suppress optoelectronic performance degradation. These steps must be sufficiently efficient so that they require ultralow energy doses to minimize photodamage to QDs, thereby ensuring high-fidelity and high-resolution patterning. Recently, Zhang et al. reported a reversible photoisomerization process using an azobenzene-functionalized ligand^41^, which is conceptually feasible for reversible photolithography. However, this system suffers from unfavorable energy transfer and hole transfer, arising from the intrinsic visible-light absorption of azobenzene and its energy level misalignment with QDs.

**Fig. 2:**
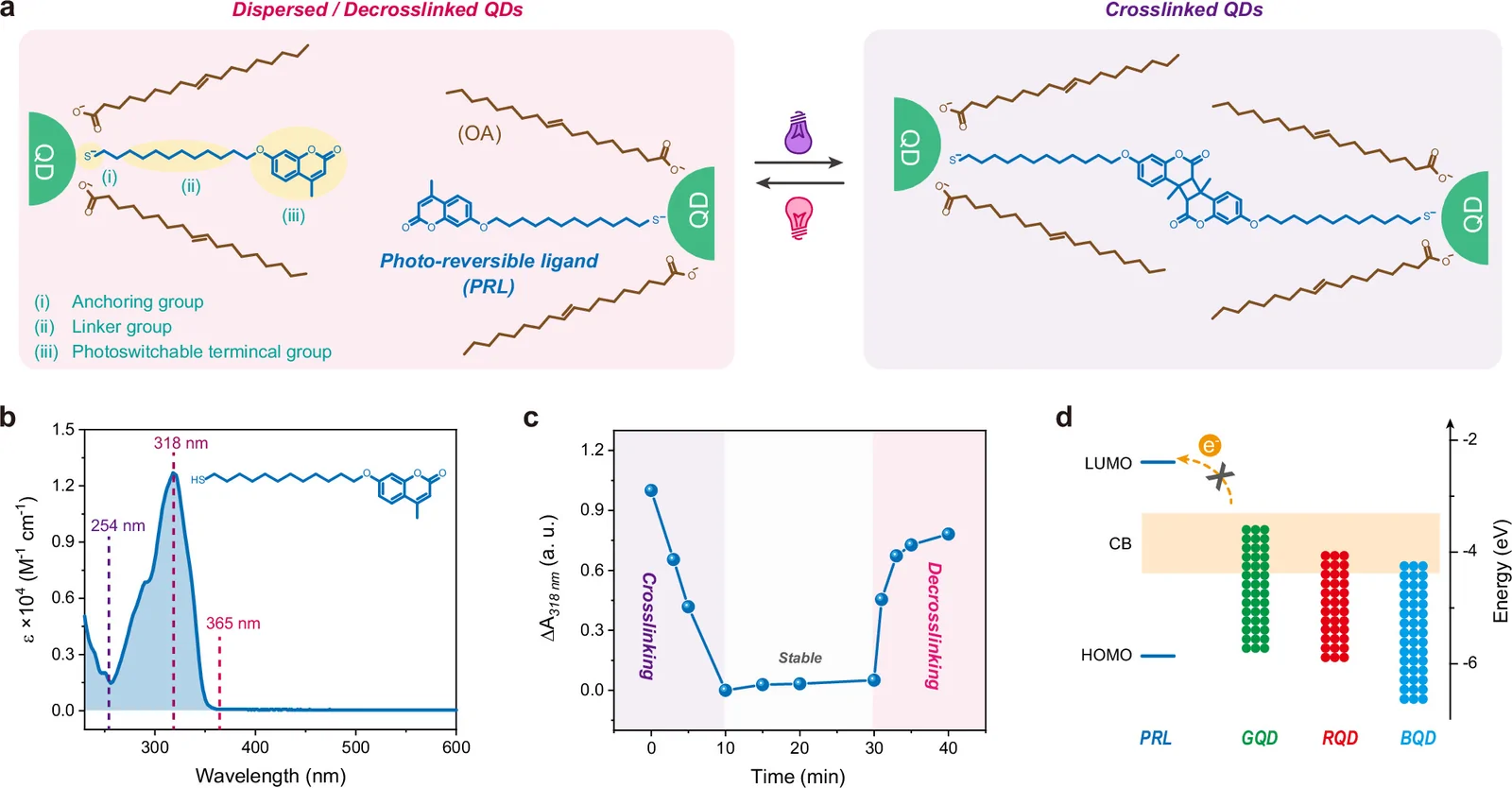
Design of reversible photolithography of QDs via PRL. **a** Schematic of reversible photocycloaddition of PRL for reversible crosslinking of QDs. **b** *ε* of PRL in CH_3_CN. **c** Relative absorption changes at 318 nm in the crosslinking, stable, and decrosslinking processes. 310 nm UV exposure (~5 mW cm^−2^) and 254 nm UV exposure (~10 mW cm^−2^) are used for the crosslinking and decrosslinking processes, respectively. **d** Schematic of electronic energy level alignments between PRL and commercial CdSe-based RGB QDs.

Coumarin derivatives exhibit a reversible [2 + 2] photocycloaddition reaction under wavelength-selective irradiation (Fig. 2a): long-wavelength UV irradiation (>300 nm) induces efficient and site-specific cyclization to form stable cyclobutane adducts, while subsequent short-wavelength UV irradiation (<300 nm) enables structural reversion through photocleavage^42^. Meanwhile, the intrinsic photoinitiator-free and catalyst-free nature of this reversible transformation contributes to exceptional chemical purity and precise spatiotemporal control. These features provide a foundation for developing a promising strategy toward high-fidelity photolithographic outcomes. Unfortunately, although this photochemistry has demonstrated multiple applications (e.g., drug delivery systems, shape-memory polymers)^43–46^, its implementation in direct photolithography of QDs remains unexplored.

Inspired by this, we designed and synthesized a coumarin-functionalized photo-reversible ligand (termed PRL) to achieve reversible photolithography of QDs. As shown in Fig. 2a, PRL integrates a coumarin moiety as the photoswitchable terminal group for wavelength-gated crosslinking and decrosslinking cycles, coupled with a thiol anchoring group that facilitates robust coordination to QDs^47^. This thiol functionality concurrently promotes effective passivation of surface trap states, which is verified later. Meanwhile, an extended alkyl linker group further ensures colloidal stability of PRL-capped QDs in low-polarity processing solvents (e.g., toluene)^48^, thereby supporting uniform thin-film formation for solution-processed QLEDs. Synthetic procedures and characterization of PRL are detailed in Supplementary Note 3.

PRL exhibits a distinct absorption maximum at 318 nm with a high molar extinction coefficient (*ε*) of 1.27 × 10^4^ L mol^−1^ cm^−1^ (Fig. 2b), demonstrating its attractive photosensitivity. The reversible photocyclization behavior of PRL is quantified via in situ UV–Vis absorption analysis. Upon irradiation with a handheld UV LED lamp (centered at 310 nm), the absorption intensity decreases significantly (Fig. 2c), consistent with the formation of cyclobutane adducts, which is a photocyclization/crosslinking hallmark of coumarin derivatives^49,50^. The cyclobutane adducts remain stable but could be reverted to the original state through short-wavelength UV irradiation. Here, a deep-UV (DUV) LED lamp (centered at 254 nm) is employed, owing to its structural simplicity and cost-effectiveness, as well as its ready extendability to the commercially more prevalent 248 nm KrF lithography systems. These phenomena confirm the reversible photo-crosslinking capacity of PRL for reversible QD photolithography.

In addition, thermodynamic considerations require that the conduction band (CB) of the QDs lies below the lowest unoccupied molecular orbital (LUMO) of the photosensitive materials. Electrochemical characterization confirms that the LUMO of PRL is positioned at −2.41 eV (Supplementary Note 4 and Supplementary Fig. 1), which lies energetically above the CBs of commercial CdSe-based core-shell red-, green-, and blue-emitting QDs (RQD: −4.00 eV; GQD: −3.54 eV; and BQD: −4.16 eV) (Fig. 2d). This favorable energy-level alignment is critical for suppressing charge trapping and luminescence quenching^32^.

### High-efficient and high-fidelity photolithography of QDs

**Fig. 3:**
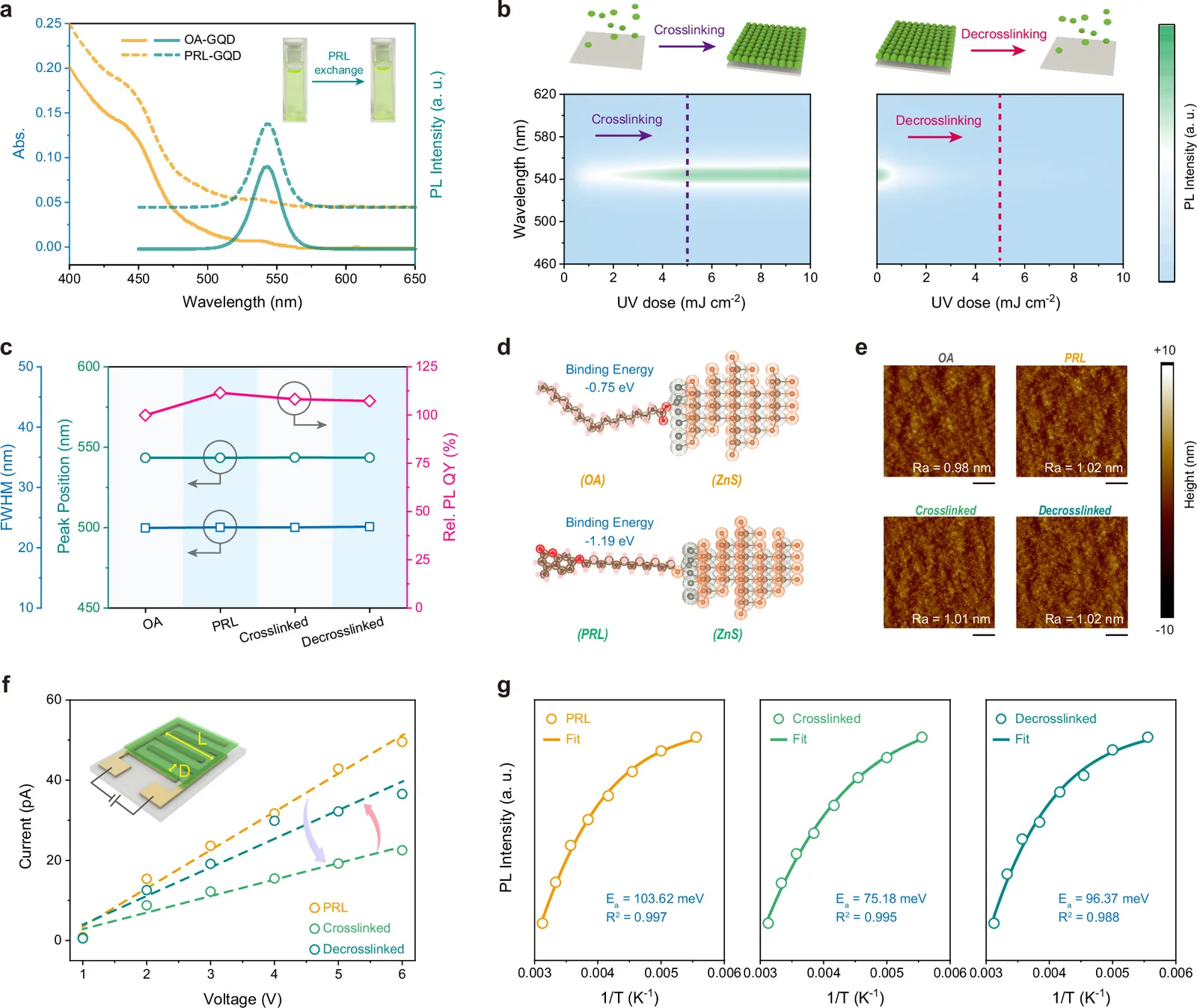
High-efficient and high-fidelity photolithography of QDs via PRL. **a** UV–Vis absorption and PL spectra of OA-GQD and PRL-GQD in toluene. Inset: photos of the OA-GQD solution before and after PRL exchange. **b** Schematic and normalized PL intensities of PRL-GQD films under the crosslinking process (left) and the decrosslinking process (right) with different exposure doses. UV lamps (310 nm, ~0.5 mW cm^−2^ for the crosslinking process; 254 nm, ~1 mW cm^−2^ for the decrosslinking process) and toluene development are used here. **c** PL peak position, FWHM, and relative PL QYs of OA-GQD, PRL-GQD, crosslinked PRL-GQD, and decrosslinked PRL-GQD. **d** Theoretical simulation of the binding energy of OA or PRL with ZnS on the QD surface. **e** AFM images of GQD films under different reversible photolithography processes. Scale bar: 200 nm. **f** Schematic and current–voltage analysis of PRL-GQD, crosslinked PRL-GQD, and decrosslinked PRL-GQD using a lateral device structure with a stripe length (*L*) of 6.8 mm and channel width (*D*) of 100 μm. **g** The integrated PL intensities of PRL-GQD (left), crosslinked PRL-GQD (middle), and decrosslinked PRL-GQD (right) as a function of reciprocal temperature.

Taking commercial green-emitting QDs capped with oleic acid (OA) and oleylamine (OAm) ligands (termed OA-GQD) as an example, an efficient ligand exchange process is carried out (Supplementary Note 5), yielding QDs tailored with PRL (termed PRL-GQD). The ligand composition is quantitatively monitored via ¹H-NMR spectroscopy. As shown in Supplementary Fig. 2, OA-GQD exhibits characteristic OA signals (δ 5.33 ppm, CH=CH), whereas PRL-GQD displays additional signals from PRL moieties. Integration of the corresponding peak areas confirms the successful surface integration of PRL with a surface content of ~8 mol%. UV–Vis and photoluminescence (PL) spectra reveal no shift in absorption signals or emission signals before and after PRL exchange (Fig. 3a).

The designed reversible photolithography involves the following steps: (i) spin-coating of PRL-GQD solution onto a substrate; (ii) exposure to long-wavelength UV light (centered at 310 nm) to induce crosslinking; (iii) developing to remove uncrosslinked components, yielding a crosslinked film; and (iv) exposure to short-wavelength DUV (centered at 254 nm) to trigger decrosslinking. The detailed description and schematic of operation procedures are provided in the “Method” and Supplementary Fig. 3.

The feasibility of reversible photolithography was systematically investigated as a first step. As shown in Fig. 3b, unexposed PRL-GQD film (UV dose = 0 mJ cm^−2^) exhibits complete PL quenching after toluene development, ensuring high-contrast patterning. PL intensity increases sharply with UV dose, saturating at ~5 mJ cm^−2^. Notably, the ultralow dose is far below the doses typically required for commercial photoresists and semiconducting photoresists^51^ (80–150 mJ cm^−2^ and >200 mJ cm^−2^, respectively), and comparable to those of reported direct QD photolithography systems (Supplementary Table 1). In addition, the decrosslinking process could be achieved through ultralow-dose DUV exposure, represented by the reduction in PL intensities after toluene development (e.g., ~94% at 5 mJ cm^−2^) (Fig. 3b), highlighting the remarkable photochemical efficiency. Moreover, we performed atomic force microscopy (AFM) measurements to directly characterize the film thickness of PRL-GQD under different exposure doses. As shown in Supplementary Fig. 4, the resulting thickness data are highly consistent with the PL intensity trends, further confirming the reliability of the reversible photolithography process.

In situ Fourier transform infrared (FT‑IR) spectroscopy was employed to monitor the switching of coumarin ligands during the photochemical reactions (Supplementary Fig. 5). Upon 310 nm UV exposure, the signal at 1614 cm^−1^, corresponding to the C=C stretching vibration, gradually decreased, consistent with the consumption of coumarin groups during crosslinking^45,46^. Subsequently, upon DUV irradiation, this signal gradually recovered, reflecting the restoration of coumarin groups through the decrosslinking process. This reversible behavior was further confirmed by in situ UV–Vis absorption spectroscopy, which showed reversible changes in the absorbance of PRL (Supplementary Fig. 6).

To assess the contribution of photo-crosslinking between ligands on neighboring QDs (the inter‑QD pathway) to reversible QD photolithography, we examined the crosslinking kinetics of QD solutions at different concentrations (0.10 and 0.02 mg mL^−1^). As shown in Supplementary Fig. 7, the normalized relative absorbance changes show a clear increase with rising QD concentration—a concentration‑dependent trend that is characteristic of the inter‑QD pathway. More importantly, under the same irradiation conditions, the QD film exhibits larger relative absorbance changes, indicating a significant inter-QD crosslinking contribution arising from the close proximity of neighboring QDs.

Then, we systematically characterize the optical properties of QD films throughout the entire reversible photolithography workflow. PL spectra reveal that both the emission peak position and full width at half maximum (FWHM) remain unchanged (Supplementary Fig. 8). Notably, PL QY measurements reveal an 11.6% enhancement after PRL exchange (Fig. 3c), similar to the PL QY change in solution (Supplementary Fig. 9). Even after the complete reversible photolithography process, the relative PL QY remains as high as 107.4%, which is higher than that of the pristine OA-GQD, indicating effective suppression of non-radiative recombination pathways. This enhancement is attributed to robust thiol-ZnS shell coordination at the QD surface, which passivates surface trap states. X-ray photoelectron spectroscopy (XPS) verifies this mechanism. As shown in Supplementary Fig. 10, the Zn *2p* peak for PRL-GQD shifts to a higher binding energy, indicative of strong interfacial dipole formation and thiol-mediated surface passivation^52,53^. Theoretical simulation further supports this observation, revealing a more negative binding energy for PRL (−1.19 eV) compared to OA (−0.75 eV) (Fig. 3d and Supplementary Note 6). Here, the PL QY retention after the complete reversible photolithography process remains as high as 96.2% even when referenced to PRL-GQD, indicating minimal photochemical damage of the QDs.

High-fidelity structural preservation of QDs is confirmed through surface morphology and compositional analyses. AFM data reveal that OA-GQD exhibits excellent film-forming properties with an ultralow surface roughness (arithmetic average height, Ra) of 0.98 nm (Fig. 3e). Remarkably, the Ra value increased only marginally to 1.02 nm after the entire reversible photolithography process, indicating minimal topographical disruption. XPS analysis further demonstrates that core elemental signatures of the QDs (e.g., Cd *3d*, Se *3d*) remain unchanged (Supplementary Fig. 11).

Charge transport conductivity, another key metric that influences the current density and luminance of QLED devices, was evaluated here using current–voltage analysis. As shown in Fig. 3f, a lateral device based on crosslinked PRL-GQD (termed Crosslinked) exhibited lower current than that based on PRL-GQD (termed PRL). Given the identical electrode configuration and film geometry, the lower current indicates higher resistance and thus reduced conductivity. Further analysis suggests that the reduced conductivity may arise from an increased inter-QD distance^37^. As supporting evidence, grazing-incidence small-angle X-ray scattering (GISAXS) measurements indicated that the photo-crosslinking process increased the inter-QD spacing from 8.42 to 8.70 nm (“Method” and Supplementary Fig. 12). Importantly, both the conductivity and the inter-QD distance are effectively restored after the photo-induced decrosslinking process.

We further performed temperature-dependent PL measurements on QD films during the reversible photolithography process. The thermal activation energy (*E*_*a*_) is quantitatively determined using Eq. 1, with the fitting results shown in Fig. 3g. The PRL-GQD film gave an *E*_*a*_ of 103.62 meV, whereas the crosslinked PRL-GQD film exhibited a significantly reduced value of 75.18 meV. Notably, the decrosslinking process effectively recovered *E*_*a*_ to 96.37 meV, close to its original value, further demonstrating the low‑damage nature of the reversible photolithography strategy.

1 $$I\left(t\right)=\frac{{I}_{0}}{1+Aexp \,\left(-\frac{{E}_{a}}{{k}_{B}T}\right)}$$

Given the substantially increased dipole moment and dielectric constant of coumarin after photocycloaddition^54^, the exciton binding energy (*E*_*b*_) is expected to undergo reversible modulation—decreasing upon crosslinking and recovering upon decrosslinking—due to the change in the local dielectric environment^55^. We therefore infer that the observed variation in *E*_*a*_ is closely related to the change in *E*_*b*_, given their similar trends. In this context, the reduced *E*_*a*_ after crosslinking reflects a compromised exciton binding capability, which ultimately impairs device performance, as confirmed by subsequent experiments. As a complement, we also performed time‑resolved transient absorption (TA) spectroscopy on the QD films (Supplementary Fig. 13). The lack of notable signal changes upon crosslinking and decrosslinking—similar to the trend observed in PL QYs (Fig. 3c)—suggests that surface defects and energy transfer pathways do not play a dominant role in the photochemical processes.

These results confirm that our reversible photolithography strategy not only achieves high-efficient and high-fidelity photolithography with well-maintained optical properties and structures of QDs, but overcomes the inherent degradation of the charge transport conductivity and exciton binding capability associated with direct photolithography, thereby enabling high-performance electroluminescence.

### High-performance QLEDs enabled by reversible photolithography

**Fig. 4:**
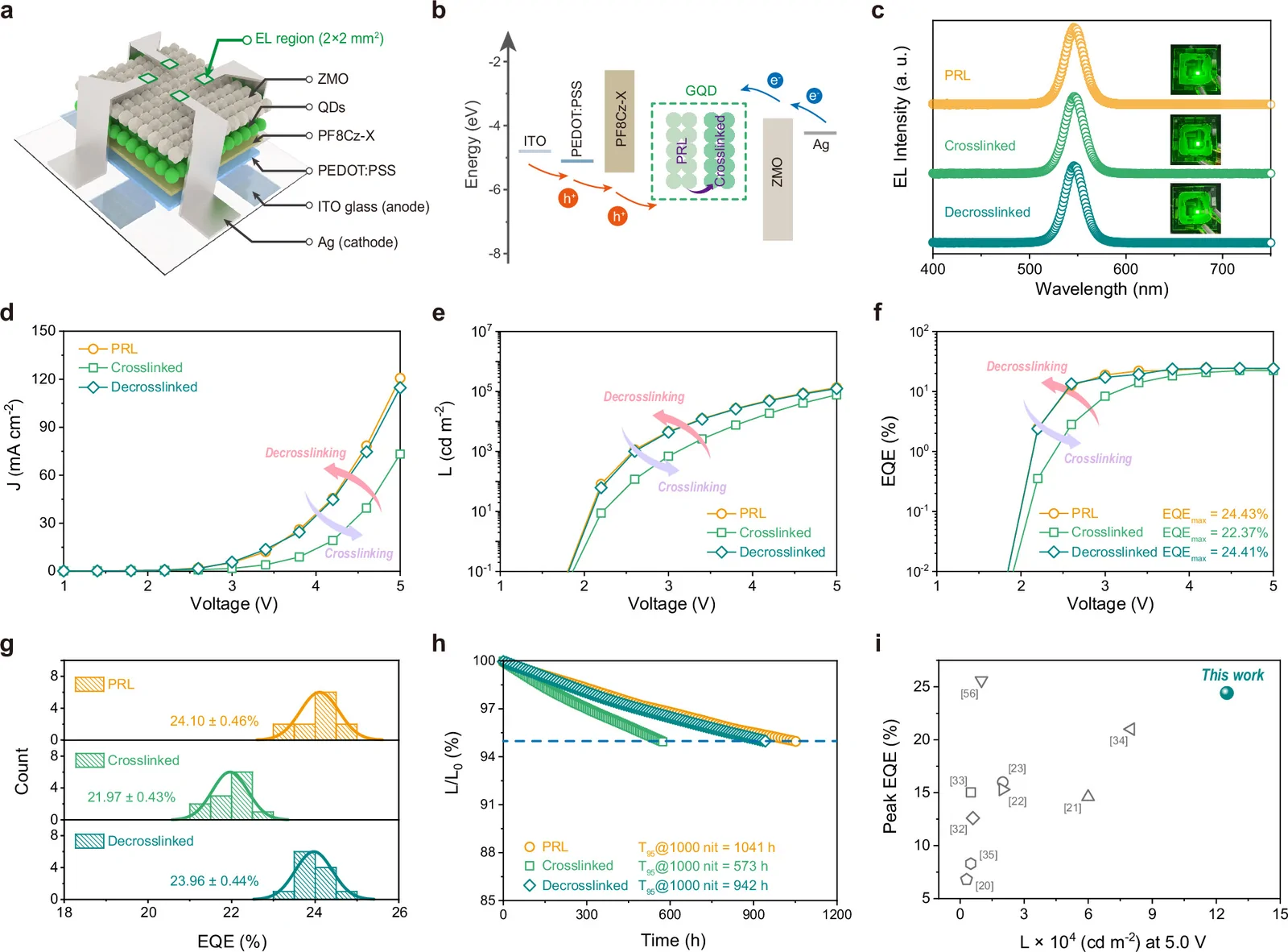
High-performance QLEDs enabled by reversible photolithography. **a** Schematic of the as-prepared QLED. **b** Energy band of the as-prepared QLED. **c**–**f** Normalized EL spectra, *J–V* curves, *L–V* curves, and EQE*–V* curves of QLEDs based on PRL-GQD (PRL), crosslinked PRL-GQD (Crosslinked), and decrosslinked PRL-GQD (Decrosslinked). Inset in **c**: EL photos of corresponding QLEDs at 5.0 V. **g** Histograms of peak EQE values obtained from 12 QLEDs, respectively. **h** Operating lifetime curves of corresponding QLEDs. **i** Comparison of *L* at 5.0 V and peak EQE of photolithographic light-emitting prototypes^20–23,32–35,56^.

Electroluminescence (EL) properties of QLEDs based on reversible photolithographic QDs were systematically measured to evaluate their potential in light-emitting display applications. The QLED based on PRL-GQD was selected as the control. In the device architecture, commercially available PEDOT:PSS, thermal-crosslinked PF8Cz (PF8Cz-X), and Mg-doped zinc oxide nanoparticles (ZMO) are employed as the hole injection layer (HIL), hole transport layer (HTL), and electron transport layer (ETL), respectively (Fig. 4a and Supplementary Note 7). The energy band diagram of the fabricated QLEDs is listed in Fig. 4b. Corresponding energy levels were determined based on references, complemented by UV–Vis absorption measurements and ultraviolet photoelectron spectroscopy (Supplementary Fig. 14).

As shown in Fig. 4c, all QLEDs exhibited uniform green emission and consistent EL spectra without any peak position shift or broadening. However, the current density–voltage (*J–V*) and luminance–voltage (*L–V*) curves revealed an obvious reduction in both *J* and *L* after photo-crosslinking (Fig. 4d, e). For example, the *L* and *J* at 5.0 V decreased from 134,796 cd m^−2^ and 120.7 mA cm^−2^ to 76,633 cd m^−2^ and 73.2 mA cm^−2^, respectively, corresponding to retention rates of only 57 and 61%. This performance trade-off is also observed in other systems based on direct QD photolithography via photo-crosslinking^34,56–58^. And it is primarily attributed to the reduced conductivity within crosslinked QD networks, as demonstrated by others^24,35,37,39^ and confirmed in this work (Fig. 3f). Meanwhile, EQE*–V* curves revealed a slight decrease in peak EQE from 24.43 to 22.37% after crosslinking (Fig. 4f). This phenomenon was more pronounced at low operating voltages (e.g., 12.60% vs. 2.82% at 2.6 V), correlating with the reduced exciton binding energy of the QD films. Here, to address concerns regarding inter-batch repeatability, the statistical distribution of peak EQEs across 12 devices was evaluated (Fig. 4g), indicating a consistent trend (24.10 ± 0.46% and 21.97 ± 0.43%, respectively). The operational lifetime, defined as the time required for the luminance to decay to 95% at an initial *L* of 1000 cd m^−2^ (*T*_*95*_@1000 cd m^−2^), was also reduced from 1041 to 573 h, attributed to the decreased EL efficiency (Fig. 4h).

In this case, our reversible photolithography strategy addresses the challenge of performance degradation through an in situ photo-induced decrosslinking process. As demonstrated in Fig. 4d, e, QLEDs based on decrosslinked PRL-GQD show significant restoration in *L* and *J*, restoring values to 125,016 cd m^−2^ and 114.7 mA cm^−2^ at 5.0 V, which corresponded to improved *L* and *J* retention rates of 93% and 95%, respectively. Both the EL efficiency and the operational lifetime of the decrosslinked QLED were restored also, with a peak EQE of 24.41% (average 23.96 ± 0.44%) and an extended *T*_*95*_@1000 cd m^−2^ of 942 h (Fig. 4f–h). To our knowledge, this value represents one of the best results among reported photolithographic light-emitting prototypes (Fig. 4i and Supplementary Table 1). A minor drawback is that the EL restoration is not 100%, due to the reversible photocleavage equilibrium of coumarin groups under 254 nm irradiation^59^. We hope to overcome this by further molecular and system optimization in the future.

In addition, we also fabricated QLEDs based on pristine OA-GQD and characterized their EL performance. As shown in Supplementary Fig. 15, these devices exhibited a current density of 87.5 mA cm^−2^ and a luminance of 93,802 cd m^−2^ at 5.0 V, with a peak EQE of only 23.53%. These values are markedly lower than those of the decrosslinked PRL-GQD devices. These results fully validate the critical value of the reversible photolithography strategy in achieving high-performance QLEDs.

### High-resolution photolithographic patterning of QDs

**Fig. 5:**
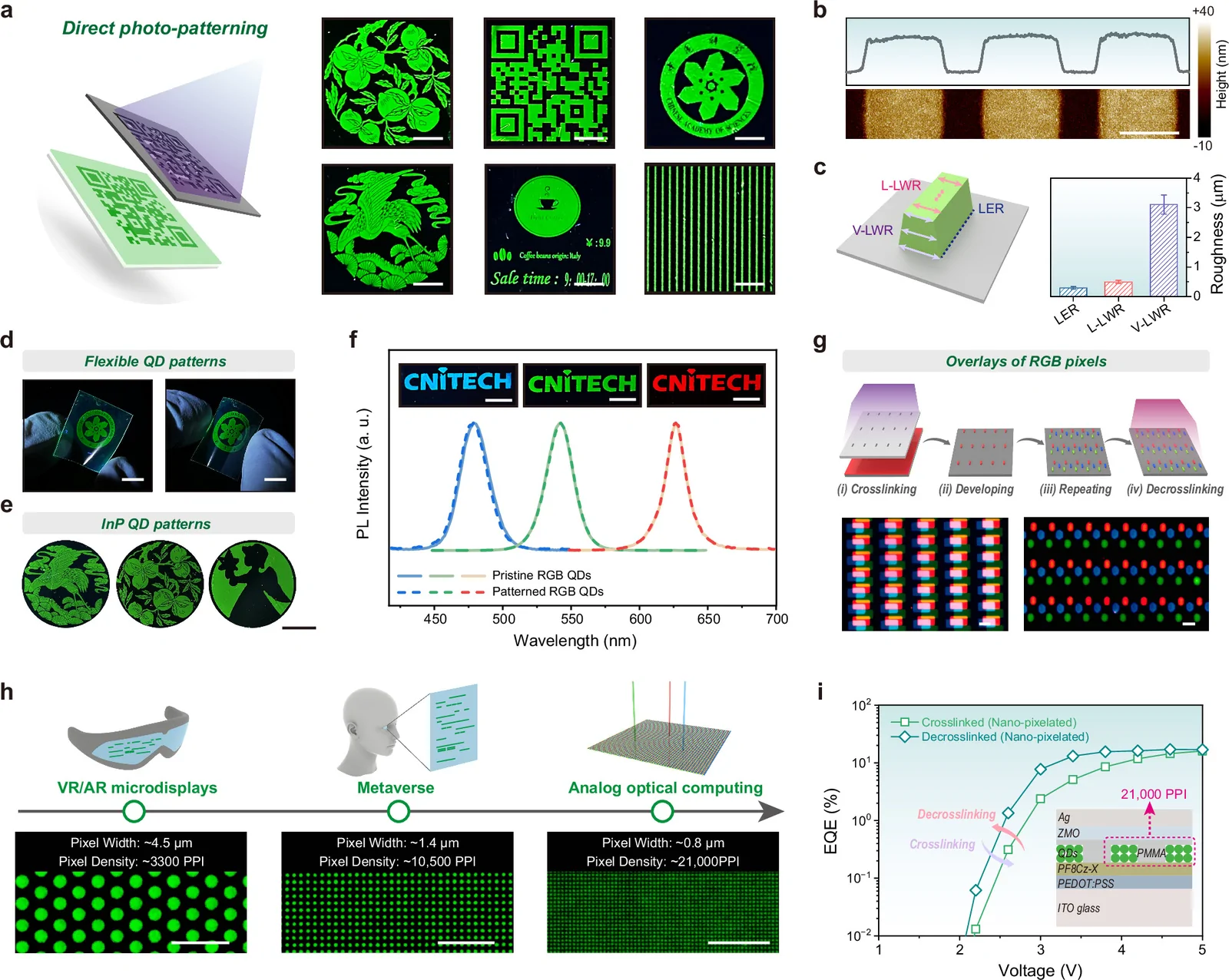
High-resolution photolithographic patterning of QDs. **a** Schematic (left) and fluorescent images (right) of direct photo-patterning of PRL-GQD. Scale bar: 5 mm. **b** AFM surface profile and image of patterned QDs. Scale bar: 10 μm. **c** Left: schematic of fidelity analysis. Right: averaged values of LER, L-LWR, and V-LWR. The data are averaged from six samples, and corresponding error bars represent the standard deviation (SD) of the data. **d** Fluorescent images of photolithographic PRL-GQD patterns on flexible PET substrates. Scale bar: 1 cm. **e** Fluorescent images of photolithographic InP QD patterns. Scale bar: 1 cm. **f** Normalized PL spectra of pristine and patterned RGB QD films. Inset: fluorescent images of RGB patterns. Scale bar: 5 mm. **g** Schematic of overlays of RGB pixels through reversible photolithography and corresponding fluorescent microscopic images. Scale bar: 20 μm. All results in (**a**–**g**) were obtained under ambient conditions and following the complete reversible photolithography workflow, including the decrosslinking step. **h** EL microscopic images of pixelated QLEDs with different photolithographic pixel width/density and their potential applications. Scale bar: 20 μm. **i** EQE*–V* curves of nano-pixelated QLEDs based on crosslinked PRL-GQD and decrosslinked PRL-GQD. Inset: schematic of nano-pixelated QLED with a PMMA blocking layer.

The PRL-mediated reversible photolithography also enables high-precision photo-patterning of QDs with cost-effective handheld UV-LED lamps under ambient conditions. For example, decorative patterns (e.g., flowers, cranes) and functional labels (e.g., QR codes, billboards, and logos) were developed with the help of photomasks (Fig. 5a). Although the PL QY retention after reversible photolithography under ambient conditions is relatively lower (~91.8% relative to OA-GQD; ~83.3% relative to PRL-GQD) than that achieved in the nitrogen-filled glovebox (Supplementary Fig. 16), likely due to UV-induced degradation of QDs assisted by water and oxygen, this result still highlights the potential of our strategy for low-cost processing in air—an attractive feature for future practical applications. Furthermore, the reversible photolithography process can be cycled more than six times (Supplementary Fig. 17).

The patterning quality is closely tied to the amount of PRL. As displayed in Supplementary Fig. 18, the PL intensity of QD patterns initially rose with increasing ligand content and then kept stable, whereas the edge sharpness progressively degraded. The ~8 mol% loading proved to be optimal. Under this parameter, the resulting QD patterns with a 12-μm line width exhibit a relatively flat-top surface and sharp edges, as revealed by AFM characterization (Fig. 5b). We further performed a quantitative fidelity analysis through three key metrics: line-edge roughness (LER), longitudinal line-width roughness (L-LWR), and vertical line-width roughness (V-LWR). All metrics were determined as three times the standard deviation (3*σ*) of the corresponding edge-position or line-width distributions, following established procedures^30,57^. As summarized in Fig. 5c, the measured LER, L-LWR, and V-LWR values are 0.29 ± 0.04 μm, 0.49 ± 0.05 μm, and 3.11 ± 0.32 μm, respectively, demonstrating high-fidelity photolithographic outcomes.

In addition, although the photosensitivity of PRL is relatively weak in the commercial i-line region (365 nm), it remains capable of direct photo-patterning (Supplementary Fig. 19), which further enhances the practical applicability of our strategy. On the other hand, the compatibility of reversible photolithography with flexible polyethylene terephthalate (PET) substrates and environmentally benign Cd-free InP QDs was experimentally validated (Fig. 5d, e).

Multicolor QD patterns were successfully developed by combining RGB QDs (Fig. 5f). In this process, patterned RGB QD films demonstrate nearly identical PL spectra to their pristine counterparts, extending the high-fidelity feature to full-color systems. Supplementary Fig. 20 reports CIE coordinates of crosslinked films at (0.69, 0.31) for RQD, (0.26, 0.72) for GQD, and (0.10, 0.14) for BQD, covering 98.2% of the widely used NTSC (National Television System Committee) color gamut. Encouragingly, the reversible photolithography strategy remains fully compatible with multicolor QD integration. As shown in Fig. 5g, multicolor QD patterns—for example, overlays of RGB pixels—are prepared through in situ photo-induced crosslinking and developing. During this process, no mutual erosion occurs, because the pre-patterned QDs remain in the crosslinked state. Subsequently, a DUV-induced decrosslinking step restores the pristine ligand states of all QD pixels. As a result, we successfully fabricated RGB QD pixel arrays on the same substrate (Fig. 5g). Furthermore, the vertical stacking of RGB pixels generated three secondary colors—cyan, orange, and pink—while maintaining well-defined pixel shapes and clear boundaries. These results lay a solid foundation for future full-color display applications. Moreover, the reversible photolithography strategy also enables reconfiguration of QD patterns. As a proof of concept, we first fabricated 20 × 50 µm QD pixels via photo-crosslinking (Supplementary Fig. 21). The photomask was then repositioned to allow spatially localized decrosslinking, yielding trimmed QD pixels with reduced dimensions (e.g., 15 × 20 µm).

To further demonstrate the potential of reversible photolithography for ultrahigh-resolution EL display applications, we fabricated QLEDs with QD pixel arrays spanning resolutions from 3300 PPI (pixel width: ~4.5 μm) to 21,000 PPI (pixel width: ~0.8 μm), as illustrated in Fig. 5h. AFM measurements further confirm that the QD pixels exhibit well-defined boundaries without mutual adhesion, even at ultrahigh resolution (Supplementary Fig. 22). We attribute the high resolution to two main factors: (i) the thiol-anchoring group of PRL binds strongly to the QD surface (demonstrated in Fig. 3d), resulting in minimal chemical diffusion; (ii) the photo-crosslinking reaction is highly site-selective and efficient (with short exposure time and low dose). These prototypes are expected to be applied to versatile applications. Specifically, a pixel density of ~3300 PPI delivers sufficient image quality at near-eye distances (e.g., 1 inch), meeting AR/VR display requirements^60^, while ~10,500 PPI aligns with the metaverse’s escalating demand for ultrahigh-definition interfaces (>10^4^ PPI). The ~21,000 PPI resolution, representing one of the highest resolution metrics reported for direct QD photolithography and exceeding human visual acuity by an order of magnitude, offers significant potential for nanophotonics and AI inference, particularly as an ultrahigh-resolution display array integrating spatial light modulators with photodetector arrays to enable faster, energy-efficient analog optical computing^61^.

EL spectra of QLEDs based on pristine, crosslinked, and pixelated PRL-GQD remained identical (Supplementary Fig. 23), confirming preservation of QD structural and optical integrity during the photolithographic patterning process. Notably, pixelated QLEDs suffer from pronounced leakage currents due to direct HTL/ETL contact in inter-pixel gaps. Consequently, research in this field has predominantly focused on non-pixelated devices^29^. To address this issue, multiple charge-blocking layers have been widely explored, such as polymethyl methacrylate (PMMA)^34,62–64^, polyvinyl alcohol^1^, nigrosin^65^, and perfluorodecyltriethoxysilane^66^.

Here, we introduced a spin-coated PMMA layer as the blocking layer in as-fabricated ultrahigh-resolution nano-pixelated QLEDs. Due to the fluidity of the liquid, the PMMA blocking layer is predominantly distributed in the inter-pixel gaps^67^. As shown in Supplementary Fig. 24, the EL microscopy image reveals that the introduction of PMMA does not affect the geometry of QD pixels. The pixel array maintains a pixel width of 0.8 µm, a pixel gap of 0.41 µm, and a pixel pitch of 1.21 µm, corresponding to a pixel density of ~21,000 PPI.

EL measurements further reveal that the device after decrosslinking exhibits improved *L* (49,292 vs. 28,769 cd m^−2^), *J* (65.6 vs. 40.7 mA cm^−2^), and peak EQE (17.08% vs. 16.02%), as shown in Fig. 5i and Supplementary Fig. 24, which is consistent with the performance trends observed in non-patterned devices. Note that the peak EQE of the nano-pixelated devices is slightly lower than that of the non-patterned ones. Potential contributing factors include the non-ideal pixel morphology at such a high resolution, the electric-field non-uniformity arising from pixel microstructures^1^, and the residual PMMA-induced difficulty in charge injection^67^. Encouragingly, this value of 17.08% at 21,000 PPI remains highly competitive (Supplementary Fig. 25 and Table 2). These results further underscore the effectiveness of our reversible photolithography strategy in simultaneously achieving high resolution and high device performance.

## Discussion

In summary, we have developed a reversible photolithography strategy of QDs based on a rationally designed photo-reversible ligand (PRL), enabling wavelength-gated, reversible control over the photolithographic process. Combined with effective ligand passivation, this strategy overcomes the inherent performance degradation of direct photolithographic QDs. Consequently, the resulting QLED devices deliver high-performance electroluminescence, demonstrating one of the highest luminance values (125,016 cd m^−2^ at 5.0 V) and peak EQE (24.41%). Moreover, an ultrahigh-resolution nano-pixelated QLED with a peak EQE of 17.08% at 21,000 PPI is successfully demonstrated. Collectively, these results establish a reversible photolithographic framework for nondestructive QD integration, providing a transformative pathway for micro-/nanofabrication in QD-based electronic and optoelectronic platforms.

## Methods

### Reversible photolithography of QDs

The reversible photolithography is illustrated with PRL-GQD (Supplementary Fig. 3):

(i) The photo-induced crosslinking process: at first, the solution of PRL-GQD was coated on a substrate (e.g., cleaned silicon) at 2000 rpm to form a PRL-GQD film. Then the film was exposed to UV irradiation (centered at 310 nm) with photomasks for the required patterns. After UV exposure, the PRL-GQD film was immersed in toluene for 10 s to wash off the uncrosslinked areas. The crosslinked PRL-GQD film or pattern was obtained successfully.

(ii) The photo-induced decrosslinking process: the crosslinked PRL-GQD film or pattern was exposed to DUV irradiation (centered at 254 nm). In this process, the crosslinked PRL-GQD are restored to their initial dispersed state.

Note: the reversible photolithography process can be conducted either under ambient conditions or in a nitrogen-filled glove box. Unless otherwise stated, all experiments were performed in a nitrogen-filled glove box by default, with a UV dose of ~5 mJ cm^−2^.

### Nanoparticle size distribution

The mean centre-to-centre distance (*d*) for neighboring QDs is calculated from the peak position in the GISAXS scattering profile using the equation:

2 $$d=2\pi {q}^{-1}$$

According to the GISAXS data shown in Supplementary Fig. 12, the *d* values of PRL-GQD, crosslinked PRL-GQD, and decrosslinked PRL-GQD are 8.42, 8.70, and 8.55 nm.

### Reporting summary

Further information on research design is available in the Nature Portfolio Reporting Summary linked to this article.

## Supplementary information

Reporting Summary

Supplementary Information

Transparent Peer Review File

## Source data

Source Data

## Data Availability

The data that support the findings of this study are available from the corresponding author upon request. Source Data are provided with this paper.
