# Supplementary material for: Reversible photolithography of quantum dots achieves high-performance electroluminescence
Source: Nat Commun. 2026 Sep 24;17:11097. doi: 10.1038/s41467-026-77916-z (PMC13696730; doi:10.1038/s41467-026-77916-z)
Supplement: Supplementary file 2 [file 41467_2026_77916_MOESM2_ESM.pdf]

Supplementary Information

for

Reversible photolithography of quantum dots achieves high-performance electroluminescence

Chang Gu, Zhixin Zhai, Shuo Ding, Changfeng Han, Ting Zhang, Chaoyu Xiang

Supplementary Figures

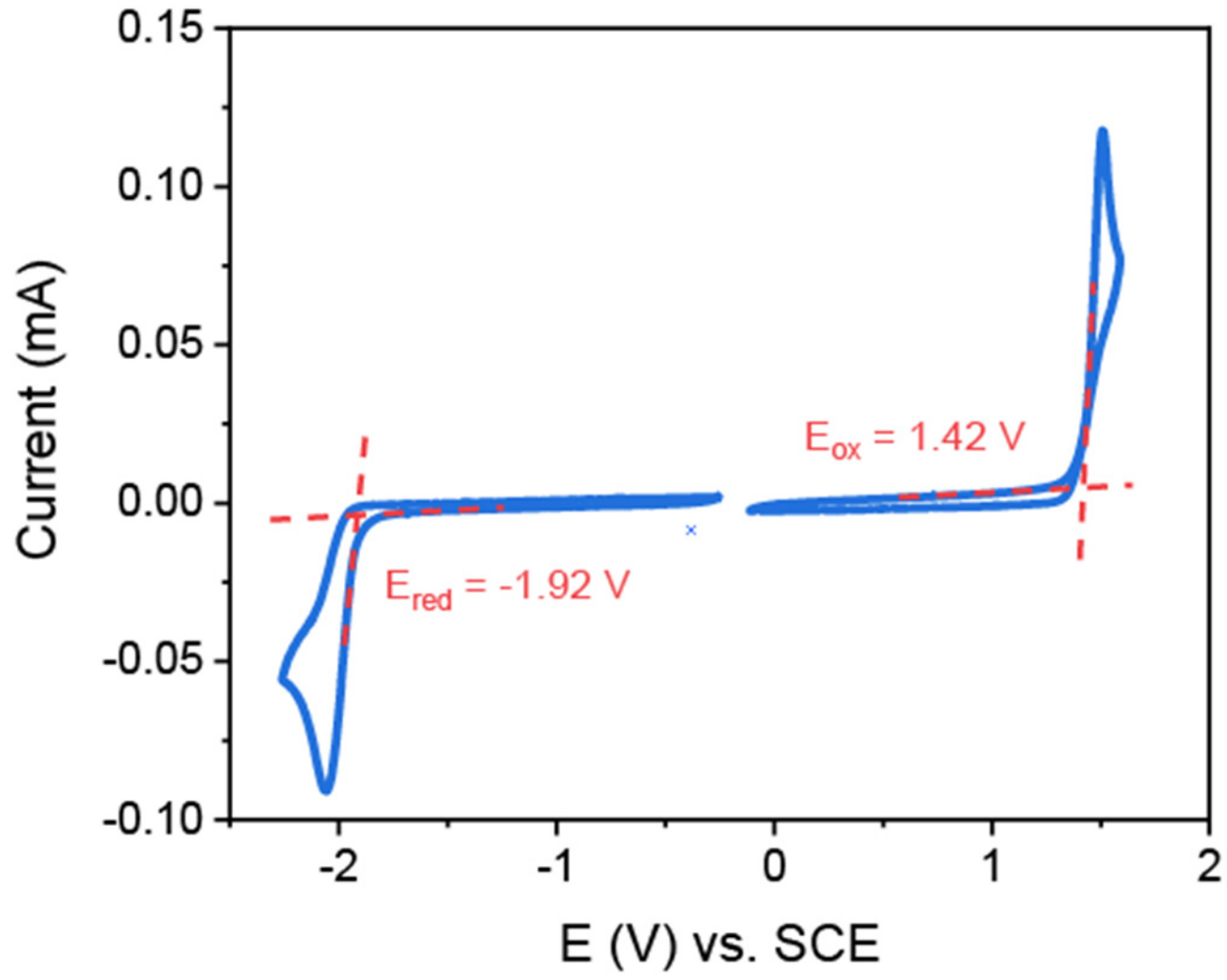

Supplementary Fig. 1 The cyclic voltammetry curves of PRL in acetonitrile. The scan rate is 100 mV s^{-1}.

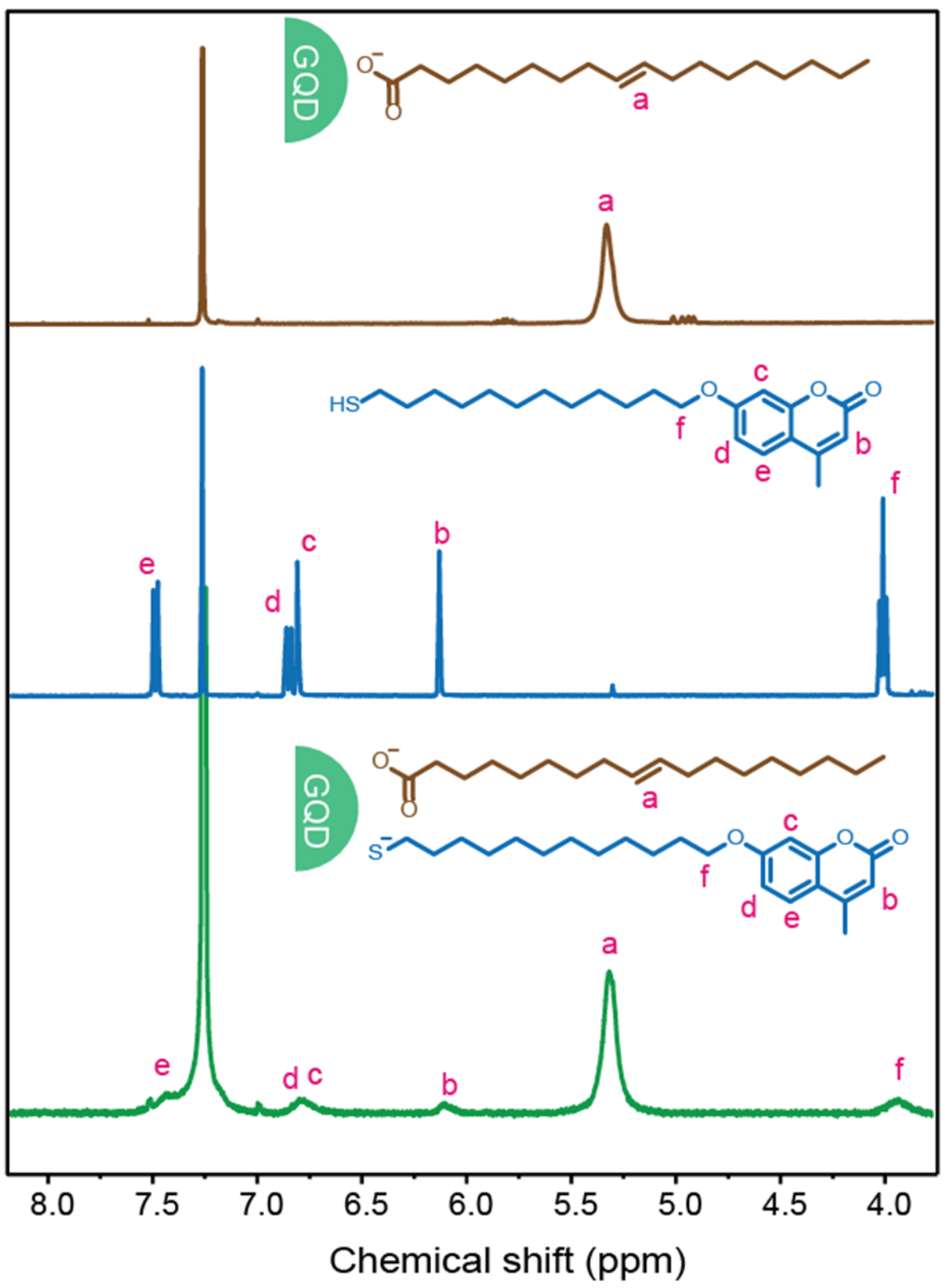

Supplementary Fig. 2 ^{1}H-NMR (400 MHz, Chloroform-*d*) of OA-GQD, PRL and PRL-GQD.

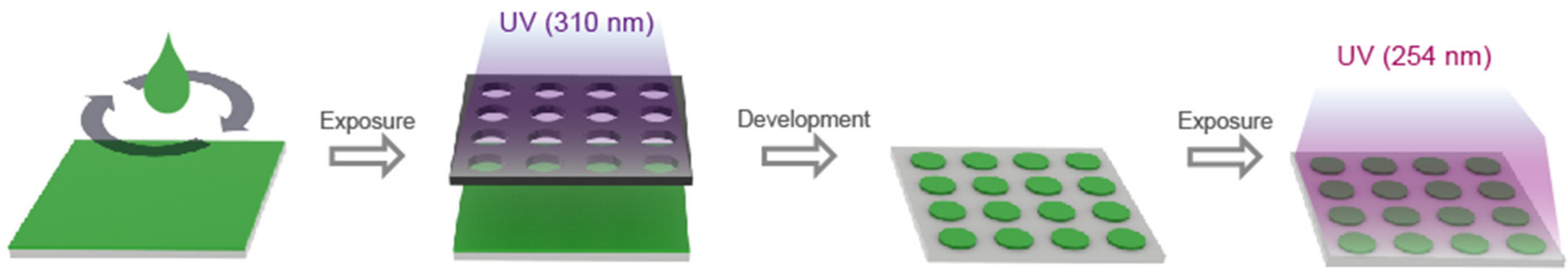

Supplementary Fig. 3 Schematic of reversible photolithography of QDs.

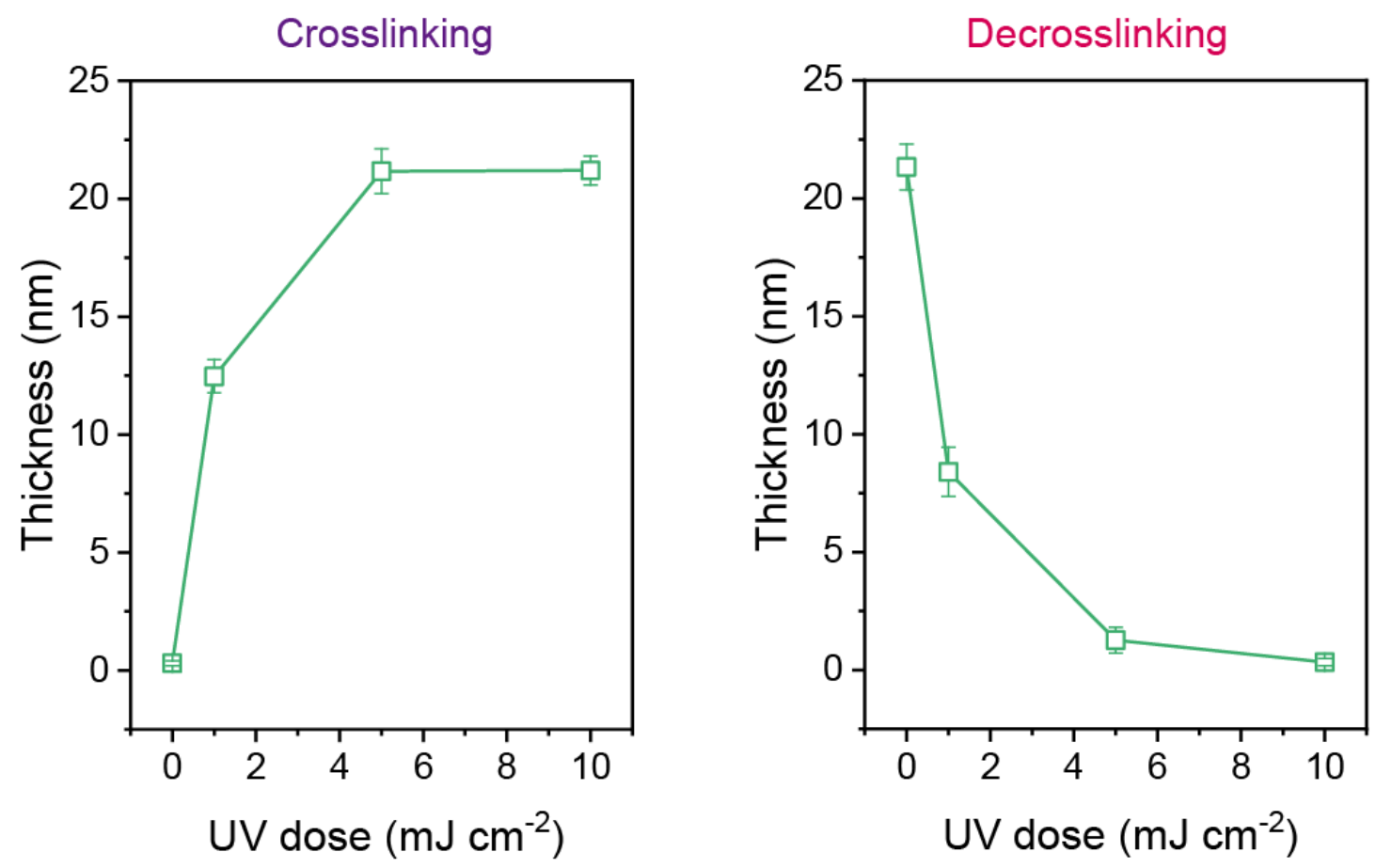

Supplementary Fig. 4 Film thickness of PRL-GQD films under the crosslinking process (left) and the decrosslinking process (right) with different exposure doses. UV lamps (310 nm, ~1 mW cm^{-2} for the crosslinking process; 254 nm, ~1 mW cm^{-2} for the decrosslinking process) and toluene development are used here. Three independent samples were measured for each parameter setting.

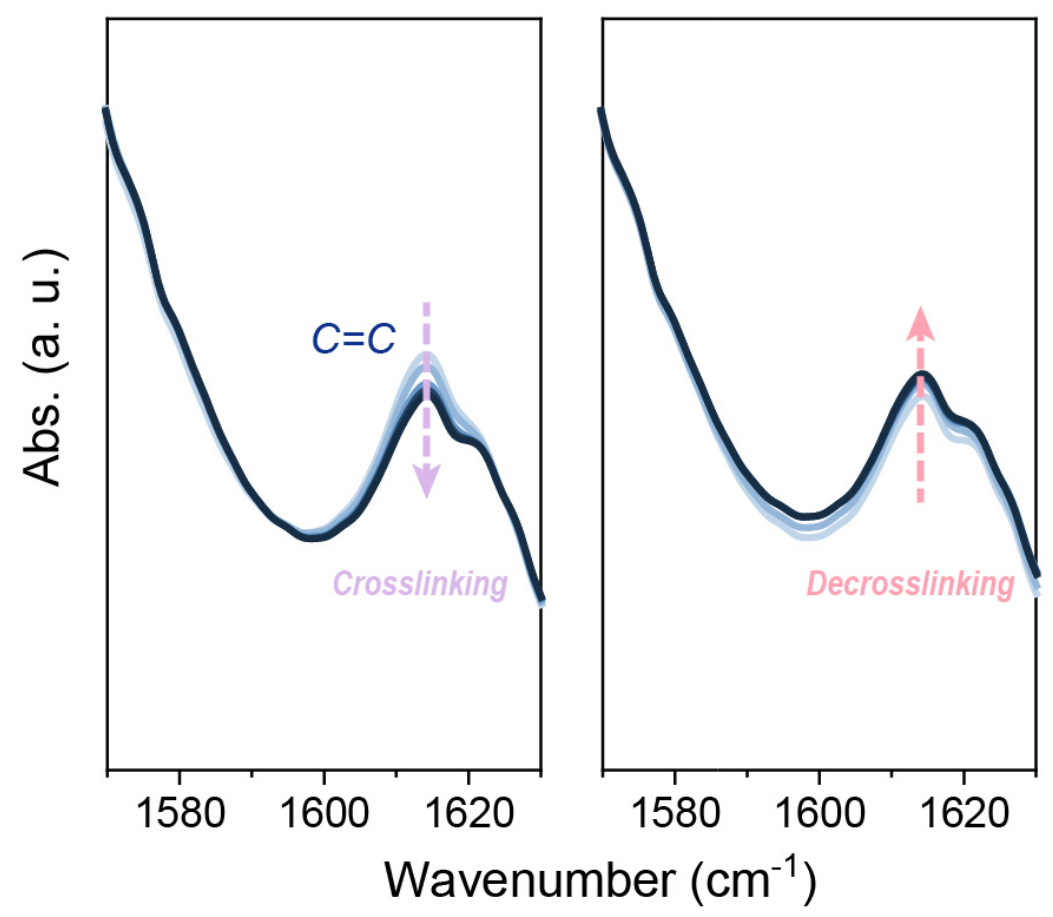

Supplementary Fig. 5 FT-IR spectra of PRL-GQD film during the photo-induced crosslinking process (left) and decrosslinking process (right).

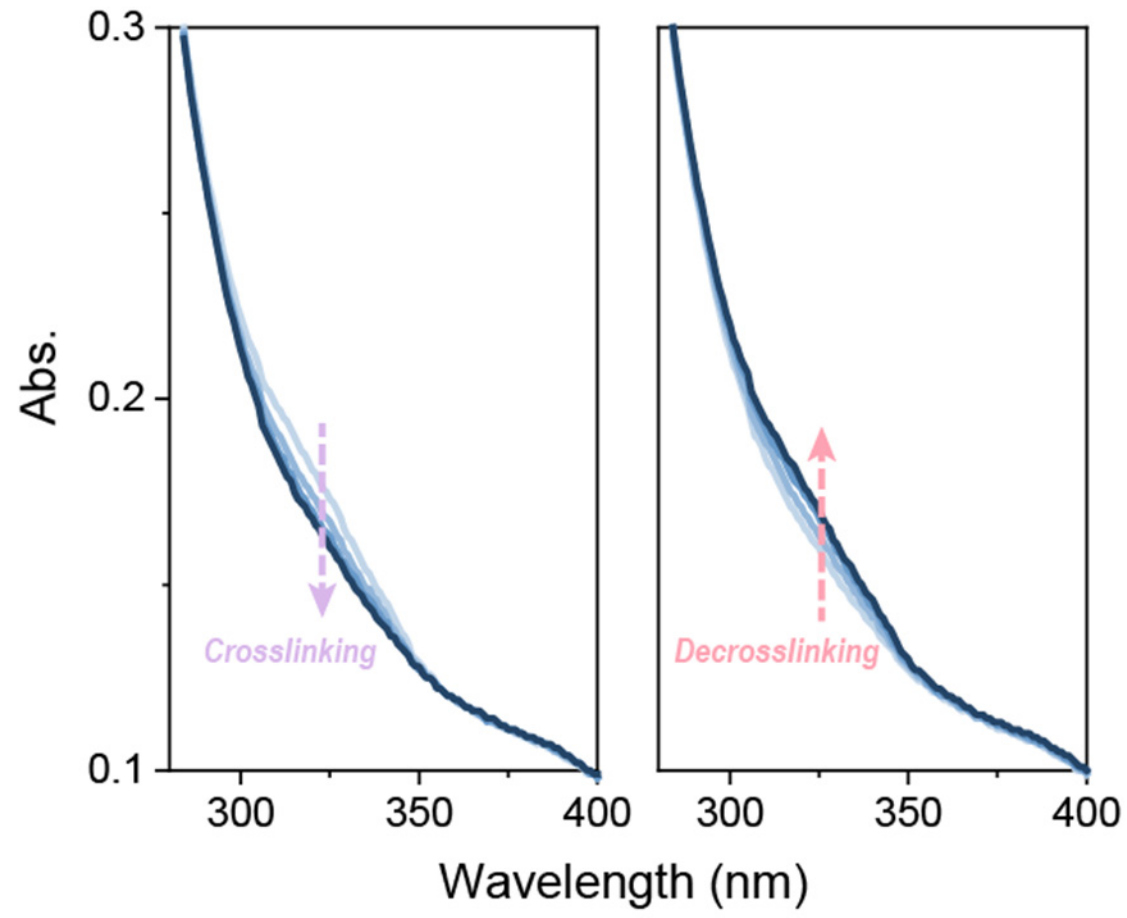

Supplementary Fig. 6 UV-Vis absorption spectra of PRL-GQD film during the photo-induced crosslinking process (left) and decrosslinking process (right).

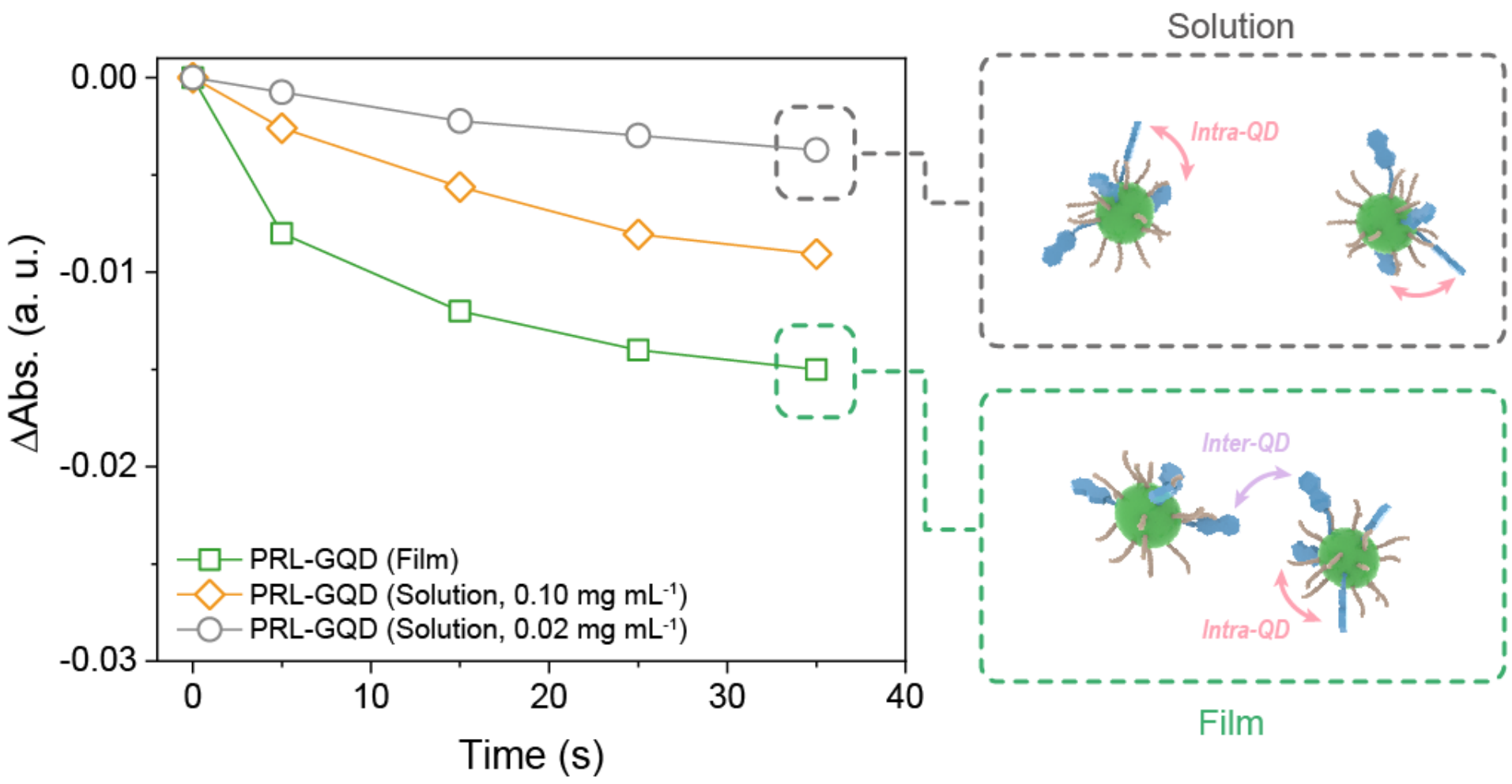

Supplementary Fig. 7 Left: normalized relative absorption changes at 318 nm of PRL-GQD film and PRL-GQD solution (0.10 mg mL^{-1} and 0.02 mg mL^{-1} in toluene) under UV exposure (centered at 310 nm, ~1 mW cm^{-2}). Right: potential intra-QD and inter-QD pathways in these systems.

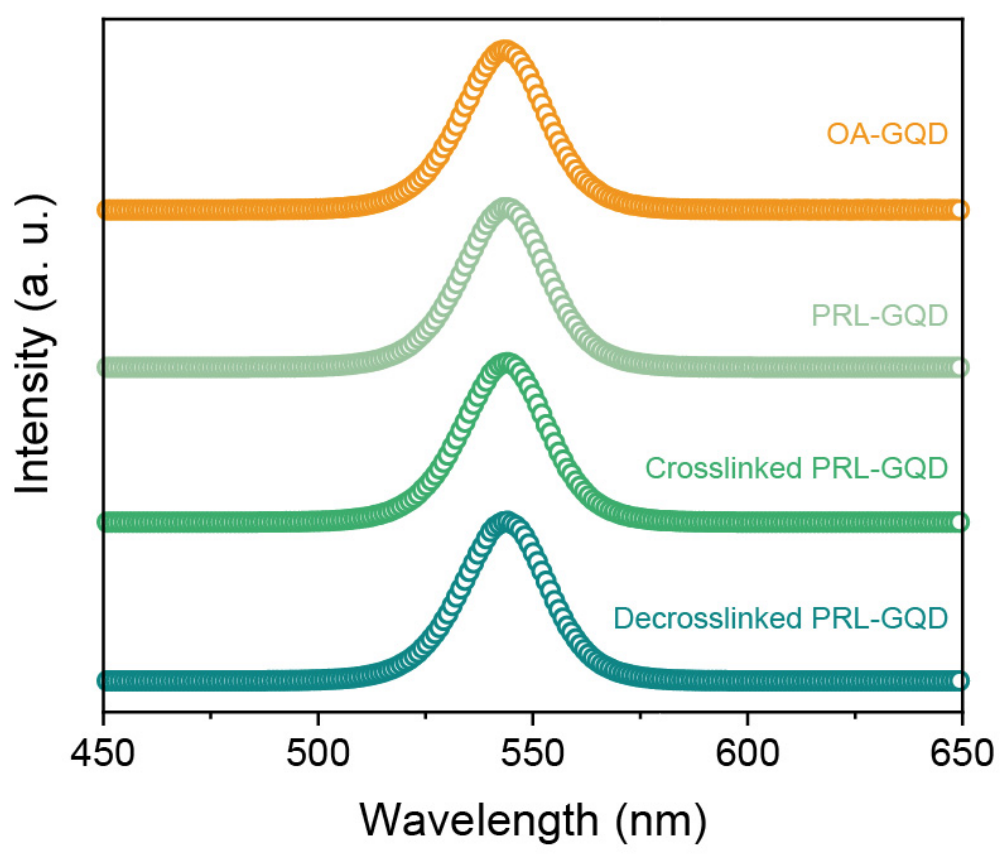

Supplementary Fig. 8 Normalized PL spectra of GQD films under reversible photolithography processes, including OA-GQD, PRL-GQD, crosslinked PRL-GQD and decrosslinked PRL-GQD.

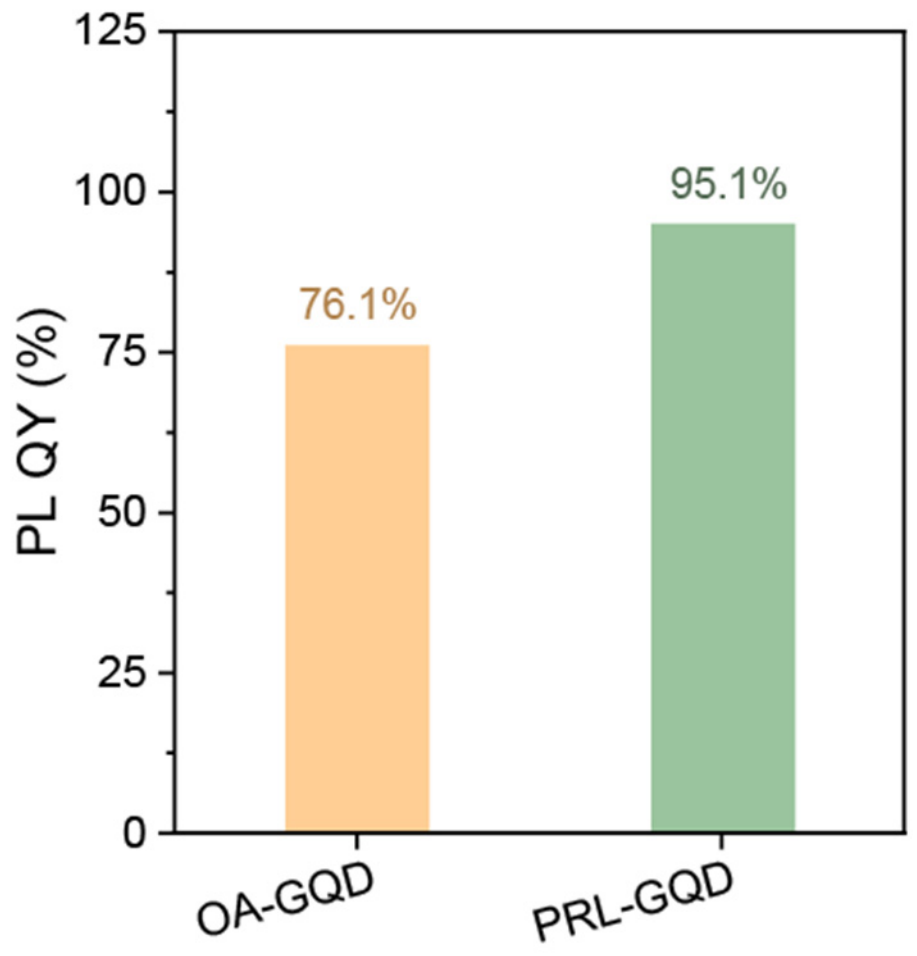

Supplementary Fig. 9 PL QYs of OA-GQD and PRL-GQD in toluene.

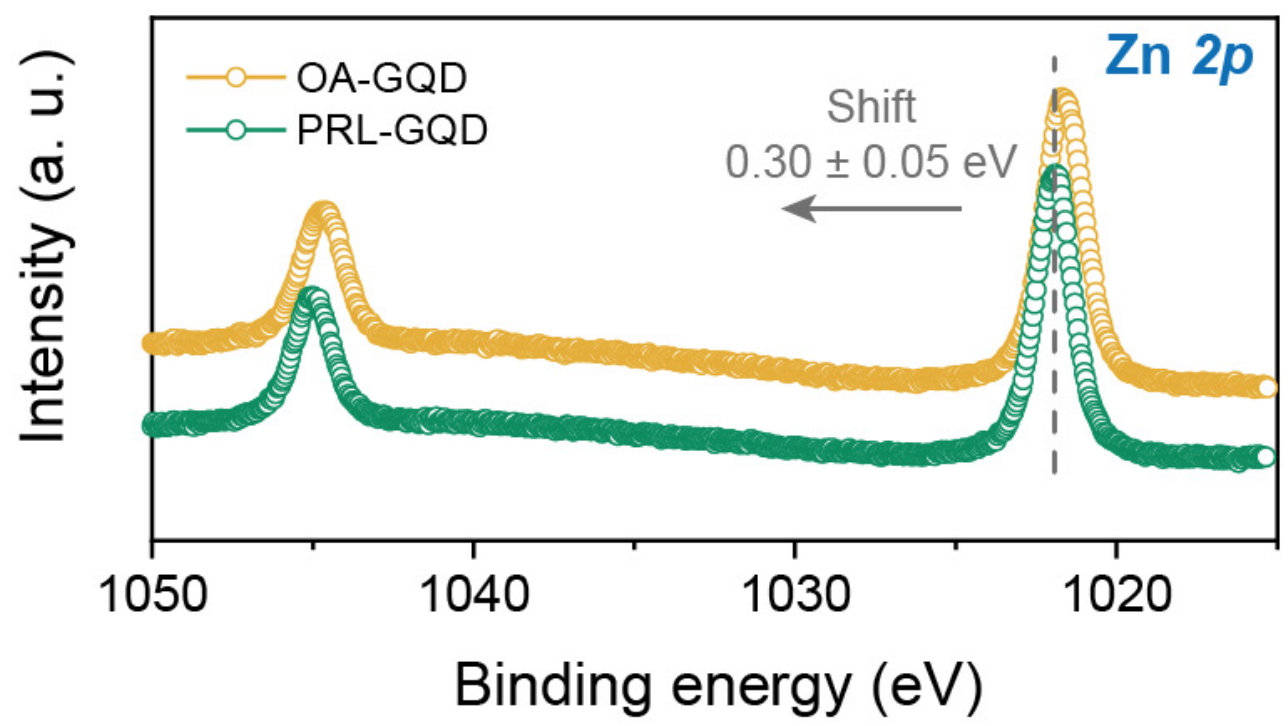

Supplementary Fig. 10 Zn *2p* spectra of OA-GQD and PRL-GQD.

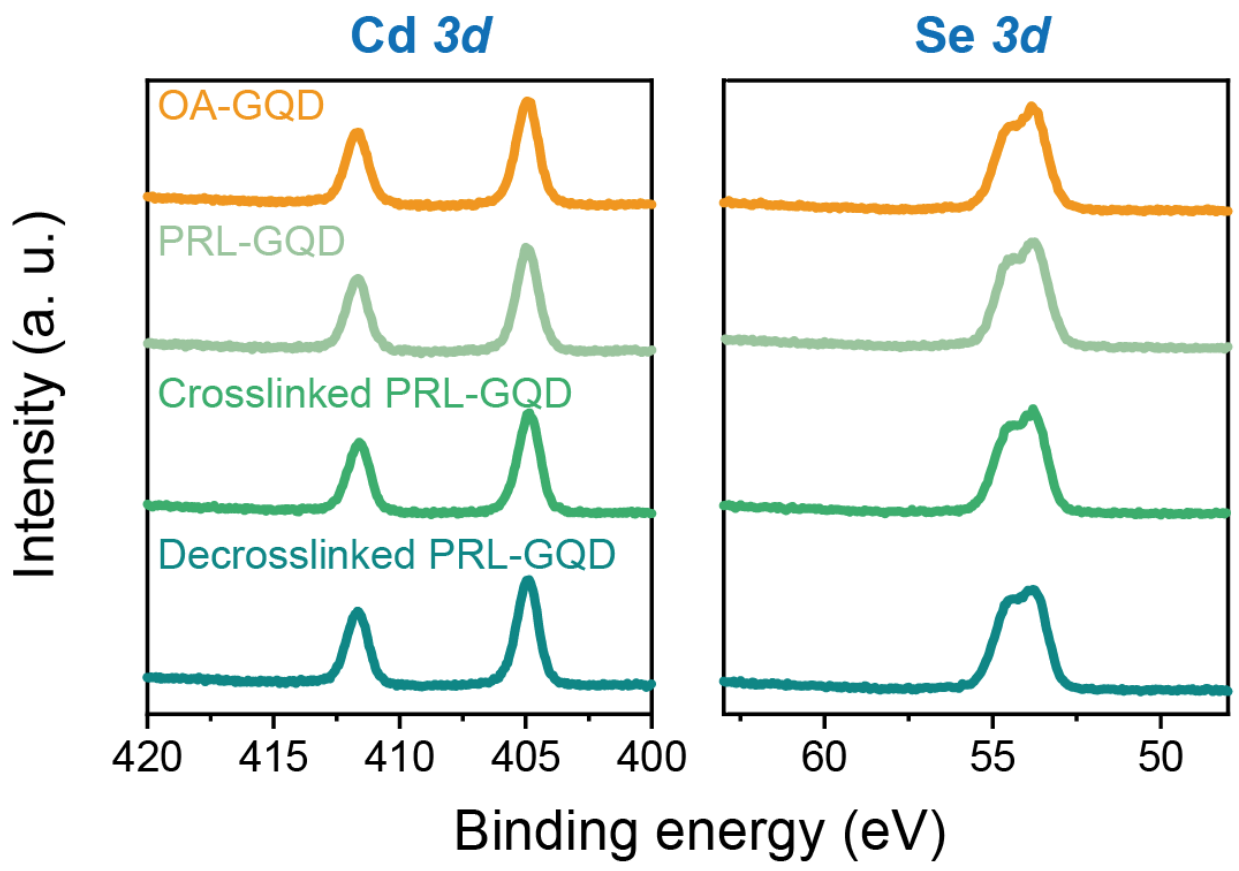

Supplementary Fig. 11 Cd *3d* (left) and Se *3d* (right) spectra of GQD films under different reversible photolithography processes.

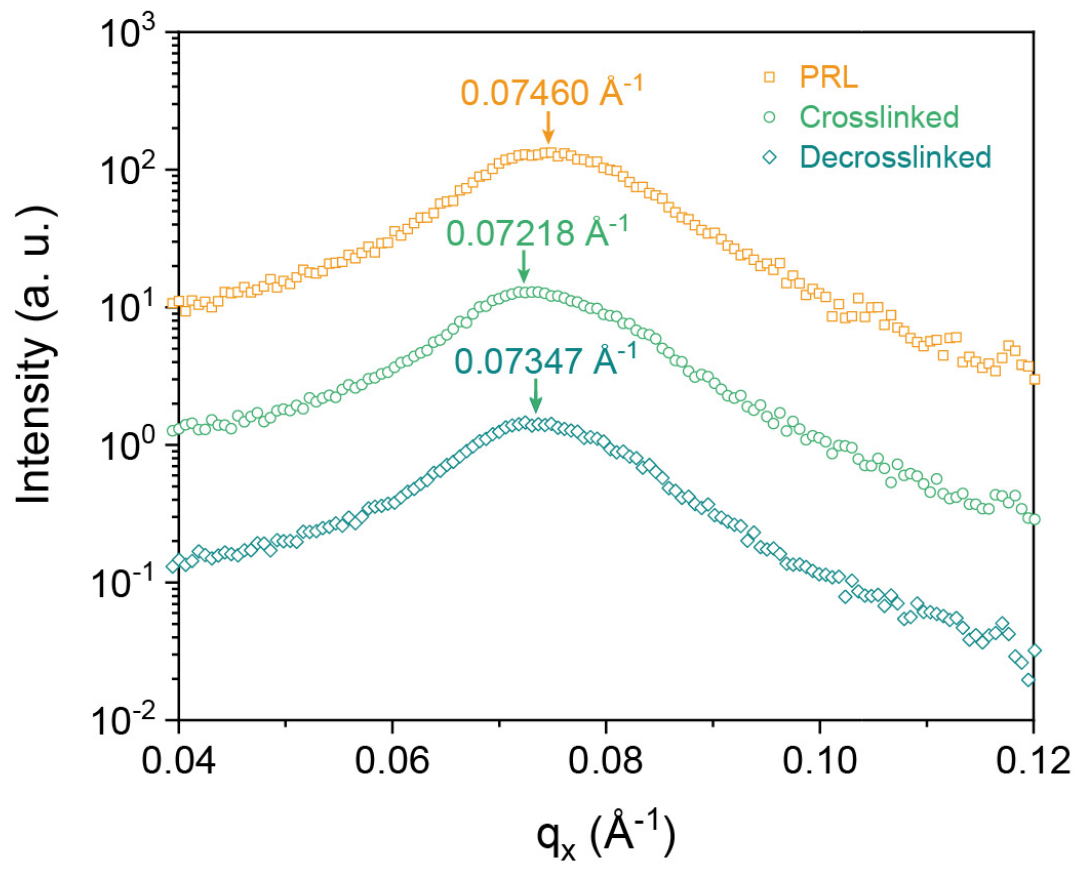

Supplementary Fig. 12 GISAXS spectra showing the interparticle distance of PRL-GQD (PRL), crosslinked PRL-GQD (Crosslinked) and decrosslinked PRL-GQD (Decrosslinked).

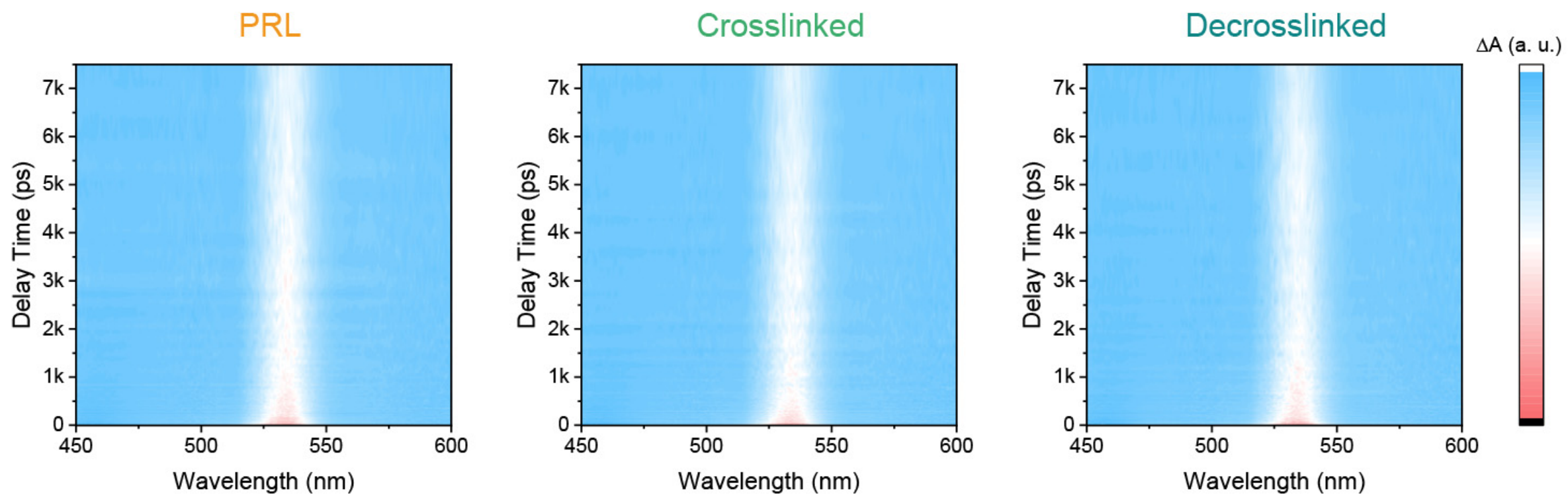

Supplementary Fig. 13 2D pseudo-color fs-TA plots of PRL-GQD (left), crosslinked PRL-GQD (middle) and decrosslinked PRL-GQD (right).

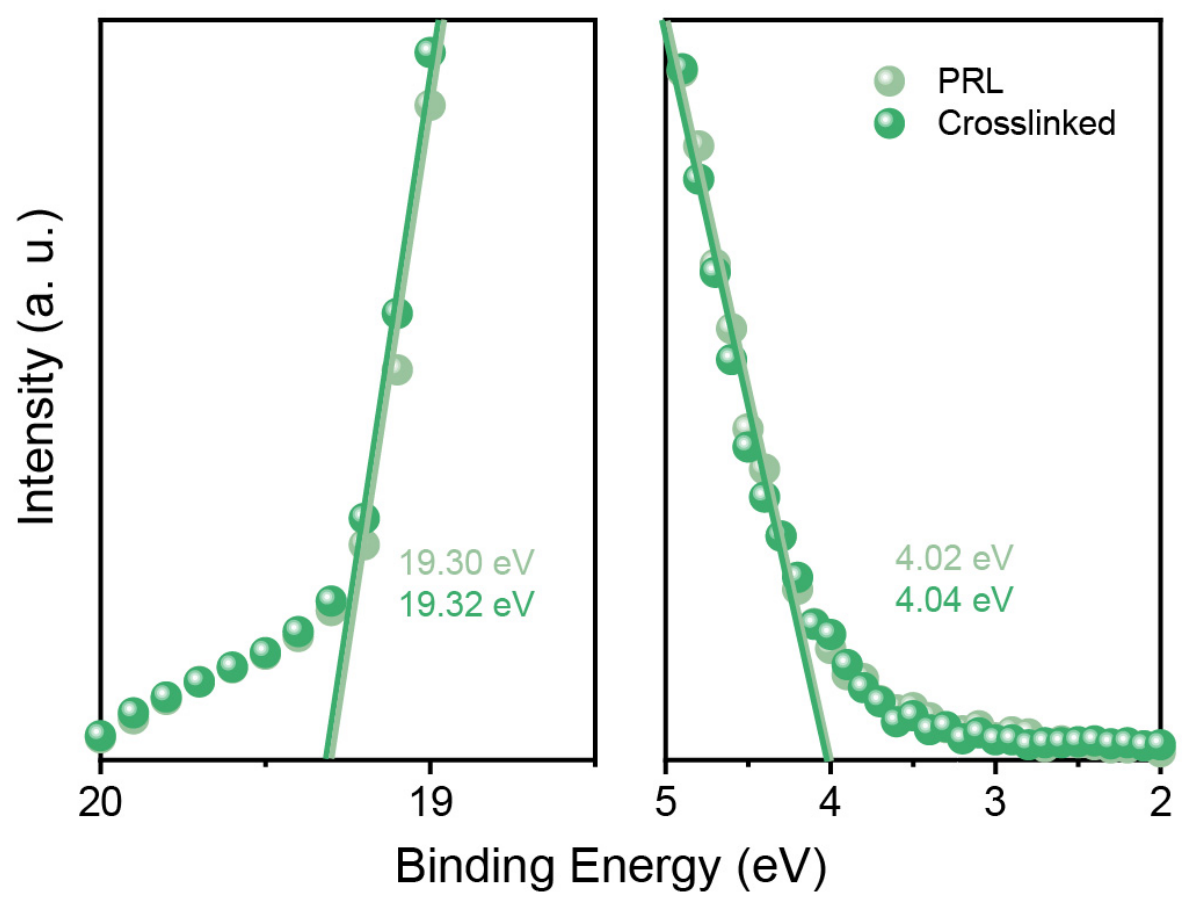

Supplementary Fig. 14 Ultraviolet photoelectron spectroscopy (UPS) results of PRL-GQD and crosslinked PRL-GQD.

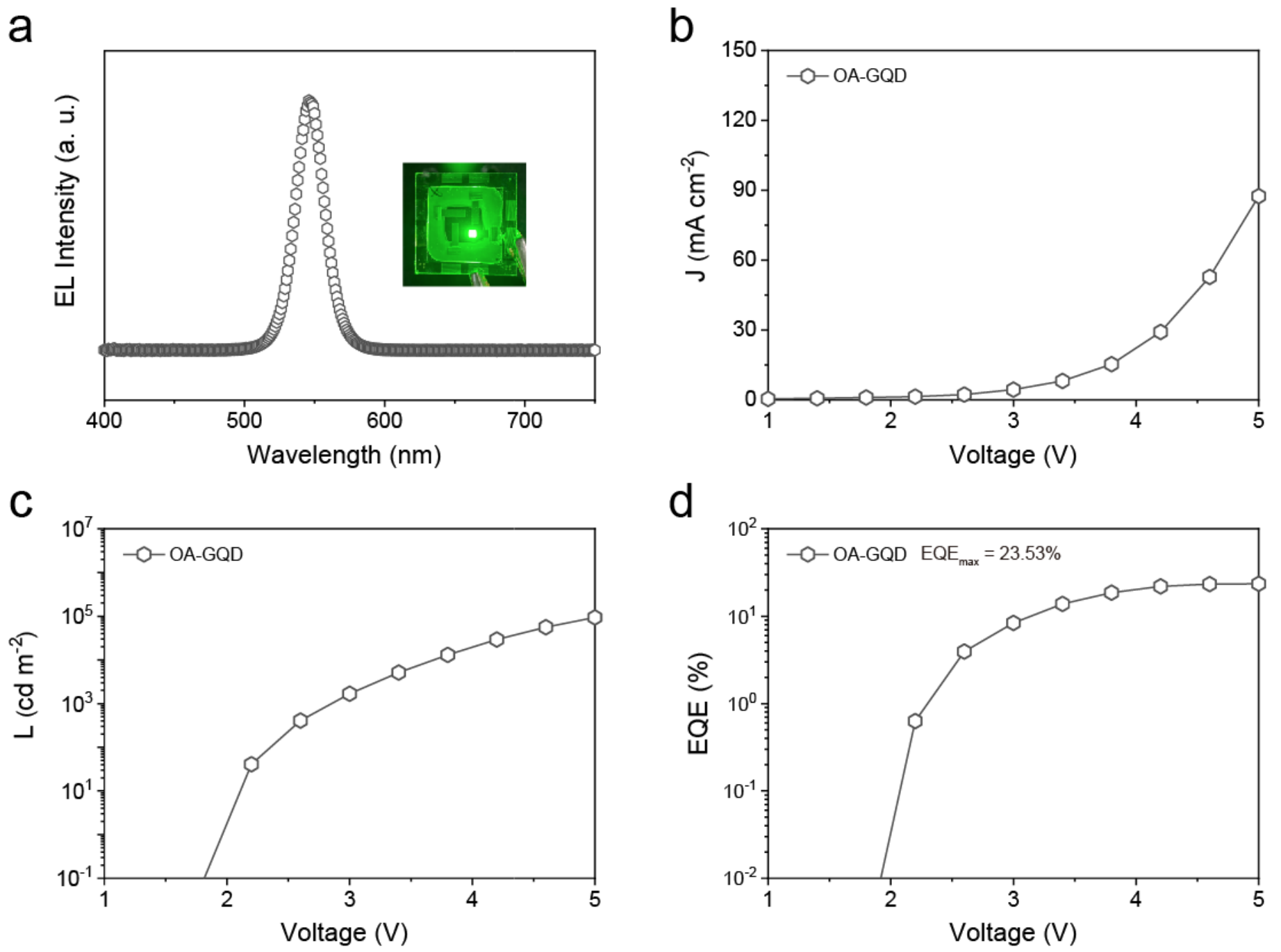

Supplementary Fig. 15 EL performance of the QLED device based on OA-GQD. a-d Normalized EL spectrum, *J*–*V* curve, *L*–*V* curve and EQE–*V* curve of the QLED. Inset in **a**: EL photo of corresponding QLED at 5.0 V.

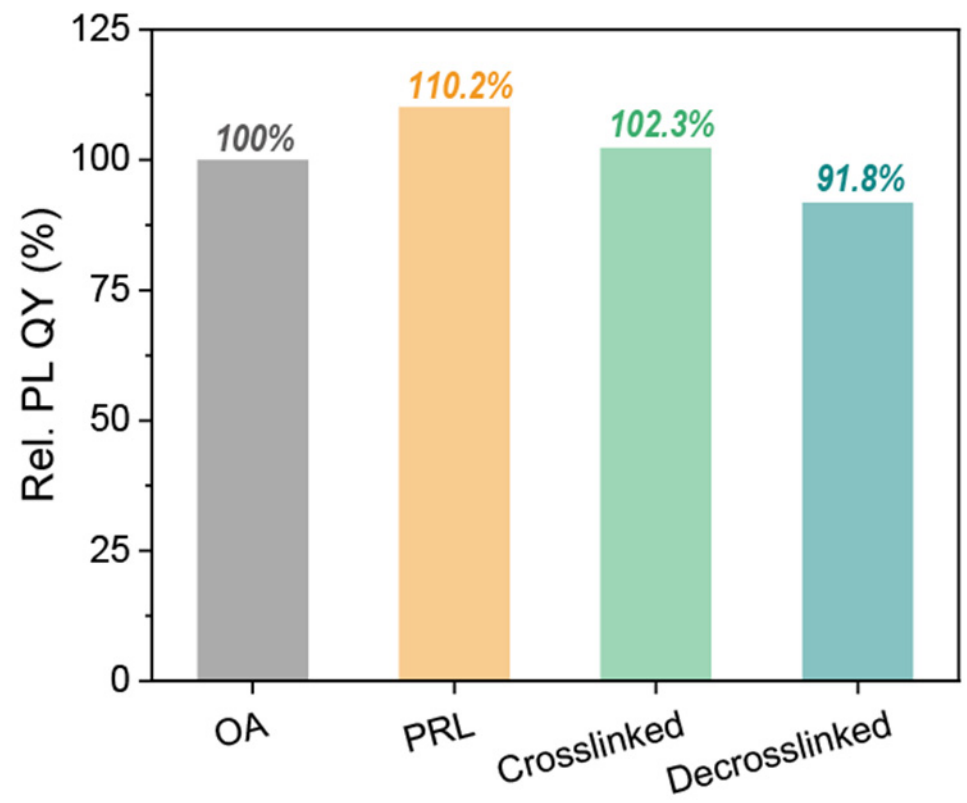

Supplementary Fig. 16 Relative PL QYs of OA-GQD, PRL-GQD, crosslinked PRL-GQD and decrosslinked PRL-GQD. The reversible photolithography process is carried out under ambient conditions.

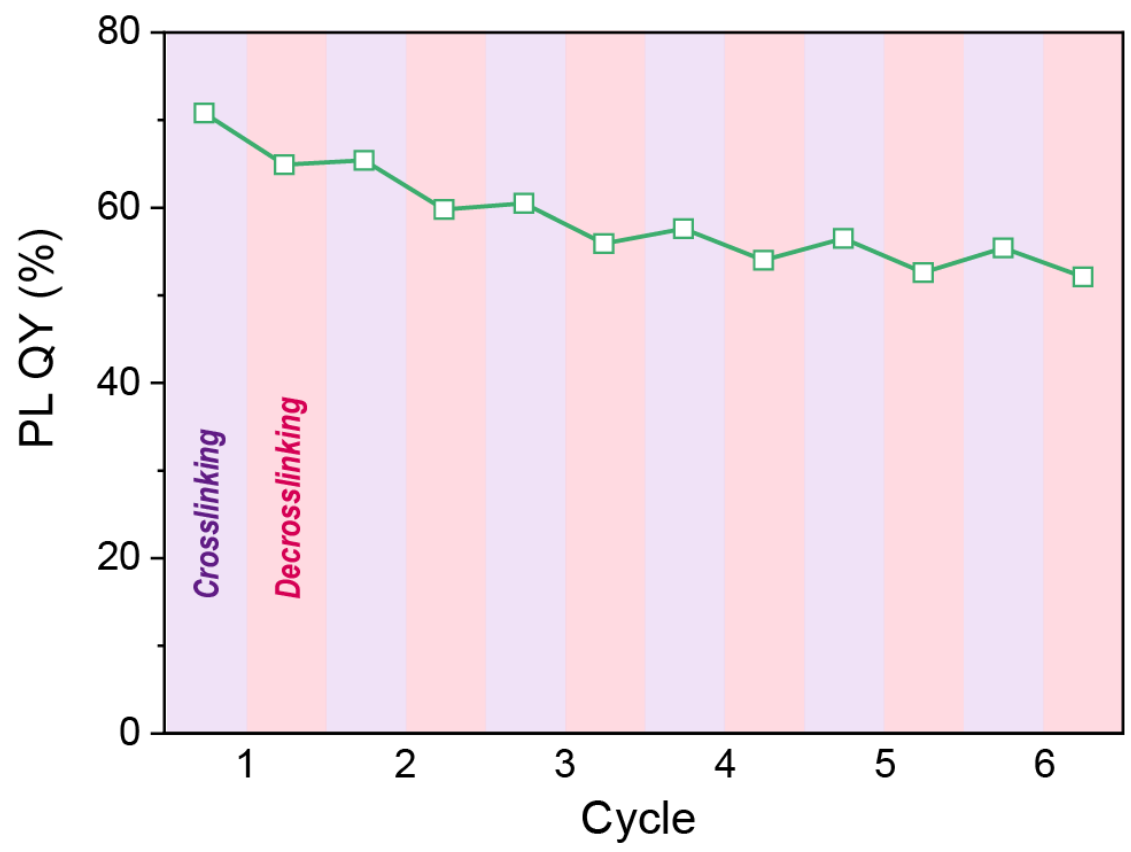

Supplementary Fig. 17 PL QYs evolution of the PRL-GQD film during cyclic crosslinking and decrosslinking processes. After each crosslinking step, the film was developed with toluene. All operations were carried out under ambient conditions.

Notably, despite a gradual decrease in overall PL QY, the crosslinked state consistently shows higher PL QY than the decrosslinked state, which may be attributed to the increased inter-QD spacing induced by crosslinking.

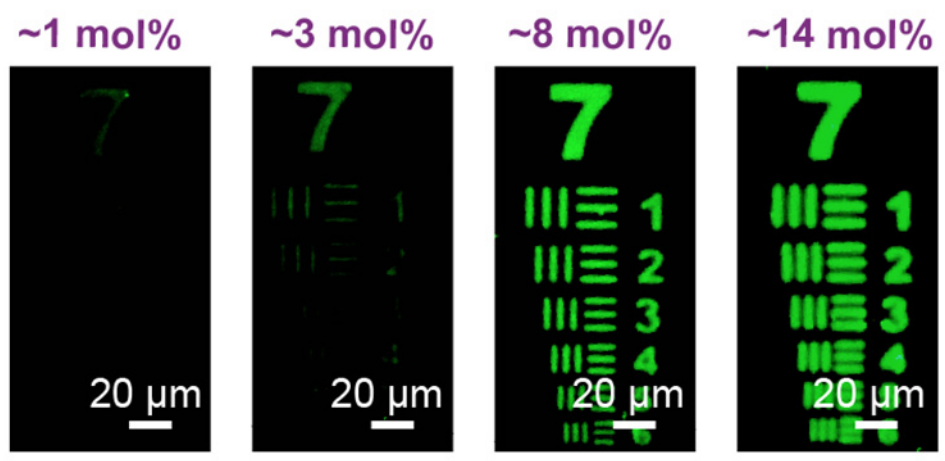

Supplementary Fig. 18 Fluorescence microscopic images of photolithographic QD patterns with different PRL contents.

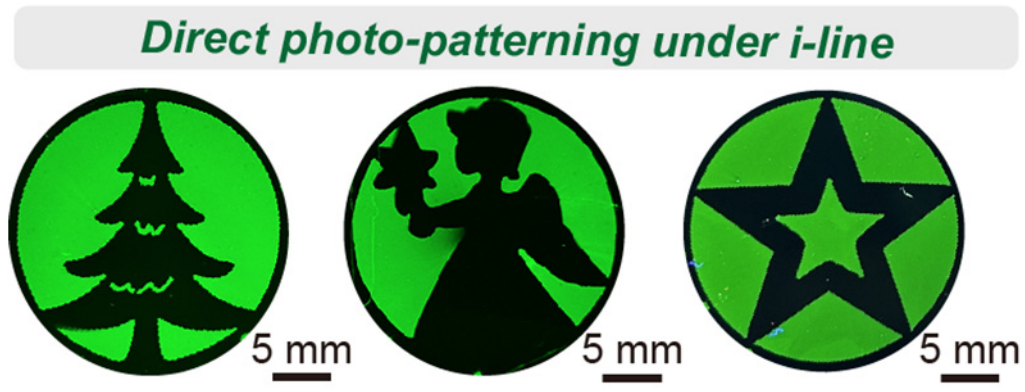

Supplementary Fig. 19 Fluorescent images of photolithographic PRL-GQD patterns under i-line. The required exposure dose is ~2 J cm^{-2}.

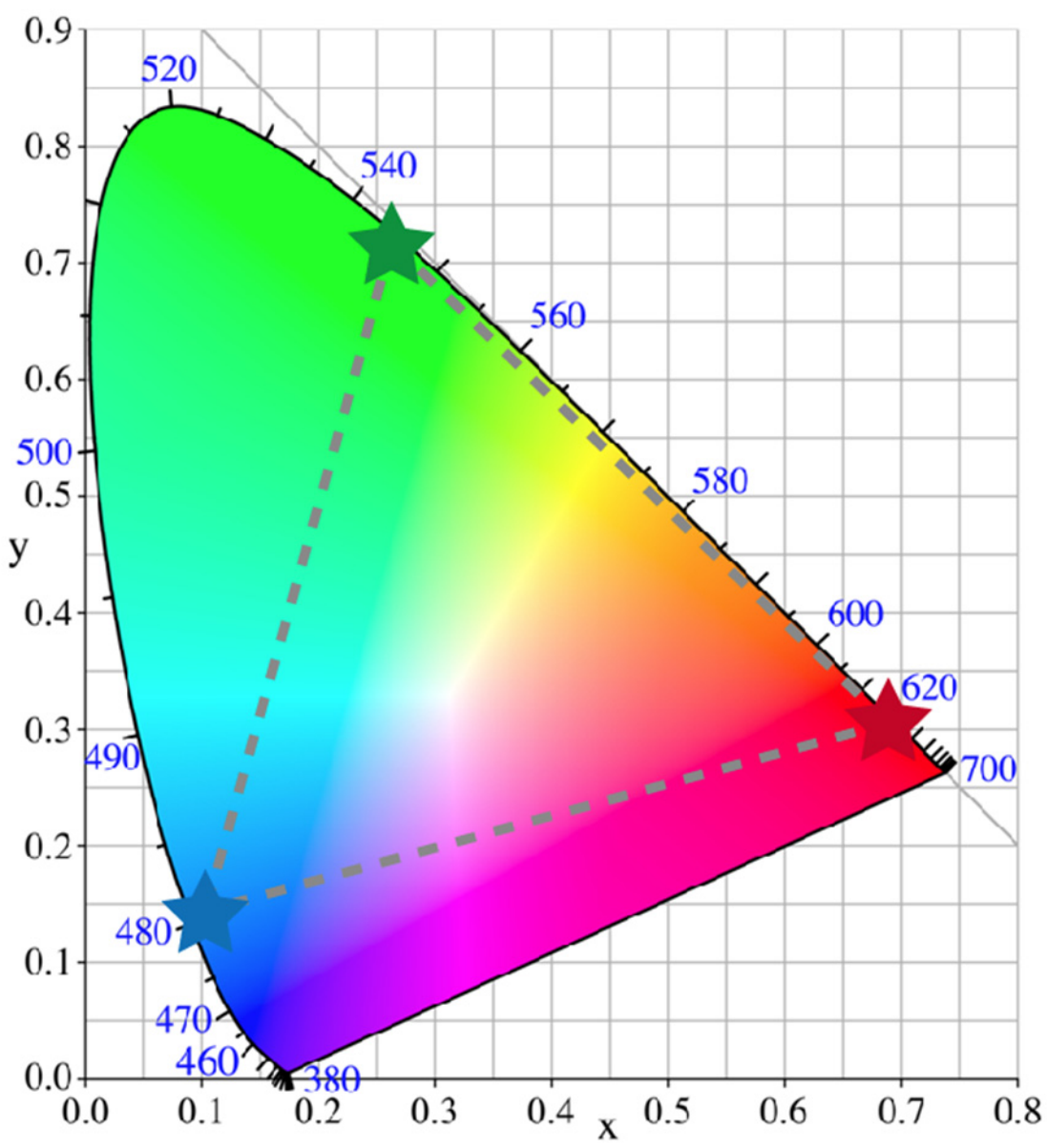

Supplementary Fig. 20 CIE coordinates of patterned RGB QDs.

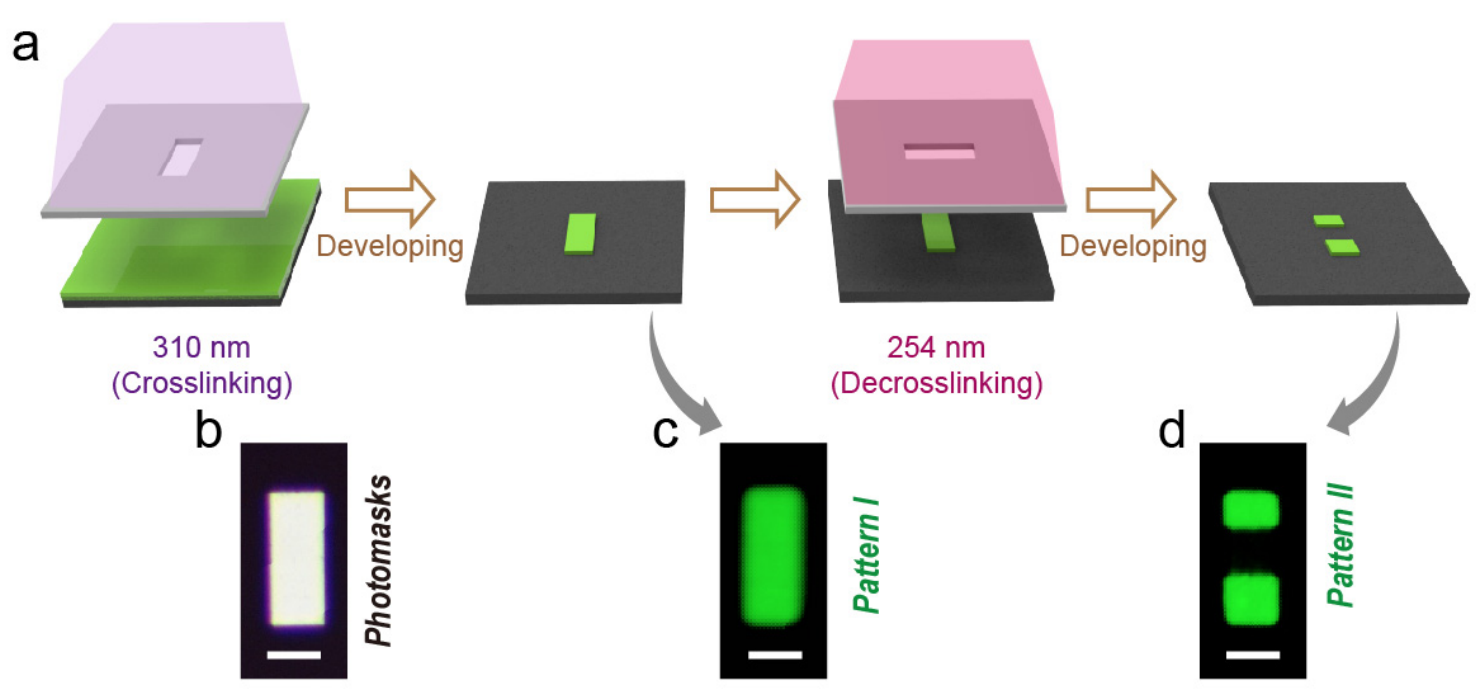

Supplementary Fig. 21 a Schematic of reconfigurable photo-patterning based on reversible photolithography strategy. **b-d** Microscopic image of the photomask and corresponding fluorescent microscopic images of QDs before and after spatially localized decrosslinking. Scale bar: 20 μm.

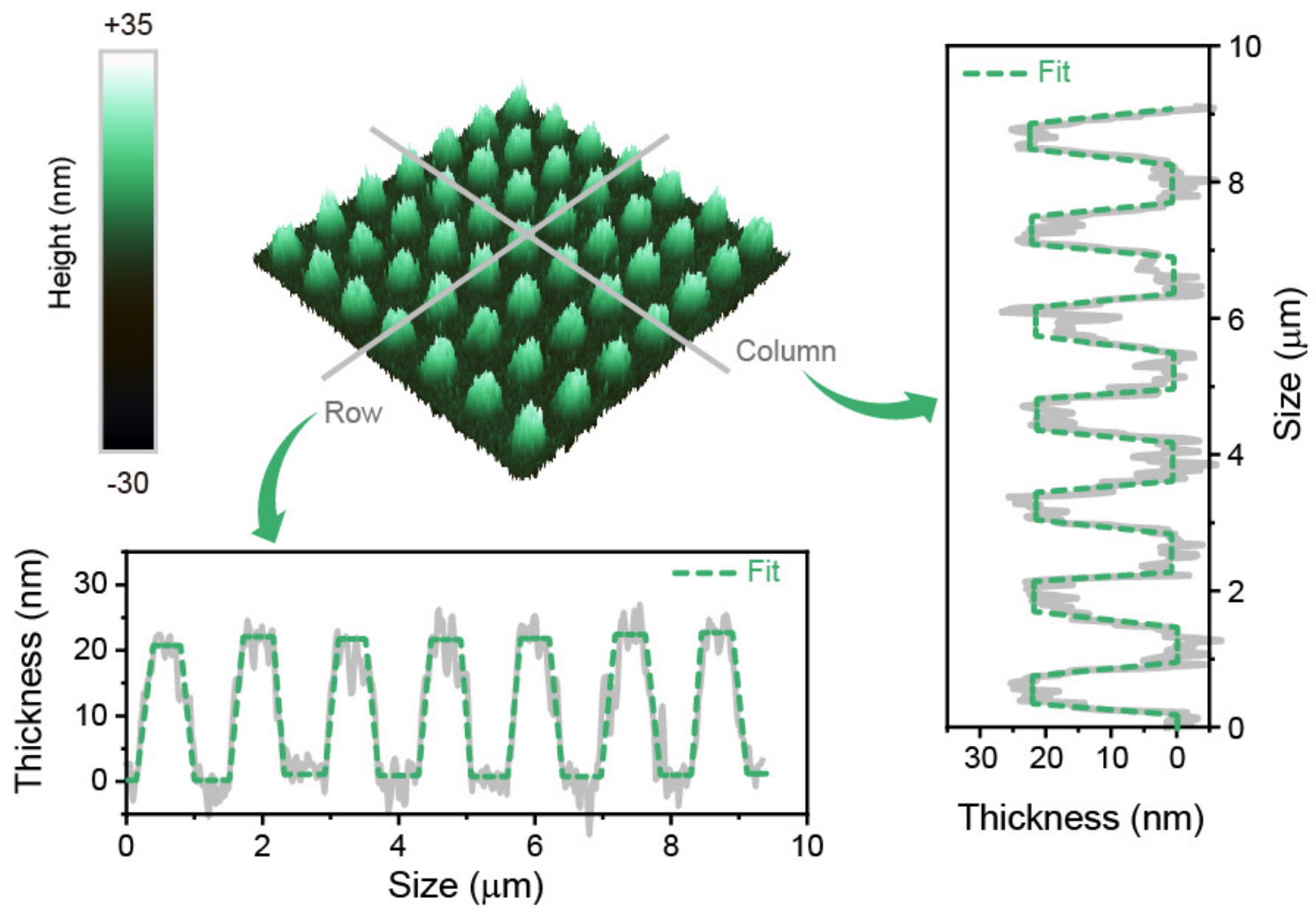

Supplementary Fig. 22 Three-dimensional AFM image and surface profile of QD pixels.

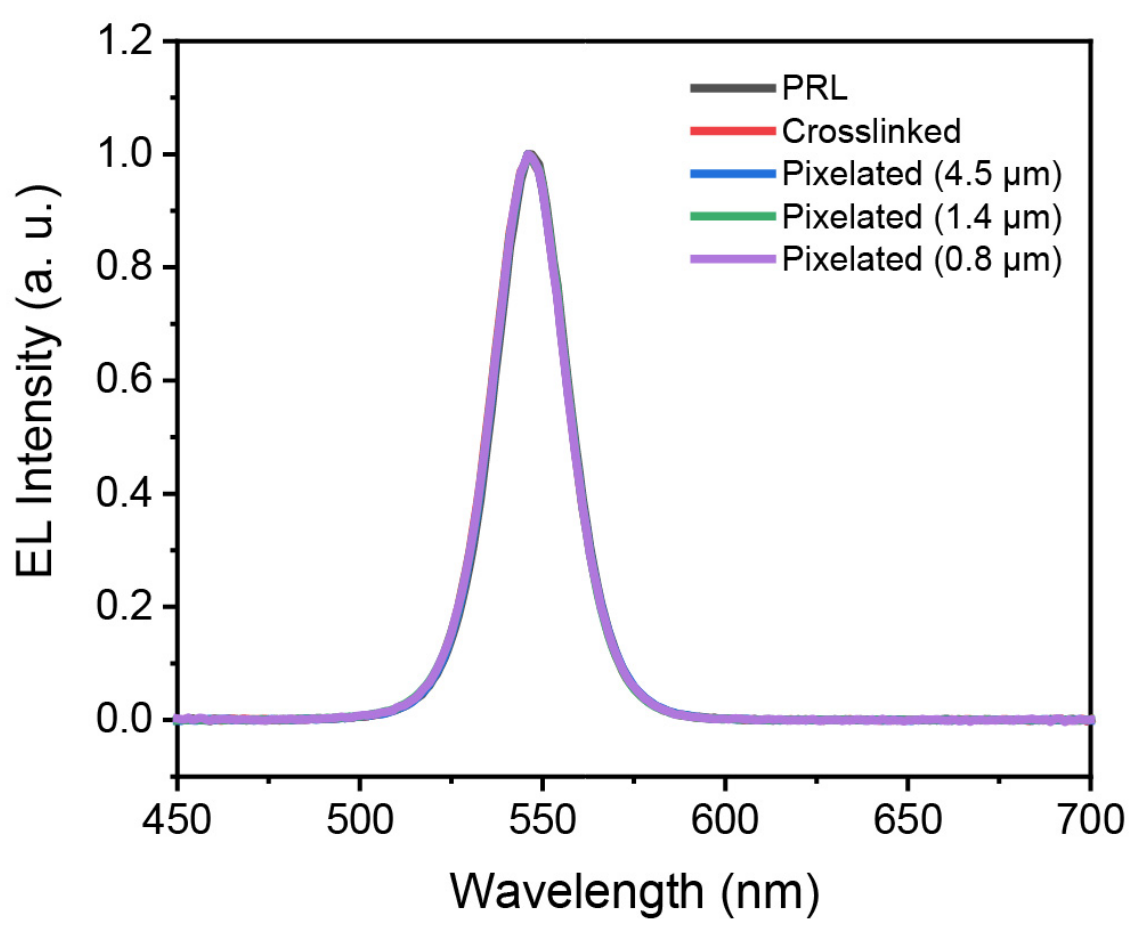

Supplementary Fig. 23 EL spectra of QLEDs based on PRL-GQD, crosslinked PRL-GQD and pixelated PRL-GQD with different pixel sizes.

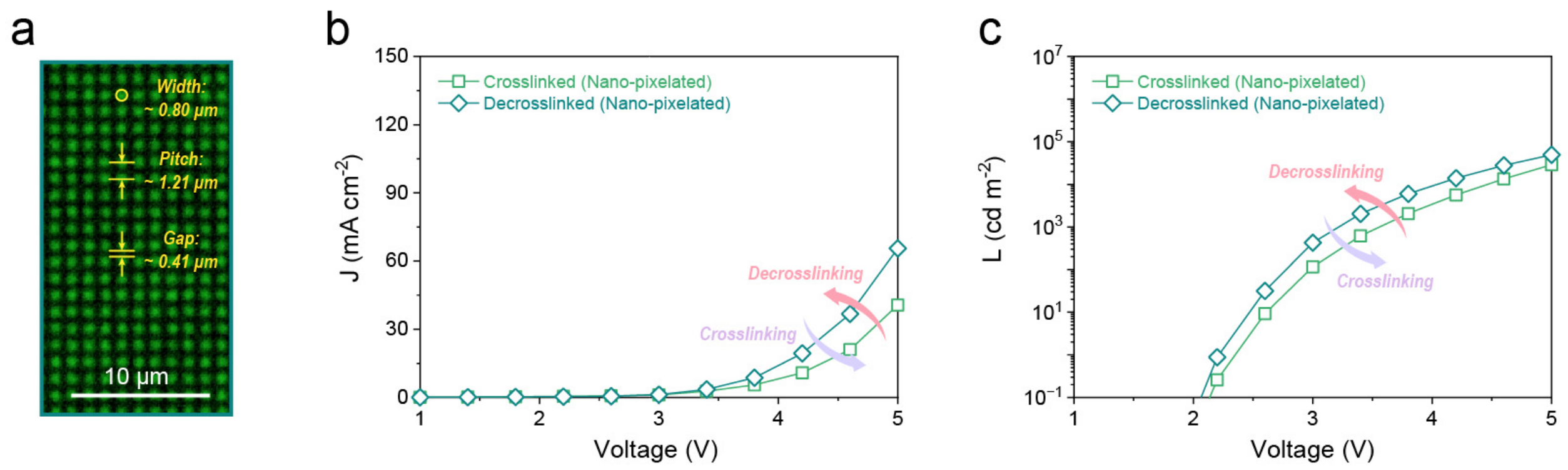

Supplementary Fig. 24 a EL microscopic image of nano-pixelated QLED based on decrosslinked PRL-GQD. **b-c** *J*–*V* curves and *L*–*V* curves of nano-pixelated QLEDs based on crosslinked PRL-GQD and decrosslinked PRL-GQD. Note that the *J* and *L* in **b-c** are instrument-derived, statistically averaged values based on the total electrode area, and these values should be corrected by the pixel fill factor (FF).

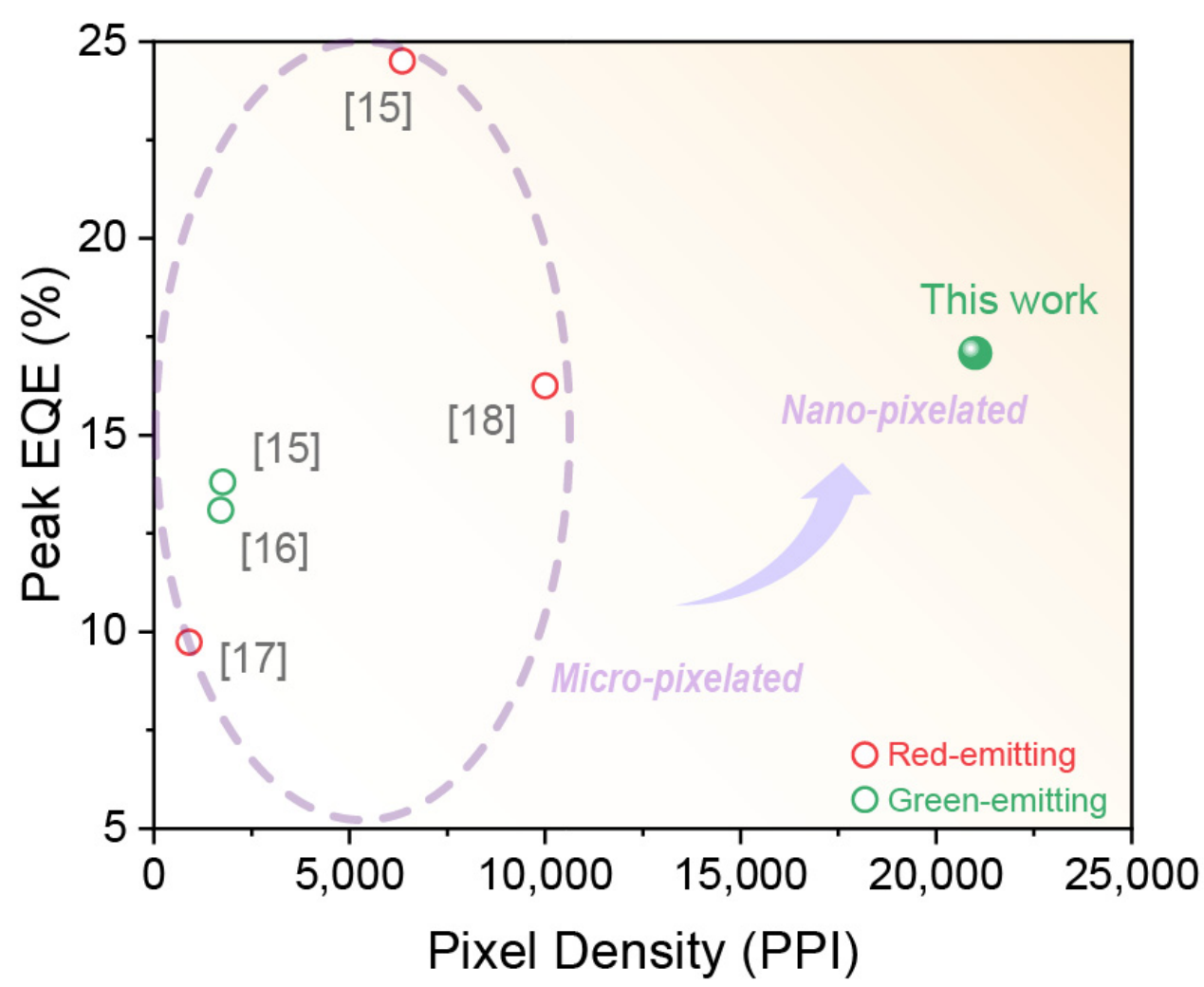

Supplementary Fig. 25 Comparation of different photolithographic QLEDs in pixel density and peak EQE.[15-18]

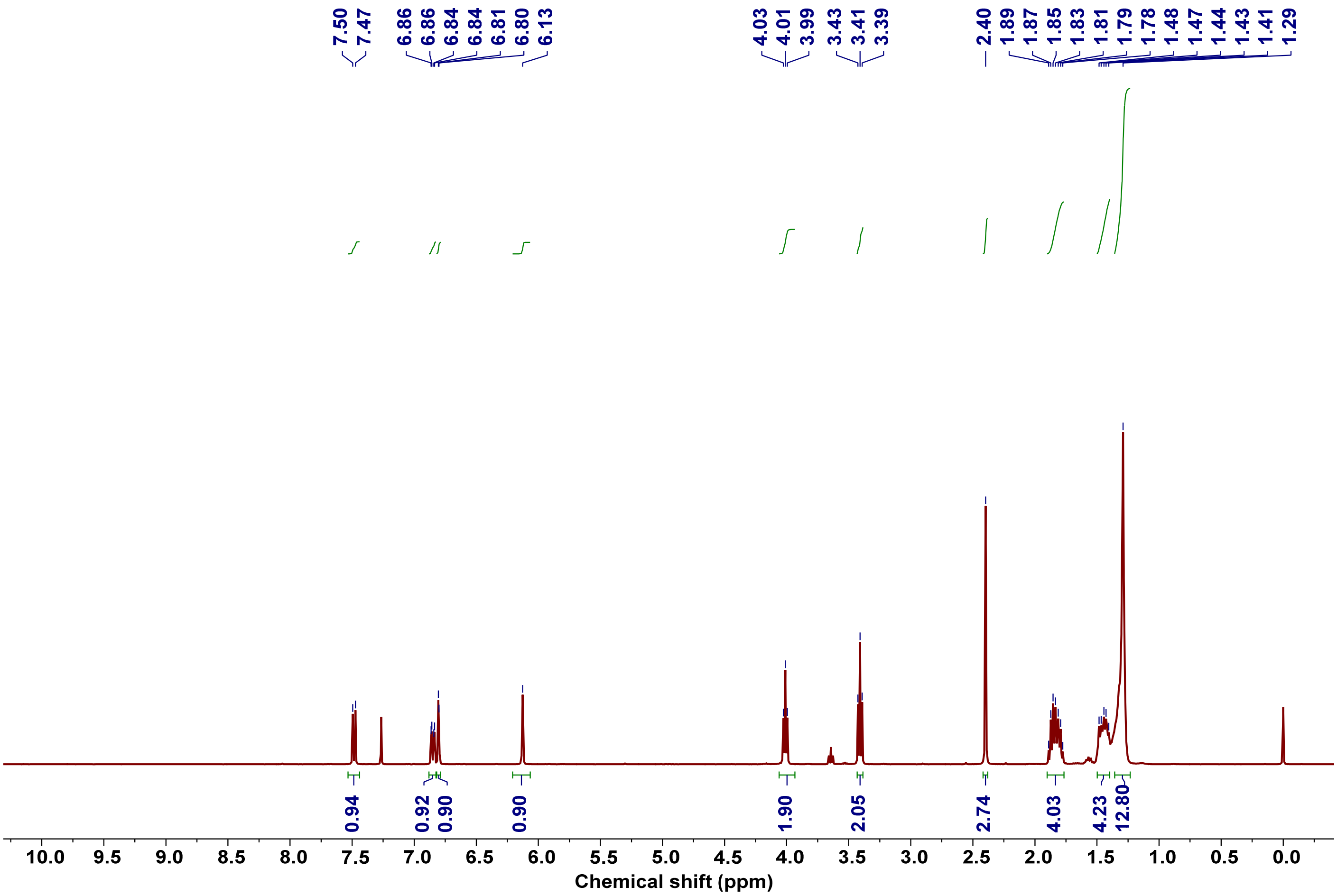

Supplementary Fig. 26 ^{1}H-NMR (400 MHz, Chloroform-*d*) of **1**.

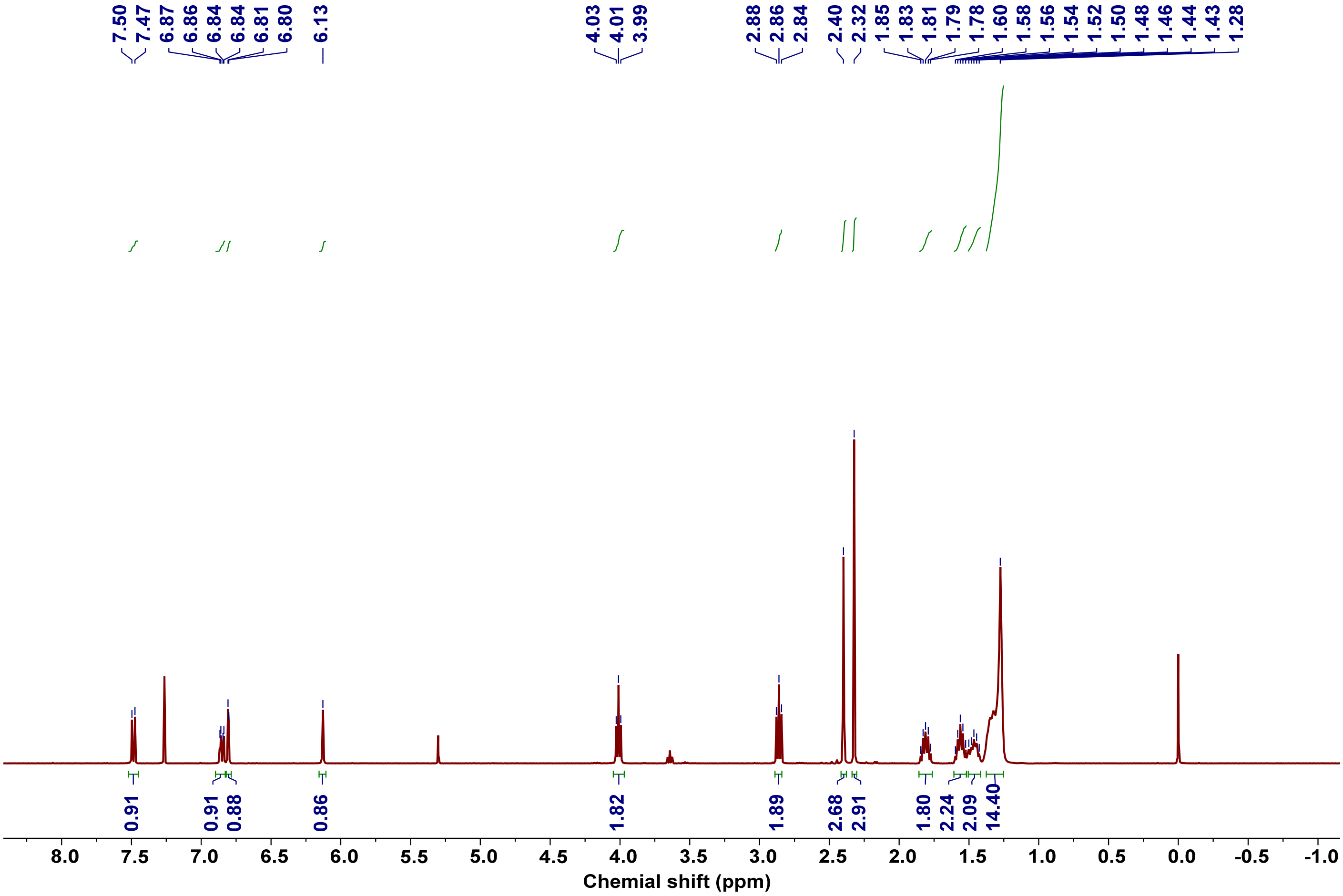

Supplementary Fig. 27 ^{1}H-NMR (400 MHz, Chloroform-*d*) of **2**.

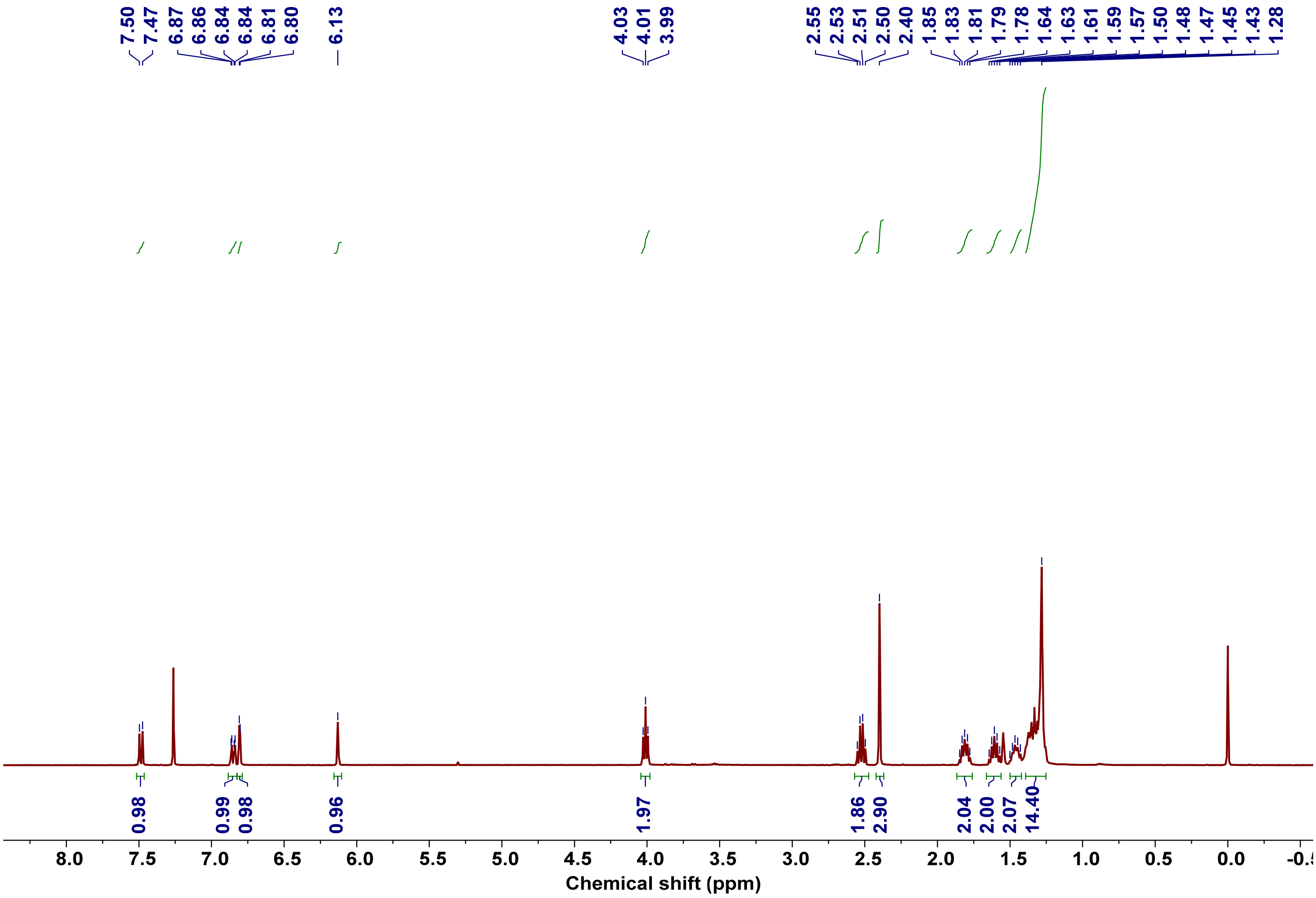

Supplementary Fig. 28 ^{1}H-NMR (400 MHz, Chloroform-*d*) of **PRL**.

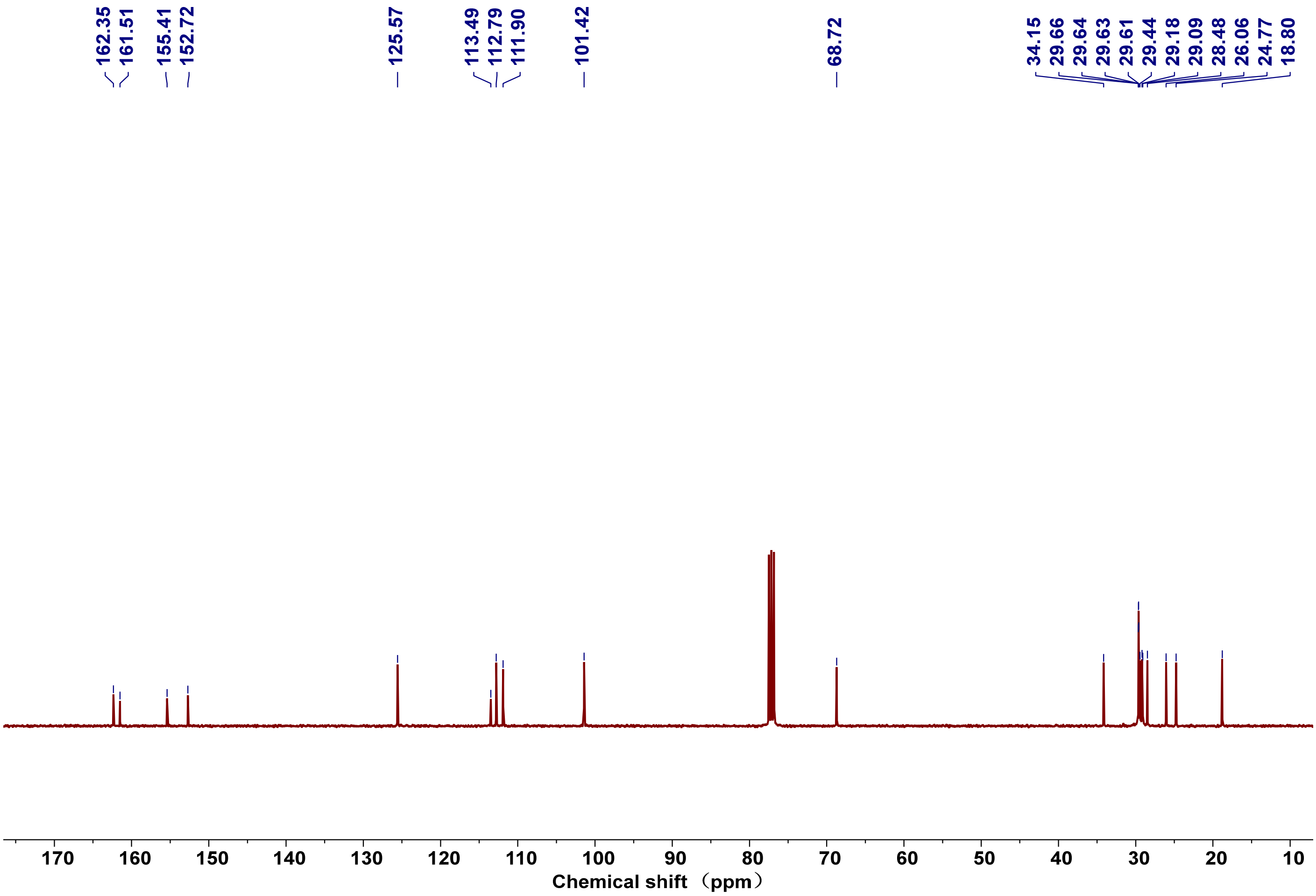

Supplementary Fig. 29 ^{13}C-NMR (101 MHz, Chloroform-*d*) of **PRL**.

Supplementary Tables

Supplementary Table 1. The parameters and outcomes of photolithographic QDs or perovskites.

Ref.	***QDs or perovskites***	***Photolithographic mechanism***	***UV dose ($mJ\ cm^{-2}$)***	***PPI***	***PL QY retention ratio***	***L ($cd\ m^{-2}$) at 5.0 V***	***Peak EQE of photolithographic (non-patterned) devices***	***Lifetime***
This work	**CdSe/ZnS (Green)**	**Reversible ligand photocycloaddition**	**~5**	**~21,000**	**107.4%** (Relative to OA-GQD); **96.2%** (Relative to PRL-GQD)	**125,016**	**24.41%**	**942 h** (T_{95}@ 1k nit)
1	CdSe/CdZnS (Red)	Nitrene C-H insertion	~2	>1,400	> 90%	~60,000	14.6%	~10 h (T_{95}@ 10k nit)
2	InP/ZnSeS (Green)	Nitrene C-H insertion	~2	3,240	> 95%	~4,200	8.3%	156 h (T_{90}@ 1k nit)
3	InP/ZnSe$_x$S$_{1-x}$ or CdSe/Cd$_x$Zn$_{1-x}$Se/ZnSe$_y$S$_{1-y}$ (Green, Red)	C-H insertion	35	~15,000	> 95%	~100,000 (Red), ~5,000 (Green)	~20% (Red), ~15% (Green)	-
4	CdSe/ZnS (Red)	Azide-alkyne click chemsitry	~36	~10,000	~106%	~200,000	20.05%	582 h (T_{95}@ 1k nit)
5	$FAPbBr_3$ (Green)	C-H insertion	~60	~400	~ 60%	~3,000	6.8%	5~7 min (T_{50}@ 100 nit)
6	InP/ZnSe/ZnS (Red)	Carbene-based insertion	100	~13,300	~90%	~20,000	15.3%	-
7	CdZnSe/CdZnS/ZnS (Red)	Nitrene- and carbene-based insertion	50 - 200	-	~90%	~6,000	11.7%<br>12.6%	1823 h (T_{95}@ 1k nit)<br>4852 h (T_{95}@ 1k nit)
8	$FAPbBr_3$ (Green)	C-H insertion	500	~4,000	~90%	~20,000	16%	26 min (T_{50}@ 100 nit)
9	CdSe (Red)	Nitrene C-H insertion	20	6,350	99%	~80,000	21%	3.06 h (T_{95}@ 10k nit)
10	CdSe/ZnS (Red)	C-H insertion	200	18,000	100%	~150,000	20.3%	620 h (T_{95}@ 1k nit)
11	CdSe/ZnS (Red, Green, Blue)	Oxygen-mediated photocrosslinking	40–270	9,534	>80%	~200 (Blue), ~10,000 (Green), ~10,000 (Red)	21.6% (Blue), 25.6% (Green), 20.2% (Red)	135 h (blue), 18261 h (green), 9280 h (red) (T_{95}@ 1k nit)

Supplementary Table 2. Comparisons of pixelated QLED performances based on direct photolithography.

Ref.	*Emission color*	*Peak EQE*	*Pixel density (PPI)*
This work	**Green**	**17.08%**	**~21,000**
15	Green	13.8%	1760
	Red	24.5%	6350
16	Green	13.09%	1707
17	Red	9.73%	~900
18	Red	16.25%	10,000

Supplementary Notes

Supplementary Note 1: Materials

Potassium thioacetate (98%), sodium hydrogensulphide ($NaHS \cdot xH_2O$, 70%), N, N-dimethylformamide (DMF, 99.8%, Extra Dry) and potassium carbonate (99%) were obtained from Energy Chemical (3A) Company. 1,12-Dibromododecane (97%) and 7-hydroxy-4-methylcoumarin (98%) were purchased from Aladdin Reagent Company. Toluene was obtained from Sinopharm Chemical Reagent Co., Ltd. Thermally crosslinked PF8Cz-X was purchased from Volt-Amp Optoelectronics Tech. Co., Ltd. Poly(3,4-ethylenedioxythiophene) poly(styrenesulfonate) (PEDOT:PSS AI 4083) was purchased from Xi'an Polymer Light Technology Co. Commercial red-emitting/green-emitting/blue-emitting core-shell CdSe/ZnS QDs (termed RQD/GQD/BQD or OA-RQD/OA-GQD/OA-BQD) (20 mg mL^{-1} in toluene) were provided by Hefei Full-Nano Co. Ltd. ZMO (Mg-doped ZnO) nanoparticles (30 mg mL^{-1} in ethanol) was obtained from Zhengzhou Yujing Co. Ltd.

ITO (30 Ω sq^{-1}) was obtained from Huayi Electronic Co., Ltd. and Liaoning Advanced Election Technology. ITO was cleaned with deionized water, acetone and isopropanol for 15 min, and then exposed under UV Ozone for 15 min before use.

Supplementary Note 2: Instrument characterization

UV-vis spectra were measured using a Shimadzu UV-2600i double-beam spectrophotometer. PL spectra were performed using FluoroMax Plus (HORIBA). QYs were measured by an absolute PL QY spectrometer (C9920-02, Hamamatsu). Liquid chromatography/quadrupole-time-of-flight mass spectrum (LC/Q-TOF MS) were performed using a TripleTOF 4600 (AB Sciex). ^{1}H- and ^{13}C-NMR spectroscopy (NMR) were performed using AVANCE NEO 400M (Bruker). The morphology was observed with Bruker DIMENSION Icon (AFM). Ultraviolet photoelectron spectroscopy (UPS) and X-ray photoelectron spectroscopy (XPS) were performed using an AXIS SUPRA+. Grazing-incidence small-angle X-ray scattering (GISAXS) measurements were performed using the Xeuss 3.0 UHR instrument (Xenocs, France) with an incident angle of 0.2°, Cu-Kα radiation wavelength of 1.54 Å, and a sample-to-detector distance of 500 mm equipped with an Eiger 500K detector. The current-voltage analysis experiments were performed using Keysight B1500A. Fourier transform infrared (FT-IR) spectra were performed using a IS50 (Thermo Fisher Scientific).

Reversible photolithography was performed with different UV LED lamps (Lamp I for ligand crosslinking: centered at 310 nm, ~0.5 to ~5 mW cm^{-2}; Lamp II for ligand decrosslinking: centered at 254 nm, ~1 to ~10 mW cm^{-2}) from Zigu Factory, Zhongshan, China. The power density was measured by LS125 with LS125-UVC-X0 Probe and UV-Integrator from Linshang Technology and Yeguan Factory, Shenzhen, China. Functional materials were spin-coated on the substrate by EZ4 SPIN COATER (Schwan technology). Images of patterned QDs and QLEDs were obtained on a fluorescence microscope (Olympus BX51 with DP74 camera).

CS-2000 (Konica Minolta) and Keithley 2400 B were applied to measure the luminance and current density of QLEDs, respectively. Note that the reported luminance and current density for pixelated QLEDs are instrument-derived, statistically averaged values based on the total electrode area, rather than the true values of the individual emitting pixels. And these values should be corrected by the pixel fill factor (FF). The EQE values, however, remain unaffected by this correction.

Supplementary Note 3. Synthesis of dynamic photo-reversible ligand (PRL)

(1) Synthesis of 7-((12-bromododecyl)oxy)-4-methyl-2H-chromen-2-one (1)

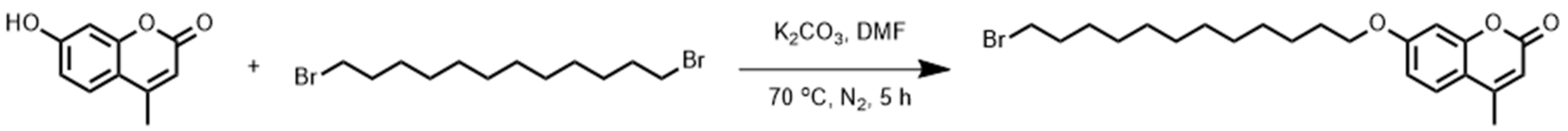

1

7-Hydroxy-4-methylcoumarin (2.66 g, 15 mmol), 1,12-dibromododecane (9.84 g, 30 mmol), potassium carbonate (20.79 g, 150 mmol) and DMF (100 mL) were placed into a round bottom flask. And the reaction mixture was stirred at 70 ºC for 5 hours under N_2. Then, the mixture was washed by deionized water and dichloromethane (DCM). The solvent was removed by vacuum. And the product (white powder, 2.80 g, yield = 44.1%) was obtained by column chromatography with PE/EtOAc as eluent. ^{1}H-NMR (400 MHz, Chloroform-*d*) δ 7.48 (d, *J* = 8.8 Hz, 1H), 6.85 (dd, *J* = 8.8, 2.3 Hz, 1H), 6.80 (d, *J* = 2.2 Hz, 1H), 6.13 (s, 1H), 4.01 (t, *J* = 6.5 Hz, 2H), 3.41 (t, *J* = 6.9 Hz, 2H), 2.40 (s, 3H), 1.83 (tt, *J* = 14.6, 6.8 Hz, 4H), 1.45 (m, 4H), 1.29 (m, 12H). Corresponding ^{1}H-NMR was shown in Supplementary Fig. 26.

(2) Synthesis of S-(12-((4-methyl-2-oxo-2H-chromen-7-yl)oxy)dodecyl) ethanethioate (2)

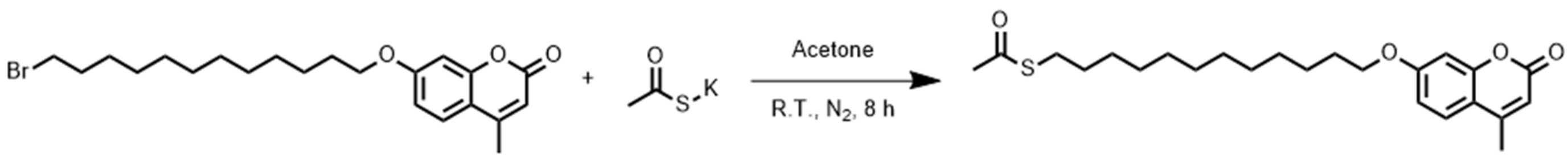

1 2

7-((12-bromododecyl)oxy)-4-methyl-2H-chromen-2-one (**1**) (1.06 g, 2.5 mmol), potassium thioacetate (0.29 g, 2.5 mmol) and acetone (10 mL) were placed into a round bottom flask. And the reaction mixture was stirred at room temperature for 8 hours under N_2. Then, the mixture was washed by deionized water and dichloromethane (DCM). The solvent was removed by vacuum. And the product (milky white powder, 0.95 g, yield = 90.5%) was obtained. ^{1}H-NMR (400 MHz, Chloroform-*d*) δ 7.48 (d, *J* = 8.8 Hz, 1H), 6.85 (dd, *J* = 8.8, 2.4 Hz, 1H), 6.81 (d, *J* = 2.4 Hz, 1H), 6.13 (s, 1H), 4.01 (t, *J* = 6.5 Hz, 2H), 2.86 (t, *J* = 7.4 Hz, 2H), 2.40 (s, 3H), 2.32 (s, 3H), 1.81 (p, *J* = 6.6 Hz, 2H), 1.56 (m, 2H), 1.47 (m, 2H), 1.28 (m, 14H). Corresponding ^{1}H-NMR was shown in Supplementary Fig. 27.

(3) Synthesis of 7-((12-mercaptododecyl)oxy)-4-methyl-2H-chromen-2-one (PRL)

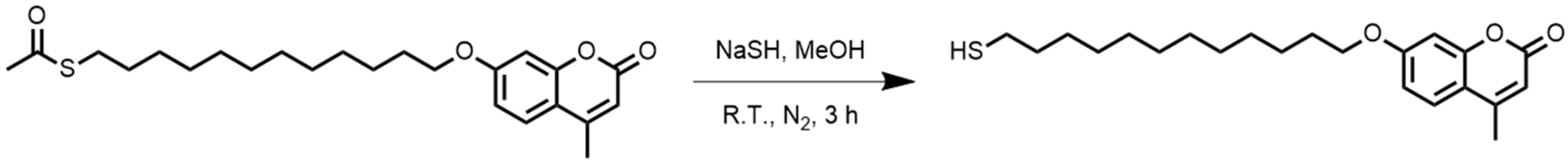

2 PRL

S-(12-((4-methyl-2-oxo-2H-chromen-7-yl)oxy)dodecyl) ethanethioate (**2**) (0.42 g, 1 mmol), dichloromethane (DCM) (5 mL) and methanol (5 mL) was placed into a round bottom flask under N_2 flow. Then, a solution of sodium hydrogensulphide ($NaHS{\cdot}xH_2O$, 0.4 g, ~4 mmol) in 10 mL methanol and dichloromethane (1:1) was added into the flask. And the reaction mixture was stirred at room temperature for 3 h under N_2 flow. Then, a solution of NH_4Cl (0.4 g, 8 mmol) in 5 mL water was added. The mixture was washed by deionized water and dichloromethane (DCM). The organic layer was washed with brine, separated and dried using anhydrous sodium sulphate. The solvent was removed by vacuum. And the product (white powder, 0.13 g, yield = 34.5%) was obtained by column chromatography with DCM as eluent. ^{1}H-NMR (400 MHz, Chloroform-*d*) δ 7.49 (d, *J* = 8.8 Hz, 1H), 6.85 (dd, *J* = 8.8, 2.3 Hz, 1H), 6.81 (d, *J* = 2.3 Hz, 1H), 6.13 (s, 1H), 4.01 (t, *J* = 6.5 Hz, 2H), 2.52 (q, *J* = 7.4 Hz, 2H), 2.40 (s, 3H), 1.81 (p, *J* = 6.7 Hz, 2H), 1.61 (p, *J* = 7.2 Hz, 2H), 1.47 (m, 2H), 1.28 (m, 14H). ^{13}C-NMR (101 MHz, Chloroform-*d*) δ 162.35, 161.51, 155.41, 152.72, 125.57, 113.49, 112.79, 111.90, 101.42, 68.72, 34.15, 29.66, 29.64, 29.63, 29.61, 29.44, 29.18, 29.09, 28.48, 26.06, 24.77, 18.80. Corresponding ^{1}H-NMR was shown in Supplementary Fig. 28. Corresponding ^{13}C-NMR was shown in Supplementary Fig. 29. LC/Q-TOF MS calcd. for $C_{22}H_{33}O_3S$ $[M+H]^+$: 377.2150, found: 377.2132.

Supplementary Note 4. Electrochemistry

Cyclic voltammetry experiments were performed using a Bio-logic electrochemical workstation at room temperature with a three-electrode cell in chromatographic grade acetonitrile containing $TBAPF_6$ (0.1 mol L^{-1}) as supporting electrolyte. The three-electrode cell consisted of a glass-carbon working electrode (3 mm dia.), a Pt wire counter electrode and an Ag wire reference electrode. The surface of the working electrode was polished with 0.3 and 0.05 μm alumina followed by ultrasonic cleaning in deionized water for three times. All related cyclic voltammetry data were calibrated by ferrocene (Fc), the standard redox potential of which is 0.4 V (vs. SCE). The sweep speed in all the cyclic voltammetry experiments was 100 mV s^{-1}.

HOMO and LUMO energy levels of PRL were calculated by following equations:

$$HOMO = -(E_{ox} - E_{ox}(Fc/Fc^{+}) + 4.8)\ eV$$

$$LUMO = -(E_{red} - E_{red}(Fc/Fc^{+}) + 4.8)\ eV$$

E_{ox}, E_{red} , $E_{ox}(Fc/Fc^{+})$ and $E_{red}(Fc/Fc^{+})$ are onset potentials of the oxidation peak of PRL, the reduction peak of PRL, the oxidation peak of ferrocene (0.33 V) and the reduction peak of Fc^{+} (0.47 V), respectively.

Supplementary Note 5. Ligand exchange of QDs with PRL

First, 50 μL of PRL solution (10 mg mL^{-1} in toluene) was added to 1 mL commercially purchased green-emitting core-shell CdSe/ZnS QDs (OA-GQD) (20 mg mL^{-1} in toluene) capped with oleic acid (OA) and oleylamine (OAm) ligands. The mixture was stirred at room temperature for 4 hours to facilitate maximum ligand exchange. Subsequently, 2 mL anhydrous ethanol was added, followed by centrifugation to remove excess PRL and stripped OA/OAm ligands. The obtained PRL-capped QDs was then redispersed in 1 mL toluene as the photosensitive PRL-GQD solution. The content of PRL in organic surface ligands of QDs is approximately 8 mol%.

Supplementary Note 6. Simulation

Ab initio simulation is performed with Cambridge Sequential Total Energy Package (CASTEP, ver. 20.1.1).[12] All geometry optimizations are performed with generalized gradient approximation (GGA) Perdew Burke Ernzerhof (PBE) exchange-correlation functional with TS correction scheme[13] under Koelling-Harmon scalar relativistic treatment, with on-the-fly generated (OTFG) ultrasoft pseudopotentials. In addition,

the cutoff energy is selected as 630 eV, while the total energy, maximum displacement, maximum stress, and maximum force are set to 5×10^{-7} eV/atom, 5×10^{-4} Å, 0.02 GPa and 0.01 eV/Å, respectively, during convergence. 3*3*3 Monkhorst-Pack k-point grids are applied for molecules geometry optimization. The binding energy is defined be the formula of $E_b = E_{System} - E_{Molecule} - E_{Surface}$. All the atomic structures were visualized with Visualization for Electronic and Structural Analysis software (VESTA, version 3.5.8).[14]

Supplementary Note 7. Preparation of QLEDs

In order to prepare QLEDs, PEDOT:PSS (AI 4083) was spin-coated onto a cleaned ITO substrate at 2000 rpm and annealed at 150 °C for 15 min at first. The substrate was then transferred to a nitrogen-filled glove box. Subsequently, PF8Cz-X (8 mg mL^{-1} in chlorobenzene) was spin-coated at 1500 rpm on PEDOT:PSS, and thermally cross-linked at 200 °C for 30 min. Then, the functional film was washed with chlorobenzene to remove the un-crosslinked component.

After that, the QD emissive layer was spin-coated at 2000 rpm. And the reversible photolithography of QDs was taken for preparing crosslinked or decrosslinked QLEDs. The pixelated photomasks with different pixel arrays were introduced to prepare pixelated QLEDs. For pixelated QLEDs, a PMMA solution (0.5 mg mL^{-1} in anhydrous acetone) was spin-coated on the pixelated QDs at 5000 rpm for 30 s, and subsequently annealed at 60 °C for 30 min. The PMMA layer is introduced to prevent current leakage caused by the direct contact between the hole transport layer and electron transport layer.

Then, ZMO nanoparticles (30 mg mL^{-1} in ethanol) were spin-coated at 4000 rpm and annealed at 80 °C for 30 min. And a silver (Ag) layer was deposited as the electrode by using vapor deposition (thickness: 150 nm). Finally, the QLED was encapsulated to isolate water and oxygen from the air.

Supplementary References

1. Yang, J. et al. High-resolution patterning of colloidal quantum dots via non-destructive, light-driven ligand crosslinking. *Nat. Commun.* **11**, 2874 (2020).

2. Yang, J. et al. Nondestructive photopatterning of heavy-metal-free quantum dots. *Adv. Mater.* **34**, 2205504 (2022).

3. Hahm, D. et al. Direct patterning of colloidal quantum dots with adaptable dual-ligand surface. *Nat. Nanotechnol.* **17**, 952–958 (2022).

4. Nie, Q. et al. Direct optical patterning of quantum dot light-emitting diodes based on ultrafast, low-energy, site-controlled click chemistry reaction. *Adv. Funct. Mater.* **35**, 2420829 (2025).

5. Liu, D. et al. Direct optical patterning of perovskite nanocrystals with ligand cross-linkers. *Sci. Adv.* **8**, eabm8433 (2022).

6. Fu, Z. et al. Direct photopatterning of colloidal quantum dots with electronically optimized diazirine cross-linkers. *J. Am. Chem. Soc.* **146**, 28895–28905 (2024).

7. Lu, S. et al. Beyond a linker: the role of photochemistry of crosslinkers in the direct optical patterning of colloidal nanocrystals. *Angew. Chem. Int. Ed.* **61**, e202202633 (2022).

8. Liu, D. et al. Nondestructive direct optical patterning of perovskite nanocrystals with carbene-based ligand cross-linkers. *ACS Nano* **18**, 6896–6907 (2024).

9. Chen, Z. et al. Rigid crosslinker-assisted nondestructive direct photolithograph for patterned QLED displays. *Light Sci. Appl.* **14**, 251 (2025).

10. Gu, C. et al. Ultra-high-resolution nondestructive photolithography of quantum dots enabled by photo-shield crosslinker. *Mater. Today* **89**, 100 (2025).

11. Guan, J. et al. Direct ambient photopatterning of RGB quantum dots for light-emitting diodes with EQE exceeding 20%. *Nat. Commun.* **16**, 9689 (2025).

12. Clark, S. J. et al. First principles methods using CASTEP. Z. *Kristallogr. Cryst. Mater.* **220**, 567–570 (2005).

13. Tkatchenko, A. & Scheffler, M. Accurate molecular van der waals interactions from ground-state electron density and free-atom reference data. *Phys. Rev. Lett.* **102**, 073005 (2009).

14. Momma, K. & Izumi, F. VESTA 3 for three-dimensional visualization of crystal, volumetric and morphology data. *J. Appl. Crystallogr.* **44**, 1272–1276 (2011).

15. Wu, C., et al. Highly efficient and ultrahigh-resolution quantum dot light-emitting diodes via photoisomeric transformation. *Light Sci. Appl.* **15**, 157 (2026).

16. Huo, Y. et al. Ambient direct lithography patterning of ultra-stable perovskite quantum dots for high-resolution light-emitting diodes. *Adv. Funct. Mater.* **35**, 2504261 (2025).

17. Kim, J.-M. et al. Role of conjugated structure of cross-linkers in patterned QLEDs. *Nano Lett.* **25**, 10435–10441 (2025).

18. Qie, Y. et al. Ligand-nondestructive direct photolithography assisted by semiconductor polymer cross-linking for high-resolution quantum dot light-emitting diodes. *Nano Lett.* **24**, 1254–1260 (2024).
