# Supplementary material for: Reversible photolithography of quantum dots achieves high-performance electroluminescence
Source: Nat Commun. 2026 Sep 24;17:11097. doi: 10.1038/s41467-026-77916-z (PMC13696730; doi:10.1038/s41467-026-77916-z)
Supplement: Supplementary file 3 [file 41467_2026_77916_MOESM3_ESM.pdf]

nature portfolio

Peer Review File

Reversible photolithography of quantum dots achieves high-performance electroluminescence

Corresponding Author: Professor Ting Zhang

This file contains all reviewer reports in order by version, followed by all author rebuttals in order by version.

Version 0:

Reviewer comments:

Reviewer #1

(Remarks to the Author)
In this work, the authors develop a reversible photolithography strategy for quantum dots (QDs), which effectively addresses the long-standing performance degradation issue in direct photolithography. The core idea of using an in-situ decrosslinking step to restore surface states and recover the optoelectronic properties is quite refreshing and conceptually distinct from previously reported photolithography frameworks. The results are also compelling, with a peak EQE of 24.41%, ranking among the best values reported to date. In my view, the manuscript is suitable for publication in Nature Communications after minor revision.
The comments are as follows:
1. In the Introduction, the authors list several adverse effects, including structural defects and compromised conductivity. These effects, however, originate from different physicochemical pathways. A more detailed discussion would help readers better appreciate the distinct mechanisms involved.
2. A recent study by Zhang et al. demonstrated reversible photoisomerization using azobenzene-functionalized ligands for QD patterning (Chem. Commun. 2026, 62, 4089). Whether this alternative approach could also enable the recovery of electroluminescence performance?
3. The coumarin-based ligand is used at a loading of ~8 mol%. What is the optimization rationale behind this specific concentration?
4. The photo-crosslinking process employs 310 nm UV light, which is not commonly used in industrial manufacturing. Have the authors explored more practical wavelengths, such as i-line?
5. In Figure 5, how do the patterned features compare with those obtained by conventional direct photolithography? Does the decrosslinking step introduce any positive or negative effects on pattern quality? Expanding the discussion along these lines would better highlight the central role of the reversible strategy.
6. There are a few minor formatting issues: please unify the capitalization of “PL intensity” across the subfigures in Figure 3, and check the reference format of Ref. 40.

Reviewer #2

(Remarks to the Author)
This work introduces an approach of crosslinking and de-crosslinking of QDs, PRL-based patterning, to demonstrate high-resolution QD-based displays. This approach appears new and the presented EL performance at the initial stage appears good. However, it should be noted that the focus of this work is about the photopatterning of QDs, not the EL performance because the high luminance and the high EQE are not simply related to the PRL-based patterning but the quality of QD and the other device fabrication process can be even more important factors. Overall, this manuscript does not contain any advance of the realization of practical QLEDs, compared to the recent well-performing, well-patterned QLED reports.

A list of more detailed serious issues:
1) The rationale for the crosslinking and de-crosslinking of QD films is not clear from the perspective of the practical fabrication of QD-based displays. The process involving 254 nm UV irradiation is not compatible with industrial fabrication processes. Furthermore, several previous studies have reported no degradation, or even improvements, in QD properties after photo-crosslinking. In contrast, in this work, photo-crosslinking degrades the QDs, and their pristine properties are not-fully restored after de-crosslinking. Moreover, the EL performance of the de-crosslinked devices remains inferior to that of the

pristine devices and exhibits a shorter operational lifetime. Therefore, it is difficult to identify any clear advantages of this crosslinking/de-crosslinking approach over previously reported direct photopatterning methods.

2) De-crosslinking of QDs may lead to device instability because the initially optimized surface ligands could become mobile during device operation. Indeed, as shown in Figure 4h, the operational lifetime of the de-crosslinked device is shorter than that of the pristine device. Therefore, from the standpoint of device stability, the proposed approach does not demonstrate a clear advantage over previously reported high-performance QLEDs.

3) The authors often claimed “high fidelity and high resolution” in the manuscript. The simply high number of PPI is not important. Rather, the demonstration of the excellent electrical performance with the practically approachable PPIs in the LED device is even more important. Based on Figure 5c, pattern fidelity is very poor. And, the authors have not shown any side views of the pixels in Figure 5f. The simple top image does not guarantee the quality of pixel features are useful for the generation of the real displays. There are several published papers regarding “how to define pattern fidelity”. Please analyze the fidelity of patterns based on such standized or well-known methods. Note that the surface roughness of the QD films is one of the simplest fidelity factor, too short of analyses.

4) Are there any fundamental reasons why this approach can achieve a higher resolution than previously reported methods? This work simply employs ligand-mediated crosslinking, i.e., direct photopatterning, which is already a well-established technique. Therefore, it is not clear that the proposed crosslinking/de-crosslinking strategy can intrinsically provide better pattern fidelity or higher resolution than existing direct photopatterning approaches.

5) This work lacks a strategy for realizing full-color displays, as it only demonstrates a single-color EL device. In particular, after de-crosslinking, the pre-patterned QDs may be washed away during the subsequent processing steps required to pattern QDs of different colors. The authors should demonstrate full-color device fabrication while preserving the integrity of the previously formed QD patterns.

6) “This performance trade-off is commonly observed in direct QD photolithography via photo-crosslinking,[24,32,56]”. This cannot be agreeable. Please find recent reports that show no trade-off. The performance trade-off has been often reported but recently many reports do not show trade-off. Rather, some recent reports show no changes or improvements after patterning.

7) For the photo-crosslinking, they described “the film was exposed to suitable UV irradiation (centered at 310 nm) with or without photomasks for required patterns in the air or the nitrogen filled glove box.”. It is not clear whether they use QD patterns processed in air or in the nitrogen atmosphere. They need to indicate the condition carefully for each datum. Also, it is recommendable to show at least PLQYs depending on both the conditions.

8) The authors claimed “we fabricated QLEDs with EL pixel arrays spanning resolutions from 3,300 PPI (pixel size: ~4.5 μm) to 21,000 PPI (pixel size: ~0.8 μm), as illustrated in Figure 5f.”. However, there is no clear description of fabricating such high-resolution EL devices. Also, it is quite unclear whether they really fabricated such high-pixellated QLEDs or just fabricated 21,000 PPI that can be applicable for real QLEDs, because they mentioned following “Notably, pixelated QLEDs suffer from pronounced leakage currents due to direct HTL/ETL contact in non-patterned regions, a challenge that typically demands further device architecture optimization.[61,62] And research in this field remains primarily concentrated on non-pixelated devices, as summarized in a recent review.[29] Consequently, comprehensive performance characterization of pixelated devices was not pursued in this study to prioritize demonstration of photosensitive materials and photolithographic fidelity.”

9) The electrical performance of the pixelated QLEDs fabricated using the crosslinking/de-crosslinking process, regardless of pixel size, is not presented. The authors should provide the electrical characteristics of these pixelated devices using a reliable test platform to demonstrate the practicality and effectiveness of their approach.

Reviewer #3

(Remarks to the Author)
This manuscript reports a reversible direct photolithography strategy for colloidal quantum dots based on a coumarin-functionalized photo-reversible ligand, termed PRL. The proposed process uses approximately 310 nm UV irradiation to induce coumarin photocycloaddition and inter-QD crosslinking for pattern formation, followed by 254 nm deep-UV irradiation to trigger decrosslinking and recover the ligand state and optoelectronic properties. The authors report low exposure dose, high PLQY retention, recovered conductivity and QLED performance after decrosslinking, a non-pixelated green QLED with a peak EQE of 24.41% and a luminance of 125,016 cd m^{-2} at 5.0 V, and EL pixel arrays with a claimed density of 21,000 PPI.
The concept is timely and potentially important. It addresses a central limitation in direct QD photolithography: the trade-off between crosslinking-enabled patternability and degraded optoelectronic performance. The manuscript is generally well organized and contains a broad set of optical, chemical, structural, electrical, and device-level characterizations.
However, the central claims are not yet sufficiently supported. The high-performance electroluminescence is demonstrated in non-patterned, full-area decrosslinked QLEDs, whereas the ultrahigh-resolution 21,000 PPI EL pixel arrays are not quantitatively characterized and appear to remain in the crosslinked patterned state. In addition, the reversible surface chemistry of PRL on QD films is inferred rather than directly demonstrated; and some mechanistic interpretations and simulation methods require correction. I therefore recommend major revision.

1. The core chemical claim is that surface-bound PRL undergoes reversible coumarin dimerization and photocleavage on QD films. The current evidence mainly includes UV-vis changes of free PRL, macroscopic solubility changes of QD films, PLQY retention, GISAXS, conductivity, and QLED performance recovery. These data are consistent with the proposed mechanism, but they do not directly prove that QD-bound PRL undergoes a reversible coumarin crosslinking/decrosslinking cycle. This issue is particularly important because PRL contains only one coumarin terminal group and the reported surface content is approximately 8 mol%. The authors should clarify whether the dominant dimerization event occurs between ligands on neighboring QDs or between ligands on the same QD. These two cases would have different implications for film insolubilization, inter-QD spacing, and charge transport.
2. State-of-the-art is overstated. (a) The decrosslinked luminance (125,016 cd/m²) is below the authors' own pristine control (134,796 cd/m²): the process recovers ~93%, it does not set a record. (b) The decrosslinked peak EQE (24.41%) is essentially equal to pristine (24.43%) and not higher than recent reports (e.g., the ref 61 at 24.5%; Guan et al. up to 25.6% green in the authors' own Table S1). (c) Table S1 lists others reaching ~200,000 cd/m². Please reframe the claims. The record 21,000 PPI claim should also be clearly limited to direct QD photolithography demonstrations.
3. The authors extract an energy Eb from Arrhenius fitting of temperature-dependent PL intensity and refer to it as the exciton binding energy. They further attribute its change to a local dipole/dielectric modification caused by coumarin dimerization, which is then linked to the low-voltage EQE decrease. This causal chain is currently under-supported. The energy extracted from thermal PL quenching is more appropriately described as a thermal activation energy for PL quenching, unless additional evidence is provided to justify its equivalence to the exciton binding energy. Temperature-dependent PL intensity in QD solids can be affected by trap activation, nonradiative recombination, carrier localization, energy transfer, and ligand dynamics. It cannot be assigned uniquely to exciton binding energy from a single Arrhenius fit.
4. The DFT section also appears inconsistent: the equation in the Supplementary Information describes molecular stacking rather than ligand adsorption on a ZnS surface, so it cannot directly support the claim that PRL binds more strongly to ZnS than OA.
5. The process relies on crosslinking to make the QD film insoluble for development, followed by decrosslinking to return the QDs toward their original ligand state. The supplementary information further states that decrosslinked PRL-QDs can be immersed in n-octane or toluene and removed, enabling QD recycling or subsequent patterning. This raises a fundamental process question: if the final QD pattern is decrosslinked, can it survive subsequent spin-coating and development steps during RGB fabrication?
6. For a "reversible photolithography platform," the manuscript currently demonstrates approximately one crosslinking–decrosslinking cycle. Coumarin photocleavage under 254 nm irradiation may be incomplete and can be accompanied by fatigue or irreversible side reactions. The authors should provide multi-cycle data over at least 3–5 crosslinking/decrosslinking cycles.
7. The RGB demonstration mainly consists of PL images and CIE coordinates. Without RGB QLED devices or sequential full-color process validation, the manuscript should avoid implying complete full-color QLED display fabrication.

Reviewer #4

(Remarks to the Author)
The authors propose a novel reversible photolithography strategy based on reversible photo-crosslinkable coumarin ligand and address the core bottleneck of QD performance degradation during direct photolithography patterning. The design is rigorous. The demonstrated device performance (EQE of 24.41%, resolution of 21,000 PPI) represents state-of-the-art results in the field. This work will provide innovative insights into micro/nanofabrication for high-performance QD devices. Therefore, I recommend acceptance after minor revisions.
1.The crosslinking/decrosslinking procedure is not clearly described. In particular, it is ambiguous whether development is required after the decrosslinking step. Please provide a complete, step by step operational flowchart.
2.As shown in Figure 3b, PL intensity is used as the sole metric to evaluate crosslinking and decrosslinking, which is an indirect method. I suggest supplementing these data with direct measurements of film thickness retention to more accurately monitor the changes in the QD films.
3.The "Pristine" device used for comparison is based on PRL-capped QDs, but the ligand exchange itself is already part of the photolithographic workflow. To provide a fair baseline, it would be valuable to include a reference device using the original QDs (without any ligand exchange or photo-treatment).
4.The authors mention that pixelated QLEDs suffer from pronounced leakage currents. I encourage expanding the discussion on potential device architecture optimizations to mitigate this issue. Notably, a recent study (doi: 10.1038/s41586-026-10333-w) addresses non-uniform electric field distribution in pixelated QLEDs; including this reference would strengthen the practical outlook of the work.
5.Although the decrosslinking step restores luminance and current density to 93% and 95% of the pristine values, respectively, the recovery is not complete (~100%). Why? In addition, they should avoid absolute terms such as "nondestructive" to maintain scientific rigor.
6.Several minor grammatical issues need correction, for example, "systemically" should be "systematically" (page 9), "through a … lamp" should be "with a … lamp" (page 11), and "representing among" should be "representing one of" or "which is among" (page 12).

Version 1:

Reviewer comments:

Reviewer #1

(Remarks to the Author)
All of my concerns have been addressed adequately. Therefore, the revised manuscript is suitable for publication.

Reviewer #2

(Remarks to the Author)
Please refer to the attached file.

Reviewer #3

(Remarks to the Author)
The revised manuscript is substantially improved, particularly by the addition of film-state spectroscopy and a decrosslinked 21,000-PPI QLED. But several issues still affect the central interpretation, I continue to recommend a revision.
1.The manuscript renames the fitted quantity as thermal activation energy Ea but then states that its variation is predominantly linked to exciton binding energy Eb and reflects compromised exciton binding capability. This inference is not established. A thermal-quenching activation energy is not generally identical to Eb, and similar room-temperature fs-TA maps do not measure Eb or exclude trapping, localization, nonradiative pathways, energy transfer, or ligand-related dynamics. Unless an independent, diagnostic Eb measurement is provided, Ea should be treated as a phenomenological thermal-quenching parameter and the Ea-to-Eb/dielectric causal chain should be removed or explicitly labelled as a hypothesis.
Equation (1) is printed as I(t) = I0/[1 + A exp(Ea/kBT)]. The independent variable should be temperature, I(T), and the usual one-channel thermal-quenching form contains exp[-Ea/(kBT)]. Please determine whether this is a typographical error or the equation actually used for fitting. If the displayed positive sign was used, the fits and reported Ea values must be recalculated.

2.The reported 107.4% PLQY 'retention' is normalized to OA-GQD, although PRL-GQD is the actual pre-photolithography state. From Fig. 3c, the process retention is 107.4/111.6 = approximately 96.2%. Under ambient conditions, it is 91.8/110.2 = approximately 83.3%, not 91.8%. The text and Supplementary Table 1 should distinguish PLQY relative to the OA feedstock from retention through photolithography and should use the latter for process comparisons. Likewise, Fig. 3f reports raw current in pA, not conductivity, and the decrosslinked current does not return to the PRL value.
Finally, approximately 1 mJ cm-2 retains only about 62% of the PL and about 59% of the saturated film thickness, whereas near-saturation requires approximately 5 mJ cm-2. Thus, 1 mJ cm-2 is an onset dose rather than a demonstrated dose-to-clear or dose-to-size. The sensitivity comparison should use a defined endpoint and the actual dose used for the high-resolution patterns/devices.
3.The film-state FT-IR and UV-vis data support a reversible change of the coumarin chromophore, which is a useful addition. They do not, by themselves, provide product-specific proof of cyclobutane formation or show that inter-QD reaction dominates over intra-QD reaction. In Supplementary Fig. 7, a dense film and a 0.1 mg mL-1 dispersion are compared using raw Delta Abs, although their initial coumarin absorbance, optical path, scattering, local concentration, and absorbed photon number are not equivalent. Faster change in the film is therefore consistent with a proximity-enhanced inter-QD contribution but does not confirm its dominance. If the authors do not provide a normalized or more discriminating experiment, the mechanistic language should be revised to this qualified conclusion.
4.A feature width of approximately 0.8 micrometres is not the pitch corresponding to 21,000 PPI; 21,000 PPI corresponds to a center-to-center pitch of approximately 1.21 micrometres. Width, pitch, gap, and the PPI conversion should be stated separately.
More importantly, current density and EQE for a discontinuous emissive array depend on whether the denominator is the total electrode area or the summed emitting-pixel area, while luminance depends on the sampled optical area and pixel fill factor.
5.Supplementary Note 7 places PMMA coating after preparation of decrosslinked pixels but does not identify the solvent. Because the decrosslinked QDs are reported to regain solubility in nonpolar solvents, the authors should identify the solvent and verify that the approximately 0.8-micrometre features remain after PMMA coating.

Reviewer #4

(Remarks to the Author)
Direct photolithography have attracted much attention as a photoresist-free route for high-precision integration in electronic and optoelectronic platforms. The paper reports a reversible photolithography strategye to achieve highefficient and high-fidelity QD photolithography. In the revised version, the authors have addressed all the comments I am concerned, and now it can be accepted in the current version.

Version 2:

Reviewer comments:

Reviewer #2

(Remarks to the Author)

My concerns are all addressed.

Reviewer #3

(Remarks to the Author)
The manuscript has been further improved, and the authors have satisfactorily addressed the pixel geometry and PMMA-processing questions.

Open Access This Peer Review File is licensed under a Creative Commons Attribution 4.0 International License, which permits use, sharing, adaptation, distribution and reproduction in any medium or format, as long as you give appropriate credit to the original author(s) and the source, provide a link to the Creative Commons license, and indicate if changes were made.
In cases where reviewers are anonymous, credit should be given to 'Anonymous Referee' and the source.
The images or other third party material in this Peer Review File are included in the article's Creative Commons license, unless indicated otherwise in a credit line to the material. If material is not included in the article's Creative Commons license and your intended use is not permitted by statutory regulation or exceeds the permitted use, you will need to obtain permission directly from the copyright holder.
To view a copy of this license, visit https://creativecommons.org/licenses/by/4.0/

Reversible photolithography of quantum dots achieves high-performance electroluminescence

(Manuscript ID: NCOMMS-26-047874)

A Point-by-point Response to the Reviewers' Comments

To reviewer 1

Comment: *In this work, the authors develop a reversible photolithography strategy for quantum dots (QDs), which effectively addresses the long-standing performance degradation issue in direct photolithography. The core idea of using an in-situ decrosslinking step to restore surface states and recover the optoelectronic properties is quite refreshing and conceptually distinct from previously reported photolithography frameworks. The results are also compelling, with a peak EQE of 24.41%, ranking among the best values reported to date. In my view, the manuscript is suitable for publication in Nature Communications after minor revision.*

The comments are as follows:

1. In the Introduction, the authors list several adverse effects, including structural defects and compromised conductivity. These effects, however, originate from different physicochemical pathways. A more detailed discussion would help readers better appreciate the distinct mechanisms involved.

Response:

Dear reviewer, thanks a lot for your time and efforts to review our manuscript. We also appreciate your positive recognition, particularly that "*The core idea … is quite refreshing and conceptually distinct from previously reported photolithography frameworks*" and "*The results are also compelling*". We believe that with the help of you and other experts, the quality of our work has been significantly improved.

We thank the reviewer for the constructive suggestion to provide a more detailed discussion of the potential adverse effects associated with photolithographic processing. We fully agree that these effects originate from distinct photochemical approaches. For instance, ligand decomposition typically generates compact ionic ligands. Unlike long-chain organic ligands, these ionic species are less effective at passivating surface traps, which can promote nonradiative recombination [Ref: Chem. Rev. 2026, 126, 3624]. Ligand exchange processes mediated by photoacid generators often suffer from acid-induced degradation and the formation of surface defects [Ref: Adv. Mater. 2020, 32, 2003805]. Ligand crosslinking approaches lead to highly crosslinked ligand networks, which tend to increase electrical resistance [Ref: Small 2026, 22, e00011; ACS Nano 2025, 19, 22253; Small Methods 2023, 7, 2300206]. Thanks again for your comment. We have now revised the relevant discussion.

Change made on the third paragraph of Page 2: "For instance, compact ionic ligands generated from ligand decomposition are less effective at passivating surface traps.[29] Ligand exchange processes mediated by photoacid generators often suffer from acid-induced degradation and the formation of surface defects.[19] Highly crosslinked ligand networks tend to increase electrical resistance.[24,35,37]"

Comment: *2. A recent study by Zhang et al. demonstrated reversible photoisomerization using azobenzene-functionalized ligands for QD patterning (Chem. Commun. 2026, 62, 4089). Whether this alternative approach could also enable the recovery of electroluminescence performance?*

Response:

Dear reviewer, thank you for your valuable comments. Zhang et al. introduced an azobenzene-functionalized ligand onto the surface of QDs [Ref: Chem. Commun. 2026, 62, 4089]. And the reversible ligand photoisomerization process under UV and visible light has been successfully exploited to modulate colloidal solubility. In principle, this system could serve as a potential alternative for the reversible photolithography strategy proposed in our work. However, we observe that **this system suffers from harmful energy transfer due to the inherent absorption of azobenzene in the visible region**, which overlaps with the QD emission spectrum. Moreover, as noted by the authors, **its energy level misalignment with QDs can give rise to unfavorable hole transfer behavior**. Therefore, this system is not ideally suited for reversible QD photolithography. We have incorporated the above discussion and the reference into the revised manuscript.

Change made on the first paragraph of Page 4: "Recently, Zhang et al. reported a reversible photoisomerization process using an azobenzene-functionalized ligand for QD patterning,[41] which is conceptually feasible for reversible photolithography. However, this system suffers from unfavorable energy transfer and hole transfer, arising from the intrinsic visible-light absorption of azobenzene and its energy level misalignment with QDs."

Change made on Page 19: "[41] Fu, Z., Zheng, Z., Xie, Y., Abudukeremu, H., Zhou, L., Zang, S., Tian, K.; Liu, Q., Li, Y. & Zhang, H. Direct photopatterning and erasing of quantum dots via ligand photoisomerization. *Chem. Commun.* **62**, 4089 (2026)."

Comment: *3. The coumarin-based ligand is used at a loading of ~8 mol%. What is the optimization rationale behind this specific concentration?*

Response:

Dear reviewer, thanks for your interesting comments. The patterning quality of QDs is closely tied to the amount of photosensitive ligand (or additives). This phenomenon has been previously reported for many times. In this work, we examined the photolithographic patterns over a range of PRL loadings, from ~1 mol% to ~14 mol%. As shown in Figure R1, the PL intensity of QD patterns initially rose with increasing ligand content and then then kept stable, whereas the edge sharpness progressively degraded. Therefore, the ~8 mol% loading proved to be optimal, enabling the fabrication of high-quality QD pixel patterns. We have added corresponding discussion in the revised manuscript and Supplementary Information.

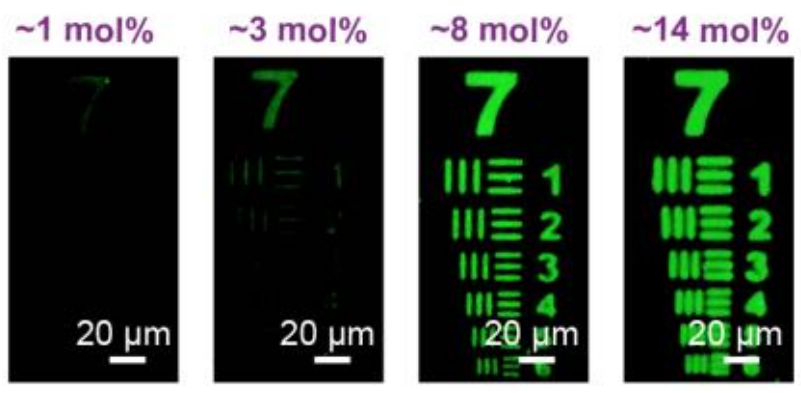

Figure R1. Fluorescence microscopic images of photolithographic QD patterns with different RPL contents.

Change made on the last paragraph of Page 12-13: "The patterning quality is closely tied to the amount of PRL. As displayed in Supplementary Fig. 18, the PL intensity of QD patterns initially rose with increasing ligand content and then kept stable, whereas the edge sharpness progressively degraded. The ~8 mol% loading proved to be optimal."

Change made on Page 10 in the revised Supplementary Information:

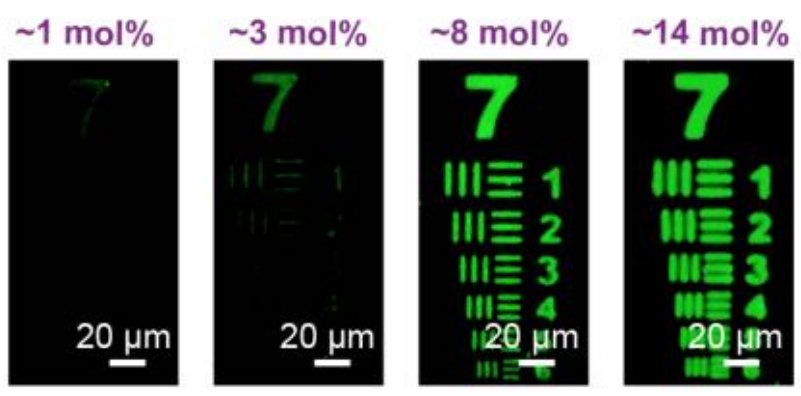

Supplementary Fig. 18 Fluorescence microscopic images of photolithographic QD patterns with different RPL contents.

Comment: *4. The photo-crosslinking process employs 310 nm UV light, which is not commonly used in industrial manufacturing. Have the authors explored more practical wavelengths, such as i line?*

Response:

We thank the reviewer for this thoughtful comment. In response, we have conducted additional experiments using i-line (365 nm) irradiation as an alternative to 310 nm UV irradiation. As shown in Figure R2, the i-line exposure also enables direct photo-patterning of QDs, confirming the feasibility of this approach. However, the photosensitivity of the developed photo-reversible ligand (PRL) at 365 nm is substantially lower (Figure 2b in the original/revised manuscript), requiring a much higher exposure dose of approximately ~2 J/cm^2. We have included the i-line photolithography data in the revised manuscript to further enhance the practical applicability of our strategy.

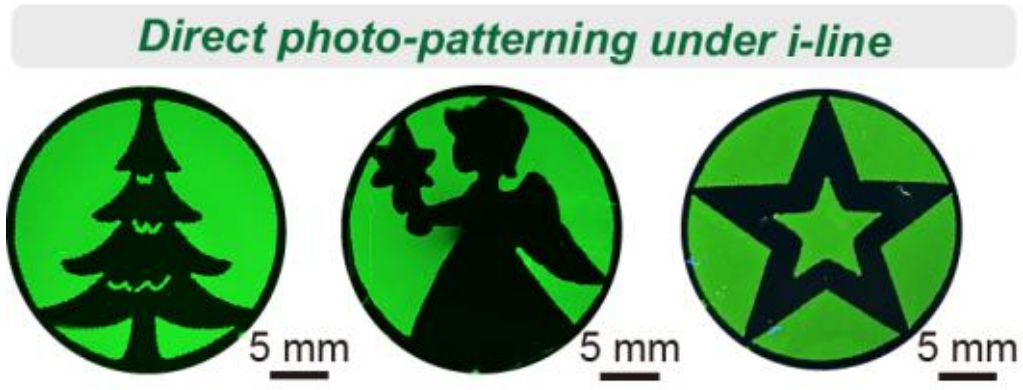

Figure R2. Fluorescent images of photolithographic PRL-GQD patterns under i-line. The required exposure dose is ~2 J/cm^2.

Change made on the second paragraph of Page 13: "In addition, although the photosensitivity of RPL is relatively weak in the commercial i-line region (365 nm), it remains capable of direct photo-patterning (Supplementary Fig. 20), which further enhances the practical applicability of our strategy."

Change made on Page 11 in the revised Supplementary Information:

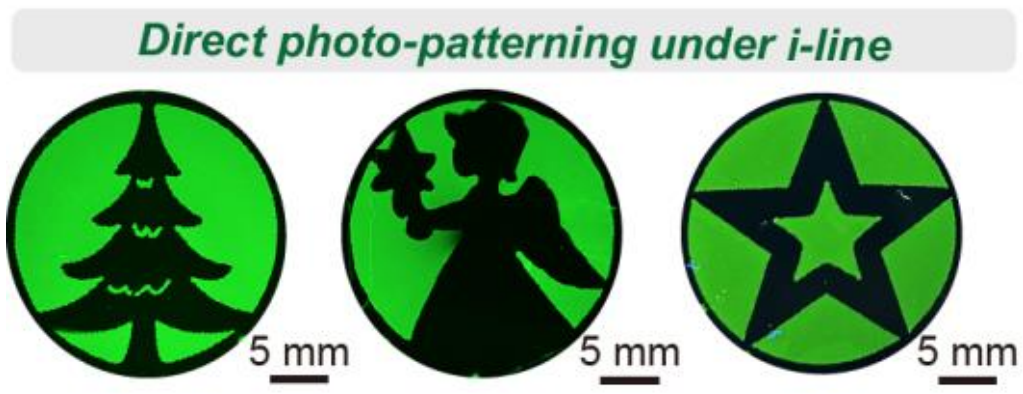

Supplementary Fig. 19 Fluorescent images of photolithographic PRL-GQD patterns under i-line. The required exposure dose is ~2 J/cm^2.

Comment: *5. In Figure 5, how do the patterned features compare with those obtained by conventional direct photolithography? Does the decrosslinking step introduce any positive or negative effects on pattern quality? Expanding the discussion along these lines would better highlight the central role of the reversible strategy.*

Response:

Dear reviewer, thanks for your insightful and constructive suggestions. We would like to clarify that all direct photo-patterning results presented in Figures 5a-g and the associated Supplementary Materials were obtained through

the complete reversible photolithography workflow under ambient conditions. Specifically, after achieving direct photo-crosslinking patterning of QDs using 310 nm LED UV irradiation and photomasks, the QD patterns were subsequently exposed to 254 nm UV irradiation to induce decrosslinking. This procedural detail has now been explicitly indicated in the revised manuscript.

Change made on the legend of Page 14: "All results in (a-g) were obtained under ambient conditions and following the complete reversible photolithography workflow, including the decrosslinking step."

The reversible photolithography strategy achieves an attractive pixel density of 21,000 PPI, which is comparable to or even better than that of conventional direct photolithography approaches. We attribute this to two main factors: (1) the thiol-anchoring group of the photo-reversible ligand binds strongly to the QD surface, which has been demonstrated in Figure 3d, resulting in minimal chemical diffusion; (2) the photo-crosslinking reaction is highly site-selective and efficient (with short exposure time and low dose), offering great potential for high resolution. On the other hand, the decrosslinking process has negligible effect on the morphology of the QD films, as evidenced by the surface roughness values before and after treatment (1.02 nm vs. 1.01 nm, Figure 3e).

Furthermore, your suggestion prompted us to explore the potential of our reversible photolithography strategy for enhancing the processability of QDs. Specifically, **by applying a secondary trimming step to the crosslinked QD patterns through decrosslinking process, a reconfigurable photo-patterning could be achieved**. As a proof of concept, we first fabricated 20 × 50 µm QD pixels *via* photo-crosslinking, which faithfully replicated the photomask pattern (Figure R3). Subsequently, we rotated the photomask and performed 254 nm exposure and development, successfully obtaining trimmed QD pixels. Notably, the dimensions of the trimmed pixels (e.g., 15 × 20 µm) are smaller than the original ones, demonstrating the significant reconfigurable potential of our reversible crosslinking strategy for achieving high-resolution QD patterns. And we will systematically investigate and discuss this aspect in detail in future work.

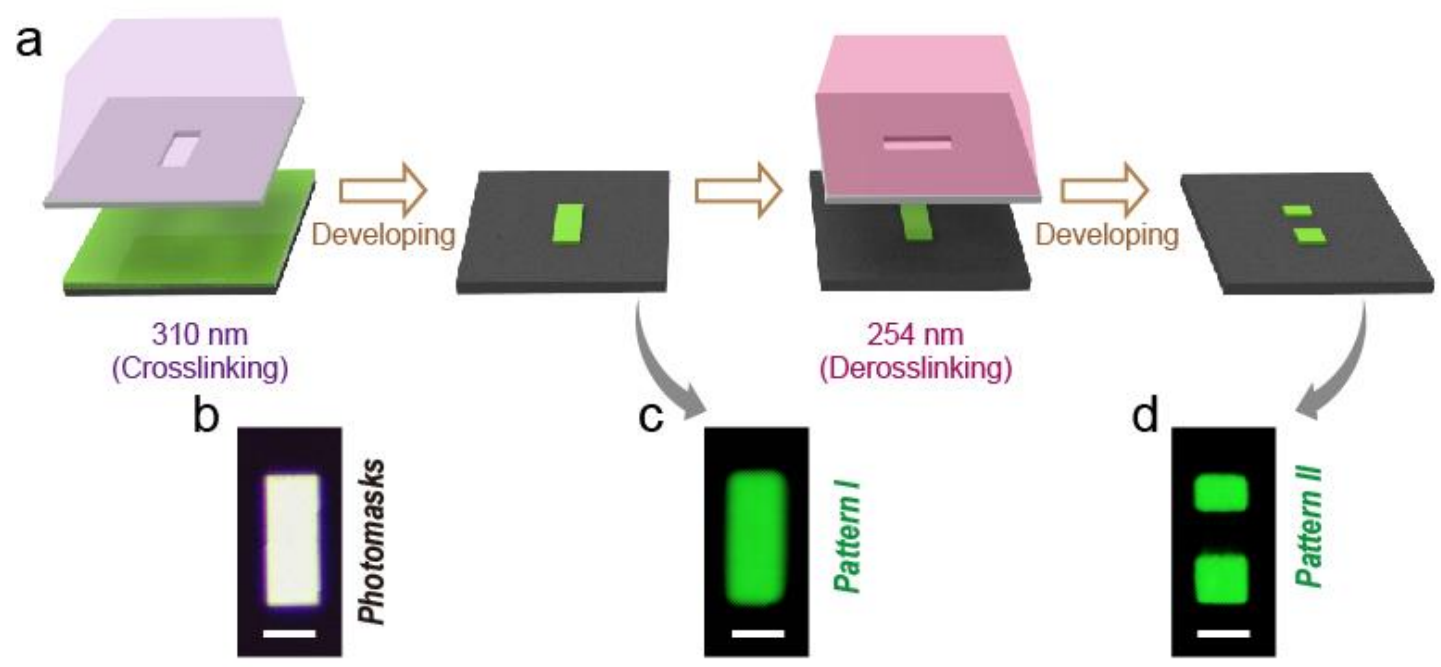

Figure R3. (a) Schematic of reconfigurable photo-patterning based on reversible photolithography strategy. (b-d) Microscopic image of the photomask (b) and corresponding fluorescent microscopic images of QDs before (c) and after (d) spatially localized decrosslinking. Scale bar: 20 μm.

Once again, we greatly appreciate your valuable suggestions, which have further strengthened the quality of our paper. We have incorporated the relevant discussions into the revised manuscript.

Change made on the first paragraph of Page 15: "We attribute the attractive high resolution to two main factors: (i) the thiol-anchoring group of RPL binds strongly to the QD surface (demonstrated in **Figure 3d**), resulting in minimal chemical diffusion; (ii) the photo-crosslinking reaction is highly site-selective and efficient (with short exposure time and low dose)."

Change made on the last paragraph of Page 13: "Moreover, the reversible photolithography strategy also enables reconfiguration of QD patterns. As a proof of concept, we first fabricated 20 × 50 μm QD pixels via photo-crosslinking (Supplementary Fig. 21). The photomask was then repositioned to allow spatially localized decrosslinking, yielding trimmed QD pixels with reduced dimensions (e.g., 15 × 20 μm)."

Change made on Page 11 in the revised Supplementary Information:

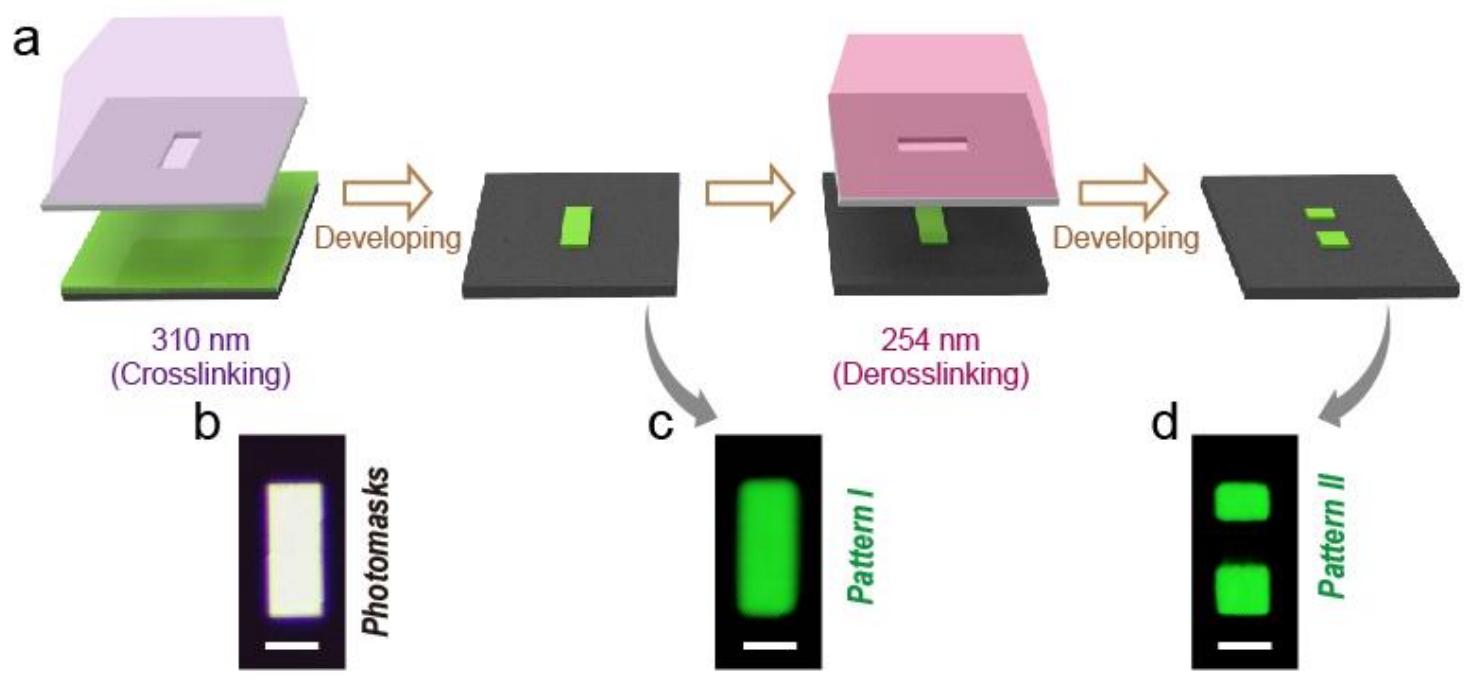

Supplementary Fig. 21 (a) Schematic of reconfigurable photo-patterning based on reversible photolithography strategy. (b-d) Microscopic image of the photomask (b) and corresponding fluorescent microscopic images of QDs before (c) and after (d) spatially localized decrosslinking. Scale bar: 20 μm.

Comment: *6. There are a few minor formatting issues: please unify the capitalization of "PL intensity" across the subfigures in Figure 3, and check the reference format of Ref. 40.*

Response:

Dear reviewer, we thank you for pointing out the formatting issues in our manuscript. We have systematically reviewed the entire manuscript and corrected the relevant issues accordingly.

To reviewer 2

Comment: *This work introduces an approach of crosslinking and de-crosslinking of QDs, PRL-based patterning, to demonstrate high-resolution QD-based displays. This approach appears new and the presented EL performance at the initial stage appears good. However, it should be noted that the focus of this work is about the photopatterning of QDs, not the EL performance because the high luminance and the high EQE are not simply related to the PRL-based patterning but the quality of QD and the other device fabrication process can be even more important factors. Overall, this manuscript does not contain any advance of the realization of practical QLEDs, compared to the recent well-performing, well-patterned QLED reports.*

Response:

Dear reviewer, we sincerely thank you for your time and efforts in reviewing our manuscript. We also greatly appreciate your positive recognition of our work, for example, your comment that "*this approach appears new and the presented EL performance at the initial stage appears good.*" At the same time, we have taken careful note of your concerns regarding the "advance" of our work. In response, **we have now thoroughly revised the manuscript, systematically supplemented the experiments, and analyzed the corresponding results**. We believe that the revised version will clarify your concerns and further highlight the significance of our work.

First, we fully understand your concern that *"the central focus of this work should be on the photopatterning of QDs rather than on electroluminescence performance*". As we all know, most reported devices based on photolithographically patterned QDs still exhibit EQEs below 20% [Ref: Chem. Rev. 2026, 126, 3624], although peak external quantum efficiency (EQE) values exceeding 20% are commonly achieved in mainstream QD platforms [Ref: Chem. Rev. 2023, 123, 4663]. In this case, we certainly agree that the quality of the QDs and the device fabrication processes play a critical role in this outcome, as you have rightly emphasized. Nevertheless, **it is undeniable that the patterning photochemistry involved in direct QD photolithography also exert a substantial influence on device performance**, as systematically summarized in a recent work [Ref: J. Am. Chem. Soc. 2024, 146, 28895]. For instance, ligand decomposition typically generates compact ionic ligands. Unlike long-chain organic ligands, these ionic species are less effective at passivating surface traps, which can promote nonradiative recombination [Ref: Chem. Rev. 2026, 126, 3624]. Ligand exchange processes mediated by photoacid generators often suffer from acid-induced degradation and the formation of surface defects [Ref: Adv. Mater. 2020, 32, 2003805].

Ligand crosslinking approaches lead to highly crosslinked ligand networks, which tend to increase electrical resistance [Ref: ACS Nano 2025, 19, 22253; Small Methods 2023, 7, 2300206].

The abovementioned negative impact of photochemical reactions on device performance is precisely the core issue addressed in our work. We would like to emphasize that, for this work, **achieving high-performance electroluminescence is the target, while the developed reversible photolithography is the strategy**. Specifically, through a carefully designed photo-induced decrosslinking process, we overcome the performance degradation typically induced by the altered QD structure and functionality (e.g., charge transport conductivity and exciton binding capability) during photo-crosslinking photolithography, thereby providing an important pathway toward high-performance QLED devices. Thanks again for your comment. For better understanding, we have now revised the relevant discussion.

Change made on the second paragraph of Page 2: "Unfortunately, while peak external quantum efficiencies (EQEs) exceeding 20% are commonly achieved in mainstream QD platforms,[14] most photolithography-based devices still fall below this threshold.[29]"

Change made on the third paragraph of Page 2: "The primary cause lies in the inherent surface ligand changes of QDs during photolithographic processing (e.g., ligand decomposition, exchange, or crosslinking, along with the resulting harmful photochemical products),[22] which can readily induce adverse effects.[26-28] For instance, compact ionic ligands generated from ligand decomposition are less effective at passivating surface traps.[29] Ligand exchange processes mediated by photoacid generators often suffer from acid-induced degradation and the formation of surface defects.[19] Highly crosslinked ligand networks tend to increase electrical resistance.[24,35,37]"

Regarding your concern about how our work compares with the recent well-patterned QLED reports, **we have fabricated an ultrahigh-resolution nano-pixelated QLED, achieving a highly competitive peak EQE of 17.08% at 21,000 PPI**. Full details are provided in our response to your following comments.

We sincerely thank you again for your time and careful evaluation of our manuscript. We believe that, with the constructive comments from you and the other reviewers, the quality of our work has been significantly improved.

Comment: *A list of more detailed serious issues:*

1) The rationale for the crosslinking and de-crosslinking of QD films is not clear from the perspective of the practical fabrication of QD-based displays. The process involving 254 nm UV irradiation is not compatible with industrial

fabrication processes. Furthermore, several previous studies have reported no degradation, or even improvements, in QD properties after photo-crosslinking. In contrast, in this work, photo-crosslinking degrades the QDs, and their pristine properties are not-fully restored after de-crosslinking. Moreover, the EL performance of the de-crosslinked devices remains inferior to that of the pristine devices and exhibits a shorter operational lifetime. Therefore, it is difficult to identify any clear advantages of this crosslinking/de-crosslinking approach over previously reported direct photopatterning methods.

Response:

Dear reviewer, we will address your concern regarding "advantages of this crosslinking/de-crosslinking approach over previously reported direct photopatterning methods" from the following aspects.

(1) Regarding the use of 254 nm UV irradiation

First, **the 254 nm wavelength is a classic choice for laboratory-level proof-of-concept studies**, as the associated light sources are structurally simple and cost-effective. Indeed, this wavelength has been widely adopted in recent QD photolithography reports [Ref: Nat. Commun. 2025, 16, 9689; Mater. Today 2025, 89, 100; Light Sci. Appl. 2025, 14, 251; Sci. Adv. 2022, 8, eabm8433; Adv. Mater. 2022, 34, 2205504; Nat. Commun. 2020, 11, 2874]. Second, **the 254 nm wavelength can be readily extended to the commercially more prevalent 248 nm KrF lithography systems**, which are widely used and well-established in the semiconductor industry.

Therefore, we consider the choice of 254 nm to be justified for validating the feasibility of the reversible photolithography strategy. We thank the reviewer again for raising this valuable comment. In the revised manuscript, we have supplemented a discussion to further clarify the rationale behind our wavelength selection.

Change made on the second paragraph of Page 5: "Here, deep-UV (DUV) LED lamp (centered at 254 nm) is employed, owing to its structural simplicity and cost-effectiveness, as well as its ready extendability to the commercially more prevalent 248 nm KrF lithography systems."

(2) Regarding the performance results presented in this work

To date, various strategies have been explored to minimize photolithography-induced damage, including photosensitive wavelength regulation [Ref: Nat. Nanotechnol. 2022, 17, 952], functional unit/skeleton engineering [Ref: Light Sci. Appl. 2025, 14, 251; Adv. Mater. 2022, 34, 2205504], and compatibility optimization [Ref: Mater. Today 2025, 89, 100]. In addition, post-photolithographic treatments such as film-state ligand exchange and

passivation have been employed to maintain, recover, or even positively enhance device performance [Ref: Small 2026, 22, e00011; Adv. Mater. 2025, 37, e08217; ACS Energy Lett. 2023, 8, 4210]. These efforts underscore that **achieving low-damage or nondestructive direct photolithography of QDs has long been a central goal in the field**. We do not intend to overlook or dismiss these advances; indeed, **these works have been properly cited in the Introduction of our original manuscript**.

Nevertheless, it remains regrettable that most reported approaches still suffer from performance degradation after photolithography, a phenomenon that can be rationalized from the perspective of the inevitable surface ligand changes induced by photochemical processes, as systematically summarized in a recent work [Ref: J. Am. Chem. Soc. 2024, 146, 28895]. To address this issue, **this present work introduces a novel reversible photolithography strategy, which bypasses the adverse effects of photochemical reactions on the QD surface ligand states**. Based on this strategy, we successfully demonstrate attractive results, such as **a luminance of 125,016 cd m^{-2} at 5.0 V and a peak external quantum efficiency of 24.41%**, represent one of the best performances in the field (as shown in Figure 4i of the original/revised manuscript). These advances clearly demonstrate the significance of our progress, which have also received consistent positive recognition from the other three reviewers.

Furthermore, in the revised manuscript, **we have fabricated an ultrahigh-resolution nano-pixelated QLED, achieving a highly competitive peak EQE of 17.08% at 21,000 PPI**. This result further underscores the effectiveness of our reversible photolithography strategy in simultaneously achieving high-resolution patterning and high device performance. Full details are provided in our response to your following comments.

(3) Regarding performance retention before and after reversible photolithography

In our original manuscript, we used QLEDs fabricated from PRL-tailored green-emitting QDs (termed PRL-GQD) as the "pristine" group. This choice was intended to provide a clear basis for tracking performance changes throughout the photo-crosslinking/decrosslinking process, thereby highlighting the effectiveness of our strategy in overcoming conventional performance degradation.

We acknowledge that this choice was not entirely suitable, as the incorporation of the photosensitive ligand is itself an integral part of the reversible photolithography strategy, as suggested by *Reviewer 4*. **To provide a more meaningful baseline, we have now fabricated QLED devices based on conventional pristine QDs** (oleic acid-capped QDs in this work, termed OA-GQD) using the same functional materials and fabrication processes, and evaluated their electroluminescence performance in the revised manuscript. As shown in Figure R4, the device

exhibits a current density of 87.5 mA cm^{-2} and a luminance of 93,801 cd m^{-2} at 5.0 V, with a peak EQE of only 23.53%. These values are significantly lower than those of the devices after reversible photolithography (e.g., 114.7 mA/cm^2 and 125,016 cd m^{-2} at 5.0 V, a peak EQE of 24.41%). We attribute this improvement after reversible photolithography to the efficient passivation effect of photosensitive ligand (PRL), which has been comprehensively demonstrated in our original manuscript. In a word, by using OA-GQD as a standard reference, we demonstrate that **our reversible photolithography strategy not only enables effective photo-patterning but also yields positive enhancement in the electroluminescence performance**—an encouraging outcome that is fully competitive with the most advanced results currently reported in the field.

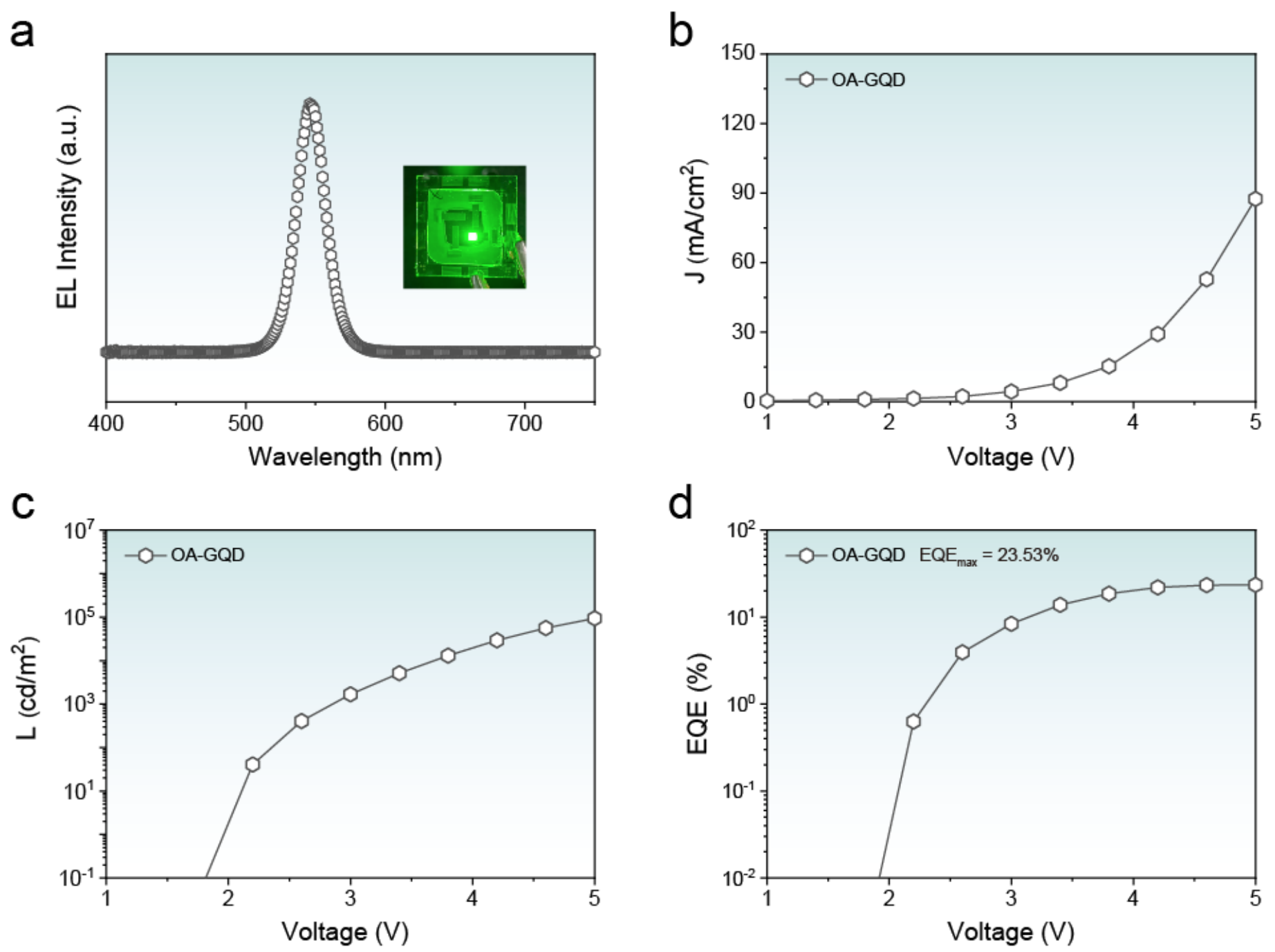

Figure R4. Electroluminescence performance of the QLED device based on OA-GQD. (a-d) Normalized EL spectra (a), *J*–*V* curves (b), *L*–*V* curves (c) and EQE–*V* curves (d) of QLEDs based on OA-GQD. Insert in (a): EL photo of corresponding QLED at 5.0 V.

Change made on the last paragraph of Page 11: "In addition, we also fabricated QLEDs based on pristine OA-GQD and characterized their EL performance. As shown in Supplementary Fig. 15, these devices exhibited a current density of 87.5 mA cm^{-2} and a luminance of 93,801 cd m^{-2} at 5.0 V, with a peak EQE of only 23.53%. These values are markedly lower than those of the decrosslinked PRL-GQD devices."

Change made on Page 9 in the revised Supplementary Information:

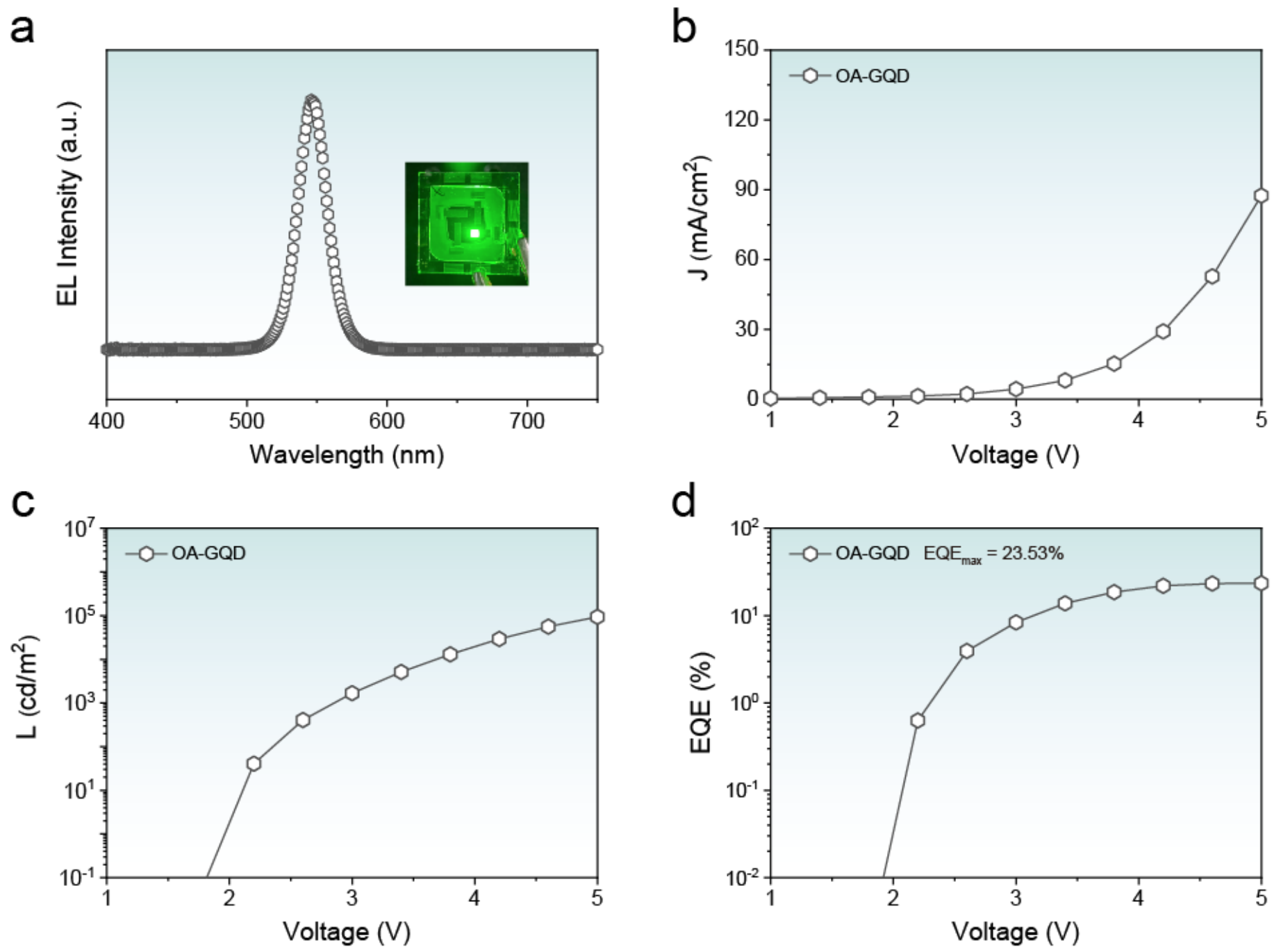

Supplementary Fig. 15 EL performance of the QLED device based on OA-GQD. (a-d) Normalized EL spectra (a), *J–V* curves (b), *L–V* curves (c) and EQE–*V* curves (d) of the QLED. Insert in (a): EL photo of corresponding QLED at 5.0 V.

On the other hand, it should be acknowledged that, when limited to the crosslinking and decrosslinking processes alone, complete recovery (~100%) of EL performance has not yet been realized. This is primarily **due to the fact that the photocleavage (decrosslinking) and photodimerization (crosslinking) reactions of coumarin derivatives are in a dynamic equilibrium under 254 nm light irradiation**. This phenomenon has been documented in previous studies [Ref: Adv. Mater. 2021, 33, 2008057; Polymer 2012, 53, 2691; ACS Appl. Mater. Interfaces 2018, 10, 16793]. And we look forward to further optimizing the molecular structure and QD photosensitive material system in future work to achieve more complete performance recovery.

Despite this, we want like to emphasize that **the core contribution of this work lies in the conceptual innovation of a reversible photolithography strategy to address the performance degradation inherent in conventional direct QD photolithography**. The coumarin-based photosensitive ligand is employed here as a demonstrative route to validate the feasibility of the concept, rather than as the sole embodiment of the strategy. Importantly, this technical route has already yielded compelling results. We therefore believe that the novelty and technical significance of our work merit due recognition.

Change made on the second paragraph of Page 11: "A minor drawback is that the EL recovery is not 100%, due to the reversible photocleavage equilibrium of coumarin groups under 254 nm irradiation.[59] And we hope to overcome this by further molecular and system optimization in the future."

Change made on Page 20: "[59] Zhu, C. N. et al. Reconstructable gradient structures and reprogrammable 3D deformations of hydrogels with coumarin units as the photolabile crosslinks. *Adv. Mater.* **33**, 2008057 (2021)."

Comment: *2) De-crosslinking of QDs may lead to device instability because the initially optimized surface ligands could become mobile during device operation. Indeed, as shown in Figure 4h, the operational lifetime of the de-crosslinked device is shorter than that of the pristine device. Therefore, from the standpoint of device stability, the proposed approach does not demonstrate a clear advantage over previously reported high-performance QLEDs.*

Response:

Dear reviewer, we appreciate your concerns regarding material stability and device lifetime, both of which are indeed critical issues for the commercialization of QD-based devices. However, we would like to clarify that the reversible crosslinking strategy developed in this work does not introduce additional instability, and the lifetime results we obtained remain competitive within the field. Our reasoning is as follows:

(1) Impact of the decrosslinking process on QD stability

First, we respectfully offer a different perspective on your concern that "*de-crosslinking of QDs may lead to device instability because the initially optimized surface ligands could become mobile during device operation.*" We note that in most mainstream QLED systems, the QD surface ligands—such as oleylamine, oleic acid, or thiol-based ligands—are typically in an un-crosslinked state [Ref: Nat. Nanotechnol. 2023, 18, 1168; Nat. Photon. 2022, 16, 505]. In these well-established systems, potential ligand mobility is common.

In our work, the as-developed photo-reversible ligand (PRL) contains a thiol anchoring group, which binds strongly to the QD surface with a binding energy significantly higher than that of the native oleic acid (OA) ligand, as confirmed by both theoretical calculations and XPS measurements (Figure 2d and Supplementary Fig. 10). Therefore, the ligand (still the PRL) does not become more mobile compared with native OA. Accordingly, we do not anticipate that the decrosslinked ligands would introduce additional instability or lifetime issues in our system.

(2) Operational lifetime of QLED devices before and after reversible photolithography

We sincerely thank you for your careful observation and detailed analysis of our data. We acknowledge that a slight lifetime reduction does remain after decrosslinking, as you have rightly pointed out. We attribute this primarily to **the dynamic equilibrium between the photocleavage (decrosslinking) and photodimerization (crosslinking) reactions of coumarin derivatives under 254 nm irradiation** [Ref: Adv. Mater. 2021, 33, 2008057], which inherently limits complete recovery. This bottleneck and potential strategies to address it have been discussed in our response to your previous comment.

It is worth noting that **photolithography-induced lifetime degradation remains a common issue, even in recent high-quality reports**, as reflected by the limited retention rates of 85%-90% [Ref: Nat. Commun. 2025, 16, 9689] and 88% [Ref: Mater. Today 2025, 89, 100]. In our work, the photo-crosslinking process leads to a decrease in operational lifetime (T_{95}@1000 cd m^{-2} from 1041 h to 573 h). After decrosslinking, the lifetime recovers substantially to 942 h, corresponding to a 90% retention rate—a value that is comparable to reported values.

Finally, we wish to reiterate that **the coumarin-based photosensitive ligand serves as a demonstrative route to validate the feasibility of the reversible photolithography concept, rather than as the sole embodiment of the strategy**. The core contribution of this work lies in the conceptual innovation of a reversible photolithography strategy to overcome the performance degradation during direct QD photolithography. We are grateful for your thorough review, and we have added a discussion addressing the lifetime in the revised manuscript.

Change made on the second paragraph of Page 11: "A minor drawback is that the EL recovery is not 100%, due to the reversible photocleavage equilibrium of coumarin groups under 254 nm irradiation.[59] And we hope to overcome this by further molecular and system optimization in the future."

Change made on Page 20: "[59] Zhu, C. N. et al. Reconstructable gradient structures and reprogrammable 3D deformations of hydrogels with coumarin units as the photolabile crosslinks. *Adv. Mater.* **33**, 2008057 (2021)."

(3) Operational lifetime comparison of photolithographic QLEDs

We fully agree with the reviewer on the importance of operational lifetime. Unfortunately, **studies on the operational lifetime of photolithographic QDs remain relatively scarce, with most reports focusing on red-emitting QD systems**. Moreover, the reported lifetime values in this subfield are still at a relatively early stage and fall considerably short of those achieved by state-of-the-art QLEDs (e.g., 7200 h for green-emitting [Ref: Nat.

Photon. 2022, 16, 505–511], 48,000 h for red-emitting [Ref: Nat. Nanotechnol. 2023, 18, 1168]). Meanwhile, it is well recognized that device lifetime is highly dependent on the intrinsic properties of the QD core/shell structure, the device architecture, charge transport layers, and fabrication conditions. The photochemical process involved in direct photolithography is one of many contributing factors, and in some high-quality reports in the field, this factor often plays a negative role [Ref: Nat. Commun. 2025, 16, 9689; Mater. Today 2025, 89, 100].

Given the substantial variations in QD platforms, device structures, and lifetime testing protocols across different studies, direct comparison of absolute lifetime values is of limited significance. Nevertheless, to provide an objective assessment of our results, we have summarized "the lifetime parameters and testing conditions of reported photolithographic QD (and perovskite) devices**"** in Supplementary Table 1 in the revised Supplementary Information. Within this comparative framework, our photolithographic QLED devices exhibit a T_{95} lifetime of 942 h at an initial luminance of 1000 cd m^{-2}, which represents a competitive level. We anticipate that further optimization of QD materials and device fabrication parameters will enable continued improvements in lifetime and bring us closer to practical applications.

We sincerely thank you once again for your careful review and insightful comments on our manuscript.

Comment: *3) The authors often claimed "high fidelity and high resolution" in the manuscript. The simply high number of PPI is not important. Rather, the demonstration of the excellent electrical performance with the practically approachable PPIs in the LED device is even more important. Based on Figure 5c, pattern fidelity is very poor. And, the authors have not shown any side views of the pixels in Figure 5f. The simple top image does not guarantee the quality of pixel features are useful for the generation of the real displays. There are several published papers regarding "how to define pattern fidelity". Please analyze the fidelity of patterns based on such standized or well-known methods. Note that the surface roughness of the QD films is one of the simplest fidelity factor, too short of analyses.*

Response:

Dear reviewer, thanks for your comment about the fidelity and resolution of patterns.

First, regarding the concern about the poor pattern fidelity observed in Figure 5c of the original manuscript, we would like to clarify that those patterns were prepared without systematic optimization of the photolithographic

parameters. To further address your concern, **we have provided a detailed AFM image and corresponding fidelity analysis of photolithographic QD patterns** with a line width of ~12 μm. AFM characterization revealed that it exhibits a relatively flat-top surface and sharp edges (Figure R5a). Then, we also performed a quantitative fidelity analysis on the fabricated QD patterns, evaluating three key metrics: line-edge roughness (LER), longitudinal line-width roughness (L-LWR), and vertical line-width roughness (V-LWR). All parameters were determined as three times the standard deviation (3σ) of the corresponding edge-position or line-width distributions, following established procedures [Ref: Nat. Commun. 2026, 17, 4162; Sci. Adv. 2023, 9, eadi6950]. As summarized in Figure R5b, the measured LER, L-LWR, and V-LWR values are only 0.29 ± 0.04 μm, 0.49 ± 0.05 μm, and 3.11 ± 0.32 μm, respectively. Corresponding data and discussion have added in the revised manuscript.

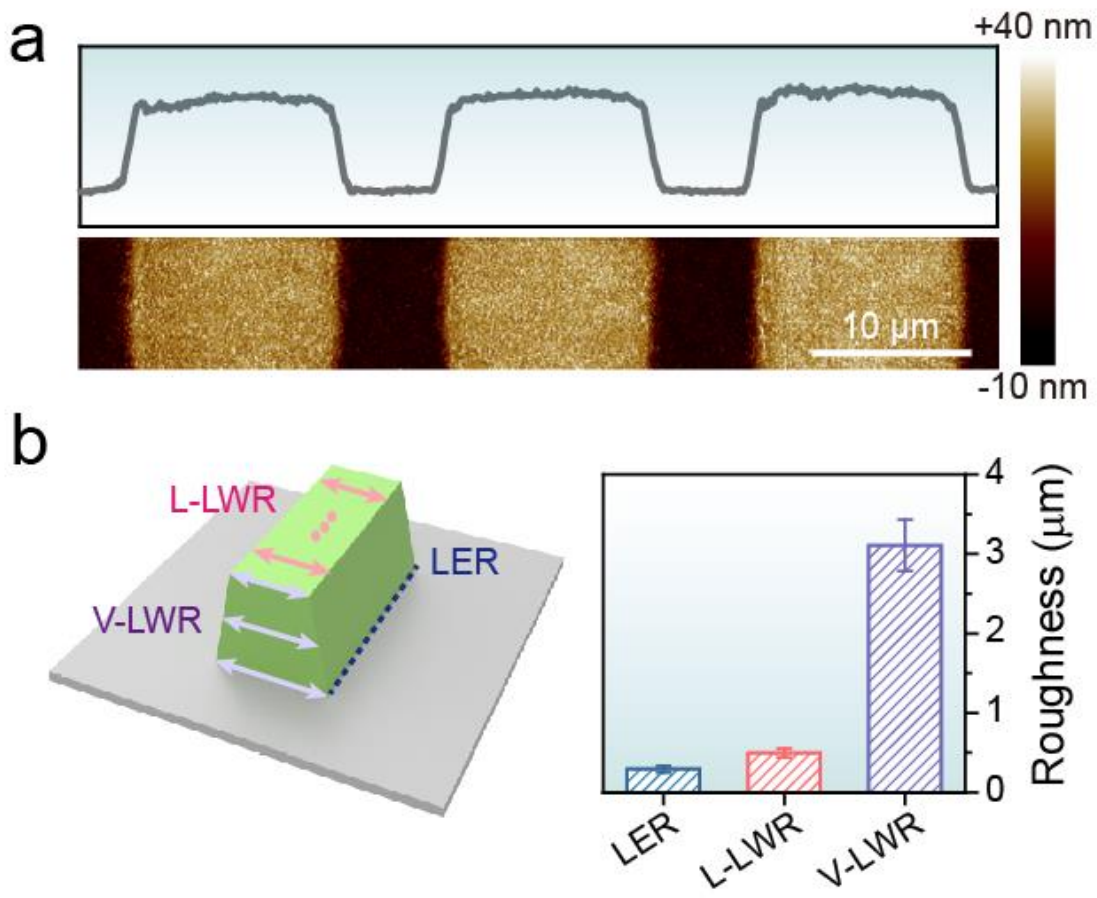

Figure R5. (a) AFM image and surface profile of photolithographic QD patterns. (b) Left: Schematic of and corresponding LER, V-LWR and L-LWR. Right: Averaged values of LER, L-LWR and V-LWR. The data are averaged from six samples and corresponding error bars represent the standard deviation of the data.

Change made on the first paragraph of Page 13: "Under this parameter, the resulting QD patterns with a 12-μm line width exhibit a relatively flat-top surface and sharp edges, as revealed by AFM characterization (**Figure 5b**). We further performed a quantitative fidelity analysis through three key metrics: line-edge roughness (LER), longitudinal line-width roughness (L-LWR), and vertical line-width roughness (V-LWR). All metrics were determined as three times the standard deviation (3σ) of the corresponding edge-position or line-width distributions, following established procedures.[30,57] As summarized in **Figure 5c**, the measured LER, L-LWR, and V-LWR values are 0.29 ± 0.04 μm, 0.49 ± 0.05 μm, and 3.11 ± 0.32 μm, respectively, demonstrating high-fidelity photolithographic outcomes."

Change made on Page 14:

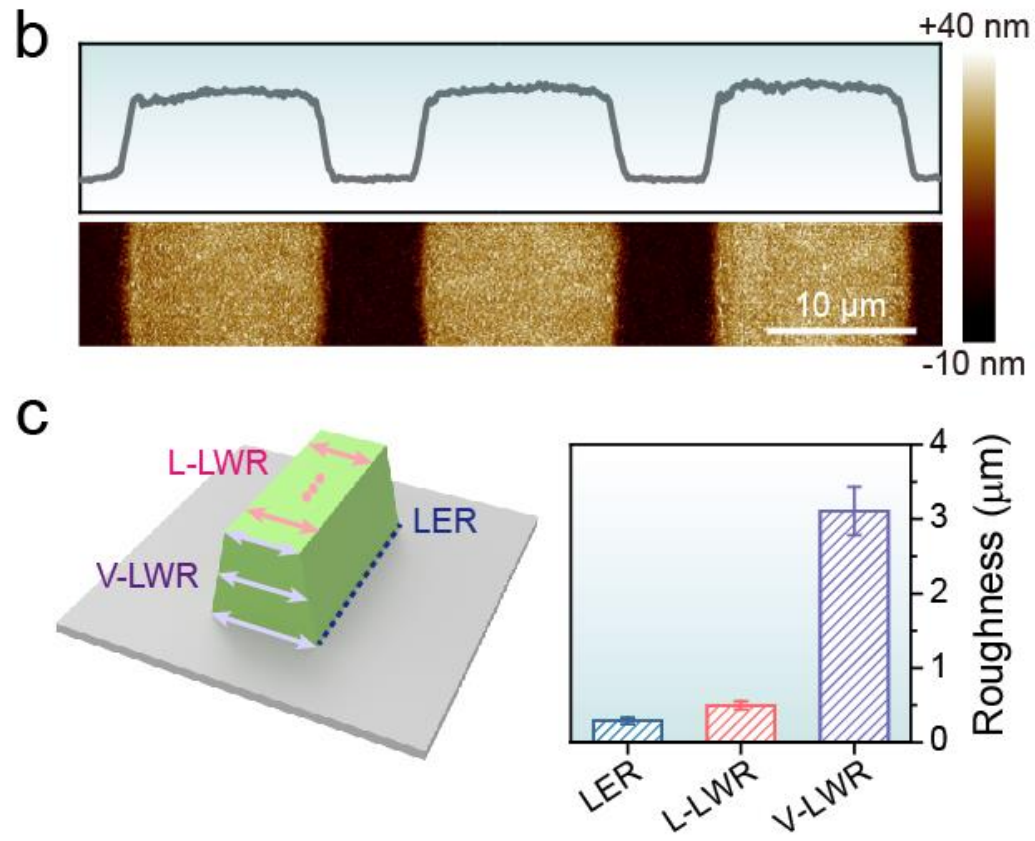

Figure 5. (b) AFM image and surface profile of photolithographic QD patterns. (c) Left: Schematic of fidelity analysis through LER, L-LWR and V-LWR. Right: Averaged values of LER, L-LWR and V-LWR. The data are averaged from six samples and corresponding error bars represent the standard deviation of the data.

Change made on Page 20: "[57] Kim, H. et al. Photoresist-guided indirect photopatterning of quantum dots via carbene-mediated ligand thermocrosslinking. *Nat. Commun.* **17**, 4162 (2026)."

On the other hand, we fully agree with your viewpoint that the practical potential should be demonstrated by high-resolution and high-performance QLED devices, rather than relying solely on high-resolution PL or EL images. In response, we also performed AFM measurements on the QD pixels at 21,000 PPI. As shown in Figure R6, **the pixels exhibit well-defined boundaries without mutual adhesion, ensuring high contrast for electroluminescent pixels.**

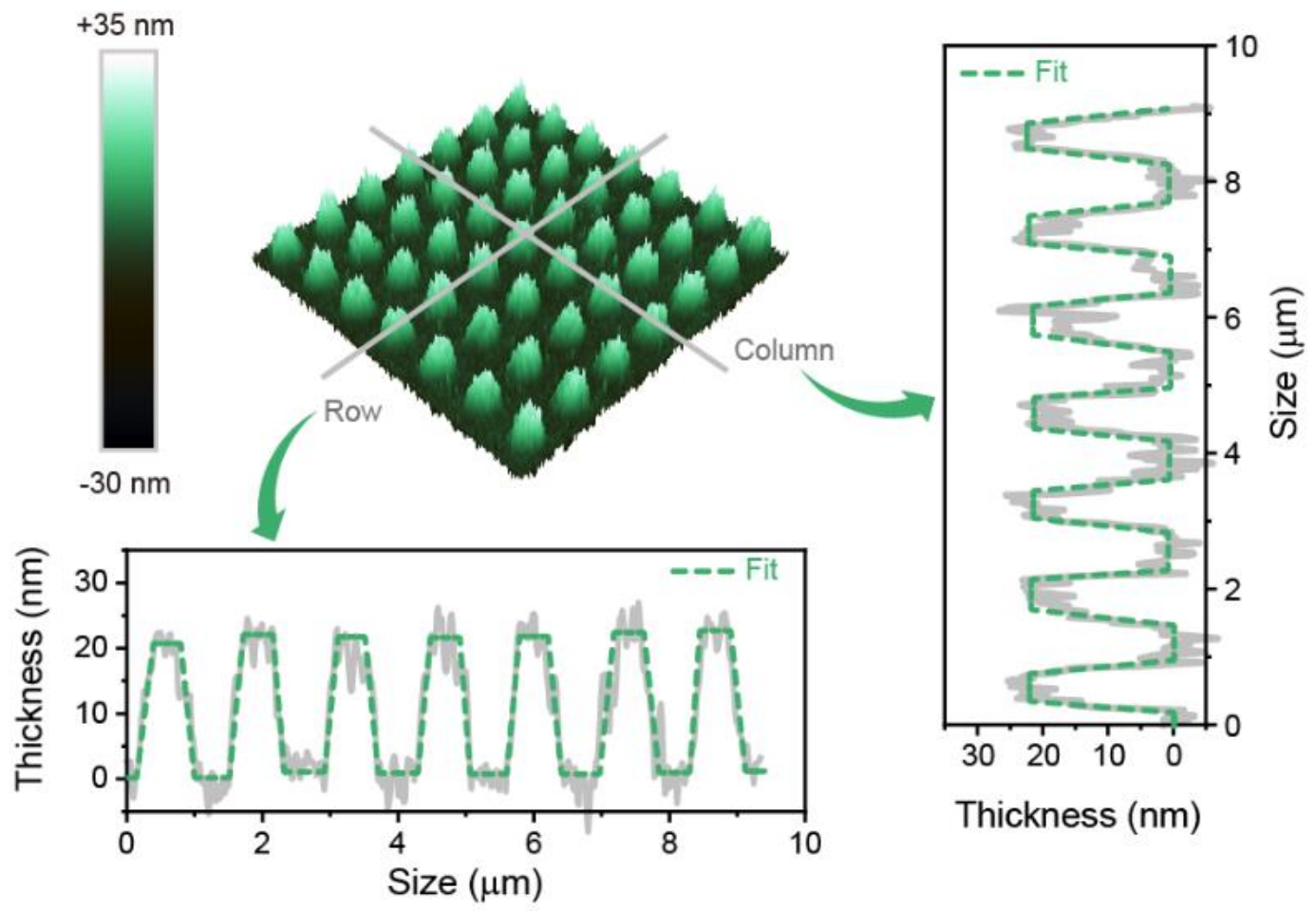

Figure R6. Three-dimensional AFM image and surface profile of QD pixels on silicon wafer at ~21,000 PPI.

Furthermore, **we have fabricated corresponding nano-pixelated QLED devices at such an ultrahigh resolution.** Here, a polymethyl methacrylate (PMMA) layer was introduced to suppress inter-pixel leakage current.

Gratifyingly, electroluminescence measurements revealed that the device after decrosslinking exhibited improved *L* (49,292 vs. 28,769 cd/m^2), *J* (65.6 vs. 40.7 mA/cm^2), and peak EQE (17.08% vs. 16.02%), as shown in Figures R7. It is consistent with the performance trends observed in non-patterned devices (Figure 4 in the original/revised manuscript). While the peak EQE of the nano-pixelated devices is slightly lower than that of the non-patterned ones, likely due to the non-ideal pixel morphology at such a high resolution and the electric-field non-uniformity from pixel microstructures [Ref: Nature 2026, 652, 349]. Nevertheless, **the peak EQE of 17.08% at 21,000 PPI still represents one of the highest reported values in the field** (Figure R8). These results further underscore the effectiveness of our reversible photolithography strategy in simultaneously achieving high-resolution patterning and high device performance.

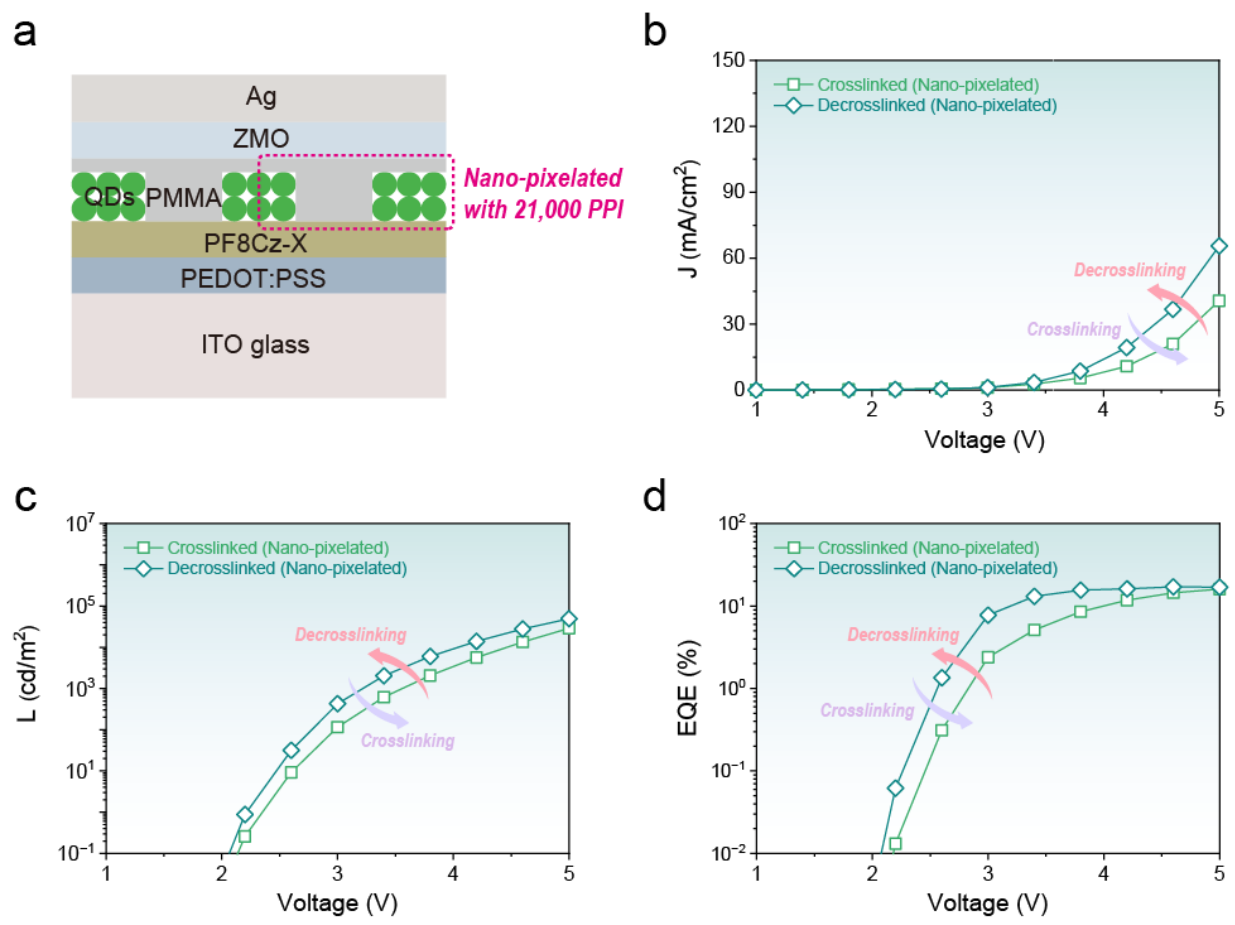

Figure R7. Electroluminescence performance of nano-pixelated QLED devices at 21,000 PPI. (a) Schematic of nano-pixelated QLED with a PMMA layer. (b-d) Normalized EL spectra (a), *J–V* curves (b), *L–V* curves (c) and EQE–*V* curves (d) of QLEDs based on OA-GQD. Insert in (a): EL photos of corresponding QLED at 5.0 V.

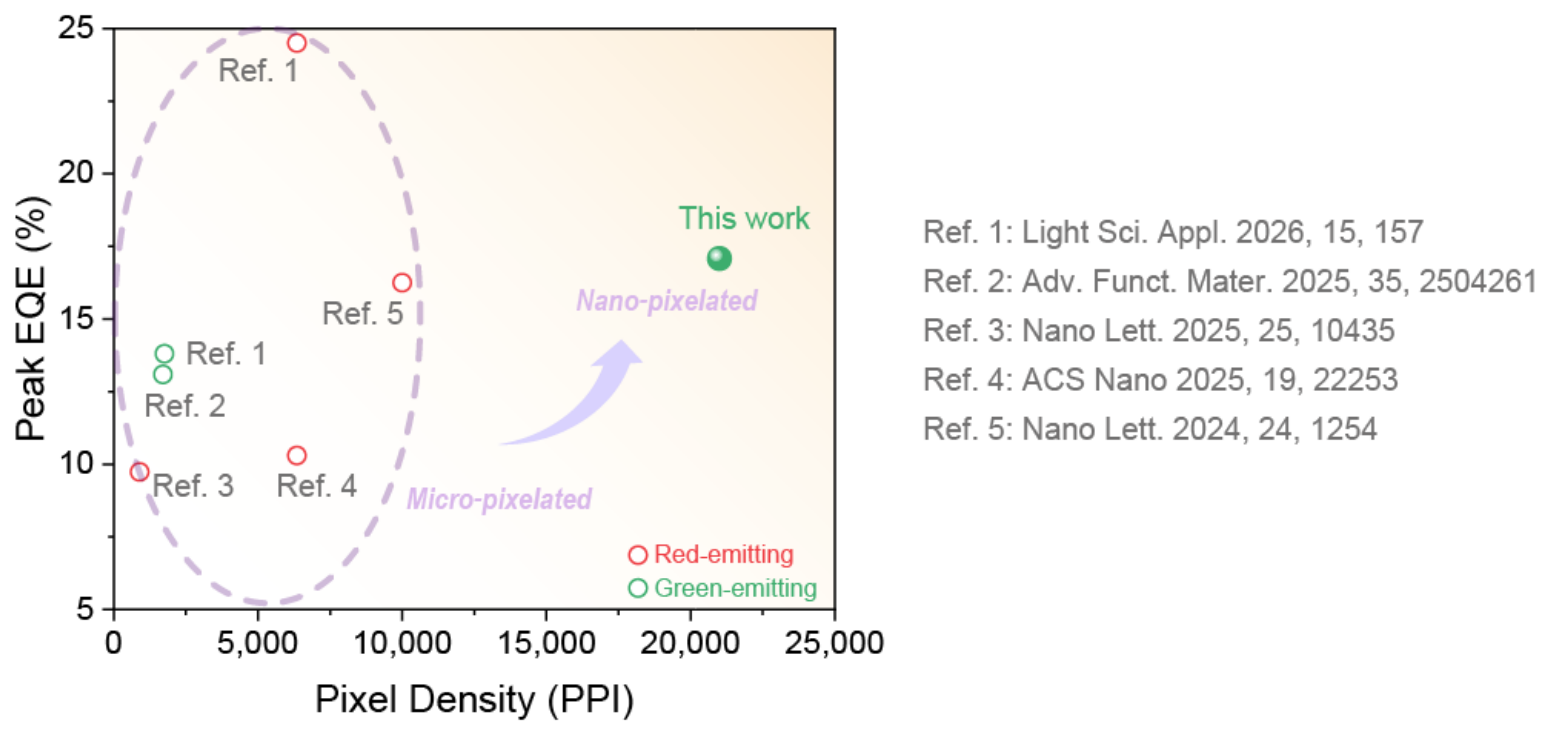

Figure R8. Comparation of different photolithographic QLEDs in pixel density and peak EQE.

Change made on the last paragraph of Page 15: "Here, we introduced a PMMA layer in as-fabricated ultrahigh-resolution nano-pixelated QLEDs with 21,000 PPI. EL measurements revealed that the device after decrosslinking

exhibited improved L (49,292 vs. 28,769 cd/m^2), J (65.6 vs. 40.7 mA/cm^2), and peak EQE (17.08% vs. 16.02%), as shown in **Figure 5i** and Supplementary Fig. 24, which is consistent with the performance trends observed in non-patterned devices. While the peak EQE of the nano-pixelated devices is slightly lower than that of the non-patterned ones, likely due to the non-ideal pixel morphology at such a high resolution and the electric-field non-uniformity from pixel microstructures,[1] this value (17.08% at 21,000 PPI) remains highly competitive (Supplementary Fig. 25 and Table 2). These results further underscore the effectiveness of our reversible photolithography strategy in simultaneously achieving high resolution and high device performance."

Change made on Page 14:

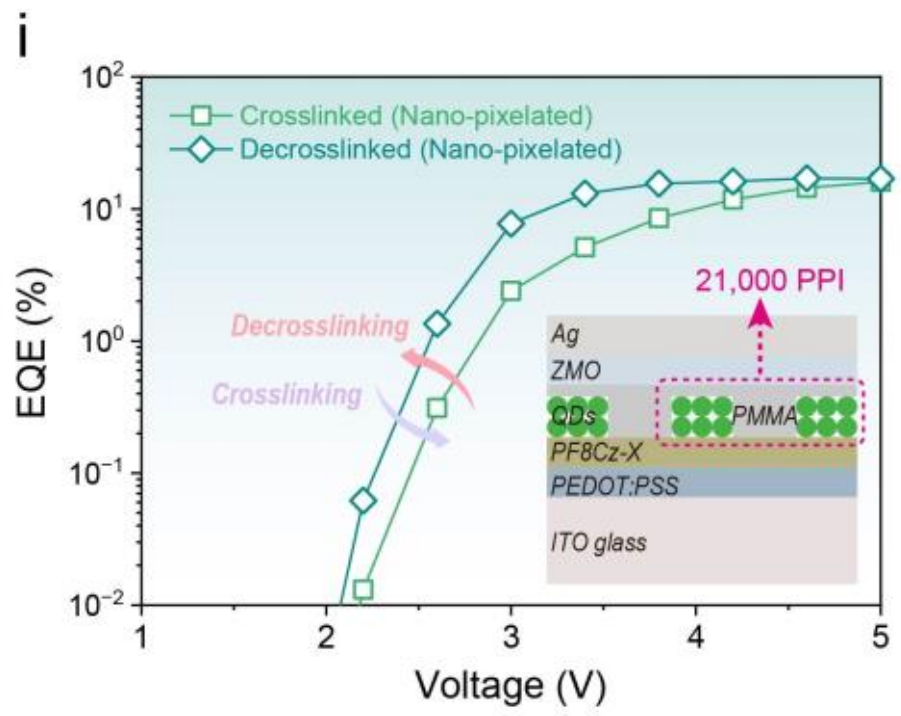

Figure 5 (i) EQE–V curves of nano-pixelated QLEDs based on crosslinked PRL-GQD and decrosslinked PRL-GQD. Insert: schematic of nano-pixelated QLED with a PMMA layer.

Change made on Page 25 in the revised Supplementary Information: "For pixelated QLEDs, a PMMA blocking layer (0.5 mg/mL, 5000 rpm) was spin-coated on the pixelated QDs to prevent current leakage caused by the direct contact between the hole transport layer and electron transport layer."

Change made on Page 12-13 in the revised Supplementary Information:

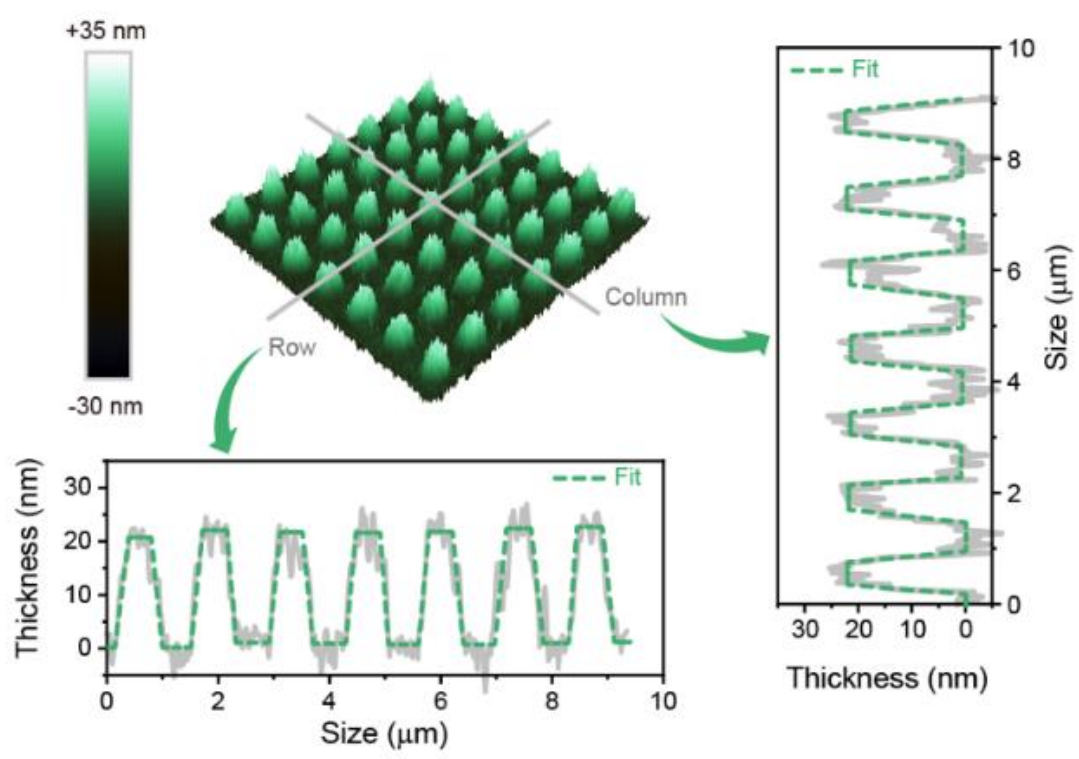

Supplementary Fig. 22 Three-dimensional AFM image and surface profile of QD pixels on silicon wafer at ~21,000 PPI.

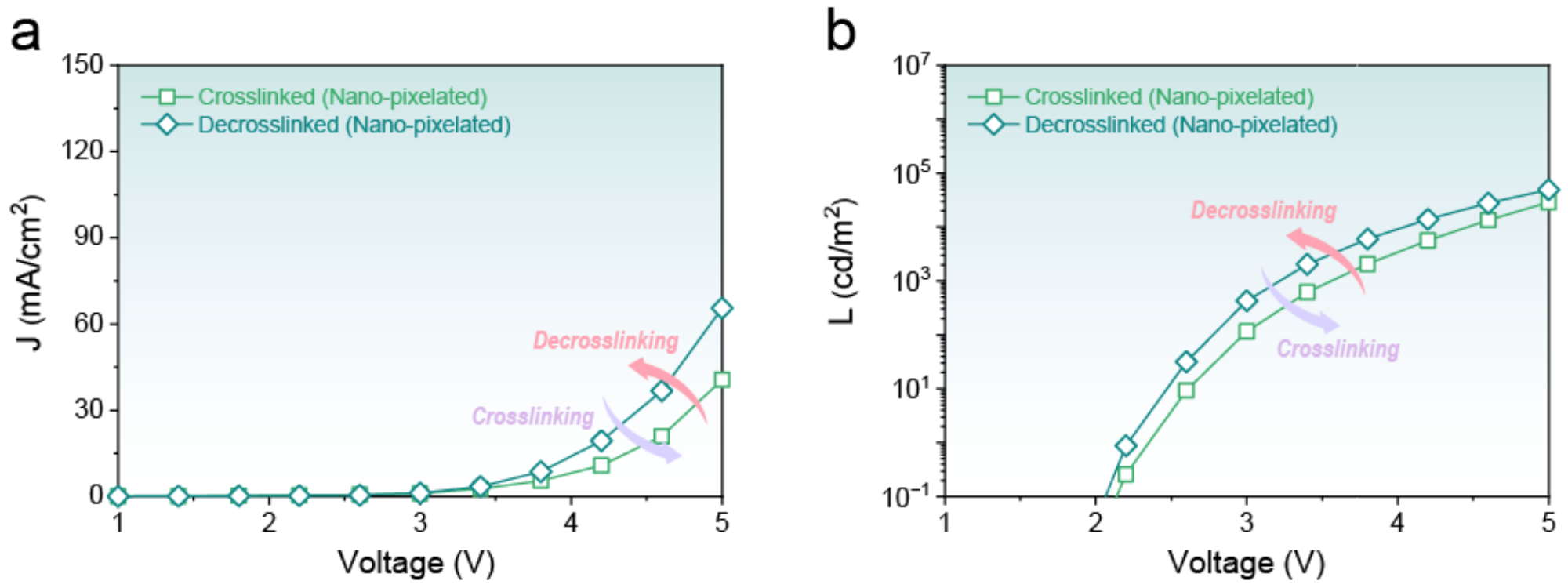

Supplementary Fig. 24 *J–V* curves (a) and *L–V* curves (b) of nano-pixelated QLEDs based on crosslinked PRL-GQD and decrosslinked PRL-GQD.

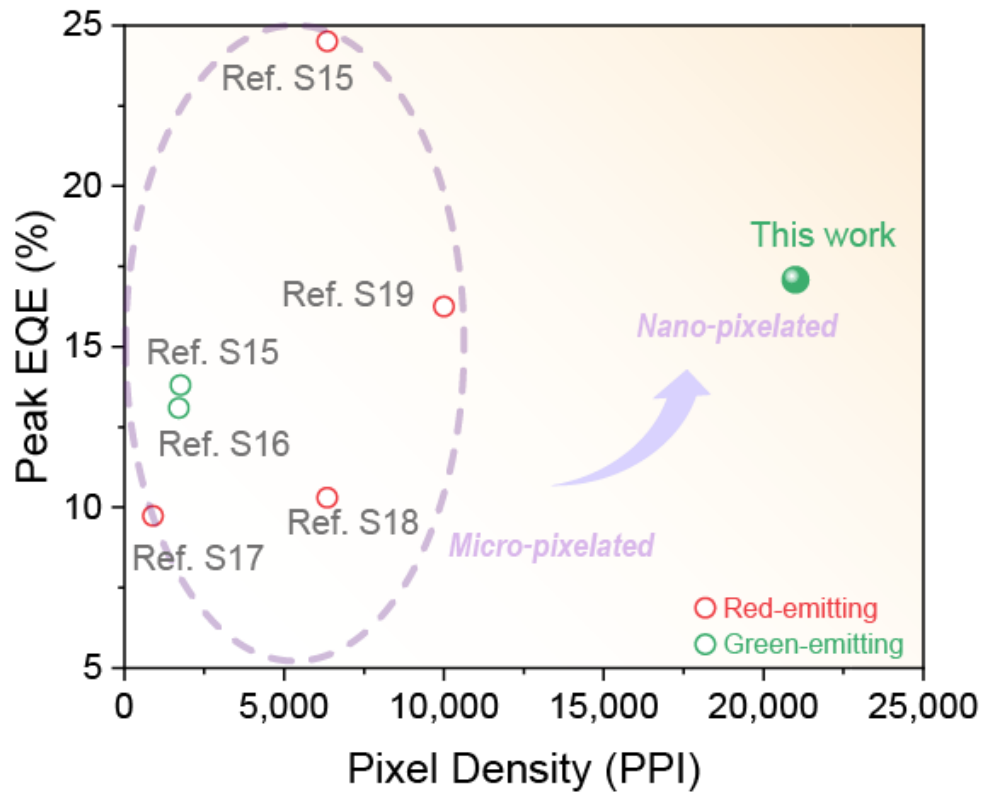

Supplementary Fig. 25 Comparation of different photolithographic QLEDs in pixel density and peak EQE.

Comment: *4) Are there any fundamental reasons why this approach can achieve a higher resolution than previously reported methods? This work simply employs ligand-mediated crosslinking, i.e., direct photopatterning, which is already a well-established technique. Therefore, it is not clear that the proposed crosslinking/de-crosslinking strategy can intrinsically provide better pattern fidelity or higher resolution than existing direct photopatterning approaches.*

Response:

Dear reviewer, thanks for your insightful and constructive comment. In terms of resolution, our reversible photolithography strategy achieves an attractive pixel density of ~21,000 PPI, which is comparable to or even better than that of conventional direct photolithography approaches. We attribute this to two main factors: **(1) the thiol-anchoring group of the photo-reversible ligand binds strongly to the QD surface**, which has been demonstrated in Figure 3d, **resulting in minimal chemical diffusion**; (2) **the photo-crosslinking reaction is highly site-selective**

and efficient (with short exposure time and low dose), offering great potential for high resolution. Corresponding discussion has been added in the revised manuscript.

Change made on the first paragraph of Page 15: "We attribute the attractive high resolution to two main factors: (i) the thiol-anchoring group of RPL binds strongly to the QD surface (demonstrated in **Figure 3d**), resulting in minimal chemical diffusion; (ii) the photo-crosslinking reaction is highly site-selective and efficient (with short exposure time and low dose)."

Furthermore, your comment further prompted us to explore the potential of our reversible photolithography strategy for enhancing the processability of QDs. Specifically, by applying a secondary trimming step to the crosslinked QD patterns through decrosslinking, **a reconfigurable photo-patterning for resolution tuning could be achieved, which remains beyond the reach of conventional direct photolithography**. As a proof of concept, we first fabricated 20 × 50 μm QD pixels *via* photo-crosslinking, which faithfully replicated the photomask pattern (Figure R9). Subsequently, we rotated the photomask and performed 254 nm exposure and development, successfully obtaining trimmed QD pixels. Notably, the dimensions of the trimmed pixels (e.g., 15 × 20 μm) are reduced compared to the original patterns, highlighting the reconfigurability enabled by our reversible crosslinking strategy. This approach offers a viable route to achieve QD patterns with resolution beyond the photomask limit, holding great promise for future super-resolution patterning applications. And we will systematically investigate and discuss this aspect in detail in future work.

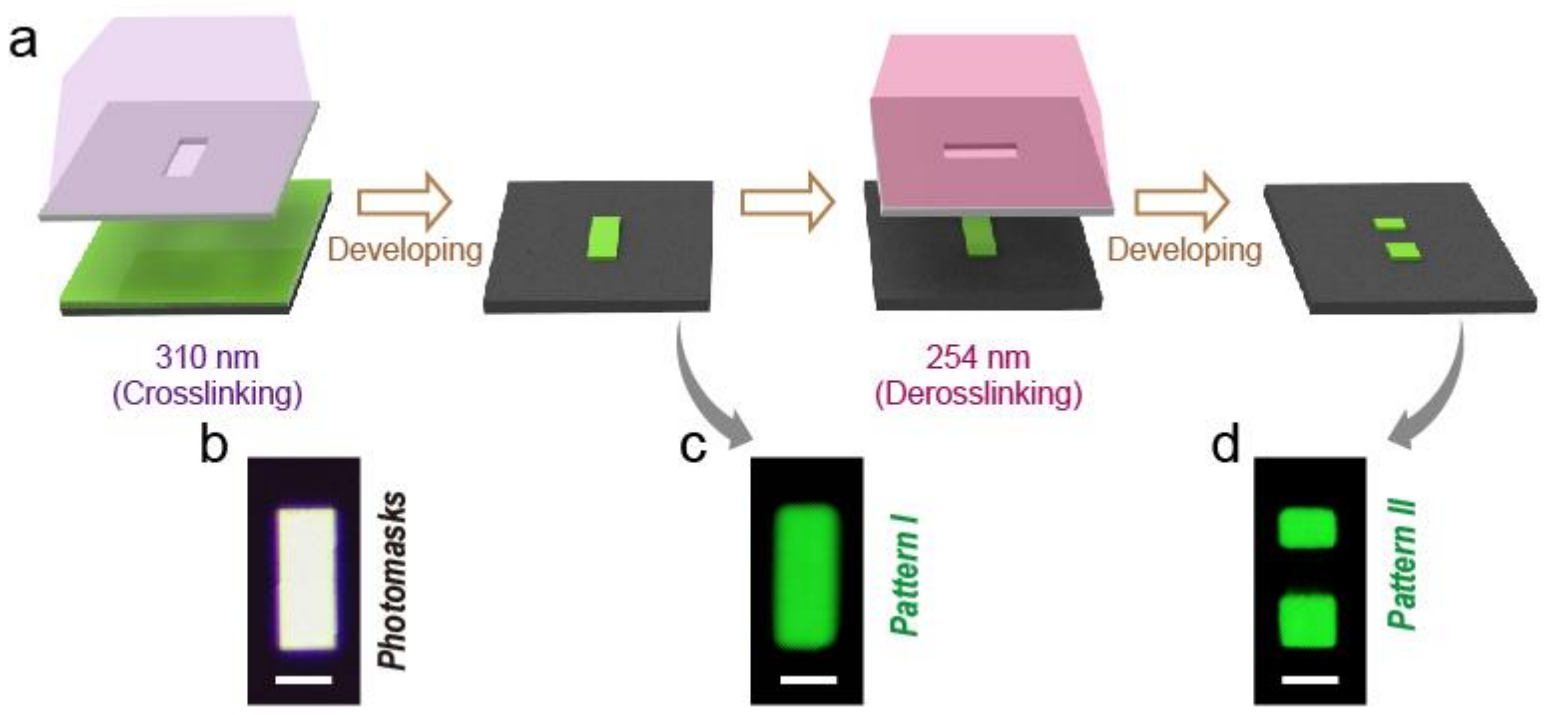

Figure R9. (a) Schematic of reconfigurable photo-patterning based on reversible photolithography strategy. (b-d) Microscopic image of the photomask (b) and corresponding fluorescent microscopic images of QDs before (c) and after (d) spatially localized decrosslinking. Scale bar: 20 μm.

Once again, we greatly appreciate your valuable suggestions, which have further strengthened the quality of our paper. We have incorporated the relevant discussions into the revised manuscript.

Change made on the last paragraph of Page 13: “Moreover, the reversible photolithography strategy also enables reconfiguration of QD patterns. As a proof of concept, we first fabricated 20 × 50 μm QD pixels *via* photo-crosslinking (Supplementary Fig. 21). The photomask was then repositioned to allow spatially localized decrosslinking, yielding trimmed QD pixels with reduced dimensions (e.g., 15 × 20 μm).”

Change made on Page 11 in the revised Supplementary Information:

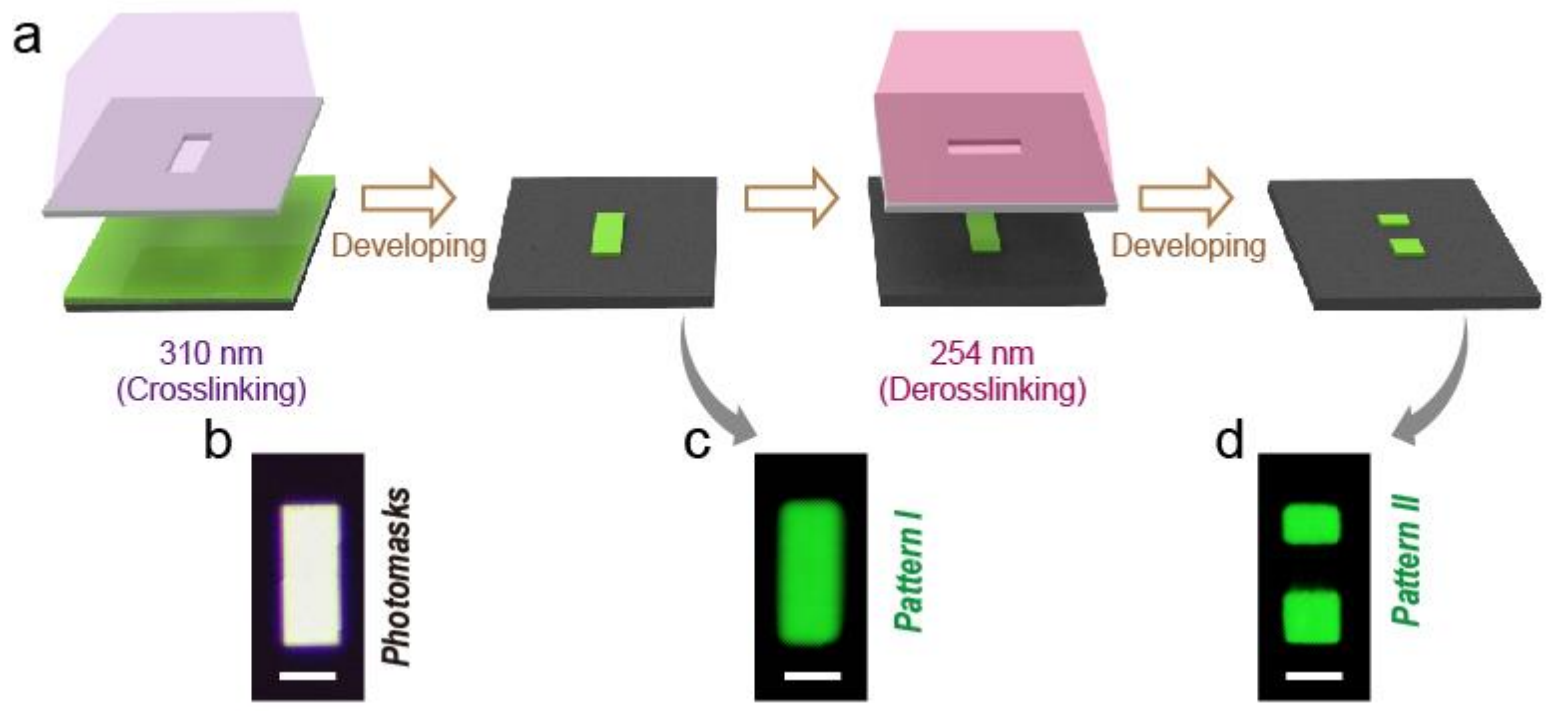

Supplementary Fig. 21 (a) Schematic of reconfigurable photo-patterning based on reversible photolithography strategy. (b-d) Microscopic image of the photomask (b) and corresponding fluorescent microscopic images of QDs before (c) and after (d) spatially localized decrosslinking. Scale bar: 20 μm.

Comment: *5) This work lacks a strategy for realizing full-color displays, as it only demonstrates a single-color EL device. In particular, after de-crosslinking, the pre-patterned QDs may be washed away during the subsequent processing steps required to pattern QDs of different colors. The authors should demonstrate full-color device fabrication while preserving the integrity of the previously formed QD patterns.*

Response:

Dear reviewer, thanks for raising such an interesting and important question. We understand your core concern that the pre-patterned QDs in the decrosslinked state could be washed away during subsequent photolithographic patterning of other colors. Encouragingly, **our reversible photolithography strategy is fully compatible with the fabrication of multicolor QD patterns for achieving full-color displays**, following the procedure outlined below.

As shown in Figure R10a, first, red QD pixels are prepared through in situ photo-induced crosslinking and developing. Multicolor QD pixels are then obtained by repeating this procedure for green and blue QDs. In this case, no mutual erosion occurs between QD patterns, because the patterned QDs remain in the crosslinked state.

Subsequently, a DUV-induced decrosslinking step is performed to restore the pristine ligand states of all QD pixels. Using this procedure, multicolor QD integration based on reversible photolithography is readily achievable.

As a proof of concept, **we successfully fabricated corresponding red (R), green (G), and blue (B) QD pixel arrays** (Figure R10b). Furthermore, we also verified the vertical stacking capability of these QD patterns (Figure R10c). The overlapping regions of RGB pixels effectively generated three secondary colors (e.g., cyan, orange, and pink). Meanwhile, all QD pixels exhibited well-defined shapes with clear boundaries, confirming that no mutual erosion occurred during the multiple reversible photolithography cycles. This result fully alleviates your concern about the incompatibility of multicolor QD integration.

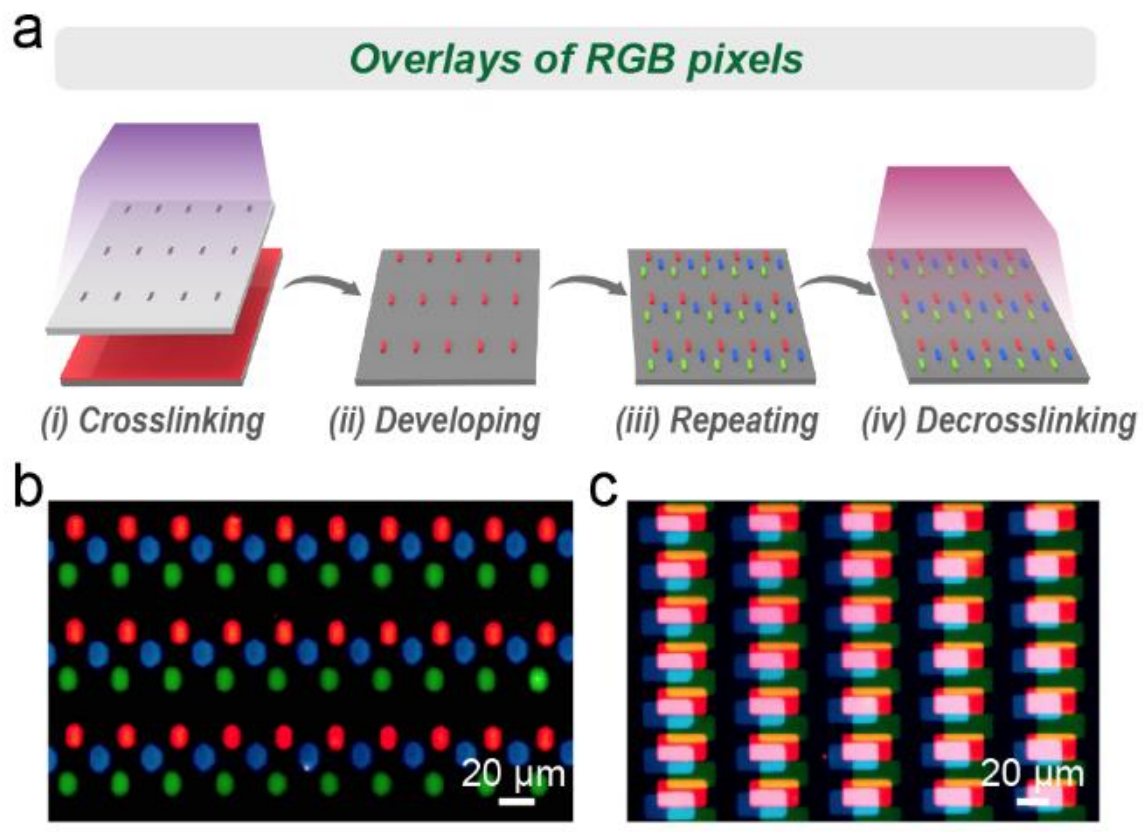

Figure R10. (a) Schematic of overlays of RGB pixels through reversible photolithography. (b) Fluorescent microscopic image of lateral overlays of RGB pixels. (c) Fluorescent microscopic image of vertical stacking of RGB pixels.

Furthermore, because different QD colors typically require distinct charge transport layers and device structures, the electroluminescence performance of multicolor QLEDs with non-pixelated functional layers is not yet satisfactory. We have therefore omitted a detailed presentation of these device results here, as they fall outside the scope of this work.

Change made on the third paragraph of Page 13: “Encouragingly, the reversible photolithography strategy remains fully compatible with multicolor QD integration. As shown in **Figure 5g**, multicolor QD patterns—for example, overlays of RGB pixels—are prepared through in-situ photo-induced crosslinking and developing. During this process, no mutual erosion occurs, because the pre-patterned QDs remain in the crosslinked state. Subsequently, a DUV-induced decrosslinking step restores the pristine ligand states of all QD pixels. As a result, we successfully fabricated RGB QD pixel arrays on the same substrate (**Figure 5g**). Furthermore, the vertical stacking of RGB pixels

generated three secondary colors—cyan, orange, and pink—while maintaining well-defined pixel shapes and clear boundaries. These results lay a solid foundation for future full-color display applications."

Change made on Page 14:

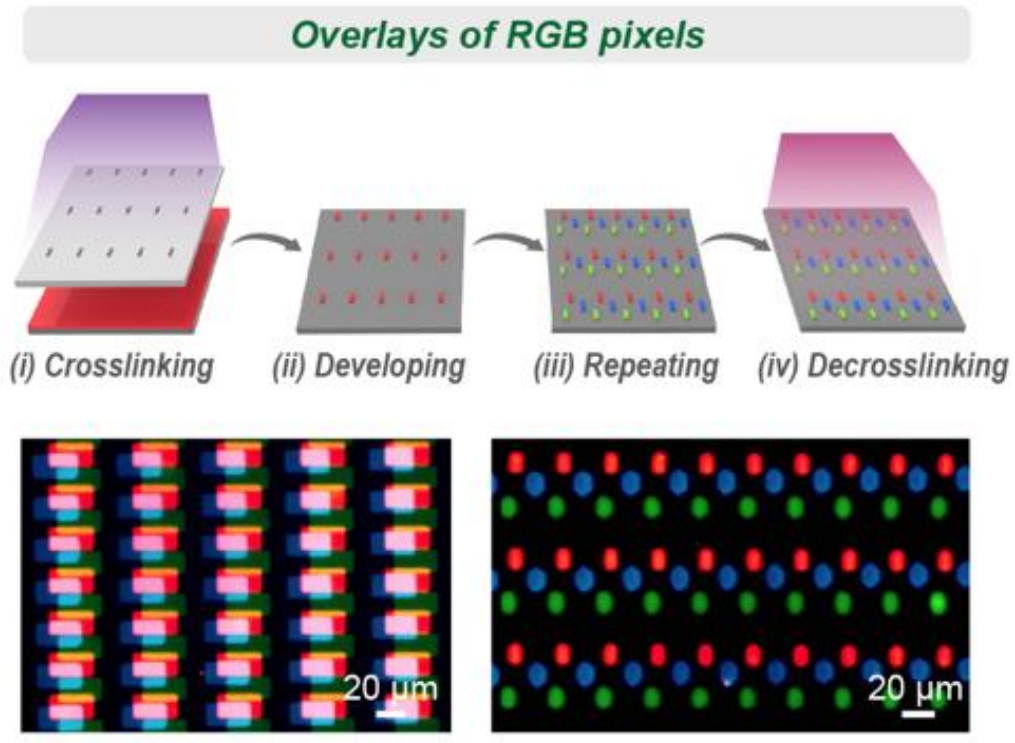

Figure 5 (g) Top: Schematic of overlays of RGB pixels through reversible photolithography. Bottom: Fluorescent microscopic images of overlays of RGB pixels.

Comment: *6) "This performance trade-off is commonly observed in direct QD photolithography via photo-crosslinking,[24,32,56]". This cannot be agreeable. Please find recent reports that show no trade-off. The performance trade-off has been often reported but recently many reports do not show trade-off. Rather, some recent reports show no changes or improvements after patterning.*

Response:

Dear reviewer, we thank you for raising this important point, which allows us to refine our discussion.

From a broader perspective, **the limitations of patterning photochemistry in direct QD photolithography have been widely recognized in the field**, as systematically summarized in a recent work [Ref: J. Am. Chem. Soc. 2024, 146, 28895]. For instance, ligand decomposition generates compact ionic ligands that are less effective at passivating surface traps [Ref: Chem. Rev. 2026, 126, 3624]; photoacid generator-mediated ligand exchange often suffers from acid-induced degradation and surface defects [Ref: Adv. Mater. 2020, 32, 2003805]; ligand crosslinking leads to highly crosslinked networks that increase electrical resistance [Ref: ACS Nano 2025, 19, 22253; Small Methods 2023, 7, 2300206]. **These examples collectively illustrate that the performance trade-off is not an artifact of specific conditions, but rather a fundamental consequence of the photochemistry involved in the patterning process itself**.

Change made on the last paragraph of Page 2: "For instance, compact ionic ligands generated from ligand decomposition are less effective at passivating surface traps.[29] Ligand exchange processes mediated by photoacid generators often suffer from acid-induced degradation and the formation of surface defects.[19] Highly crosslinked ligand networks tend to increase electrical resistance.[24,35,37]"

It is also worth noting that **even in several recent high-quality reports, a clear decline in luminance (*L*) and conductivity after photo-crosslinking is still observable**. For example, rigid azide-based crosslinkers reduced the L_{max} of red, green, and blue QLEDs from 241,844/436,033/9,079 cd m^{-2} to 179,872/244,844/4,939 cd m^{-2}, respectively [Ref: Light Sci. Appl. 2025, 14, 251]. A triphenylphosphine-based system similarly decreased the L_{max} of blue QLEDs from 7,851 to 7,472 cd m^{-2} [Ref: Nat. Commun. 2025, 16, 9689], while a diazo-based thermocrosslinker reduced current density from 18.74 to 12.80 mA cm^{-2} and *L* from 3,949.85 to 2,606.06 cd m^{-2} at 6.0 V [Ref: Nat. Commun. 2026, 17, 4162]. **The reduced conductivity induced by the crosslinkers and the crosslinked network is widely regarded as a major cause,** supported by both reports [Ref: Small 2026, 22, e00011; ACS Nano 2025, 19, 22253; Small Methods 2023, 7, 2300206; Adv. Mater. 2022, 34, 2205504] and our own measurements (Figure 3f).

On the other hand, we agree that some recent studies have made encouraging progress in mitigating performance loss and even achieving performance improvement. For instance, post-patterning ligand exchange—such as replacing long-chain ligands with short-chain ammonium halides—has been shown to enable denser surface passivation and enhanced charge transport [Ref: Adv. Mater. 2025, 37, e08217]. However, while effective, these approaches are invasive and challenging to control precisely. Therefore, a straightforward strategy that can restore the surface ligand states of QDs is highly desirable for achieving high-performance electroluminescence. Here, we have realized this goal through non-invasive reversible photolithography of QDs. This has already been discussed in the Introduction of our original manuscript.

In light of both the persistent trade-off and the continued progress in the field, we have revised the relevant statement accordingly. We hope that this revision adequately addresses your concern.

Change made on the first paragraph of Page 11: "This performance trade-off is also observed in other systems based on direct QD photolithography *via* photo-crosslinking.[34,56,57] And it is primarily attributed to the reduced conductivity within crosslinked QD networks, as demonstrated by others[24,35,37,39] and confirmed in this work (**Figure 3f**)."

Change made on Page 20: "[57] Kim, H. et al. Photoresist-guided indirect photopatterning of quantum dots via carbene-mediated ligand thermocrosslinking. *Nat. Commun.* **17**, 4162 (2026)."

Comment: *7) For the photo-crosslinking, they described "the film was exposed to suitable UV irradiation (centered at 310 nm) with or without photomasks for required patterns in the air or the nitrogen filled glove box.". It is not clear whether they use QD patterns processed in air or in the nitrogen atmosphere. They need to indicate the condition carefully for each datum. Also, it is recommendable to show at least PLQYs depending on both the conditions.*

Response:

Dear reviewer, we thank you for pointing out this ambiguity in our original manuscript. In our work, **the reversible photolithography process was, unless otherwise stated, conducted in a nitrogen-filled glovebox**. Only the direct photo-patterning validation presented in Figures 5a-g was performed under ambient conditions. To avoid any potential misunderstanding, we have now clearly annotated these conditions throughout the revised manuscript.

Change made on the legend of Page 14: "All results in (a-g) were obtained under ambient conditions and following the complete reversible photolithography workflow, including the decrosslinking step."

Change made on Method of Page 16: "Note: The reversible photolithography process can be conducted either under ambient conditions or in a nitrogen-filled glove box. Unless otherwise stated, all experiments were performed in a nitrogen-filled glove box by default."

According to your valuable suggestion, **we have supplemented additional experiments monitoring the PL QYs during the reversible photolithography process under ambient conditions**. As shown in Figure R11, the results show that the introduction of PRL leads to a certain degree of PL QY enhancement (similar to the PLQY change in the glovebox), which we attribute to effective passivation of PRL to the QD surface. However, in contrast to the glovebox results, both the crosslinking and decrosslinking steps in air caused a certain degree of PL QY decrease, likely due to UV-induced degradation of the QDs assisted by water and oxygen. Nevertheless, **the as-prepared decrosslinked PRL-GQD film still retained a high PL QY of 91.8% relative to the initial value**. This result suggests that our strategy also holds potential for low-cost processing under ambient conditions. The corresponding experimental results and discussions have been incorporated into the revised manuscript.

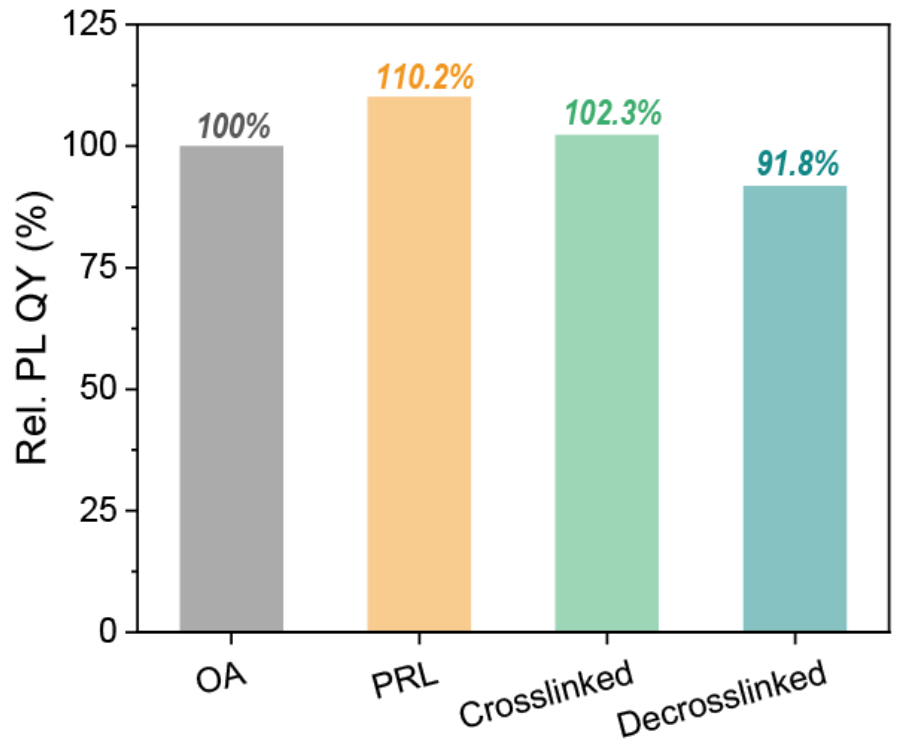

Figure R11. Relative PL QYs of OA-GQD, PRL-GQD, crosslinked PRL-GQD and decrosslinked PRL-GQD. The reversible photolithography process is carried out under ambient conditions.

Change made on the first paragraph of Page 12: "Although the PL QY retention under ambient conditions is relatively lower (~91.8%) than that achieved in the nitrogen-filled glovebox (Supplementary Fig. 16), likely due to UV-induced degradation of QDs assisted by water and oxygen, this result still highlights the potential of our strategy for low-cost processing in air—an attractive feature for future practical applications."

Change made on Page 9 in the revised Supplementary Information:

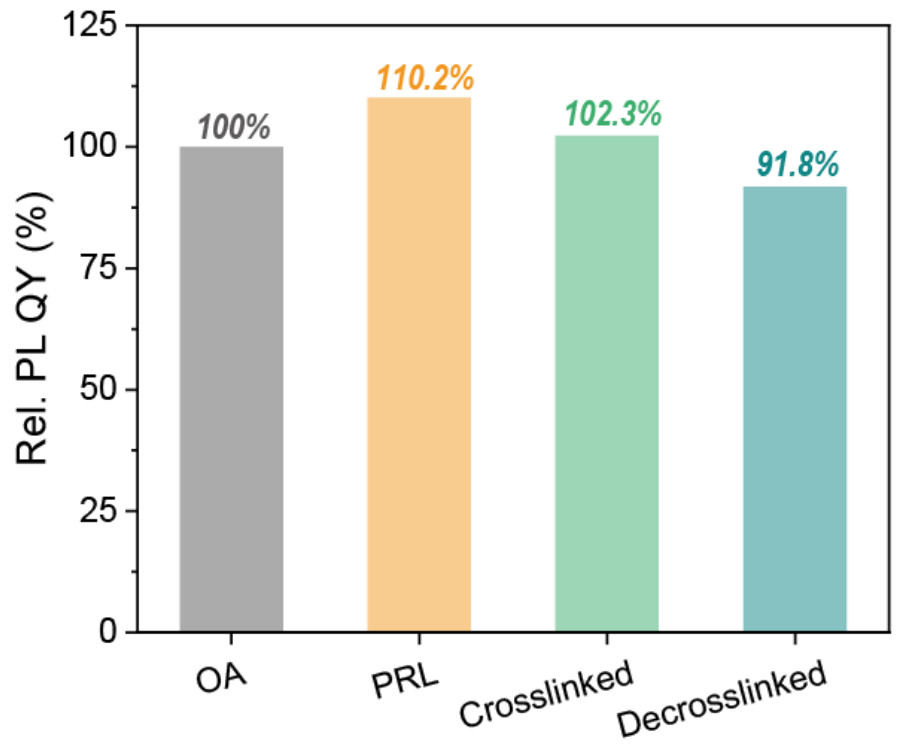

Supplementary Fig. 16 Relative PL QYs of OA-GQD, PRL-GQD, crosslinked PRL-GQD and decrosslinked PRL-GQD. The reversible photolithography process is carried out under ambient conditions.

Comment: *8) The authors claimed "we fabricated QLEDs with EL pixel arrays spanning resolutions from 3,300 PPI (pixel size: ~4.5 μm) to 21,000 PPI (pixel size: ~0.8 μm), as illustrated in Figure 5f.". However, there is no clear description of fabricating such high-resolution EL devices. Also, it is quite unclear whether they really fabricated such high-pixellated QLEDs or just fabricated 21,000 PPI that can be applicable for real QLEDs, because they mentioned following "Notably, pixelated QLEDs suffer from pronounced leakage currents due to direct HTL/ETL*

contact in non-patterned regions, a challenge that typically demands further device architecture optimization.[61,62] And research in this field remains primarily concentrated on non-pixelated devices, as summarized in a recent review.[29] Consequently, comprehensive performance characterization of pixelated devices was not pursued in this study to prioritize demonstration of photosensitive materials and photolithographic fidelity."

<u>Response:</u>

Dear reviewer, we sincerely thank you for your interest in the fabrication and performance of pixelated QLED devices. This is indeed a very crucial but also very challenging research topic in this field.

In fact, **the images presented in Figure 5f of our original manuscript already show pixelated QLED devices** spanning resolutions from 3,300 PPI (pixel size: ~4.5 μm) to 21,000 PPI (pixel size: ~0.8 μm). Briefly, we integrated QD pixels (fabricated our reversible photolithography strategy) into the device fabrication process, as explicitly described in the original manuscript and Supplementary Information. For example, the description in ***Supplementary Note 8 (Preparation of QLEDs)*** described that "*<u>the pixelated photomasks with different pixel arrays were introduced to prepare pixelated QLEDs.</u>*"

On the other hand, as we all know, QLED devices based on patterned/pixelated QDs generally suffer from severe leakage current, primarily due to direct HTL/ETL contact in non-patterned regions, leading to very poor electroluminescence performance. Consequently, research in this field has predominantly focused on non-pixelated devices, as summarized in a recent review [Ref: Chem. Rev. 2026, 126, 3624]. This point has already been described in detail in our original manuscript.

Nevertheless, we fully understand your interest in the electroluminescence performance of the pixelated QLEDs. **In response, we have fabricated corresponding nano-pixelated QLED devices at a resolution of 21,000 PPI, achieving a highly competitive peak EQE of 17.08%** (Figure 5i and Supplementary Fig. 24). This result further underscores the effectiveness of our reversible photolithography strategy in simultaneously achieving high-resolution patterning and high device performance. Full details are provided in our response to your preceding comment, and we do not elaborate further here.

Comment: *9) The electrical performance of the pixelated QLEDs fabricated using the crosslinking/de-crosslinking process, regardless of pixel size, is not presented. The authors should provide the electrical characteristics of these pixelated devices using a reliable test platform to demonstrate the practicality and effectiveness of their approach.*

Response:

Dear reviewer, we thank you again for your interest in our pixelated QLED devices. In the revised manuscript, **we have added the performance results of ultrahigh-resolution nano-pixelated QLEDs with a highly competitive peak EQE of 17.08% at 21,000 PPI**. As extensively discussed above, this point is not repeated here.

We are also grateful for your time and careful evaluation. Based on your constructive suggestions, we have thoroughly revised the manuscript and supplemented it with extensive new experimental data. We believe these improvements have further strengthened the novelty and advancements of our work, making it suitable for *Nature Communications*. We sincerely hope that the revised version meets your expectations and those of the journal's readership.

To reviewer 3

Comment: *This manuscript reports a reversible direct photolithography strategy for colloidal quantum dots based on a coumarin-functionalized photo-reversible ligand, termed PRL. The proposed process uses approximately 310 nm UV irradiation to induce coumarin photocycloaddition and inter-QD crosslinking for pattern formation, followed by 254 nm deep-UV irradiation to trigger decrosslinking and recover the ligand state and optoelectronic properties. The authors report low exposure dose, high PLQY retention, recovered conductivity and QLED performance after decrosslinking, a non-pixelated green QLED with a peak EQE of 24.41% and a luminance of 125,016 cd m^{-2} at 5.0 V, and EL pixel arrays with a claimed density of 21,000 PPI.*

The concept is timely and potentially important. It addresses a central limitation in direct QD photolithography: the trade-off between crosslinking-enabled patternability and degraded optoelectronic performance. The manuscript is generally well organized and contains a broad set of optical, chemical, structural, electrical, and device-level characterizations.

However, the central claims are not yet sufficiently supported. The high-performance electroluminescence is demonstrated in non-patterned, full-area decrosslinked QLEDs, whereas the ultrahigh-resolution 21,000 PPI EL pixel arrays are not quantitatively characterized and appear to remain in the crosslinked patterned state. In addition, the reversible surface chemistry of PRL on QD films is inferred rather than directly demonstrated; and some mechanistic interpretations and simulation methods require correction. I therefore recommend major revision.

Response:

Dear reviewer, thanks a lot for your time and efforts to review our manuscript. We also appreciate your positive recognition, particularly that "*The concept is timely and potentially important*" and "*The manuscript is generally well organized and contains a broad set of optical, chemical, structural, electrical, and device-level characterizations*". We have now provided comprehensive responses to all of your comments and have revised both the manuscript and the Supplementary Information accordingly. We believe that, with the constructive input from you and the other reviewers, the quality of our work has been significantly enhanced.

(1) Regarding the structure and performance of the electroluminescent pixels

In our original manuscript, we presented detailed performance data for non-patterned QLEDs to demonstrate the value of reversible photolithography in achieving high-performance electroluminescence. We also fabricated

nano-pixelated QLEDs with a resolution of 21,000 PPI. However, an in-depth discussion of the pixel morphology was not included.

To address this, **we performed atomic force microscopy (AFM) measurements on the QD pixels at this resolution**. As shown in Figure R11, the pixels exhibit well-defined boundaries without mutual adhesion. And **the as-fabricated nano-pixelated QLED devices still achieve a highly competitive EQE of 17.08%** (Figure R12). These additional results further reinforce the advancements of our work. Detailed data and discussion have been incorporated into the revised manuscript and Supplementary Information.

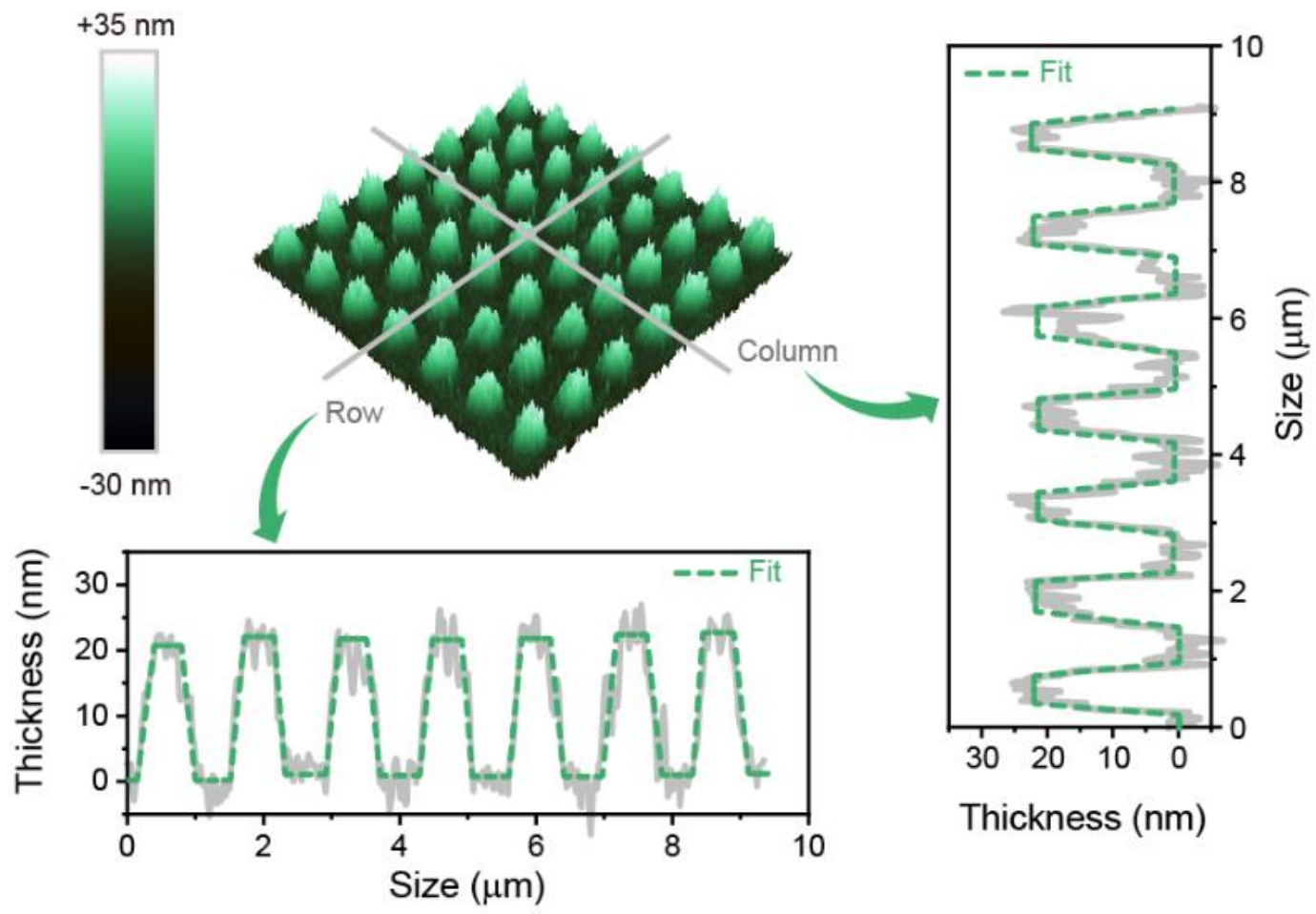

Figure R11. Three-dimensional AFM image and surface profile of QD pixels on silicon wafer at ~21,000 PPI.

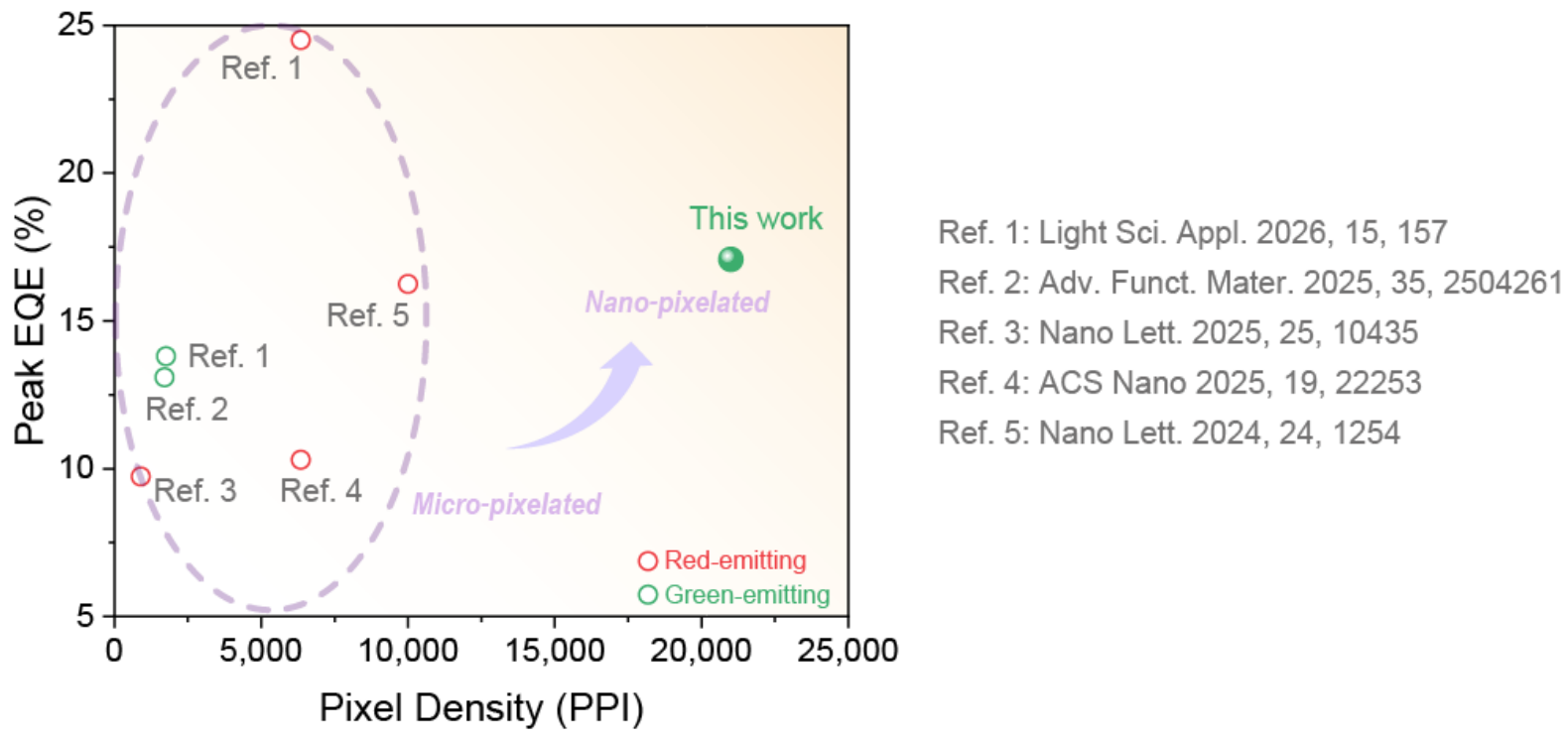

Figure R12. Comparation of different photolithographic QLEDs in pixel density and peak EQE.

Meanwhile, it should be clarified that **all QD patterns were fabricated through the complete reversible photolithography workflow, including the decrosslinking step**. This point was not explicitly stated in the original manuscript, and we have now added a clear statement: "All results in (a-g) were obtained under ambient conditions and following the complete reversible photolithography workflow, including the decrosslinking step" on page 14.

(2) Regarding the direct evidence for the reversible surface chemistry of PRL and others

In addition, we have addressed your other comments—including those concerning direct evidence for the reversible surface chemistry of PRL—in a point-by-point manner in the following sections.

Comment: *1. The core chemical claim is that surface-bound PRL undergoes reversible coumarin dimerization and photocleavage on QD films. The current evidence mainly includes UV-vis changes of free PRL, macroscopic solubility changes of QD films, PLQY retention, GISAXS, conductivity, and QLED performance recovery. These data are consistent with the proposed mechanism, but they do not directly prove that QD-bound PRL undergoes a reversible coumarin crosslinking/decrosslinking cycle. This issue is particularly important because PRL contains only one coumarin terminal group and the reported surface content is approximately 8 mol%. The authors should clarify whether the dominant dimerization event occurs between ligands on neighboring QDs or between ligands on the same QD. These two cases would have different implications for film insolubilization, inter-QD spacing, and charge transport.*

Response:

Dear reviewer, thanks a lot for your meaningful suggestion. To provide direct evidence for the reversible crosslinking/decrosslinking cycle of PRL on the QD surface, **we performed in situ FT-IR monitoring of the PRL-GQD film during the reversible photolithography process**. As shown in Figure R13, upon exposure to 310 nm UV irradiation, the signal at 1614 cm^{-1} corresponding to the C=C stretching vibration gradually decreased, indicating the consumption of coumarin groups (cyclobutane ring formation) [Ref: Adv. Funct. Mater. 2020, 30, 1909359]. This phenomenon confirms the occurrence of photo-crosslinking reactions on the QD surface. And then, upon subsequent 254 nm UV irradiation, this signal gradually recovered, reflecting the decrosslinking process. **This reversible behavior was further verified by in situ UV-vis absorption spectroscopy of the PRL-GQD film**. As shown in Figure R14, alternating exposure to 310 nm and 254 nm UV light resulted in decreases and increases in the absorbance at ~318 nm, which is consistent with the spectral changes of free PRL.

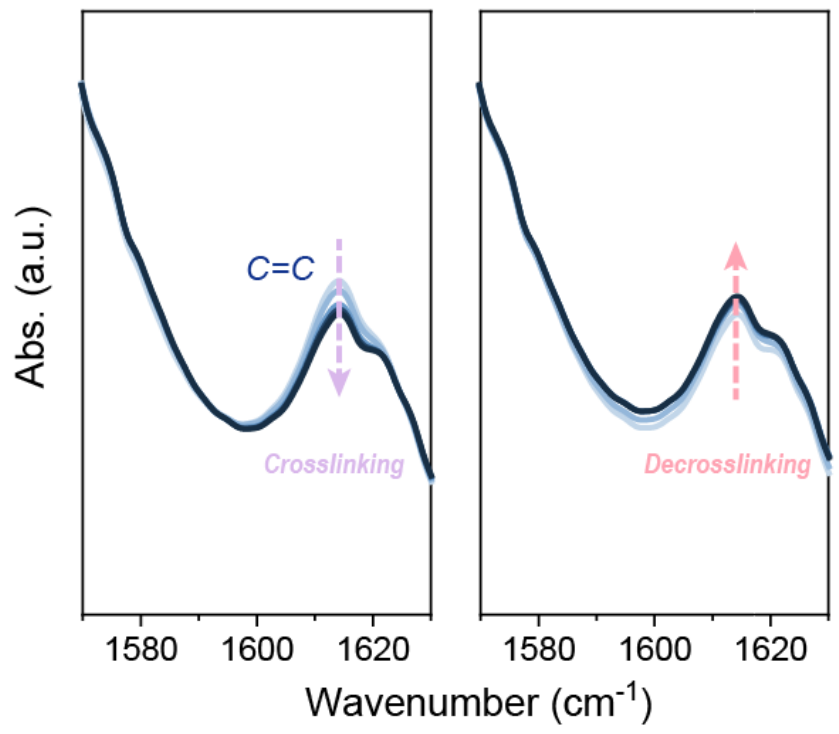

Figure R13. FT-IR spectra of PRL-GQD film during the photo-induced crosslinking process (left) and decrosslinking process (right).

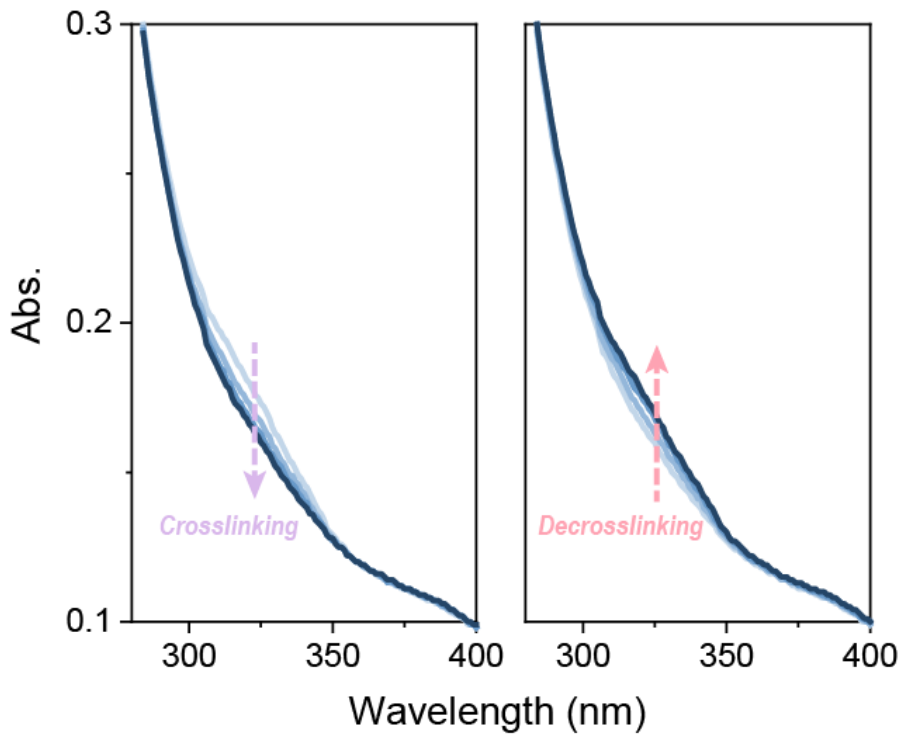

Figure R14. UV-vis spectra of PRL-GQD film during the photo-induced crosslinking process (left) and decrosslinking process (right).

Change made on the second paragraph of Page 7: "The mechanism of reversible photolithography of PRL-GQD is verified through in-situ Fourier transform infrared (FT-IR) spectroscopy (Supplementary Fig. 5). Upon 310 nm UV exposure, the signal at 1614 cm^{-1}, corresponding to the C=C stretching vibration, gradually decreased, indicating the consumption of coumarin groups during crosslinking. Subsequently, upon DUV exposure, this signal gradually recovered, reflecting the decrosslinking process. This reversible behavior was further confirmed by in-situ UV-Vis absorption spectroscopy, which showed reversible changes in the absorbance of PRL (Supplementary Fig. 6)."

Change made on Page 4 in the revised Supplementary Information:

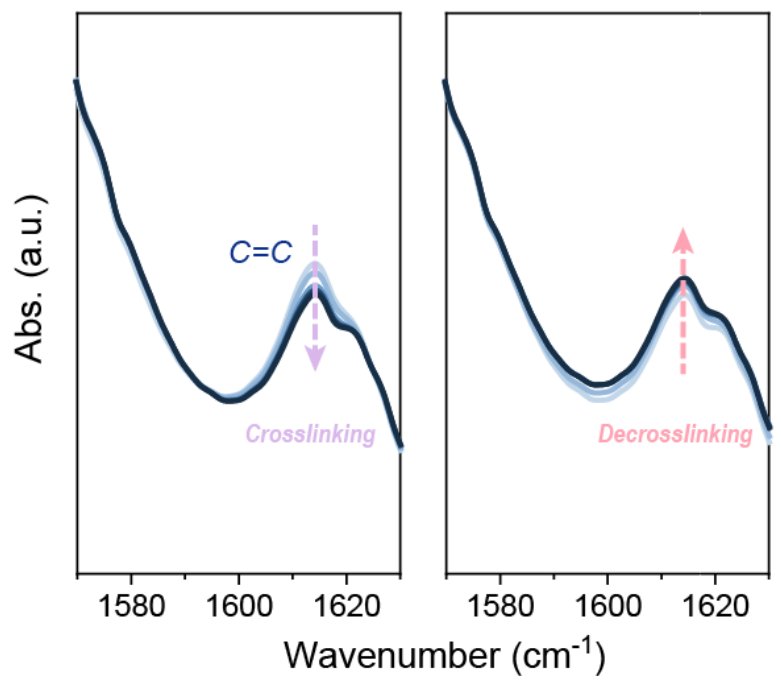

Supplementary Fig. 5 FT-IR spectra of PRL-GQD film during the photo-induced crosslinking process (left) and decrosslinking process (right).

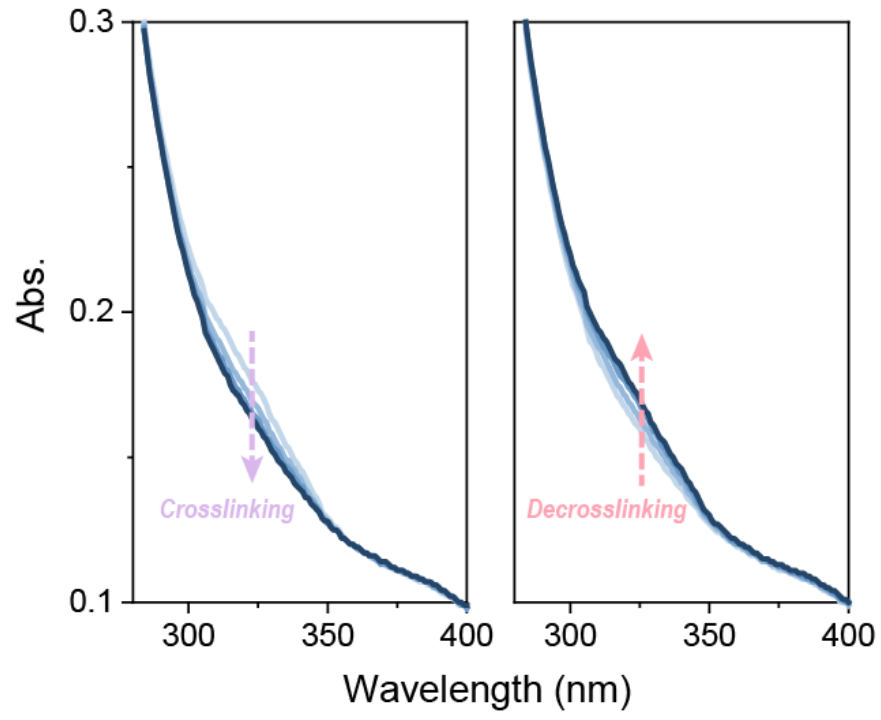

Supplementary Fig. 6 UV-Vis spectra of PRL-GQD film during the photo-induced crosslinking process (left) and decrosslinking process (right).

Regarding your another question concerning whether the crosslinking of PRL occurs primarily between ligands on neighboring QDs (inter-QD pathway) or between ligands on the same QD (intra-QD pathway), **we conclude that the inter-QD pathway is dominant**. This inference is based on the observation that intra-QD crosslinking pathway would have a relatively minor impact on QD dispersibility, whereas our system exhibits a pronounced dispersibility change and enables photolithographic patterning at ultralow exposure doses.

To further support this inference, we monitored the characteristic absorption signal of coumarin groups under 310 nm UV irradiation in a dilute PRL-GQD solution, where the inter-QD pathway is significantly limited. As shown in Figure R15, **the characteristic absorption signal showed only negligible changes with increasing exposure time, indicating that photocycloaddition (photo-crosslinking) of coumarin groups occurs only to a very limited extent**. In contrast, under the same exposure time, the dense PRL-GQD film exhibited a rapid decrease of the characteristic absorption signal—particularly within the typical exposure time used in this work (5 s). These results collectively confirm that **inter-QD crosslinking is the dominant pathway during the reversible photolithography** of the PRL-GQD film, which effectively supports the main conclusions of this work.

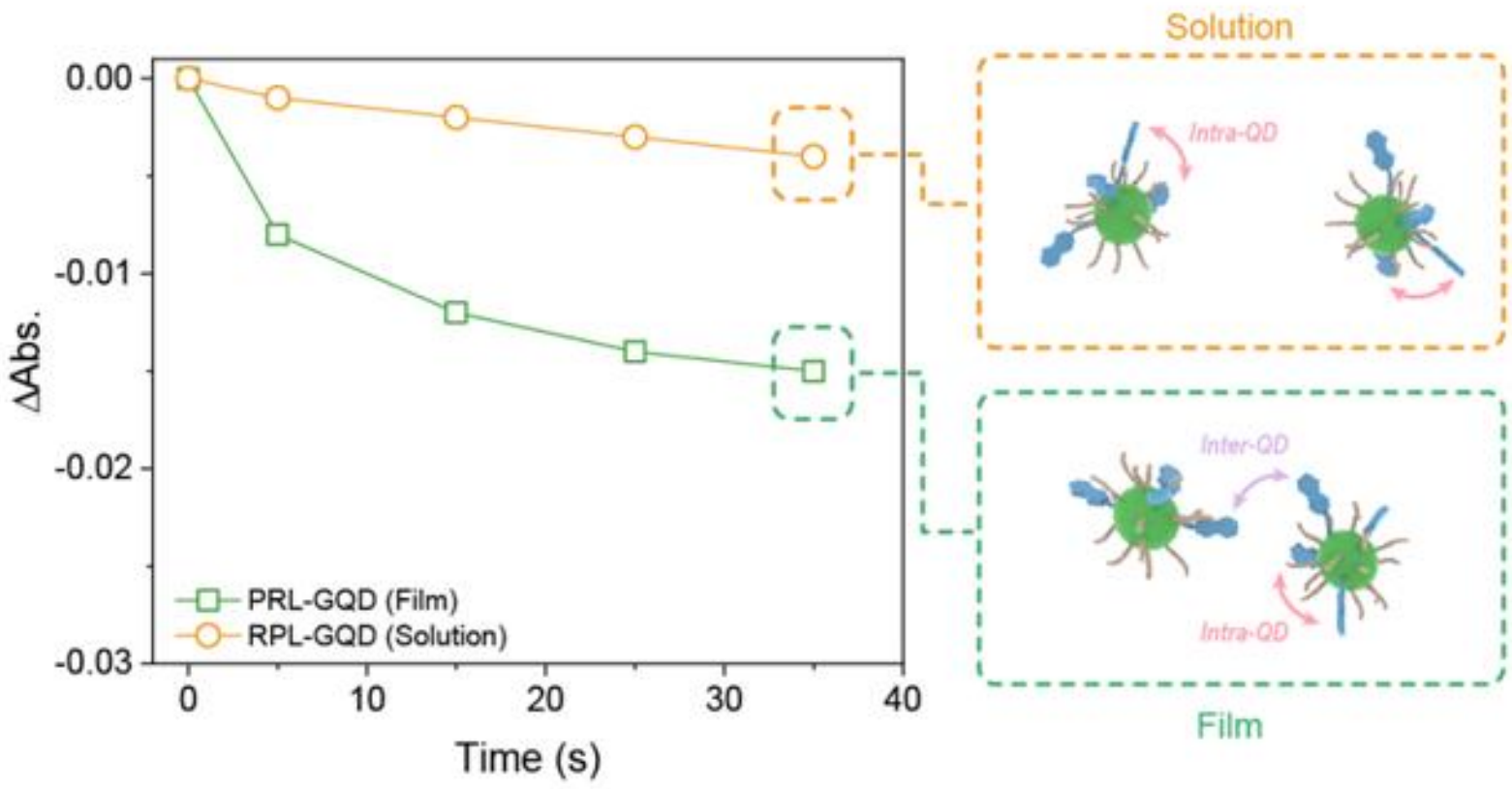

Figure R15. Left: absorption changes at 318 nm of RPL-GQD film and PRL-GQD solution (0.1 mg/mL in toluene) under UV exposure (centered at 310 nm, ~1 mW/cm^2). Right: potential intra-QD and inter-QD pathways in these systems.

Change made on the last paragraph of Page 7: "To determine whether the photo-crosslinking occurs predominantly between ligands on neighboring QDs (inter-QD pathway) or between ligands on the same QD (intra-QD pathway), we compared the crosslinking kinetics between a dilute PRL-GQD solution and a dense PRL-GQD film. As shown in Supplementary Fig. 7, the PRL-GQD solution with limited inter-QD crosslinking exhibited a substantially slower reaction rate compared to the PRL-GQD film, particularly within the typical exposure dose used in this work (≤ 5 mJ/cm^2). This result effectively confirms that inter-QD crosslinking is the dominant pathway."

Change made on Page 5 in the revised Supplementary Information:

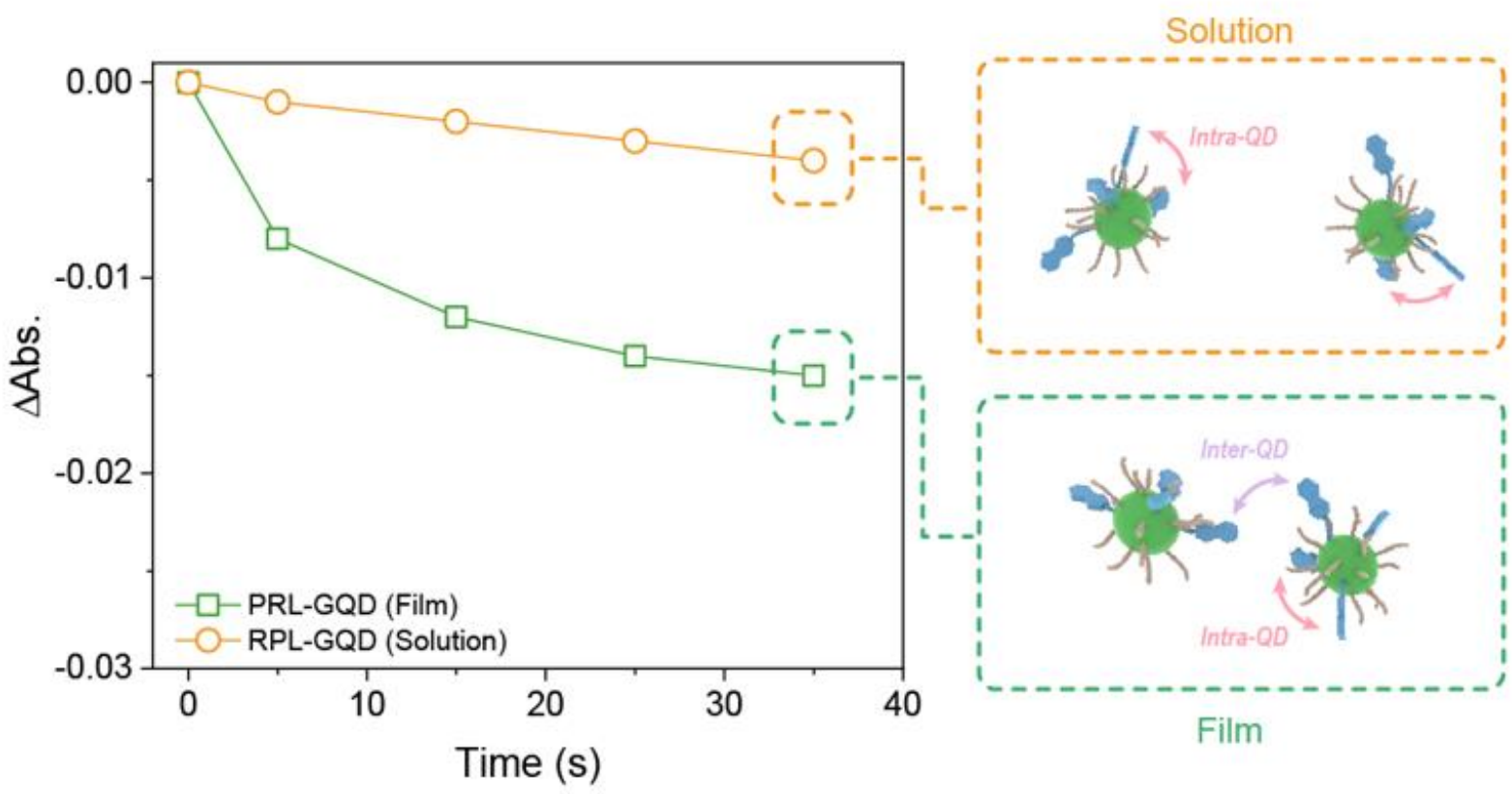

Supplementary Fig. 7 Left: absorption changes at 318 nm of RPL-GQD film and PRL-GQD solution (0.1 mg/mL in toluene) under UV exposure (centered at 310 nm ~1 mW/cm^2). Right: potential intra-QD and inter-QD pathways in these systems.

Comment: *2. State-of-the-art is overstated. (a) The decrosslinked luminance (125,016 cd/m²) is below the authors' own pristine control (134,796 cd/m²): the process recovers ~93%, it does not set a record. (b) The decrosslinked peak EQE (24.41%) is essentially equal to pristine (24.43%) and not higher than recent reports (e.g., the ref 61 at 24.5%; Guan et al. up to 25.6% green in the authors' own Table S1). (c) Table S1 lists others reaching ~200,000 cd/m². Please reframe the claims. The record 21,000 PPI claim should also be clearly limited to direct QD photolithography demonstrations.*

Response:

Dear reviewer, we appreciate your constructive suggestions. Your careful and critical reading has been very helpful in improving our manuscript. In the revised manuscript, we have avoided the use of "state-of-the-art" and adopted more objective phrasing such as "**one of the highest**" or "**competitive**". In addition, the claim of a "record 21,000 PPI" has been explicitly contextualized within the field of direct QD photolithography.

Comment: *3. The authors extract an energy Eb from Arrhenius fitting of temperature-dependent PL intensity and refer to it as the exciton binding energy. They further attribute its change to a local dipole/dielectric modification caused by coumarin dimerization, which is then linked to the low-voltage EQE decrease. This causal chain is currently under-supported. The energy extracted from thermal PL quenching is more appropriately described as a thermal activation energy for PL quenching, unless additional evidence is provided to justify its equivalence to the exciton binding energy. Temperature-dependent PL intensity in QD solids can be affected by trap activation, nonradiative recombination, carrier localization, energy transfer, and ligand dynamics. It cannot be assigned uniquely to exciton binding energy from a single Arrhenius fit.*

Response:

We sincerely thank the reviewer for the insightful comments on the potential mechanisms of thermal PL quenching, which have significantly enhanced the scientific rigor of our work.

We fully agree that trap activation, non-radiative recombination, carrier localization, energy transfer, and ligand dynamics can all influence the temperature-dependent PL behavior. Therefore, we acknowledge that the energy extracted from a single Arrhenius fit cannot be uniquely assigned to the exciton binding energy without additional evidence. In the revised manuscript, **we have revised the terminology accordingly in the manuscript**

that replacing "exciton binding energy (E_b)" with "thermal activation energy (E_a)" for PL quenching. And the descriptions in Eq (1) and Figure 3g have been revised also.

To further clarify the origin of the observed E_a changes, we performed time-resolved transient absorption (TA) spectroscopy on QD films before and after crosslinking and decrosslinking. As shown in Figure R16, the TA spectra and decay dynamics remain essentially unchanged upon crosslinking and decrosslinking, which effectively excludes the interference of trap activation, non-radiative recombination, carrier localization, energy transfer, and ligand dynamics. A detailed analysis is provided below.

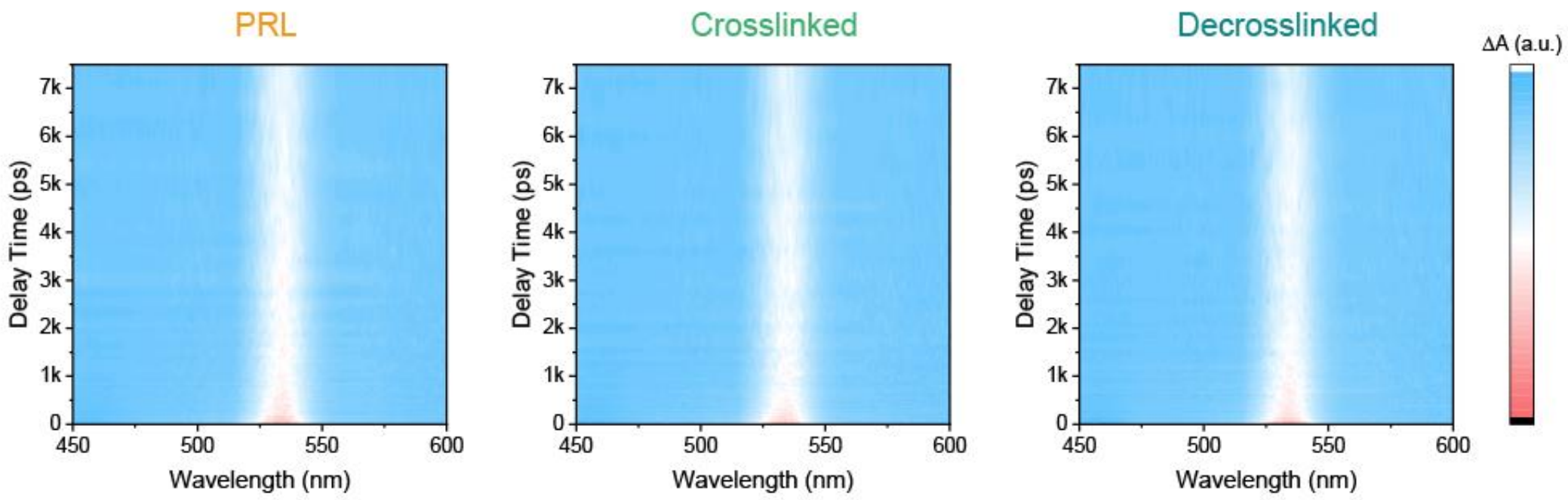

Figure R16. 2D pseudo-color fs-TA plots of PRL-GQD (left), crosslinked PRL-GQD (middle) and decrosslinked PRL-GQD (right).

(i) Trap Activation & Non-radiative Recombination: The TA spectra of QD films in the reversible photolithography process exhibit nearly identical features and decay dynamics. Since TA is highly sensitive to carrier trapping and non-radiative recombination pathways, this result effectively rules out trap activation and non-radiative recombination as the primary causes of the observed E_a variation.

(ii) Carrier Localization & Energy Transfer: The TA spectra show no significant changes or new features upon crosslinking and decrosslinking processes, ruling out severe carrier localization and any anomalous energy transfer processes from quantized states to surface trap states.

(iii) Ligand Dynamics: We note that the structural change of the photo-reversible ligand upon crosslinking and/or decrosslinking occurs at the terminal coumarin moiety, while the thiol anchoring group remains firmly bound to the QD surface. Given the strong binding affinity of thiols to the QD surface and the mild processing conditions, significant ligand detachment or surface reconstruction is unlikely. This is further supported by the unchanged TA dynamics, which would be expected to respond to any substantial changes in surface ligand configuration.

Taken together, these results allow us to exclude extrinsic factors as the dominant contributor to the E_a variation. Therefore, **the variation in E_a is predominantly linked to the exciton binding energy (E_b)**, which further led to the regulation of the electroluminescence performance. And the observed reversible change in E_a—decreasing upon crosslinking and recovering upon decrosslinking—is most consistently explained by the change in the local dielectric environment [Ref: ACS Energy Lett. 2023, 8, 731]. This interpretation is supported by a substantially increased dipole moment and dielectric constant of coumarin after photocycloaddition [Ref: Adv. Funct. Mater. 2026, 36, e16451].

We have revised the discussion in the manuscript accordingly to present a more rigorous interpretation. And we believe these revisions also improve the overall readability of the paper.

Change made on the last paragraph of Page 9: "To elucidate the origin of E_a, time-resolved transient absorption (TA) spectroscopy was employed. As shown in Supplementary Fig. 13, the lack of notable signal changes upon crosslinking effectively excludes any significant contribution from defects or energy transfer. Therefore, the variation in E_a is predominantly linked to the exciton binding energy (E_b). The post-crosslinking reduction further reflects a compromised exciton binding capability, which ultimately impairs device performance, as confirmed by subsequent experiments."

Change made on Page 8 in the revised Supplementary Information:

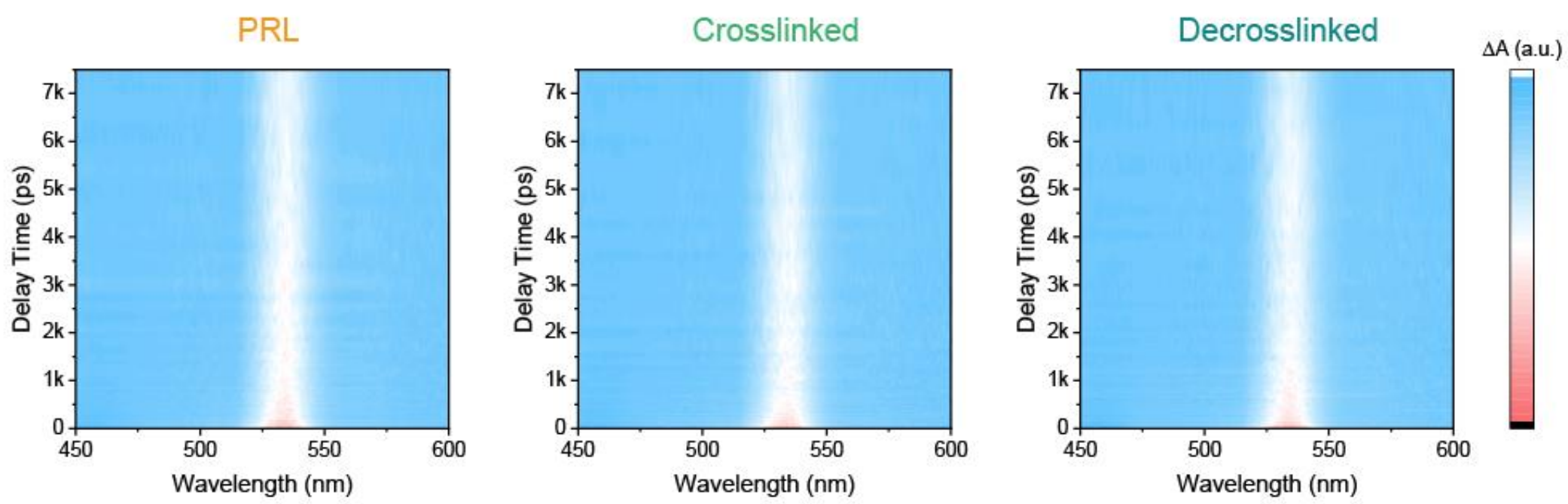

Supplementary Fig. 13 2D pseudo-color fs-TA plots of PRL-GQD (left), crosslinked PRL-GQD (middle) and decrosslinked PRL-GQD (right).

Comment: *4. The DFT section also appears inconsistent: the equation in the Supplementary Information describes molecular stacking rather than ligand adsorption on a ZnS surface, so it cannot directly support the claim that PRL binds more strongly to ZnS than OA.*

Response:

Dear reviewer, we apologize for the error in the presentation of the Binding Energy calculation formula in the Supplementary Information of our original manuscript. The correct expression should be "$E_b = E_{System} - E_{Molecule} - E_{Surface}$". We wish to emphasize that this is a simple writing error, and all data and conclusions in the work were obtained using the correct formula. We are grateful to you for your careful review.

Comment: *5. The process relies on crosslinking to make the QD film insoluble for development, followed by decrosslinking to return the QDs toward their original ligand state. The supplementary information further states that decrosslinked PRL-QDs can be immersed in n-octane or toluene and removed, enabling QD recycling or subsequent patterning. This raises a fundamental process question: if the final QD pattern is decrosslinked, can it survive subsequent spin-coating and development steps during RGB fabrication?*

Response:

Dear reviewer, thanks for raising such an interesting and important question. Encouragingly, **our reversible photolithography strategy is fully compatible with the fabrication of multi-color QD patterns**. As shown in Figure R17a, multicolor QD patterns—for example, overlays of RGB pixels—are prepared through in situ photo-induced crosslinking and developing. In this case, no mutual erosion occurs between QD patterns, because the patterned QDs remain in the crosslinked state. Subsequently, a DUV-induced decrosslinking step is performed to restore the pristine ligand structure.

As a proof of concept, we successfully fabricated corresponding red (R), green (G), and blue (B) QD pixel arrays (Figure R17b). Furthermore, the vertical stacking of these QD patterns also generates three secondary colors—cyan, orange, and pink (Figure R17c). Meanwhile, all QD pixels exhibited well-defined shapes with clear boundaries, confirming that no mutual erosion occurred during the multiple reversible photolithography cycles. This result fully alleviates the concern about the incompatibility of multicolor QD integration.

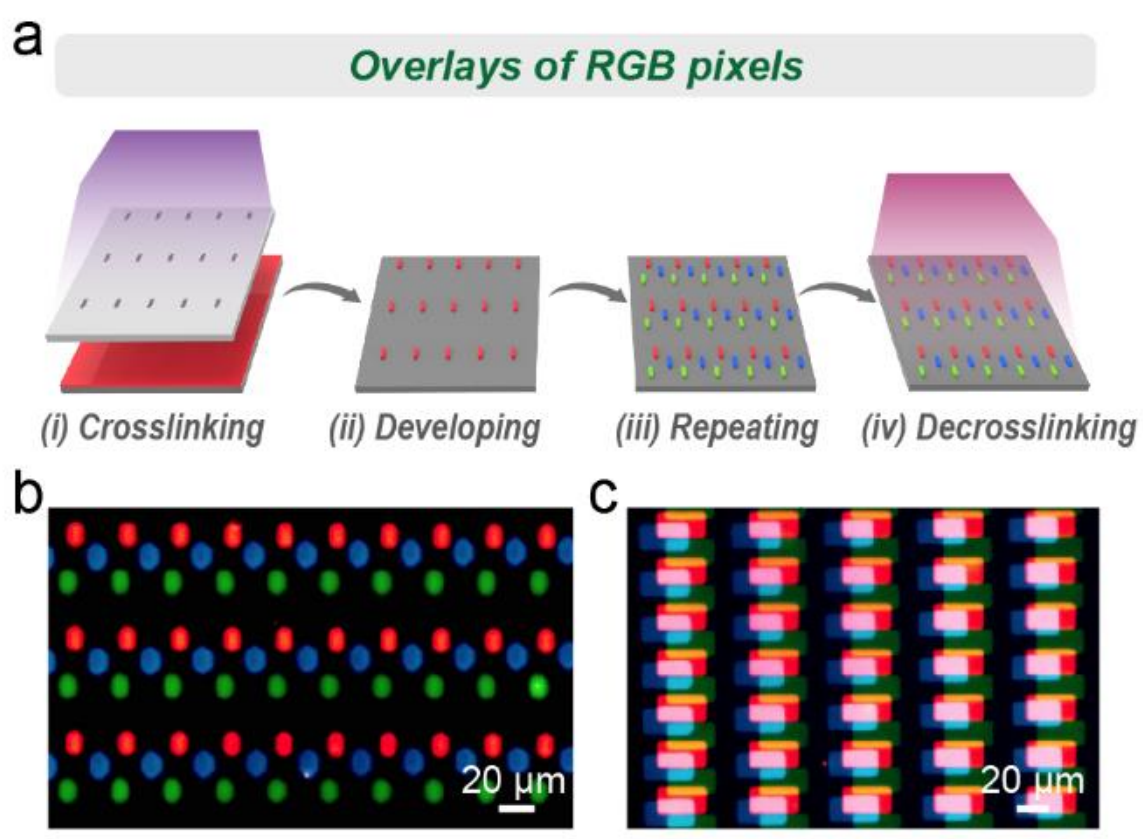

Figure R17. (a) Schematic of overlays of RGB pixels through reversible photolithography. (b) Fluorescent microscopic image of lateral overlays of RGB pixels. (c) Fluorescent microscopic image of vertical stacking of RGB pixels.

Change made on the third paragraph of Page 13: "Encouragingly, the reversible photolithography strategy remains fully compatible with multicolor QD integration. As shown in **Figure 5g**, multicolor QD patterns—for example, overlays of RGB pixels—are prepared through in-situ photo-induced crosslinking and developing. During this process, no mutual erosion occurs, because the pre-patterned QDs remain in the crosslinked state. Subsequently, a DUV-induced decrosslinking step restores the pristine ligand states of all QD pixels. As a result, we successfully fabricated RGB QD pixel arrays on the same substrate (**Figure 5g**). Furthermore, the vertical stacking of RGB pixels generated three secondary colors—cyan, orange, and pink—while maintaining well-defined pixel shapes and clear boundaries. These results lay a solid foundation for future full-color display applications."

Change made on Page 14:

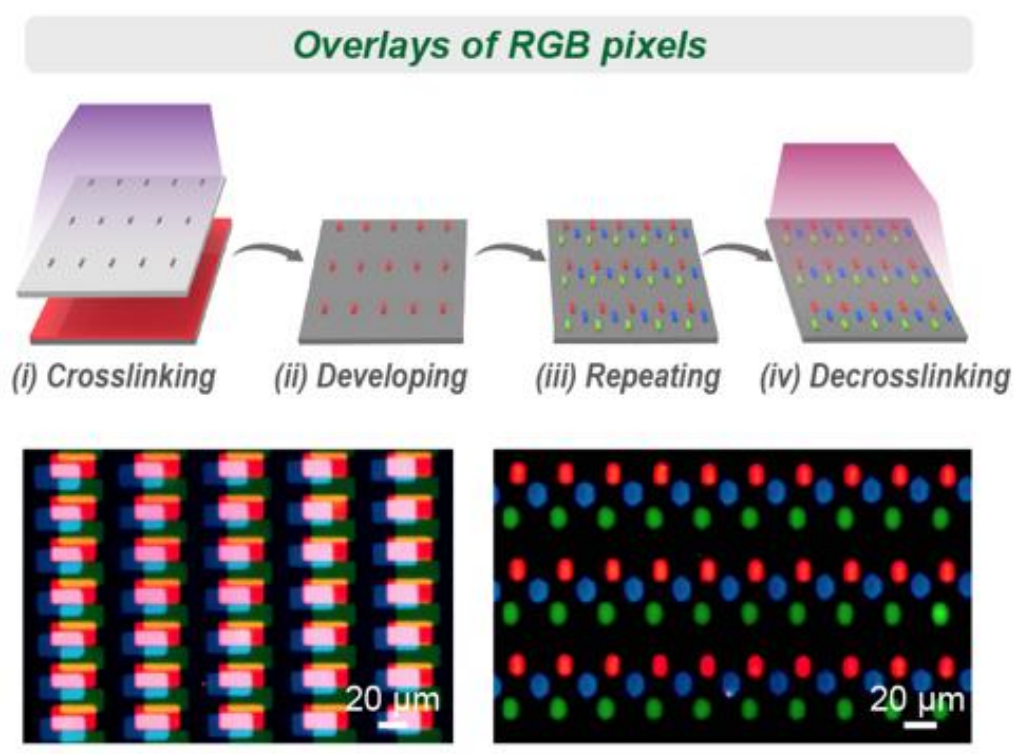

Figure 5 (g) Top: Schematic of overlays of RGB pixels through reversible photolithography. Bottom: Fluorescent microscopic images of overlays of RGB pixels.

Comment: *6. For a "reversible photolithography platform," the manuscript currently demonstrates approximately one crosslinking–decrosslinking cycle. Coumarin photocleavage under 254 nm irradiation may be incomplete and can be accompanied by fatigue or irreversible side reactions. The authors should provide multi-cycle data over at least 3–5 crosslinking/decrosslinking cycles.*

Response:

Dear reviewer, we sincerely appreciate your deep understanding of this field.

Indeed, as you rightly pointed out, **the photocleavage (decrosslinking) and photodimerization (crosslinking) reactions of coumarin derivatives are in a dynamic equilibrium under 254 nm light irradiation, along with potential fatigue or irreversible side reactions** [Ref: Adv. Mater. 2021, 33, 2008057; Polymer 2012, 53, 2691; ACS Appl. Mater. Interfaces 2018, 10, 16793]. Improving the photocleavage efficiency of coumarin derivatives has been explored through single- and two-photon absorption [Ref: J. Photochem. Photobiol., A 2008, 197, 239], solvent dependence [J. Chem. Soc., Perkin Trans. 1 1983, 1, 1083], and functional group modification [Ref: J. Phys. Chem. A 2018, 122, 7587; Chem. Phys. Lett. 2013, 588, 91].

Here, we would like to emphasize that **the core contribution of this work lies in the conceptual innovation of a reversible photolithography strategy to address the performance degradation inherent in conventional direct QD photolithography**. The coumarin-based photo-reversible ligand is employed here as a demonstrative route to validate the feasibility of the concept, rather than as the sole embodiment of the strategy. Importantly, this technical route has already yielded compelling results. We look forward to further optimizing the molecular structure and QD photosensitive material system to achieve more ideal photolithography outcomes in future work. Corresponding discussion has been incorporated into the revised manuscript.

Change made on the second paragraph of Page 11: "A minor drawback is that the EL recovery is not 100%, due to the reversible photocleavage equilibrium of coumarin groups under 254 nm irradiation.[59] And we hope to overcome this by further molecular and system optimization in the future."

Change made on Page 20: "[59] Zhu, C. N. et al. Reconstructable gradient structures and reprogrammable 3D deformations of hydrogels with coumarin units as the photolabile crosslinks. *Adv. Mater.* **33**, 2008057 (2021)."

On the other hand, following your valuable suggestion, **we have also monitored the multiple cycling behavior of the photosensitive QD films (PRL-GQD) under ambient conditions by alternating 310 nm and 254 nm UV exposure to sequentially induce crosslinking and decrosslinking reactions** (Figure R18). After each crosslinking

step, the PRL-GQD film was developed with toluene. The results show that even after six cycles, the QD films could still be effectively crosslinked (cured), while maintaining ~74% of the initial PL QY. The gradual decrease in PL QY over cycling is most likely attributable to UV-induced degradation of QDs assisted by water and oxygen, as well as the fatigue or irreversible side reactions as you mentioned. We also note that the crosslinked state consistently exhibits higher PL QY than the decrosslinked state, which may be attributed to the increased inter-QD spacing induced by crosslinking. Collectively, these results demonstrate that **our reversible photolithography system holds promising cycling potential, even under ambient conditions**.

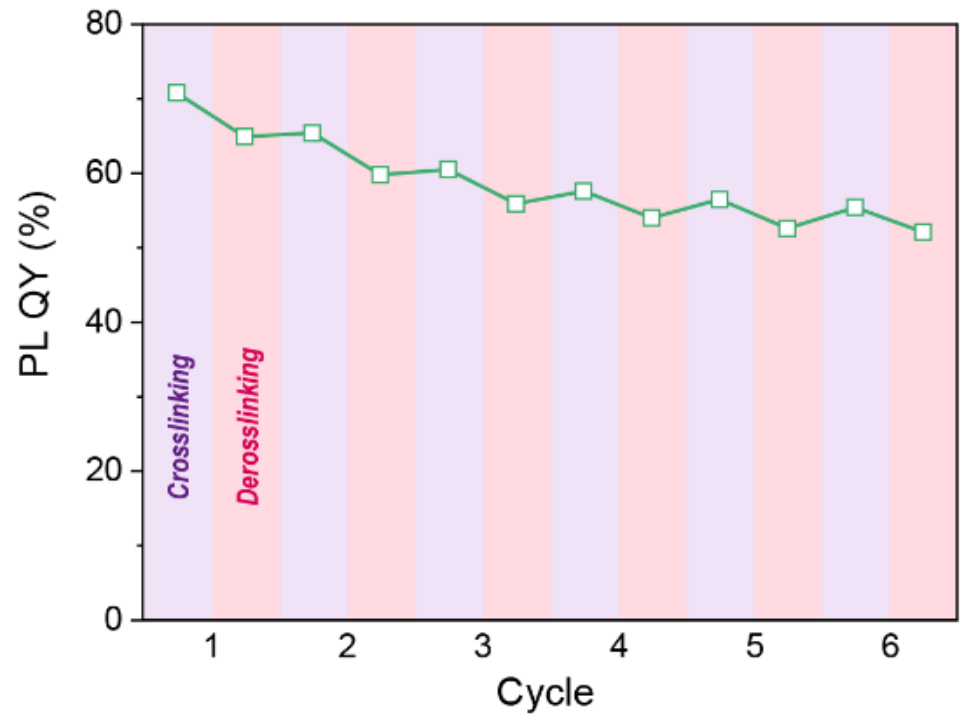

Figure R18. PL QYs evolution of the PRL-GQD film during cyclic crosslinking and decrosslinking processes. After each crosslinking step, the film was developed with toluene. All operations were carried out under ambient conditions.

Change made on the first paragraph of Page 13: "Furthermore, the reversible photolithography process can be cycled more than six times (Supplementary Fig. 17)."

Change made on Page 10 in the revised Supplementary Information:

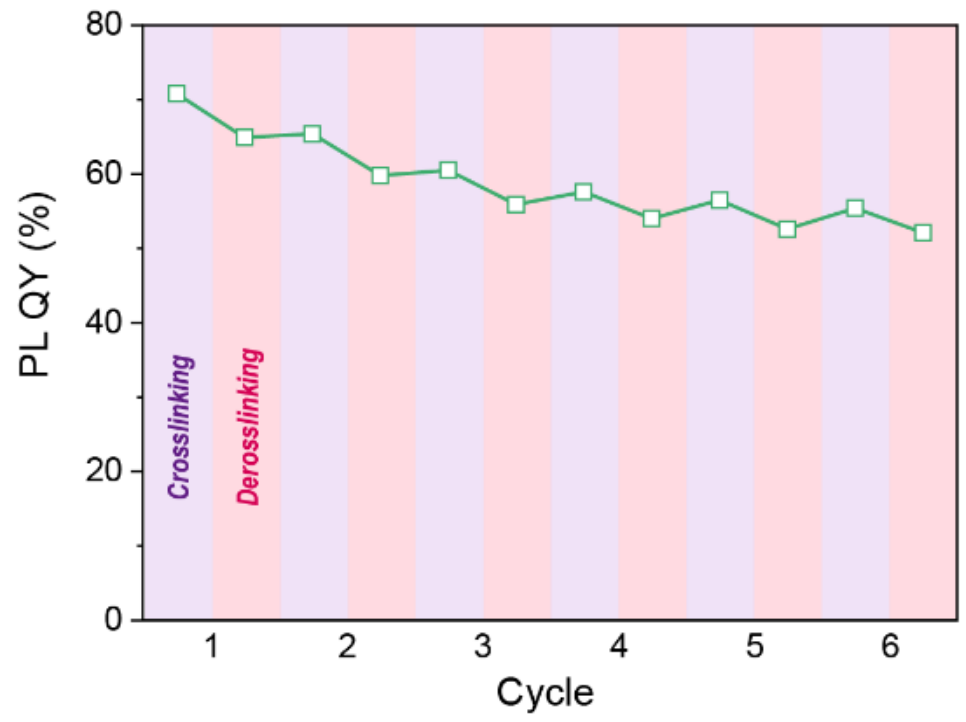

Supplementary Fig. 17 PL QYs evolution of the PRL-GQD film during cyclic crosslinking and decrosslinking processes. After each crosslinking step, the film was developed with toluene. All operations were carried out under ambient conditions.

Notably, despite a gradual decrease in overall PL QY, the crosslinked state consistently shows higher PL QY than the decrosslinked state, which may be attributed to the increased inter-QD spacing induced by crosslinking.

Comment: *7. The RGB demonstration mainly consists of PL images and CIE coordinates. Without RGB QLED devices or sequential full-color process validation, the manuscript should avoid implying complete full-color QLED display fabrication.*

Response:

Dear reviewer, thanks a lot for your time and efforts to review our manuscript. The reversible photolithography strategy proposed in this work is fully compatible with the fabrication of multicolor QD patterns for achieving full-color displays. The corresponding experimental results and discussion have been presented in our response to your previous comment.

Thank you again for your time and careful review of our manuscript. We sincerely believe that the manuscript has greatly benefited from your valuable suggestions.

To reviewer 4

Comment: *The authors propose a novel reversible photolithography strategy based on reversible photo-crosslinkable coumarin ligand and address the core bottleneck of QD performance degradation during direct photolithography patterning. The design is rigorous. The demonstrated device performance (EQE of 24.41%, resolution of 21,000 PPI) represents state of the art results in the field. This work will provide innovative insights into micro/nanofabrication for high-performance QD devices. Therefore, I recommend acceptance after minor revisions.*

1.The crosslinking/decrosslinking procedure is not clearly described. In particular, it is ambiguous whether development is required after the decrosslinking step. Please provide a complete, step by step operational flowchart.

Response:

Dear reviewer, thanks a lot for your time and efforts to review our manuscript. We also appreciate your positive recognition, particularly that "*this work will provide innovative insights into micro/nanofabrication for high-performance QD devices*". We believe that with the help of you and other experts, the quality of our work has been significantly improved.

We thank the reviewer for the constructive suggestion to provide a clear description for better understanding. The designed reversible photolithography involves the following steps: (i) spin-coating of photosensitive QD solution onto a substrate (e.g., the cleaned silicon) at 2000 rpm; (ii) exposure to long-wavelength UV light (centered at 310 nm) with photomasks for required crosslinked QD patterns; (iii) developing with n-octane or toluene to wash off the un-crosslinked components; and (iv) exposure to short-wavelength UV light (centered at 254 nm) to trigger decrosslinking. It should be noted that the reversible photolithography process can be conducted either under ambient conditions or in a nitrogen-filled glove box. Unless otherwise stated, all experiments were performed in a nitrogen-filled glove box by default.

Change made on Page 16 (Method):

"The reversible photolithography is illustrated with PRL-GQD (Supplementary Fig. 3):

(i) The photo-induced crosslinking process: at first, the solution of PRL-GQD was coated on a substrate (e.g., the cleaned silicon) at 2000 rpm to form a PRL-GQD film. And then the film was exposed to UV irradiation (centered at 310 nm) with photomasks for required patterns. After UV exposure, the PRL-GQD film was immersed in toluene for 10 s to wash off the un-crosslinked areas. And the crosslinked PRL-GQD film or pattern was obtained successfully.

(ii) The photo-induced decrosslinking process: the crosslinked PRL-GQD film or pattern was exposed to DUV irradiation (centered at 254 nm). In this process, the crosslinked PRL-GQD will return to their initial dispersed state.

Note: The reversible photolithography process can be conducted either under ambient conditions or in a nitrogen-filled glove box. Unless otherwise stated, all experiments were performed in a nitrogen-filled glove box by default."

Change made on Page 3 in the revised Supplementary Information:

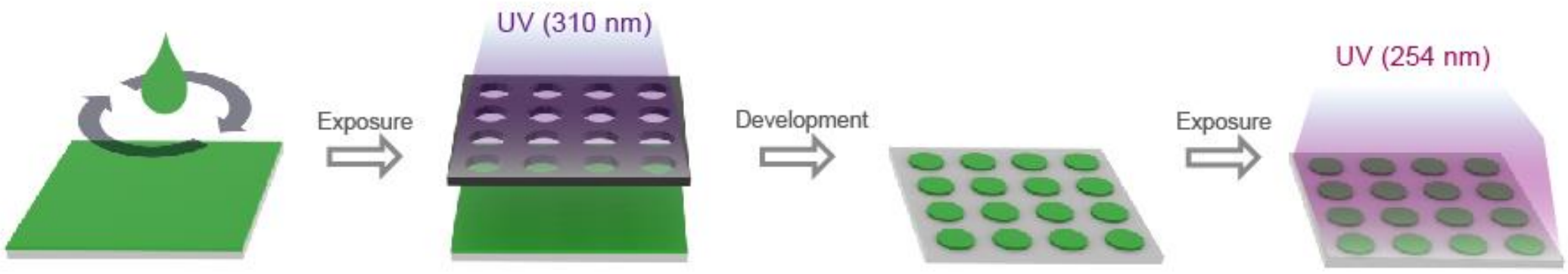

Supplementary Fig. 3 Schematic of reversible photolithography of QDs.

Comment: *2.As shown in Figure 3b, PL intensity is used as the sole metric to evaluate crosslinking and decrosslinking, which is an indirect method. I suggest supplementing these data with direct measurements of film thickness retention to more accurately monitor the changes in the QD films.*

Response:

Dear reviewer, we thank you for your constructive suggestion. In response, we performed atomic force microscopy (AFM) measurements to directly characterize the film thickness of QDs under different exposure doses. As shown in Figure R19, during the crosslinking process, the film thickness gradually increased with rising exposure dose and eventually reached a plateau. Notably, at an ultralow exposure dose of ~1 mJ cm^{-2}, the film thickness retention reached 58.8%. Upon subsequent decrosslinking, the film thickness (after development) gradually decreased. Importantly, these thickness results are highly consistent with the PL intensity trends (indirect evidence, Figure 3b), further confirming the reliability of the reversible photolithography process. Following your suggestion, we have incorporated the relevant thickness data and corresponding discussion into the revised manuscript and the Supplementary Information.

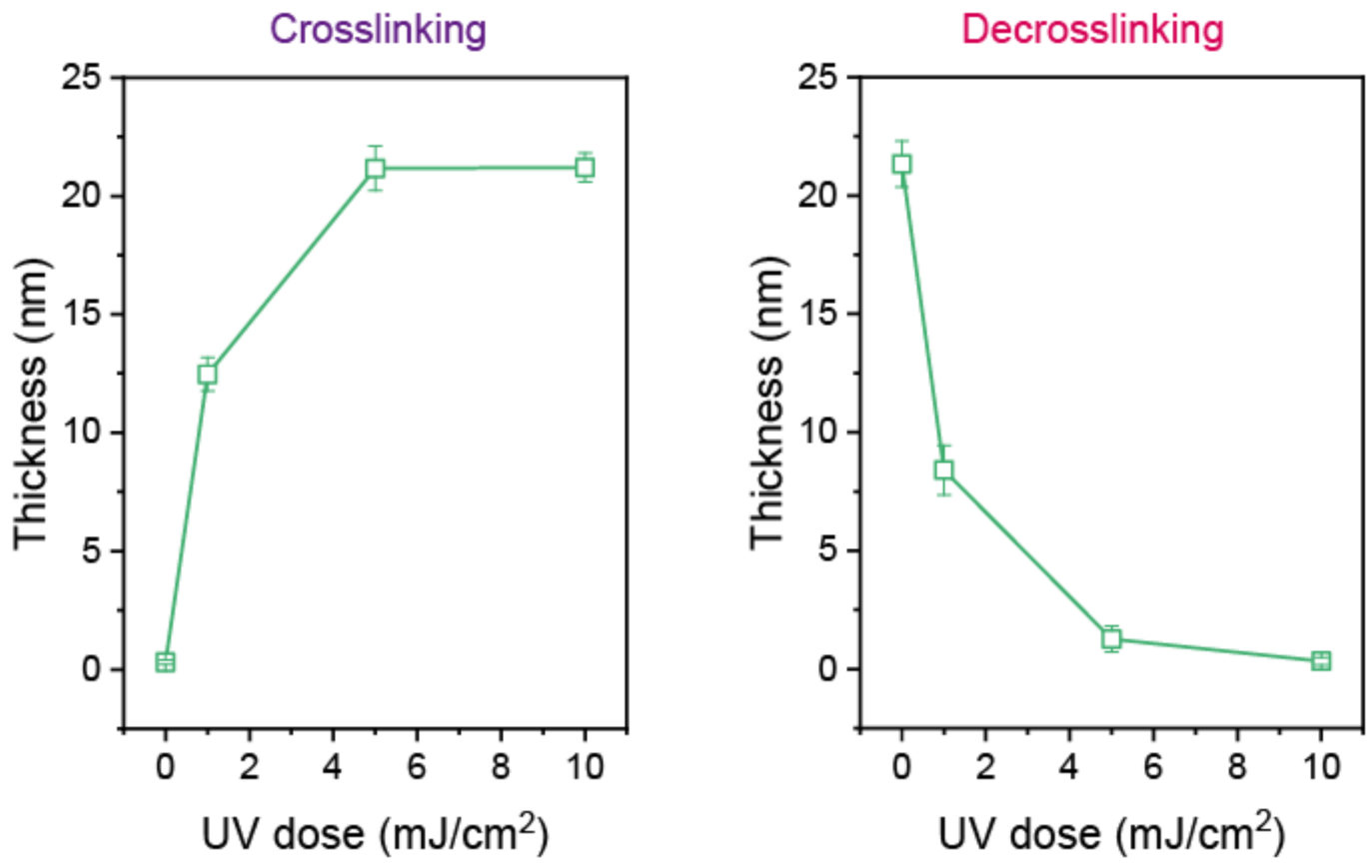

Figure R19. Film thickness of PRL-GQD films under the crosslinking process (left) and the decrosslinking process (right) with different UV doses. UV lamps (310 nm, ~1 mW/cm^2 for the crosslinking process; 254 nm, ~1 mW/cm^2 for the decrosslinking process) and toluene development are used here. Three independent samples were measured for each parameter setting.

Change made on the first paragraph of Page 7: "Moreover, we performed atomic force microscopy (AFM) measurements to directly characterize the film thickness of PRL-GQD under different exposure doses. As shown in Supplementary Fig. 4, the resulting thickness data are highly consistent with the PL intensity trends, further confirming the reliability of the reversible photolithography process."

Change made on Page 3 in the revised Supplementary Information:

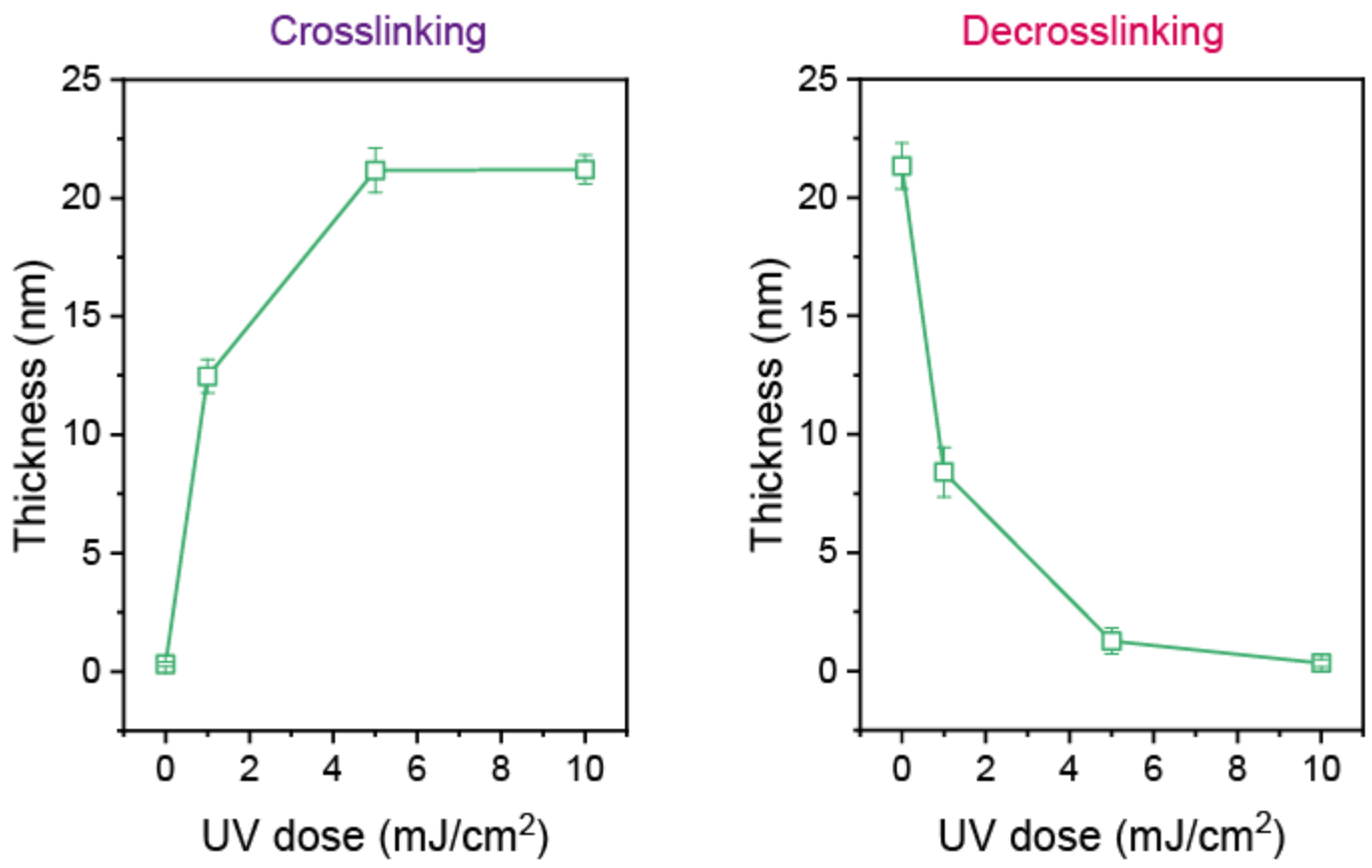

Supplementary Fig. 4 Film thickness of PRL-GQD films under the crosslinking process (left) and the decrosslinking process (right) with different UV doses. UV lamps (310 nm, ~1 mW/cm^2 for the crosslinking process; 254 nm, ~1 mW/cm^2 for the decrosslinking process) and toluene development are used here. Three independent samples were measured for each parameter setting.

Comment: *3.The "Pristine" device used for comparison is based on PRL-capped QDs, but the ligand exchange itself is already part of the photolithographic workflow. To provide a fair baseline, it would be valuable to include a reference device using the original QDs (without any ligand exchange or photo-treatment).*

Response:

Dear reviewer, thanks for your meaningful comment. In our original manuscript, we used QLEDs fabricated from PRL-tailored green-emitting QDs (termed PRL-GQD) as the "pristine" group. This choice was intended to provide a clear basis for tracking performance changes throughout the photo-crosslinking/decrosslinking process, thereby highlighting the effectiveness of our strategy in overcoming conventional performance degradation.

We also fully agree with your suggestion that a fairer baseline could be established by including QLEDs based on pristine oleic acid-capped QDs (termed OA-GQD). **In response, we fabricated the corresponding devices and characterized their electroluminescence performance**. As shown in Figure R20, these devices exhibited a current density of 87.5 mA cm^{-2} and a luminance of 93,801 cd m^{-2} at 5.0 V, with a peak EQE of only 23.53%. These values are significantly lower than those of the devices after reversible photolithography (e.g., 114.7 mA/cm^2 and 125,016 cd m^{-2} at 5.0 V, a peak EQE of 24.41%). We attribute this improvement after reversible photolithography to the efficient passivation effect of PRL, which has been comprehensively demonstrated in our original manuscript.

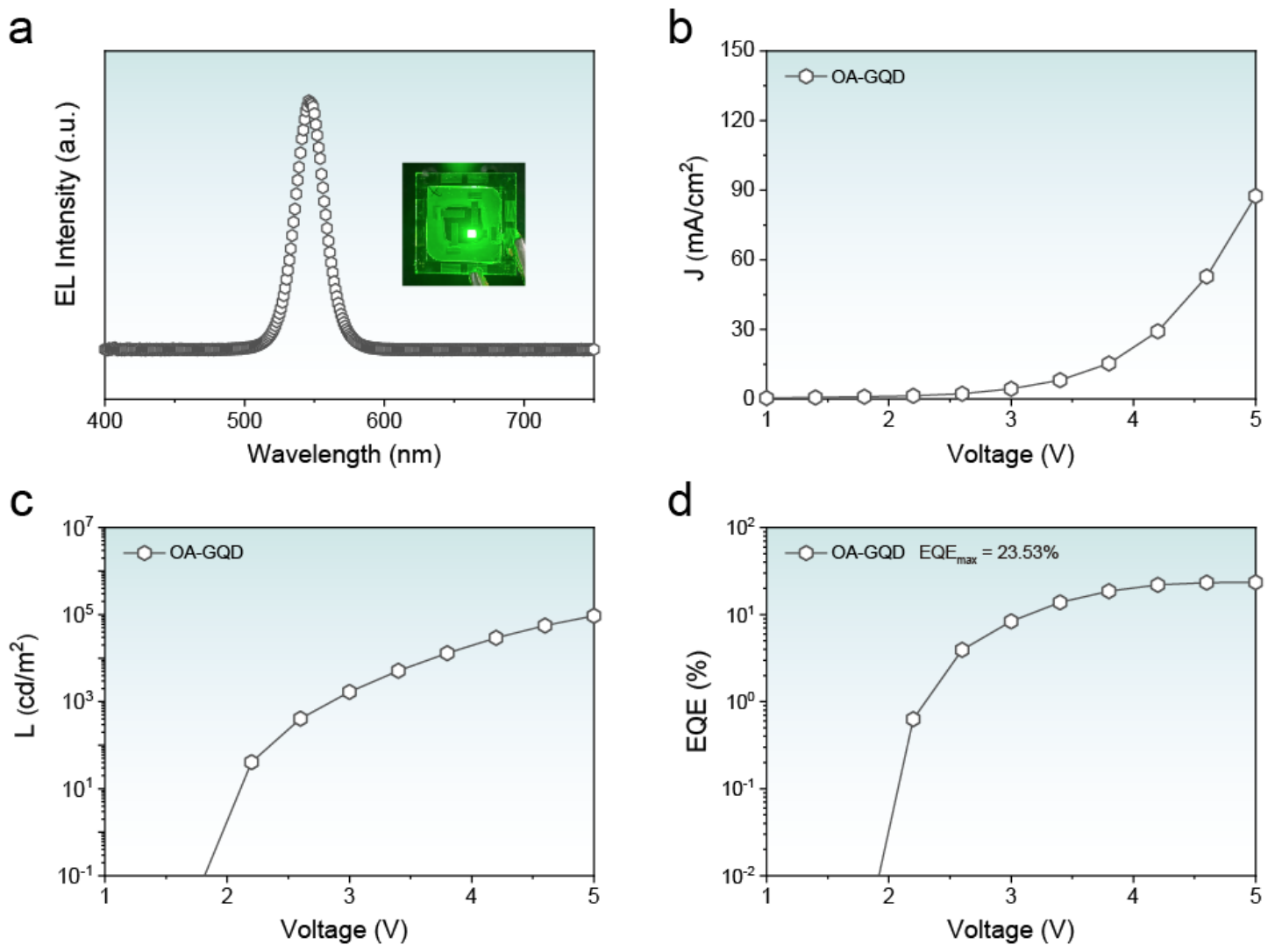

Figure R20. Electroluminescence performance of the QLED device based on OA-GQD. (a-d) Normalized EL spectra (a), *J–V* curves (b), *L–V* curves (c) and EQE–*V* curves (d) of QLEDs based on OA-GQD. Insert in (a): EL photo of corresponding QLED at 5.0 V.

In a word, by using OA-GQD as a standard reference, we demonstrate that **our reversible photolithography strategy not only enables effective photo-patterning but also yields positive enhancement in the electroluminescence performance**. This further underscores the promising potential of our approach for achieving high-performance electroluminescence. We sincerely thank you again for this valuable suggestion. We believe that the additional experimental data will further reinforce the central conclusions of our work and enhance the overall quality of the manuscript.

Change made on the third paragraph of Page 11: "In addition, we also fabricated QLEDs based on pristine OA-GQD and characterized their EL performance. As shown in Supplementary Fig. 15, these devices exhibited a current density of 87.5 mA cm^{-2} and a luminance of 93,801 cd m^{-2} at 5.0 V, with a peak EQE of only 23.53%. These values are markedly lower than those of the decrosslinked PRL-GQD devices."

Change made on Page 9 in the revised Supplementary Information:

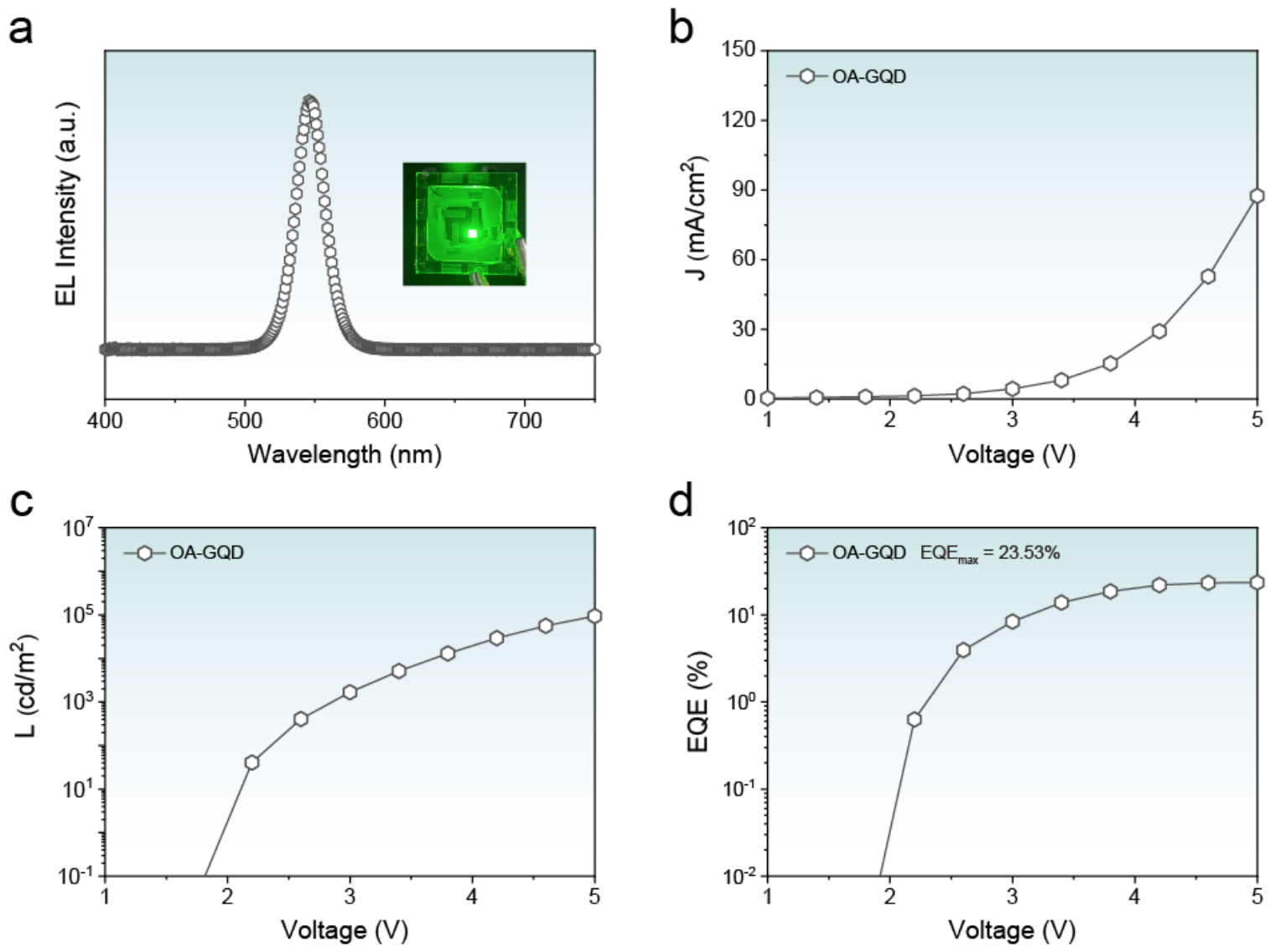

Supplementary Fig. 15 EL performance of the QLED device based on OA-GQD. (a-d) Normalized EL spectra (a), *J–V* curves (b), *L–V* curves (c) and EQE–*V* curves (d) of QLEDs based on OA-GQD. Insert in (a): EL photo of corresponding QLED at 5.0 V.

Comment: *4.The authors mention that pixelated QLEDs suffer from pronounced leakage currents. I encourage expanding the discussion on potential device architecture optimizations to mitigate this issue. Notably, a recent study (doi: 10.1038/s41586-026-10333-w) addresses non-uniform electric field distribution in pixelated QLEDs; including this reference would strengthen the practical outlook of the work.*

Response:

We sincerely thank the reviewer for the highly insightful comments and for the constructive expectations regarding the enhancement of our manuscript.

We fully agree that QLED devices based on patterned/pixelated QDs generally suffer from severe leakage current, primarily due to direct HTL/ETL contact in non-patterned regions. Consequently, research in this field has predominantly focused on non-pixelated devices, as summarized in a recent review [Ref: Chem. Rev. 2026, 126, 3624]. To address this issue, device architecture optimizations have been widely explored. The most representative approach involves the deposition of an insulating polymethyl methacrylate (PMMA) layer over the QD pixels, which has been validated in both our group and others [Ref: Nano Res. 2026, DOI: 10.26599/NR.2026.94908915; Light Sci. Appl. 2025, 14, 251; Adv. Funct. Mater. 2025, 35, 2504261; Nano Lett. 2024, 24, 1254]. Recently, Some other materials, such as Nigrosin [Ref: Adv. Funct. Mater. 2025, 35, 2510076] and perfluorodecyltriethoxysilane [Ref: Light Sci. Appl. 2026, 15, 157], have also been employed for the same purpose.

Another common challenge in pixelated QLEDs is the significant drop in EQE as the pixel size decreases [Ref: Light Sci. Appl. 2025, 14, 370]. Recently, Li et al. attributed this degradation to electric-field non-uniformity arising from pixel microstructures and successfully overcame it by modulating the dielectric constant using TiO_2 nanoparticles [Ref: Nature 2026, 652, 349]. We have incorporated the relevant discussion into the revised manuscript.

Change made on the second paragraph of Page 15: "Consequently, research in this field has predominantly focused on non-pixelated devices.[29] To address this issue, multiple charge-blocking layers have been widely explored, such as polymethyl methacrylate (PMMA),[34,62-64] polyvinyl alcohol,[1] nigrosin,[65] and perfluorodecyltriethoxysilane.[66]"

Change made on the third paragraph of Page 15: "the peak EQE of the nano-pixelated devices is slightly lower than that of the non-patterned ones, likely due to the non-ideal pixel morphology at such a high resolution and the electric-field non-uniformity from pixel microstructures,[1]"

Change made on Page 21: "[62] Tan, H., Zhai, Z., Li, C., Chen, K., Wang, W., Gu, C., Xiang, C. & Zhang, T. Robust and ultralow-energy direct photolithography of quantum dots via dual-ligand passivated thiol-ene click chemistry. Nano Res. DOI: 10.26599/NR.2026.94908915 (2026).

[63] Huo, Y. et al. Ambient direct lithography patterning of ultra-stable perovskite quantum dots for high-resolution light-emitting diodes. Adv. Funct. Mater. 35, 2504261 (2025).

[64] Qie, Y. et al. Ligand-nondestructive direct photolithography assisted by semiconductor polymer cross-linking for high-resolution quantum dot light-emitting diodes. Nano Lett. 24, 1254–1260 (2024)."

In our original work, we successfully fabricated ultrahigh-resolution nano-pixelated QLED devices (pixel size: ~ 0.8 μm, 21,000 PPI) using the reversible photolithography strategy. Building on this, **we further integrated the classical PMMA approach to suppress inter-pixel leakage current**. Electroluminescence measurements revealed that the device after decrosslinking exhibited improved *L* (49,292 vs. 28,769 cd/m^2), *J* (65.6 vs. 40.7 mA/cm^2), and peak EQE (17.08% vs. 16.02%), as shown in Figures R21. It is consistent with the performance trends observed in non-patterned devices (Figure 4 in the original/revised manuscript).

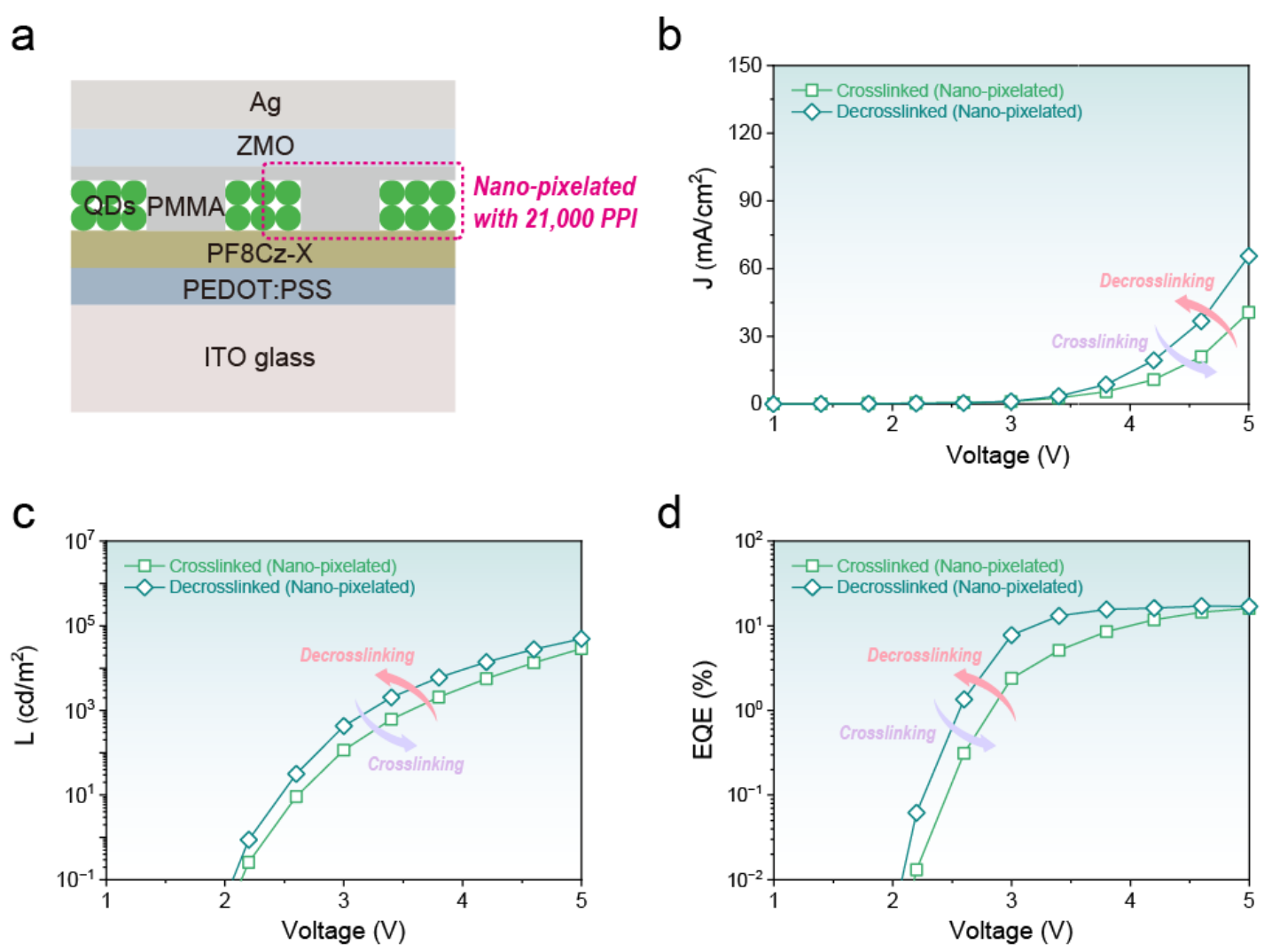

Figure R21. Electroluminescence performance of nano-pixelated QLED devices at 21,000 PPI. (a) Schematic of nano-pixelated QLED with a PMMA layer. (b-d) Normalized EL spectra (a), *J*–*V* curves (b), *L*–*V* curves (c) and EQE–*V* curves (d) of QLEDs based on OA-GQD. Insert in (a): EL photos of corresponding QLED at 5.0 V.

These results further underscore the effectiveness of our reversible photolithography strategy in simultaneously achieving high-resolution patterning and high device performance. Notably, **the peak EQE of 17.08% at 21,000 PPI represents one of the highest values in the field** (Figure R22). The corresponding experimental data and discussion have been included in the revised manuscript and Supplementary Information.

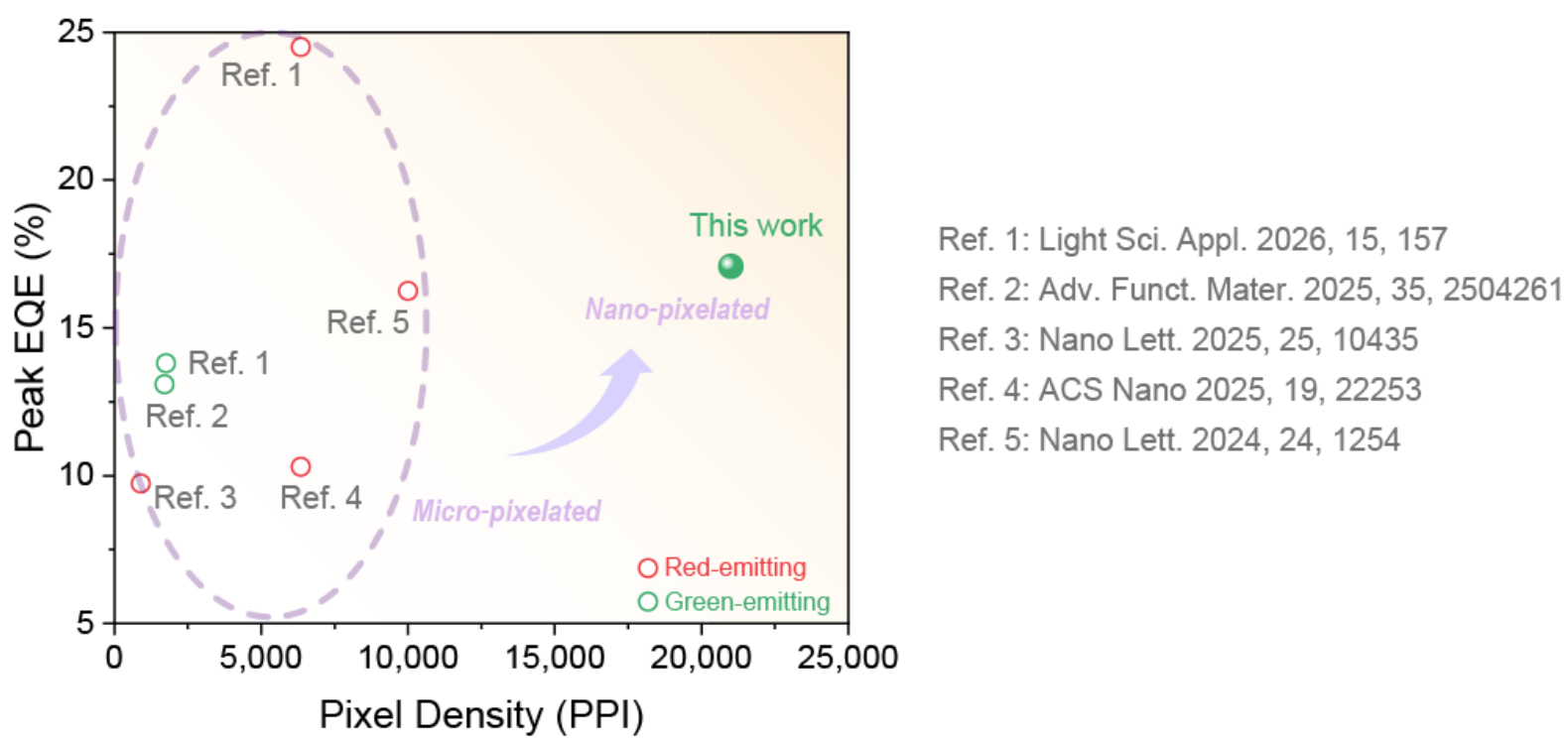

Figure R22. Comparation of different photolithographic QLEDs in pixel density and peak EQE.

Change made on the last paragraph of Page 15: "Here, we introduced a PMMA layer in as-fabricated ultrahigh-resolution nano-pixelated QLEDs with 21,000 PPI. EL measurements revealed that the device after decrosslinking exhibited improved *L* (49,292 vs. 28,769 cd/m^2), *J* (65.6 vs. 40.7 mA/cm^2), and peak EQE (17.08% vs. 16.02%), as shown in **Figure 5i** and Supplementary Fig. 24, which is consistent with the performance trends observed in non-patterned devices. While the peak EQE of the nano-pixelated devices is slightly lower than that of the non-patterned ones, likely due to the non-ideal pixel morphology at such a high resolution and the electric-field non-uniformity from pixel microstructures,[1] this value (17.08% at 21,000 PPI) remains highly competitive (Supplementary Fig. 25 and Table 2). These results further underscore the effectiveness of our reversible photolithography strategy in simultaneously achieving high resolution and high device performance."

Change made on Page 14:

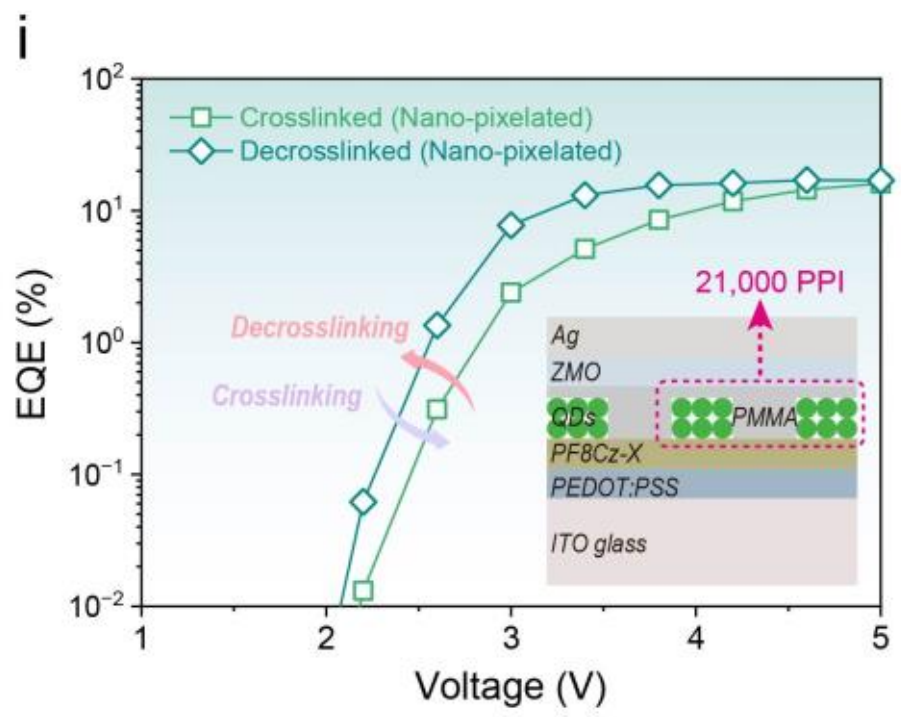

Figure 5i. EQE–*V* curves of nano-pixelated QLEDs based on crosslinked PRL-GQD and decrosslinked PRL-GQD. Insert: schematic of nano-pixelated QLED with a PMMA layer.

Change made on Page 25 in the revised Supplementary Information: "For pixelated QLEDs, a PMMA blocking layer (0.5 mg/mL, 5000 rpm) was spin-coated on the pixelated QDs to prevent current leakage caused by the direct contact between the hole transport layer and electron transport layer."

Change made on Page 13 in the revised Supplementary Information:

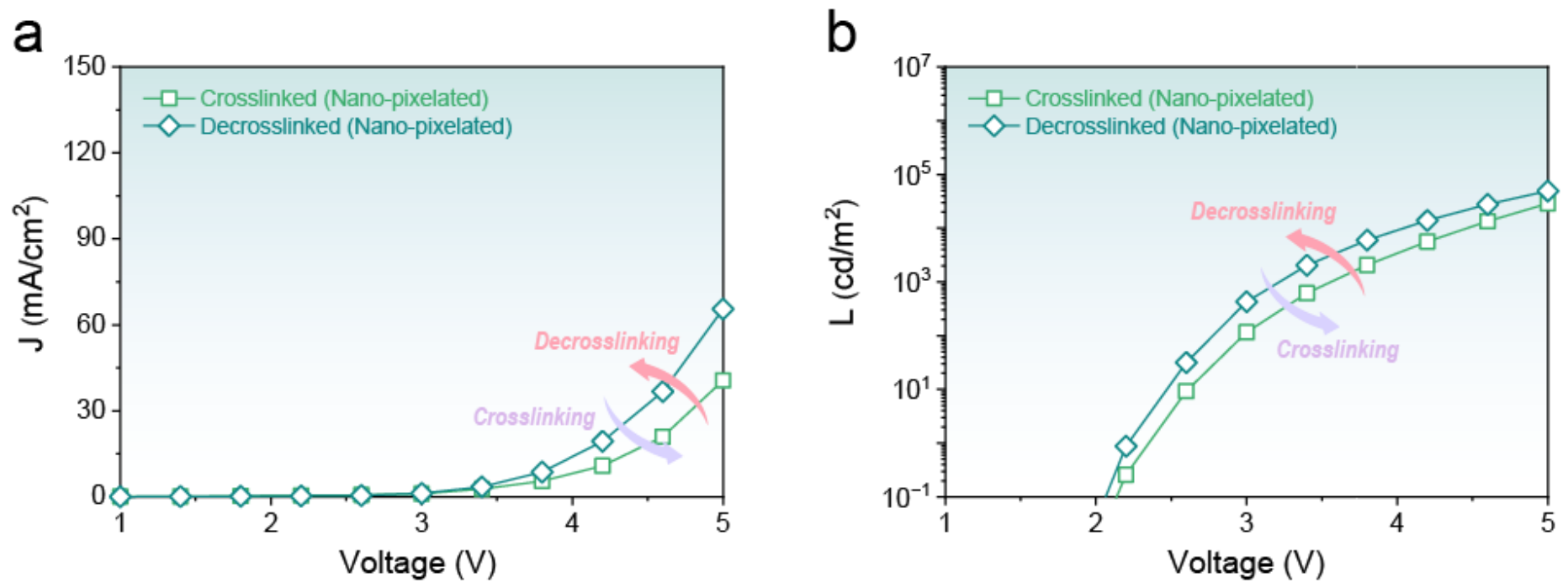

Supplementary Fig. 24 *J–V* curves (a) and *L–V* curves (b) of nano-pixelated QLEDs based on crosslinked PRL-GQD and decrosslinked PRL-GQD.

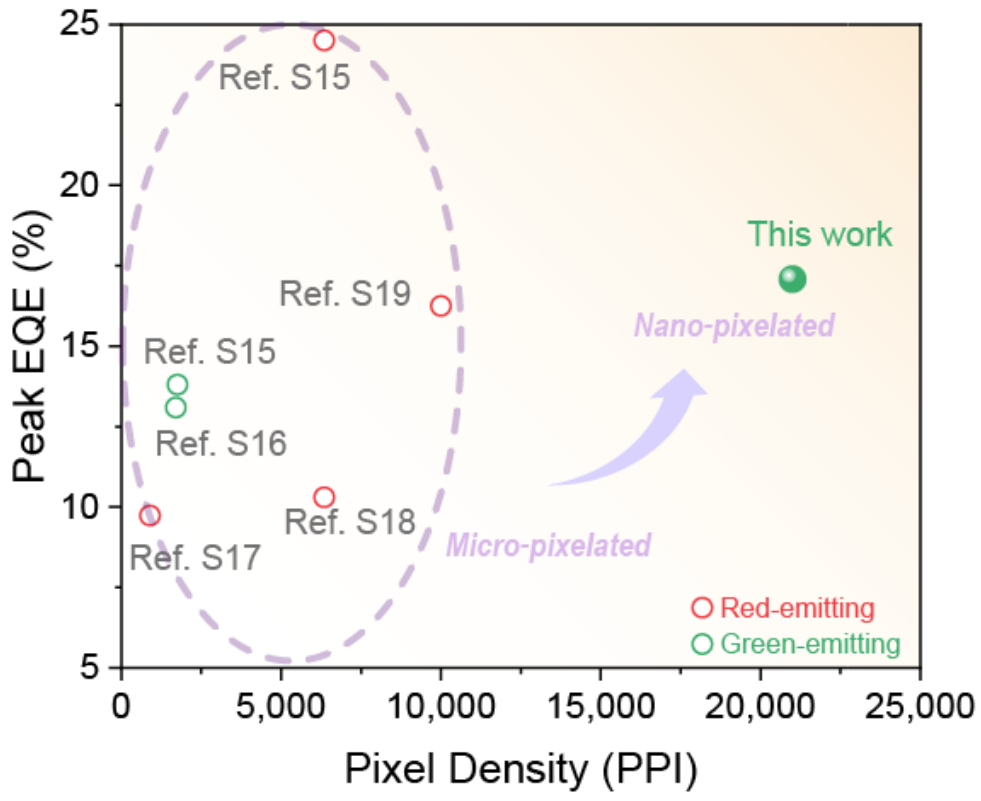

Supplementary Fig. 25 Comparation of different photolithographic QLEDs in pixel density and peak EQE.

Comment: *5.Although the decrosslinking step restores luminance and current density to 93% and 95% of the pristine values, respectively, the recovery is not complete (~100%). Why? In addition, they should avoid absolute terms such as "nondestructive" to maintain scientific rigor.*

Response:

Dear reviewer, we sincerely thank you for this insightful comment. We acknowledge that, complete recovery of electroluminescence performance has not yet been attained, despite the promising results achieved in this work. **We attribute this primarily to the fact that the photocleavage (decrosslinking) and photodimerization (crosslinking) reactions of coumarin derivatives are in a dynamic equilibrium under 254 nm light irradiation**, which prevents 100% restoration to the initial state. This phenomenon has been reported for many times [Ref: Adv. Mater. 2021, 33, 2008057; Polymer 2012, 53, 2691; ACS Appl. Mater. Interfaces 2018, 10, 16793]. It is also observable in our material system, as shown in Figure 2c in the original/revised manuscript, and further leads to incomplete restoration of conductivity, exciton binding energy, and electroluminescence performance.

In this case, **improving the photocleavage efficiency of coumarin derivatives is itself an important research topic**. Several strategies have been developed, including single- and two-photon absorption [Ref: J. Photochem. Photobiol., A 2008, 197, 239], solvent dependence [Ref: J. Chem. Soc., Perkin Trans. 1 1983, 1, 1083], and functional group modification [Ref: J. Phys. Chem. A 2018, 122, 7587; Chem. Phys. Lett. 2013, 588, 91]. We look forward to

optimizing the molecular structure and QD photosensitive material system to further achieve ideal photolithography outcomes. Corresponding discussion has been added in the revised manuscript.

Change made on the second paragraph of Page 11: "A minor drawback is that the EL recovery is not 100%, due to the reversible photocleavage equilibrium of coumarin groups under 254 nm irradiation.[59] And we hope to overcome this by further molecular and system optimization in the future."

Change made on Page 20: "[59] Zhu, C. N. et al. Reconstructable gradient structures and reprogrammable 3D deformations of hydrogels with coumarin units as the photolabile crosslinks. *Adv. Mater.* **33**, 2008057 (2021)."

Finally, we would like to emphasize that the core contribution of this work lies in **the conceptual innovation of a reversible photolithography strategy to address the performance degradation inherent in conventional direct QD photolithography**. This strategy represents a significant advancement over previous technical frameworks. The coumarin-based photosensitive ligand is employed here as a demonstrative route to validate the feasibility of the concept, rather than as the sole embodiment of the strategy. Importantly, this technical route has already yielded compelling results, particularly in device performance compared to pristine OA-capped GQDs and in nano-pixelated QLED devices. We therefore believe that the novelty and technical significance of our work merit due recognition.

By the way, following your suggestion, **we have removed the term "nondestructive" and revised the corresponding descriptions throughout the manuscript**.

Comment: *6.Several minor grammatical issues need correction, for example, "systemically" should be "systematically" (page 9), "through a ... lamp" should be "with a ... lamp" (page 11), and "representing among" should be "representing one of" or "which is among" (page 12).*

Response:

Dear reviewer, we thank you for pointing out the formatting issues in our manuscript. We have systematically reviewed the entire manuscript and corrected the relevant issues accordingly.

Reversible photolithography of quantum dots achieves high-performance electroluminescence

(Manuscript ID: NCOMMS-26-047874A)

A Point-by-point Response to the Reviewers' Comments

To reviewer 1

Comment: *All of my concerns have been addressed adequately. Therefore, the revised manuscript is suitable for publication.*

Response:

Dear reviewer, thanks a lot for your time and efforts to review our manuscript. We also appreciate your positive recognition. We believe that with the help of you and other experts, the quality of our work has been significantly improved.

To reviewer 2

Comment: *The manuscript has been substantially improved and is much clearer than the original version. I appreciate the authors' efforts in addressing the previous comments. Nevertheless, a few minor issues remain and should be addressed, as detailed below.*

1) In Figure 1, the term "optoelectronic performance degradation" used for the previous works may be misleading. Such degradation is not necessarily observed in all of the reported systems; some studies show degradation, whereas others do not. Therefore, the authors should reconsider this terminology and use a more neutral and accurate description.

Response:

Dear reviewer, we sincerely appreciate your time and thorough review of our manuscript. We are also grateful for your encouraging feedback on our revisions. To address the remaining issues you have kindly pointed out, we have made further revisions.

First, we thank the reviewer for this careful and constructive comment regarding the term "optoelectronic performance degradation" in Figure 1. We fully agree that this term might be misleading, as it implies that performance loss is universally observed in all reported systems. To address this concern, we have revised the term in Figure 1 to a more neutral and accurate phrasing: "**Typical performance variation**." The corresponding description in the manuscript has also been revised to further contextualize this variation.

Change made on the last paragraph of Page 3: "Existing direct photolithography of QDs, represented by the most promising ligand crosslinking approaches, typically induces variations in optoelectronic performance due to surface ligand changes caused by the inherent irreversibility of the photolithographic process. Moreover, in many representative studies in the field, such variations are often observed to result in performance degradation.[32–35,37]"

2) In Figure 1, the term "optoelectronic performance recovery" used for the present work in Figure 1 may also be misleading. The results presented here demonstrate recovery of the optoelectronic properties from the crosslinked state toward the precrosslinking state; they do not indicate that the optoelectronic performance is fully recovered

relative to the pristine, uncrosslinked state. In fact, the optoelectronic performance after crosslinking remains lower than that of the pristine, uncrosslinked material. Therefore, the authors should revise the terminology in Figure 1.

Response:

We also thank the reviewer for the precise and constructive comment regarding the term "optoelectronic performance recovery." We agree that this term may be misleading, as it could imply full restoration to the pristine (uncrosslinked) state. Considering that the optoelectronic performance of QDs after reversible photolithography, although significantly restored, remains slightly lower than that of the pristine QDs, we have revised the term in Figure 1 to "**Suppressed performance degradation**," which more accurately reflects our obtained results. The corresponding text in the manuscript has also been revised to avoid any potential misunderstanding.

Change made on the first paragraph of Page 4: "a photo-induced decrosslinking process is applied to restore the surface ligands of QDs and suppress optoelectronic performance degradation."

3) In Figure 2a, correct the typo "RRL".

Response:

Dear reviewer, thank you for your careful reminder. We have corrected the typo in Figure 2a.

4) In page 9, it is not clear on the sentence "The postcrosslinking reduction further reflects a compromised exciton binding capability, which ultimately impairs device performance, as confirmed by subsequent experiments". How about "The reduced Ea after crosslinking further reflects a compromised exciton binding capability, which ultimately impairs device performance, as confirmed by subsequent experiments."

Response:

We thank the reviewer for this kind reminder. To improve clarity, we have revised the corresponding description in the revised manuscript following your suggestion.

Change made on the third paragraph of Page 9: "the reduced E_a after crosslinking reflects a compromised exciton binding capability, which ultimately impairs device performance, as confirmed by subsequent experiments."

5) The description of the PMMA blocking layer fabrication in the Experimental section is not sufficiently clear. The authors state that "For pixelated QLEDs, a PMMA blocking layer (0.5 mg/mL, 5000 rpm) was spin-coated on the pixelated QDs to prevent current leakage caused by the direct contact between the hole transport layer and electron transport layer." However, the detailed procedure for forming and removing the PMMA blocking layer is not provided.

First, what solvent was used to dissolve the PMMA, and what were the concentration, spin-coating time, and subsequent drying or annealing conditions? More importantly, since PMMA was spin-coated directly onto the patterned QD layer, could the PMMA solution have partially covered or left residues on the QD surface? If so, how were such PMMA residues removed before deposition of the subsequent layers? Please clarify whether any solvent or washing process was used to selectively remove PMMA from the QD pixels while retaining PMMA in the blocking regions.

Because residual PMMA on the QD surface could potentially affect charge injection, interfacial contact, and ultimately the device performance, a more detailed description of the PMMA deposition and removal process is necessary. Please provide sufficient experimental details to clarify how the PMMA blocking layer was selectively formed without adversely affecting the patterned QD pixels.

Response:

Dear reviewer, we sincerely thank you for your time and efforts in reviewing our manuscript. In our previous manuscript, we introduced a PMMA layer as the blocking layer to prevent current leakage caused by direct contact between the hole transport layer (HTL) and the electron transport layer (ETL). This approach is a well-established strategy in the fabrication of pixelated QLED devices, and has been extensively described in the reported studies [Ref: Light Sci. Appl. 2025, 14, 251; Adv. Funct. Mater. 2025, 35, 2504261; Nano Lett. 2024, 24, 1254–1260]. Our experiments were conducted following these reported procedures. The specific parameters are as follows: a PMMA solution (0.5 mg/mL in anhydrous acetone) was spin-coated onto the pixelated QD arrays at 5000 rpm for 30 s, and subsequently annealed at 60 °C for 30 min in a nitrogen-filled glovebox.

It should be noted that, due to the fluidity of the liquid, the PMMA blocking layer is predominantly distributed in the inter-pixel gaps, with only a minimal residual layer present on top of the QD pixels [Ref: Org. Electron. 2022, 108, 106609]. Importantly, we did not overlook this issue; rather, we explicitly depicted this phenomenon in the schematic illustration of our original manuscript (see the inset of Figure 5i). We did not remove the residual PMMA on the QD surface, consistent with the practice commonly adopted in other studies in the field.

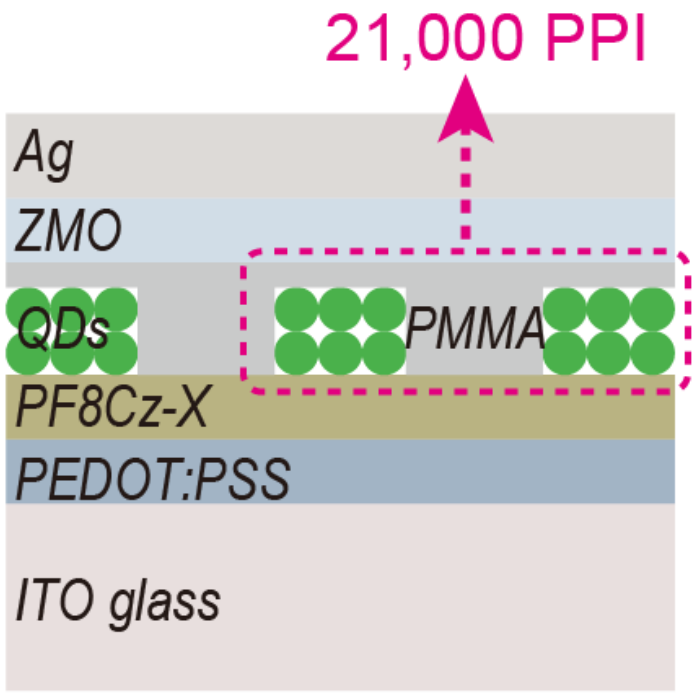

Figure 5i (inset). Schematic of nano-pixelated QLED with a PMMA layer.

As the reviewer rightly pointed out, the presence of PMMA on the QD surface could potentially affect charge injection, interfacial contact, and ultimately device performance. Therefore, the preparation parameters of the PMMA blocking layer require careful optimization [Ref: Org. Electron. 2022, 108, 106609]. Here, the abovementioned parameters have been systematically optimized for our system. Despite these efforts, the peak EQE of the fabricated nano-pixelated devices remains slightly lower than that of the non-patterned counterparts. One possible reason is the residual PMMA-induced difficulty in charge injection, which is consistent with your inference. In future work, we plan to explore alternative approaches, such as transfer printing or direct photolithography of PMMA, to confine the blocking layer exclusively to the inter-QD gaps, thereby minimizing its impact on device performance.

Thanks again for your meaningful comment. For better understanding, the PMMA parameters have been added in the revised Supplementary Information. And corresponding discussion and reference have been added in the revised manuscript also.

Change made on the second paragraph of Page 25 in the revised Supplementary Information: "For pixelated QLEDs, a PMMA solution (0.5 mg/mL in anhydrous acetone) was spin-coated on the pixelated QDs at 5000 rpm for 30 s, and subsequently annealed at 60 °C for 30 min. The PMMA layer is introduced to prevent current leakage caused by the direct contact between the hole transport layer and electron transport layer."

Change made on the last paragraph of Page 15: "we introduced a spin-coated PMMA layer as the blocking layer in as-fabricated ultrahigh-resolution nano-pixelated QLEDs. Due to the fluidity of the liquid, the PMMA blocking layer is predominantly distributed in the inter-pixel gaps.[67]"

Change made on the second paragraph of Page 16: "Note that the peak EQE of the nano-pixelated devices is slightly lower than that of the non-patterned ones. Potential contributing factors include the non-ideal pixel morphology at such a high resolution, the electric-field non-uniformity arising from pixel microstructures,[1] and the residual

PMMA-induced difficulty in charge injection.[67] Encouragingly, this value of 17.08% at 21,000 PPI remains highly competitive"

Change made on Page 21: "[67] Gao, H. J. et al. High-performance, high-resolution quantum dot light-emitting devices through photolithographic patterning. *Org. Electron.* **108**, 106609 (2022)."

Thank you again for your time and careful review of our manuscript. We sincerely believe that the manuscript has greatly benefited from your valuable suggestions.

To reviewer 3

Comment: *The revised manuscript is substantially improved, particularly by the addition of film-state spectroscopy and a decrosslinked 21,000-PPI QLED. But several issues still affect the central interpretation, I continue to recommend a revision.*

1.The manuscript renames the fitted quantity as thermal activation energy Ea but then states that its variation is predominantly linked to exciton binding energy Eb and reflects compromised exciton binding capability. This inference is not established. A thermal-quenching activation energy is not generally identical to Eb, and similar room-temperature fs-TA maps do not measure Eb or exclude trapping, localization, nonradiative pathways, energy transfer, or ligand-related dynamics. Unless an independent, diagnostic Eb measurement is provided, Ea should be treated as a phenomenological thermal-quenching parameter and the Ea-to-Eb/dielectric causal chain should be removed or explicitly labelled as a hypothesis.

Equation (1) is printed as I(t) = I0/[1 + A exp(Ea/kBT)]. The independent variable should be temperature, I(T), and the usual one-channel thermal-quenching form contains exp[-Ea/(kBT)]. Please determine whether this is a typographical error or the equation actually used for fitting. If the displayed positive sign was used, the fits and reported Ea values must be recalculated.

Response:

Dear reviewer, we sincerely appreciate your time and thorough review of our manuscript. We are also grateful for your encouraging feedback on our revisions. To address the remaining issues you have kindly pointed out, we have made further revisions.

First, we acknowledge the reviewer's point that, based on the current data, a definitive correlation between E_a and E_b cannot be unequivocally established. Accordingly, **the relevant discussion should be more appropriately framed as a hypothesis**. Specifically, temperature-dependent PL measurements on QD films during the reversible photolithography process reveal reversible changes in the thermal activation energy (E_a). On the other hand, given the substantially increased dipole moment and dielectric constant of coumarin after photocycloaddition [Ref: Adv. Funct. Mater. 2026, 36, e16451], the exciton binding energy (E_b) is expected to undergo reversible modulation—decreasing upon crosslinking and recovering upon decrosslinking—due to the change in the local dielectric

environment [Ref: ACS Energy Lett. 2023, 8, 731]. **We therefore infer that the observed variation in E_a is closely related to the change in E_b**, given their similar trends.

As a complement, we performed time-resolved transient absorption (TA) spectroscopy on the QD films (Supplementary Fig. 13 in the Supplementary Information). The absence of notable signal changes upon crosslinking and decrosslinking indicates that no significant energy transfer pathways are involved in the photochemical processes. Meanwhile, PL QYs of the QD films show only minor changes during the crosslinking and decrosslinking steps (Figure 3c in the manuscript), suggesting that these photochemical processes do not introduce appreciable surface defects. In this context, we hypothesize that the observed variation in E_a may be associated with the change in E_b.

Change made on the second paragraph of Page 9: "Notably, the decrosslinking process effectively recovered E_a to 96.37 meV, close to its original value, further demonstrating the low-damage nature of the reversible photolithography strategy."

Change made on the third paragraph of Page 9: "Given the substantially increased dipole moment and dielectric constant of coumarin after photocycloaddition,[54] the exciton binding energy (E_b) is expected to undergo reversible modulation—decreasing upon crosslinking and recovering upon decrosslinking—due to the change in the local dielectric environment.[55] We therefore infer that the observed variation in E_a is closely related to the change in E_b, given their similar trends. In this context, the reduced E_a after crosslinking reflects a compromised exciton binding capability, which ultimately impairs device performance, as confirmed by subsequent experiments. As a complement, we performed time-resolved transient absorption (TA) spectroscopy on the QD films (Supplementary Fig. 13). The lack of notable signal changes upon crosslinking and decrosslinking—similar to the trend observed in PL QYs (**Figure 3c**)—suggests that surface defects and energy transfer pathways do not play a dominant role in the photochemical processes."

Finally, as the reviewer correctly pointed out, a minus sign was missing from the formula. This is a typographical error, and we confirm that all values presented in this work were calculated using the correct formula.

2.The reported 107.4% PLQY 'retention' is normalized to OA-GQD, although PRL-GQD is the actual pre-photolithography state. From Fig. 3c, the process retention is 107.4/111.6 = approximately 96.2%. Under ambient conditions, it is 91.8/110.2 = approximately 83.3%, not 91.8%. The text and Supplementary Table 1 should distinguish PLQY relative to the OA feedstock from retention through photolithography and should use the latter for process

comparisons. Likewise, Fig. 3f reports raw current in pA, not conductivity, and the decrosslinked current does not return to the PRL value.

Finally, approximately 1 mJ cm^{-2} retains only about 62% of the PL and about 59% of the saturated film thickness, whereas near-saturation requires approximately 5 mJ cm^{-2}. Thus, 1 mJ cm^{-2} is an onset dose rather than a demonstrated dose-to-clear or dose-to-size. The sensitivity comparison should use a defined endpoint and the actual dose used for the high-resolution patterns/devices.

Response:

First, we sincerely thank the reviewer for this critical and constructive comment regarding the PLQY retention calculation. We would like to clarify that in the field of direct QD photolithography, **it is a widely adopted practice to use pristine QDs without photosensitive ligands/additives** (e.g., OA-capped QDs) **as the reference for calculating PLQY retention** [Ref: Nano Lett. 2025, 25, 10435; Adv. Funct. Mater. 2025, 35, 2420829; Small Methods 2023, 7, 2300206]. This is because the introduction of the photosensitive ligand/additive is itself considered an integral part of the photolithographic process, rather than a separate treatment. Accordingly, the 107.4% PLQY retention reported in our work was calculated relative to the pristine OA-capped QDs (OA-GQD). This choice was made to enable a fair comparison with previously reported values in the literature.

However, we fully agree with the reviewer that it is also informative to evaluate the retention relative to the actual pre-photolithography state, i.e., the PRL-GQD. Following this suggestion, we have now calculated the PLQY retention based on PRL-GQD as the reference. And the values under glovebox conditions and ambient conditions are 96.2% and 83.3%, respectively. **We have incorporated these additional calculations into the revised manuscript to provide a more comprehensive assessment**.

Furthermore, to avoid any potential misunderstanding, **we have also explicitly noted in Supplementary Table 1** that the 107.4% value and the 96.2% value represent the PLQY retention relative to the pristine OA-GQD and PRL-GQD, respectively.

Change made on the first paragraph of Page 8: “Here, the PLQY retention after the complete reversible photolithography process remains as high as 96.2% even when referenced to PRL-GQD, indicating minimal photochemical damage of the QDs.”

Change made on the last paragraph of Page 12: "Although the PLQY retention after reversible photolithography under ambient conditions is relatively lower (~91.8% relative to OA-GQD; ~83.3% relative to PRL-GQD) than that achieved in the nitrogen-filled glovebox"

Second, we thank the reviewer for the constructive suggestion regarding the conductivity analysis. The measurement protocol was established based on previously reported methods in the field [Ref: Nat. Commun. 2024, 15, 6561], serving as an indirect approach to evaluate changes in the conductivity of QD films. We fully agree with the reviewer that the obtained results represent current-voltage characteristics rather than direct measurements of conductivity. Accordingly, we have revised the description in the manuscript to the more accurate term "**current-voltage analysis**." In addition, the discussion regarding conductivity has been appropriately moderated throughout the manuscript to avoid overinterpretation.

It is worth noting that, **given the identical electrode configuration and the same QD film geometry, differences in the measured current can still reflect trends in resistance and thus conductivity**. Specifically, the lower current observed in the crosslinked QD film indicates higher resistance and reduced conductivity. We have incorporated the corresponding revisions in the revised manuscript accordingly. We thank the reviewer again for this valuable suggestion.

Change made on the last paragraph of Page 8: "Given the identical electrode configuration and film geometry, the lower current indicates higher resistance and thus reduced conductivity."

Third, we thank the reviewer for this careful and important comment regarding the UV exposure dose. We fully agree that 1 mJ cm^{-2} just represents an onset dose, whereas near-saturation is achieved at approximately 5 mJ cm^{-2}. In fact, the 5 mJ cm^{-2} dose was used as the typical exposure condition for all photolithographic devices and patterns. We acknowledge that this was not clearly stated in the previous manuscript, which may have caused confusion. To address this issue, **we have now explicitly specified in Supplementary Table 1**.

In addition, **we have added a note in the revised manuscript indicating that, unless otherwise stated, the 5 mJ cm^{-2} dose serves as the default condition**. We believe these revisions provide a clearer and more accurate presentation of our experimental conditions.

Change made on the third paragraph of Page 17: "Unless otherwise stated, all experiments were performed in a nitrogen-filled glove box by default, with a UV dose of ~5 mJ/cm^2."

3.The film-state FT-IR and UV-vis data support a reversible change of the coumarin chromophore, which is a useful addition. They do not, by themselves, provide product-specific proof of cyclobutane formation or show that inter-QD reaction dominates over intra-QD reaction. In Supplementary Fig. 7, a dense film and a 0.1 mg mL^{-1} dispersion are compared using raw Delta Abs, although their initial coumarin absorbance, optical path, scattering, local concentration, and absorbed photon number are not equivalent. Faster change in the film is therefore consistent with a proximity-enhanced inter-QD contribution but does not confirm its dominance. If the authors do not provide a normalized or more discriminating experiment, the mechanistic language should be revised to this qualified conclusion.

Response:

Dear reviewer, we thank you for your positive recognition of our supplementary film-state FT-IR and UV-vis data.

First, we fully agree with the reviewer that our FT-IR and UV-vis data, while supporting the reversible change of the coumarin chromophore, do not by themselves provide definitive product-specific proof of cyclobutane ring formation. We acknowledge that a definitive confirmation would ideally require additional evidence such as solid-state NMR. However, in our system, the coumarin ligands are present only at the QD surface with relatively low loading, and the measurements are performed on ultrathin solid films with thicknesses on the order of tens of nanometers. This makes it technically challenging to obtain interpretable solid-state NMR signals from the QD surface ligands.

We have therefore relied on FT-IR spectroscopy as the primary characterization tool. As far as we know, **the use of FT-IR spectroscopy to monitor the reversible change of coumarin chromophores**—specifically, the decrease of the C=C stretching band upon photodimerization and its recovery upon photocleavage—**is a well-established practice in the field for inferring cyclobutane ring formation** [Ref: Adv. Funct. Mater. 2020, 30, 1909359; J. Mater. Chem. 2011, 21, 18373].

Nevertheless, we agree with the reviewer that FT-IR data alone do not constitute definitive proof. To address this concern, **we have revised the relevant discussion in the manuscript to reflect a more qualified conclusion**, avoiding any overstatement regarding the definitive identification of the cyclobutane product.

Change made on the second paragraph of Page 7: "In-situ Fourier transform infrared (FT-IR) spectroscopy was employed to monitor the switching of coumarin ligands during the photochemical reactions (Supplementary Fig. 5). Upon 310 nm UV exposure, the signal at 1614 cm^{-1}, corresponding to the C=C stretching vibration, gradually decreased, consistent with the consumption of coumarin groups during crosslinking[45,46]. Subsequently, upon DUV irradiation, this signal gradually recovered, reflecting the restoration of coumarin groups through the decrosslinking process."

Change made on Page 20: "[45] Lu, D., Zhu, M., Wu, S., Lian, Q., Wang, W., Adlam, D., Hoyland, J. A. & Saunders, B. R. Programmed multiresponsive hydrogel assemblies with light-tunable mechanical properties, actuation, and fluorescence. *Adv. Funct. Mater.* **30**, 1909359 (2020).

[46] Ling, J., Rong, M. Z. & Zhang, M. Q. Coumarin imparts repeated photochemical remendability to polyurethane. *J. Mater. Chem.* **21**, 18373 (2011)."

Second, we also acknowledge the reviewer's point that the comparative kinetics between the dense film and the dilute dispersion, as presented in the previous Supplementary Fig. 7, do not by themselves provide definitive evidence for the dominance of inter-QD crosslinking over intra-QD crosslinking. We fully agree that the experimental conditions (e.g., optical path, scattering, local concentration, and absorbed photon number) may influence the observed kinetics and thus complicate the interpretation.

It should be noted, however, that in our experiments **we made efforts to control the film thickness and solution concentration, and we performed normalized corrections on the measured data to ensure comparable coumarin absorption levels**, thereby minimizing the disparity in the number of coumarin units in the optical path. The original data is shown in Figure R1a. Here, the QD film and the solution (0.02 mg/mL in toluene, rather than 0.1 mg/mL; this is a typo error that has been corrected) exhibit similar UV absorption spectral profiles and intensities, justifying a comparative analysis of their absorbance changes. To further improve the comparability of the results, we also normalized the data (Figure R1b), which clearly reveal the rapid structural switching of the coumarin ligands in the film state. **This observation strongly supports our inference regarding the significant contribution of the inter-QD pathway in the QD film**.

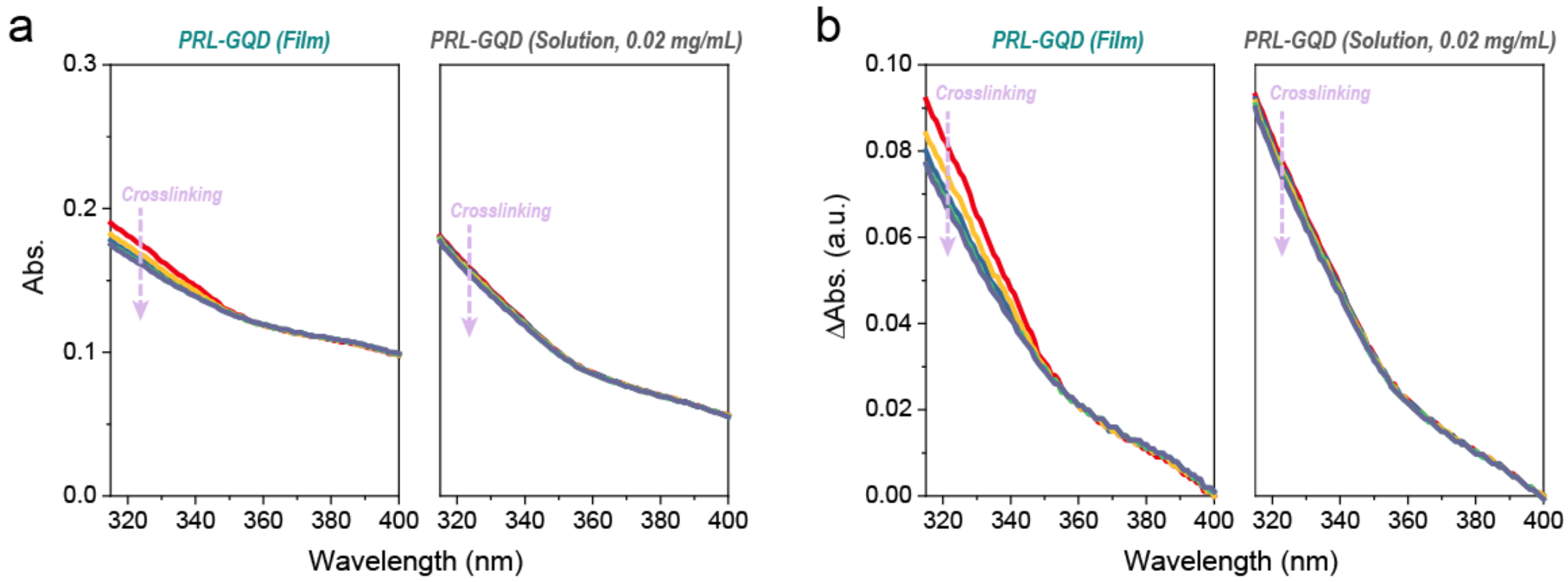

Figure R1. (a) UV-Vis absorption of PRL-GQD film (left) and solution (0.02 mg/mL in toluene) (right) under UV exposure (centered at 310 nm, ~1 mW/cm^2) from 0 s to 35 s. (b) Normalized relative UV-Vis absorption of PRL-GQD film (left) and solution (0.02 mg/mL in toluene) (right) under UV exposure (centered at 310 nm, ~1 mW/cm^2) from 0 s to 35 s.

In addition, we have supplemented the analysis with measurements on a relatively concentrated solution of photosensitive QDs (0.10 mg/mL in toluene) under UV irradiation. As shown in Figure R2, **the relative absorbance changes in the concentrated solution are more pronounced than those in the above dilute solution** (Figure R1), **exhibiting a clear concentration dependence—a characteristic feature of the inter-QD pathway**. Moreover, even in the concentrated solution, which contains more molecules in the optical path and exhibits stronger absorption, the observed relative changes remain weaker than those in the film, further indicating that the inter-QD contribution is more significant in the film state.

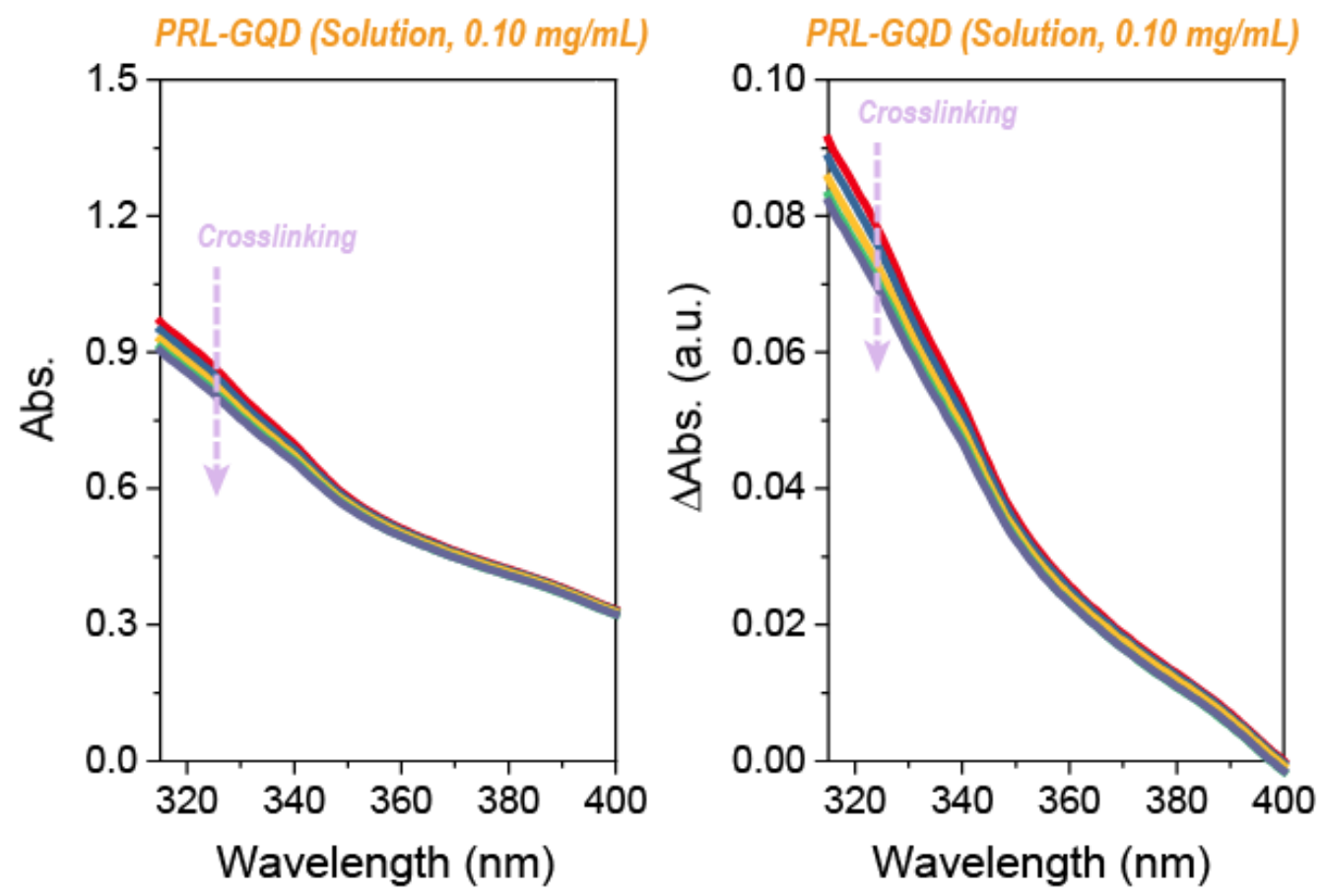

Figure R2. UV-Vis absorption (left) and normalized relative UV-Vis absorption (right) of PRL-GQD solution (0.10 mg/mL in toluene) under UV exposure (centered at 310 nm, ~1 mW/cm^2) from 0 s to 35 s.

Nonetheless, we fully agree with the reviewer that the faster photoreaction kinetics observed in the film state, while suggestive of a proximity-enhanced inter-QD contribution, do not by themselves confirm its quantitative dominance over the intra-QD pathway. To address this concern more rigorously, **we have revised the mechanistic discussion in the revised manuscript and have included the additional data** in Supplementary Fig. 7. We believe these revisions provide a more accurate and qualified interpretation of our data while appropriately acknowledging the limitations of the current evidence. We are grateful to the reviewer for helping us improve the scientific rigor of our mechanistic discussion.

Change made on the third paragraph of Page 7: "To assess the contribution of photo-crosslinking between ligands on neighboring QDs (the inter-QD pathway) to reversible QD photolithography, we examined the crosslinking kinetics of QD solutions at different concentrations (0.10 mg/mL and 0.02 mg/mL). As shown in Supplementary Fig. 7, the normalized relative absorbance changes show a clear increase with rising QD concentration—a concentration-dependent trend that is characteristic of the inter-QD pathway. More importantly, under the same irradiation conditions, the QD film exhibits larger relative absorbance changes, indicating a significant inter-QD crosslinking contribution arising from the close proximity of neighboring QDs."

Change made on Page 5 in the revised Supplementary Information:

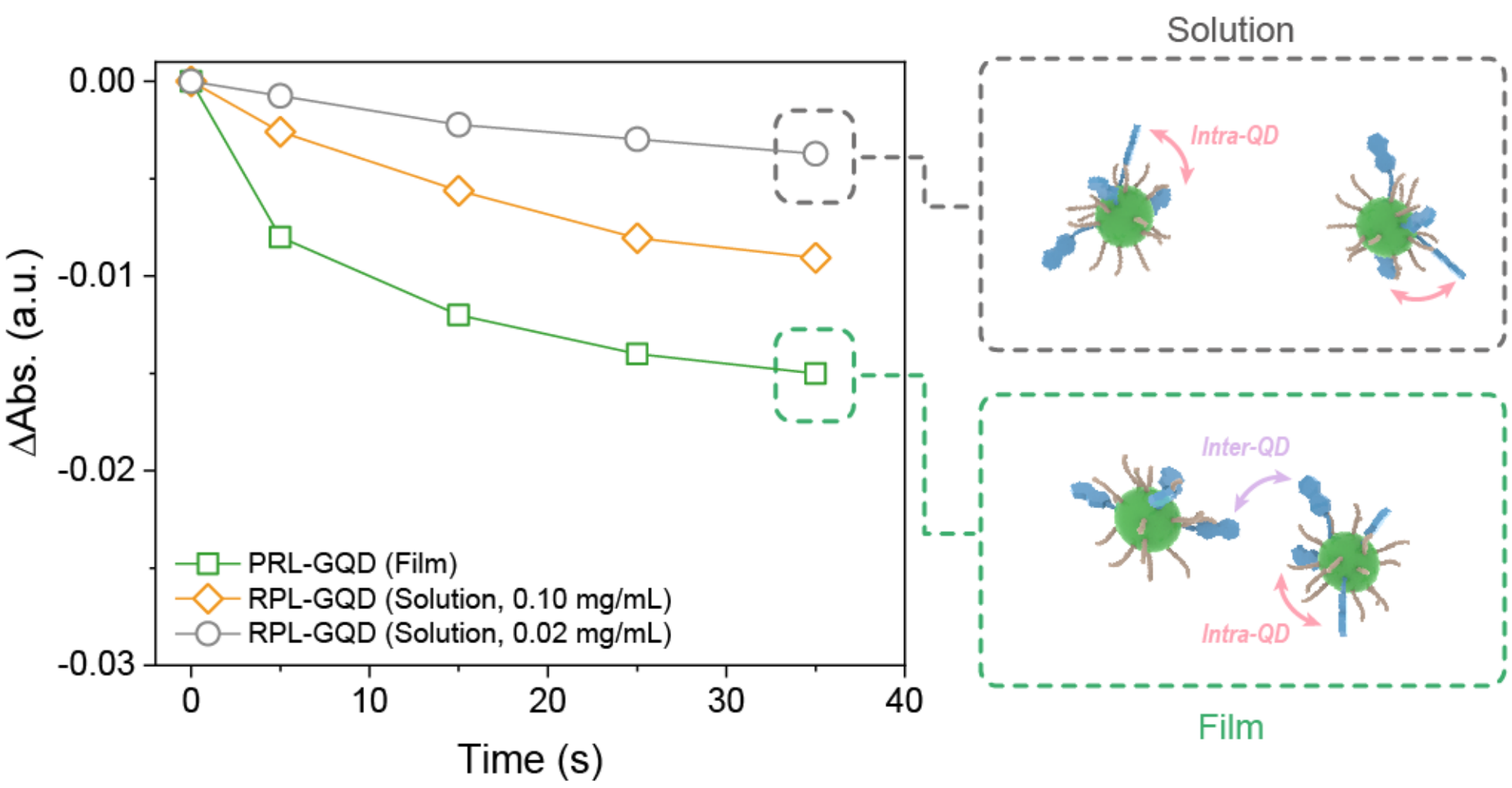

Supplementary Fig. 7 Left: normalized relative absorption changes at 318 nm of PRL-GQD film and PRL-GQD solution (0.10 mg/mL and 0.02 mg/mL in toluene) under UV exposure (centered at 310 nm, ~1 mW/cm^2). Right: potential intra-QD and inter-QD pathways in these systems.

4.A feature width of approximately 0.8 micrometres is not the pitch corresponding to 21,000 PPI; 21,000 PPI corresponds to a center-to-center pitch of approximately 1.21 micrometres. Width, pitch, gap, and the PPI conversion should be stated separately.

More importantly, current density and EQE for a discontinuous emissive array depend on whether the denominator is the total electrode area or the summed emitting-pixel area, while luminance depends on the sampled optical area and pixel fill factor.

Response:

Dear reviewer, we sincerely thank you for raising such a meaningful and constructive comment.

We would like to clarify that **the pixel size (~ 0.8 µm) mentioned in our previous manuscript refers to the pixel width, rather than the pixel pitch**. To provide a clearer presentation of the pixel geometry in our nano-pixelated QLEDs, we have now included additional electroluminescence (EL) microscopy image. As shown in Figure R3, the pixel array maintains a pixel width of 0.8 µm, a pixel gap of 0.41 µm, and a pixel pitch of 1.21 µm, corresponding to a pixel density of ~21,000 PPI. Importantly, the introduction of the PMMA layer does not affect the geometry of the QD pixels.

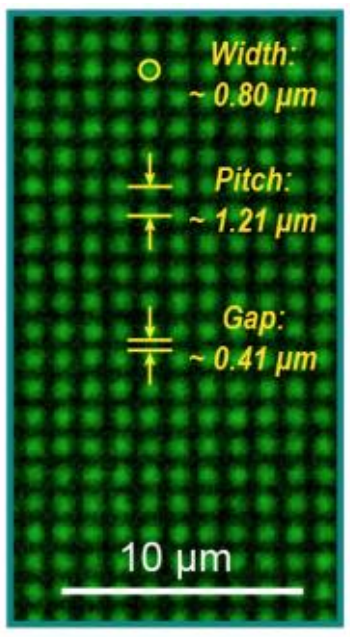

Figure R3. EL microscopic image (a) of nano-pixelated QLED based on decrosslinked PRL-GQD. A PMMA blocking layer is introduced to prevent current leakage caused by direct contact between the hole transport layer and the electron transport layer.

We have incorporated the relevant information into the revised manuscript and Supplementary Information to ensure a more accurate presentation of our results. Moreover, the description in Figure 5h has been revised also.

Change made on the last paragraph of Page 15: "As shown in Supplementary Fig. 24, the EL microscopy image reveals that the introduction of PMMA does not affect the geometry of QD pixels. The pixel array maintains a pixel width of 0.8 µm, a pixel gap of 0.41 µm, and a pixel pitch of 1.21 µm, corresponding to a pixel density of ~ 21,000 PPI."

Change made on Page 13 in the revised Supplementary Information:

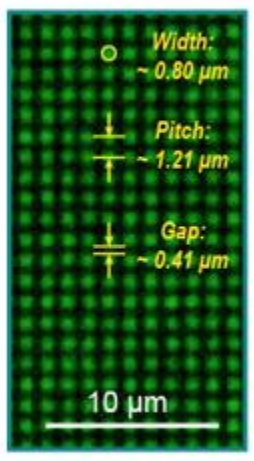

Supplementary Fig. 24 (a) EL microscopic image of nano-pixelated QLED based on decrosslinked PRL-GQD.

On the other hand, we thank the reviewer for raising this meaningful comment regarding the area definitions for luminance, current density, and EQE in discontinuous pixel arrays. This issue is of critical importance for high-resolution QLEDs, yet it is often overlooked by researchers. We fully agree with the reviewer's assessment. In the previous manuscript, **the reported luminance and current density values represent statistically averaged (area-averaged) results derived from instrument outputs**, **rather than the true values of individual emitting pixels**. Direct monitoring of microscopic pixel performance remains challenging at this stage; therefore, this approach is widely adopted as a standard practice in the field.

To obtain the actual luminance and current density, one must divide the measured total luminous flux and total current by the summed emitting-pixel area (i.e., multiplied by 1/FF, where FF is the pixel fill factor). In the present work, the FF is estimated to be approximately 0.34, as determined from the EL microscopy image shown in Figure R3. Notably, as the reviewer may recognize, the EQE value itself is independent of the area definition because the area terms cancel out in the calculation when using the raw total flux and total current. Thus, **the reported EQE remains numerically valid** regardless of whether the electrode area or the emitting area is used, as long as the input data are the raw measured values.

To avoid any ambiguity for the readers, we have added a clear note in the revised manuscript stating that: (1) the reported luminance and current density are instrument-derived, statistically averaged values based on the total electrode area; (2) these values should be corrected by the pixel fill factor when a true comparison with continuous-film devices is desired; and (3) the EQE values are unaffected by this correction. We believe this clarification adequately addresses the reviewer's concern without altering the original data presentation.

Change made on Page 13 in the revised Supplementary Information:

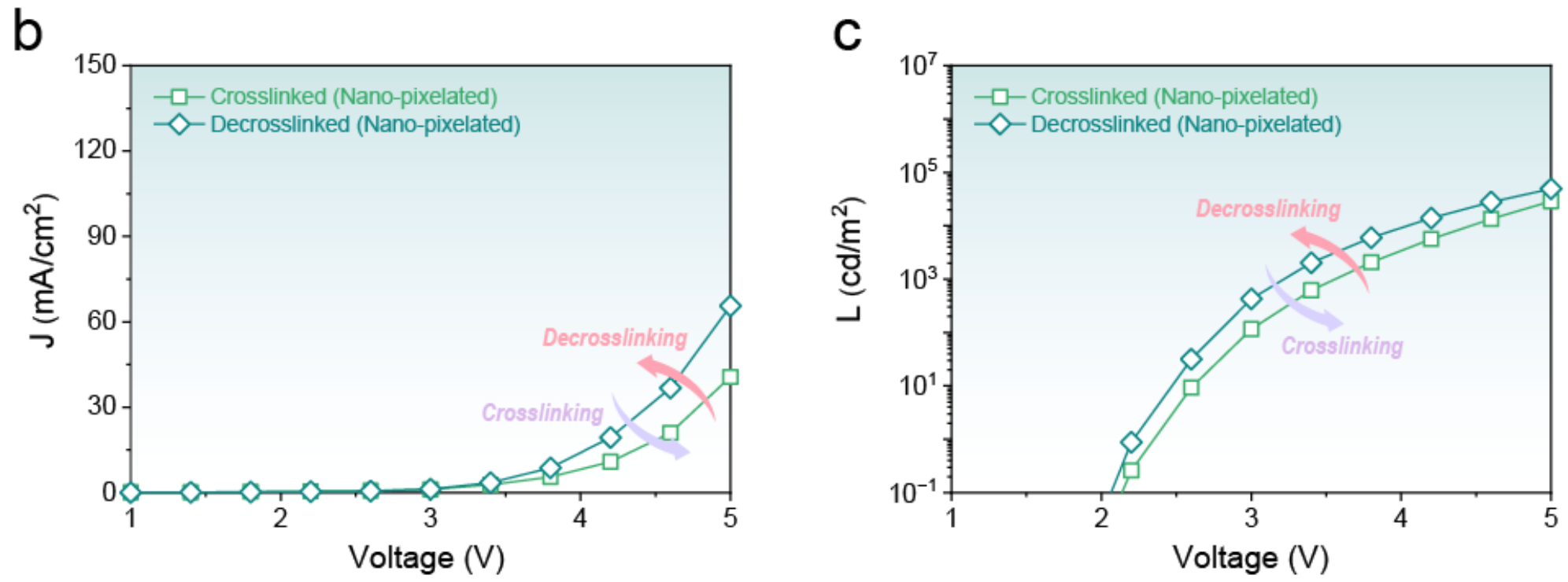

Supplementary Fig. 24 (b-c) *J–V* curves (b) and *L–V* curves (c) of nano-pixelated QLEDs based on crosslinked PRL-GQD and decrosslinked PRL-GQD. Note that the *J* and *L* in (b-c) are instrument-derived, statistically averaged values based on the total electrode area, and these values should be corrected by the pixel fill factor (FF).

Change made on the last paragraph of Page 21 in the revised Supplementary Information: "Note that the reported luminance and current density for pixelated QLEDs are instrument-derived, statistically averaged values based on the total electrode area, rather than the true values of the individual emitting pixels. And these values should be corrected by the pixel fill factor (FF). The EQE values, however, remain unaffected by this correction."

We appreciate the reviewer's careful examination and hope this response is satisfactory.

5.Supplementary Note 7 places PMMA coating after preparation of decrosslinked pixels but does not identify the solvent. Because the decrosslinked QDs are reported to regain solubility in nonpolar solvents, the authors should identify the solvent and verify that the approximately 0.8-micrometre features remain after PMMA coating.

Response:

Dear reviewer, we sincerely thank you for raising such a critical question. As you rightly pointed out, the solvent used for PMMA should not dissolve the decrosslinked QDs (as PRL-GQD), so as to avoid any damage to QD pixels during spin-coating. To address this, we carefully selected **anhydrous acetone as the solvent for PMMA**. As shown in Figure R4, this solvent does not degrade the PRL-GQD film. Therefore, the integrity of QD pixels is well preserved during the process. The electroluminescence microscopy images of the resulting nano-pixelated QLEDs have been provided in our response to your previous comment.

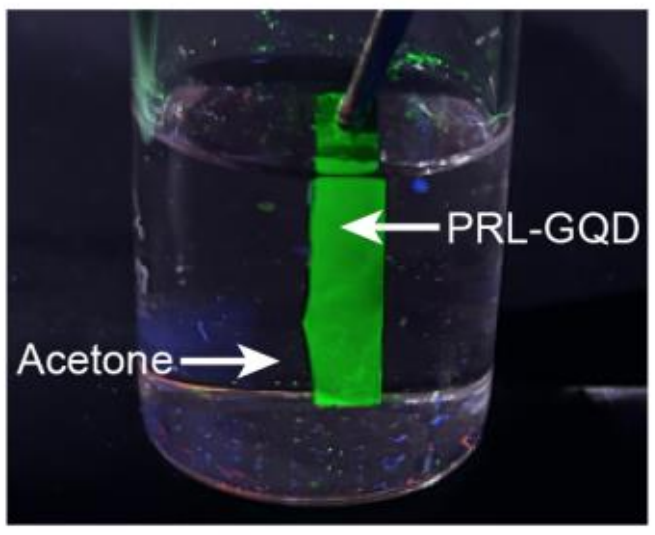

Figure R4. Fluorescent photo of PRL-GQD film immersed in acetone.

Thanks again for this comment, which made us realize that the experimental details regarding the spin-coated PMMA layer were not fully provided. Here, we specify the parameters: a PMMA solution (0.5 mg/mL in anhydrous acetone) was spin-coated onto the pixelated QD arrays at 5000 rpm for 30 s, and subsequently annealed at 60 °C for 30 min in a nitrogen-filled glovebox. We have now supplemented the relevant description in the revised Supplementary Information.

Change made on the second paragraph of Page 25 in the revised Supplementary Information: "For pixelated QLEDs, a PMMA solution (0.5 mg/mL in anhydrous acetone) was spin-coated on the pixelated QDs at 5000 rpm for 30 s, and subsequently annealed at 60 °C for 30 min. The PMMA layer is introduced to prevent current leakage caused by the direct contact between the hole transport layer and electron transport layer."

Thank you again for your time and careful review of our manuscript. We sincerely believe that the manuscript has greatly benefited from your valuable suggestions.

To reviewer 4

Comment: *Direct photolithography have attracted much attention as a photoresist-free route for high-precision integration in electronic and optoelectronic platforms. The paper reports a reversible photolithography strategy to achieve high-efficient and high-fidelity QD photolithography. In the revised version, the authors have addressed all the comments I am concerned, and now it can be accepted in the current version.*

Response:

Dear reviewer, thanks a lot for your time and efforts to review our manuscript. We also appreciate your positive recognition. We believe that with the help of you and other experts, the quality of our work has been significantly improved.

Reversible photolithography of quantum dots achieves high-performance electroluminescence

(Manuscript ID: NCOMMS-26-047874B)

A Point-by-point Response to the Reviewers' Comments

To reviewer 2

Comment: *My concerns are all addressed.*

Response:

Dear reviewer, thanks a lot for your time and efforts to review our manuscript. We believe that with the help of you and other experts, the quality of our work has been significantly improved.

To reviewer 3

Comment: *The manuscript has been further improved, and the authors have satisfactorily addressed the pixel geometry and PMMA-processing questions.*

Response:

Dear reviewer, we sincerely appreciate your time and thorough review of our manuscript. We are also grateful for your encouraging feedback on our revisions. We sincerely believe that the manuscript has greatly benefited from your valuable suggestions.
